# Rational Design and Synthesis of [5]Helicene-Derived Phosphine Ligands and Their Application in Pd-Catalyzed Asymmetric Reactions

**DOI:** 10.1038/srep36211

**Published:** 2016-11-08

**Authors:** Kosuke Yamamoto, Takashi Shimizu, Kazunobu Igawa, Katsuhiko Tomooka, Go Hirai, Hiroshi Suemune, Kazuteru Usui

**Affiliations:** 1 Graduate School of Pharmaceutical Sciences, Kyushu University, Fukuoka, 812-8582, Japan; 2 Institute for Materials Chemistry and Engineering, Kyushu University, Fukuoka, 816-8580, Japan

## Abstract

A series of novel optically active [5]helicene-derived phosphine ligands (**L1**, with a 7,8-dihydro[5]helicene core structure- and **L2**, with a fully aromatic [5]helicene core structure) were synthesized. Despite their structural similarities, **L1** and **L2** exhibit particularly different characteristics in their use as chiral ligands. **L1** was highly effective in the asymmetric allylation of indoles with 1,3-diphenylallyl acetate (up to 99% ee), and in the etherification of alcohols (up to 96% ee). In contrast, **L2** was highly effective in the stereocontrol of helical chirality in Suzuki–Miyaura coupling (SMC) reaction (up to 99% ee). Density functional theory analysis was employed to propose a model that accounts for the origin of the enantioselectivity in these reactions.

The rational design and development of new chiral ligands to enable stereocontrol in a wide variety of reactions is one of the most important topics in modern organic synthesis^1^,^2^. To date, chiral ligands containing heteroatoms with strong σ-donating properties, such as phosphorus and nitrogen have been commonly utilized in transition-metal-catalyzed asymmetric reactions. In addition, chiral ligands containing π-donating alkenes and arenes have attracted increasing attention in recent years^3^,^4^,^5^,^6^. More recently, the combination of these two features, i.e., the development of hybrid ligands containing both σ-donating and π-donating groups, has received growing attention, and unprecedented reactivity and stereoselectivity have been observed (Fig. 1a)^7^,^8^,^9^,^10^,^11^,^12^,^13^,^14^,^15^. Thus, to develop novel and efficient hybrid chiral ligands, we envisage that helicene would be a suitable π-donating group to efficiently construct a helical environment around a metal center (Fig. 1b).

Helicenes are nonplanar screw-shaped polycyclic compounds based on ortho-condensed benzene or other aromatic rings, which exhibit unique structural, optical, and electronic features. Thus, helicenes can be utilized them a broad range of applications in chiral materials, in the chiral recognition of biomolecules, and in asymmetric synthesis^16^,^17^,^18^,^19^,^20^,^21^,^22^,^23^,^24^. More specifically, chiral helicene-based trivalent phosphorus ligands are efficient asymmetric inductors in metal-catalyzed asymmetric reactions and show good to excellent enantioselectivities^25^,^26^,^27^,^28^,^29^,^30^,^31^,^32^,^33^. We hypothesized that novel helicenylphosphine ligands, which induce intramolecular metal-arene interactions, could be used to construct an efficient chiral catalytic system. To realize this concept, we designed two types of [5]helicene-derived phosphine ligands (**L1**, with a 7,8-dihydro[5]helicene core structure; and **L2**, with a fully aromatic [5]helicene core structure) as rationally simplified molecules (Fig. 1b). Unlike typical [5]helicenes that are conformationally unstable and tend to racemize^34^,^35^, **L1** and **L2** are expected to exhibit stable chirality, as helix inversion is prevented by steric hindrance from the phosphine group at the C1 position^36^,^37^,^38^. Furthermore, the helicene backbones in these ligands may affect the electronic structures of the chelating substrate around the metal, due to different helical pitches and conformational flexibility.

## Results and Discussion

### Syntheses and structural characterization of L1 and L2

**Figure 2:**
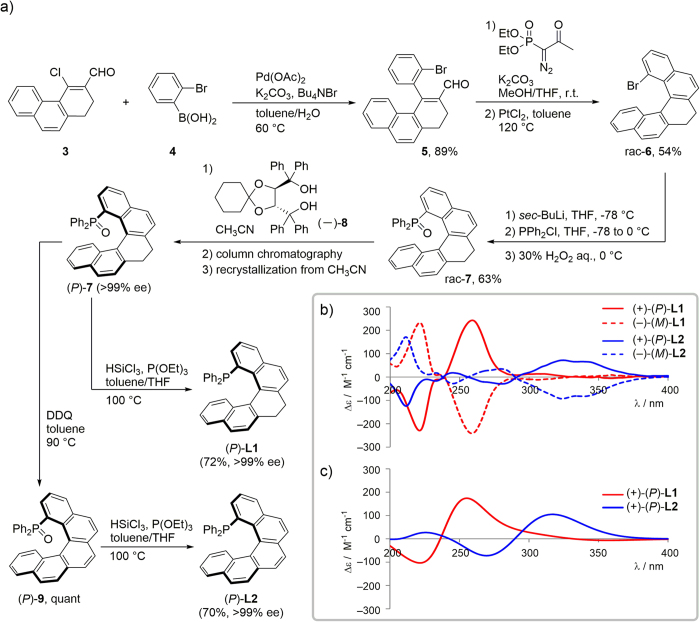
(**a**) Preparation of chiral phosphines **L1** and **L2**. (**b**) ECD spectra of (*P*)-**L1** (red solid line), (*M*)-**L1** (red dashed line), (*P*)-**L2** (blue solid line), and (*M*)-**L2** (blue dashed line) in acetonitrile (1.0 × 10^−5^ M). (**c**) Calculated (CAM-B3LYP/6-31 + G**//B97-D/6-31G*) ECD spectra of (*P*)-**L1** (red solid line) and (*P*)-**L2** (blue solid line).

Ligands **L1** and **L2** were synthesized via the route outlined in Fig. 2a. The Suzuki–Miyaura coupling (SMC) of 4-chloro-3-formy-l,2-dihydrophenanthrene **3** with 2-bromophenylboronic acid **4** was achieved in a chemoselective manner to give coupling product **5** in 89% yield^37^. Subsequent Ohira–Bestmann modification of the Seyferth–Gilbert homologation followed by cycloisomerization with 10 mol% PtCl_2_ resulted in the formation of (±)-1-bromo-7,8-dihydro[5]helicene rac-**6** (54% yield over two steps). Lithiation of rac-**6** and subsequent reaction with chlorodiphenylphosphine and treatment with hydrogen peroxide afforded the (±)-phosphine oxide (rac-**7**) in 63% yield. The optical resolution of rac-**7** was successfully achieved by application of the Keglevich procedure using spiro-TADDOL (−)-**8** as a resolving agent^39^ to afford optically pure (*P*)-**7**. Enantiomer (*M*)-**7** could also be prepared using (+)-**8** (see Supplementary Information). Phosphine oxide (*P*)-**7** was converted into the desired **L1** using trichlorosilane and P(OEt)_3_ in 72% yield, while ligand **L2** was prepared in 70% yield over two steps by the oxidative aromatization of (*P*)-**7** with 2,3-dichloro-5,6-dicyano-1,4-benzoquinone (DDQ), and subsequent reduction of (*P*)-**9**.

To determine the absolute configuration of the resolved enantiomers of **L1** and **L2**, electronic circular dichroism (ECD) spectroscopy was carried out to give the spectra shown in Fig. 2b. In all cases (within experimental errors), mirror-image plots were displayed for the (+) and (−) enantiomers. The ECD spectrum of (+)-**L2** showed two bands with a first positive band at ~325 nm and a second negative band at ~280 nm, indicating *P* helicity (opposite signs were observed for *M* helicity). These results are in agreement with the absolute configurations previously reported for 1-funcionalized [5]helicenes^37^. We also calculated the ECD spectra of (*P*)-**L1** and -**L2** to compare them with the experimental ECD spectra (Fig. 2c). The ligand structures were optimized using the B97-D/6-31G* level of theory, and the ECD spectra were calculated using the time-dependent density functional theory (TDDFT) method at the CAM-B3LYP/6-31 + G** level of theory with SMD acetonitrile solvation. Indeed, in the calculated spectra, cotton effects were observed at >200 nm, with intensity patterns similar to those experimentally observed for (*P*)-**L1** and -**L2**.

**Figure 3:**
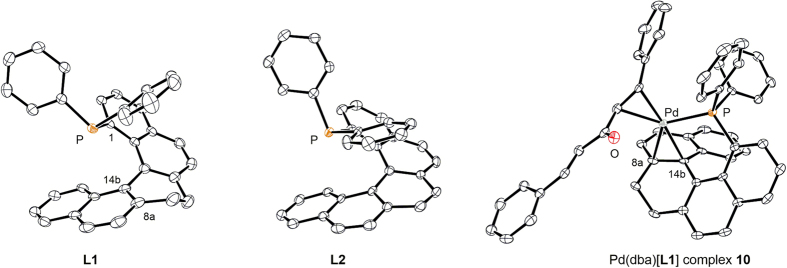
ORTEP drawings of L1, L2, and Pd(dba)[L1] complex 10 with 50% ellipsoid probability.

The structures of **L1** and **L2** were confirmed by X-ray crystallography (Fig. 3). Based on the obtained structures of **L1** and **L2**, the helical pitch diameter of **L1** (3.54–3.50 Å) is longer than that of **L2** (3.39–3.34 Å). To obtain further information regarding the phosphine-metal-arene interaction of the metal complex of **L1**, we prepared Pd(dba)[**L1**] complex **10** from Pd_2_(dba)_3_·CHCl_3_ and **L1**. As expected, results from X-ray crystallography showed that the double bond (C8a–C14b) of the helicene ligand was coordinated with the palladium center in a side-on (η^2^) fashion.

### Pd-Catalyzed asymmetric allylic substitutions

**Figure 4:**
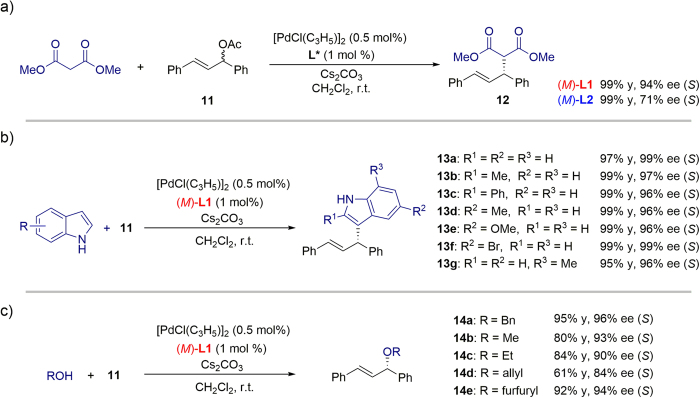
Pd-catalyzed asymmetric allylic substitution reactions.

With the phosphine ligands in hand, we investigated their effectiveness in Pd-catalyzed asymmetric allylic substitution reactions, as the mechanism of these reactions is fairly well understood^40^,^41^,^42^. As a model reaction, we studied the alkylation of racemic 1,3-diphenylallyl acetate **11** with dimethyl malonate, using Cs_2_CO_3_ as the base and [PdCl(C_3_H_5_)]_2_ (0.5 mol%) as the palladium source, in the presence of a catalytic amount of **L1** or **L2** (1 mol%) in CH_2_Cl_2_ at room temperature (Fig. 4a). Ligand (*M*)-**L1** was highly effective in this reaction, affording (*S*)-**12** in 99% yield with 94% ee, while (*M*)-**L2** afforded (*S*)-**12** in 99% yield with only 71% ee.

Encouraged by the promising results obtained with **L1**, the asymmetric allylation of indoles using **11** was subsequently investigated. In contrast to commonly studied nucleophiles, the Pd-catalyzed asymmetric allylation of indoles has been met with very limited success^10^,^43^. As shown in Fig. 4b, all reactions of indoles bearing substituents on the 2-, 3-, 5-, and 7-positions proceeded efficiently to give the desired products (**13a-g**) in excellent yields (95–99%) and enantioselectivities (96–99%) under optimized reaction conditions (see Table S1, Supplementary Information). To the best of our knowledge, this is the most efficient catalytic asymmetric allylation of indoles with **11** reported to date. **L1** was also effective in the Pd-catalyzed allylic etherification of alcohols to give the desired products (**14a-e**) in high yields (61–95%) and enantioselectivities (84–96%, Fig. 4c).

**Figure 5:**
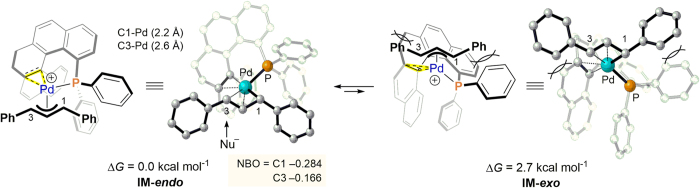
Calculated structures (DFT) of the (π-ally) palladium intermediates with (*M*)-L1 and their relative energies of formation.

To elucidate the stereoselectivity of the *exo* and *endo* (π-allyl)palladium intermediates (**IM-*****exo*** and **IM-*****endo***), DFT calculations using the B3PW91/6-31G* level of theory (LANL2DZ for the Pd atoms) were performed for geometry optimization. Figure 5 shows their calculated structures and relative energies of formation, with **IM-*****endo*** being the most stable based on these calculations. Indeed, the **IM-*****endo*** intermediate is favored because of the reduced steric repulsion between the helicene backbone and the allylic group. Assuming that nucleophilic attack on the allyl complex takes place at the allylic carbon atom (C3) due to the larger *trans* effect of phosphorus^44^, **IM-*****endo*** affords products with *S* configuration. This is clearly reflected in the computed lower natural bond orbital (NBO)-charge of the C3 position.

### Pd-Catalyzed asymmetric SMC

**Figure 6:**
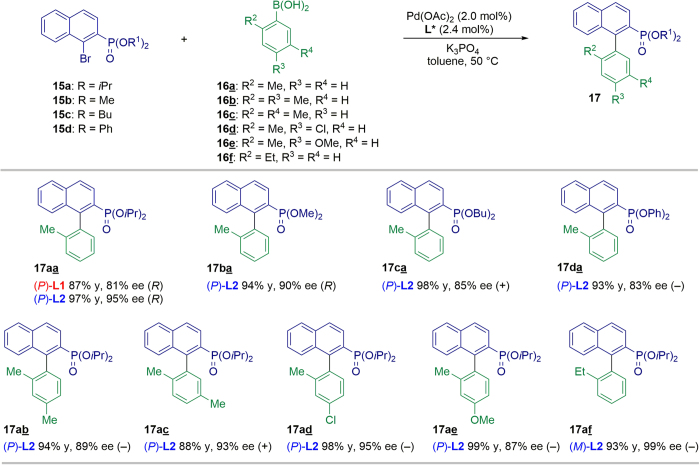
Pd-catalyzed asymmetric SMC using (*P*)-L2.

We then investigated the performance of **L1** and **L2** in the asymmetric SMC reaction^45^,^46^,^47^,^48^,^49^,^50^,^51^ between diisopropyl (1-bromonaphthalen-2-yl) phosphonate **15a** and *o*-tolylboronic acid **16a** under optimized reaction conditions (Pd(OAc)_2_/L*/toluene/50 °C, see Table S3, Supplementary Information). As shown in Fig. 6, in contrast to the results obtained with (*P*)-**L1** (87% yield, 81% ee), the use of (*P*)-**L2** gave product (*R*)-**17aa** in excellent yield and enantioselectivity (97% yield, 95% ee). We then moved on to further examine the substrate scope of this reaction. A coupling reaction between phosphonates **15a-d** and *o*-tolylboronic acid **16a** gave the corresponding axially chiral biaryls (**17aa–17da**) in excellent yields and moderate to high ee values (Fig. 6). These results demonstrate that the steric and electronic effects of substituents on the arylboronic acids **16b-f** affected the reactivities and enantioselectivities. More specifically, the introduction of an ethyl group at the C2-position of the phenyl ring increased the enantioselectivity (99% ee for **17af**). This confirmed that **L2** afforded improved enantioselectivity over **L1** (for full details, see Table S4, Supplementary Information).

**Figure 7:**
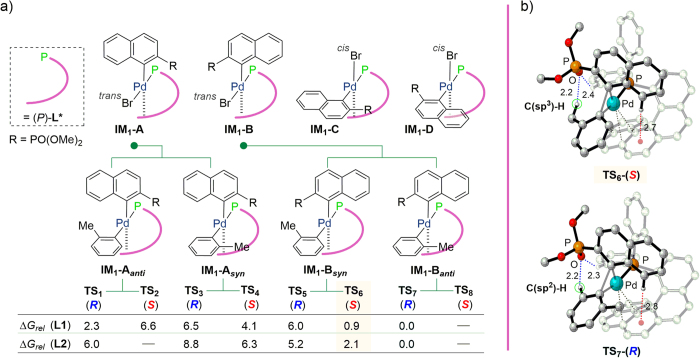
(**a**) Calculated relative Gibbs energy barriers (ΔG_*rel*_, kcal/mol) in toluene at 298 K for the transition state structures. (**b**) Calculated transition state structures of **TS**_**6**_ and **TS**_**7**_ using (*P*)-**L2**.

It was reported that the stereoselectivity of the SMC reaction is induced by reductive elimination from Pd(II)^46^,^47^. Thus, to gain an insight into the factors determining the enantioselectivity in our novel system, DFT calculations for the reductive elimination of Pd ((*P*)-**L1**) and Pd((*P*)-**L2**) complexes providing **17ba** were performed using the B97-D/6-31G* level of theory (LANL2DZ for the Pd atom). As shown in Fig. 7a, we hypothesized that four geometrical isomers (**IM**_**1**_**-A–D**) could be formed after oxidative addition. As the arene substrate and phosphorus center bearing strong *trans* influences prefer the *cis* arrangement, we surveyed the transition states (TSs) for the reductive elimination steps of **IM**_**2**_**-A**_***anti***_, **IM**_**2**_**-A**_***syn***_, **IM**_**2**_**-B**_***syn***_, and **IM**_**2**_**-B**_***anti***_, derived from **IM**_**1**_**-A** and **IM**_**1**_**-B**.

Using ligands **L1** and **L2**, we optimized seven (for **L1**) and six (for **L2**) of the eight possible transition state structures and their energies were calculated as shown in Fig. 7a. Among these, **TS**_**6**_ and **TS**_**7**_ were the most stable for (*S*)-**17ba** and (*R*)-**17ba**, respectively. The energy gap between **TS**_**6**_ and **TS**_**7**_ could arise from differences in the hydrogen bonding partners of the phosphonate sp^2^-oxygen and the hydrogen atom on the tolyl group (Fig. 7b; for all calculated transition state structures, see Figs S1 and S2, Supplementary Information). **TS**_**7**_ would therefore be energetically favorable because C(sp^2^)-H is a better hydrogen donor than C(sp^3^)-H^48^,^52^. In addition, we found a significant energy difference between **TS**_**6**_ and **TS**_**7**_ (ΔG_*rel*_ = 0.9 kcal mol^−1^ for **L1**, ΔG_*rel*_ = 2.1 kcal mol^−1^ for **L2**). Since in the transition states, the average helical pitch of **L2** (3.44 ± 0.06 Å) is smaller than that of **L1** (3.52 ± 0.10 Å), additional CH/π interactions can form between the arene moiety and the helical aromatic framework (Fig. 7b), reflecting the high enantioselectivity of coupling product **17ba**. The Boltzmann distributions of all optimized transition structures using (*P*)-**L1** and (*P*)-**L2** as ligands predicted (*R*)-product formation with selectivities of 62% ee and 91% ee, respectively. These values correlated well with the experimental results.

In summary, two novel optically active [5]helicenylphosphine ligands (**L1**, with a 7,8-dihydro[5]helicene core structure, and **L2**, with a fully aromatic [5]helicene core structure) were successfully synthesized, and the ligand structures were determined using X-ray crystallography. As expected, the X-ray crystallographic analysis of the Pd(dba)[**L1**] complex unambiguously showed that both the phosphorus atom and the double bond of the ligand are coordinated to the Pd center. These ligands were applied in Pd-catalyzed asymmetric allylic substitutions and Suzuki–Miyaura coupling (SMC) reactions. The newly developed ligands, in particular **L1**, were highly effective in asymmetric allylic substitutions. Moreover, we demonstrated that **L2** serves as a highly enantioselective ligand in the asymmetric SMC reaction to yield axially chiral biaryl compounds. Our concept may therefore open a novel route toward the application of helicene-based phosphine ligands in Pd-catalyzed asymmetric reactions.

## Methods

### General procedure for the Pd-catalyzed asymmetric allylic alkylation of indole

An oven-dried screw-capped vessel was charged with [PdCl(C_3_H_5_)]_2_ (0.5 mol%), **L1** (1.0 mol%), and CH_2_Cl_2_ (0.30 mL) under argon. The resulting mixture was stirred for 30 min at room temperature. Racemic 1,3-diphenyl-2-propenyl acetate **11** (0.15 mmol) in CH_2_Cl_2_ (0.45 mL), Cs_2_CO_3_ (0.3 mmol), and the corresponding indole (0.3 mmol) were added subsequently, and the reaction mixture was stirred at room temperature until all starting material had been consumed. The reaction mixture was then diluted with CH_2_Cl_2_, washed with water and brine, dried over Na_2_SO_4_, filtered, and concentrated under reduced pressure. The residue was purified by flash column chromatography on silica gel eluting with hexane–EtOAc (95:5) to afford **13**. The absolute configuration was determined by comparing the specific optical rotations with literature data (see Supplementary Information).

### General procedure for the Pd-catalyzed asymmetric allylic etherification

An oven-dried screw-capped vessel was charged with [PdCl(C_3_H_5_)]_2_ (0.5 mol%), **L1** (1.0 mol%), and CH_2_Cl_2_ (0.30 mL) under argon. The resulting mixture was stirred for 30 min at room temperature. Racemic 1,3-diphenyl-2-propenyl acetate **11** (0.15 mmol) in CH_2_Cl_2_ (0.45 mL), Cs_2_CO_3_ (0.45 mmol), and the corresponding alcohol (0.45 mmol) were added subsequently, and the reaction mixture was stirred at room temperature until all starting material had been consumed. The reaction mixture was then diluted with diethyl ether, washed with water and brine, dried over Na_2_SO_4_, filtered, and concentrated under reduced pressure. The residue was purified by flash column chromatography on silica gel eluting with hexane–EtOAc to afford **14**. The absolute configuration was determined by comparing the specific optical rotations with literature data (see Supplementary Information).

### General procedure for the Pd-catalyzed asymmetric SMC

An oven-dried amber screw-capped vessel was charged with Pd(OAc)_2_ (2.0 mol%), **L2** (2.4 mol%), aryl halide **15** (0.1 mmol), phenylboronic acid **16** (0.2 mmol), and K_3_PO_4_ (0.3 mmol). The vessel was then filled with argon gas. Subsequently, degassed toluene (0.5 mL) was added to the vessel and the reaction mixture was stirred at 50 °C until all starting material had been consumed. After cooling to room temperature, the reaction mixture was diluted with EtOAc, washed with water and brine, dried over Na_2_SO_4_, filtered, and concentrated under reduced pressure. The residue was purified by flash column chromatography on silica gel eluting with hexane–EtOAc to give a mixture of **17** and dehalogenated-**15**. The yields were determined by ^1^H NMR analysis. The absolute configurations of **17aa** and **17ba** were determined by comparing the specific optical rotations with literature data (see Supplementary Information). The absolute configurations of other products were not determined.

## Additional Information

**How to cite this article**: Yamamoto, K. *et al*. Rational Design and Synthesis of [5]Helicene-Derived Phosphine Ligands and Their Application in Pd-Catalyzed Asymmetric Reactions. *Sci. Rep.* **6**, 36211; doi: 10.1038/srep36211 (2016).

**Publisher’s note:** Springer Nature remains neutral with regard to jurisdictional claims in published maps and institutional affiliations.

## Supplementary Material

Supplementary Information 1

Supplementary Information 2

Supplementary Information 3

## Figures and Tables

**Figure 1:**
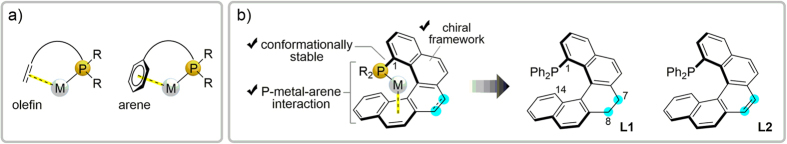
Design of [5]helicenylphosphine ligands based on metal-arene interactions.
