# Supplementary material for: Rational Design and Synthesis of [5]Helicene-Derived Phosphine Ligands and Their Application in Pd-Catalyzed Asymmetric Reactions
Source: Sci Rep. 2016 Nov 8;6:36211. doi: 10.1038/srep36211 (PMC5099951; doi:10.1038/srep36211)
Supplement: Supplementary Information 1 [file srep36211-s1.pdf]

Rational Design and Synthesis of [5]Helicene-Derived Phosphine Ligands and Their Application in Pd-Catalyzed Asymmetric Reactions

Kosuke Yamamoto, Takashi Shimizu, Kazunobu Igawa, Katsuhiko Tomooka, Go Hirai, Hiroshi Suemune* and Kazuteru Usui*

Contents

1.	General information	S2
2.	Preparation of compounds **L1** and **L2**	S2
3.	Preparation of spiro-TADDOLs	S6
4.	General procedure for asymmetric allylic alkylation of 1,3-diphenylallyl acetate **11** with dimethyl Malonate	S7
5.	Table S1. Optimizing the asymmetric allylation of indoles using (*M*)-**L1**	S8
6.	Asymmetric allylation of indoles (Figure 1b)	S8
7.	Table S2. Optimizing the asymmetric allylic etherification using (*M*)-**L1**	S10
8.	Asymmetric allylic etherifications (Figure 1c)	S10
9.	Table S3. Optimizing the asymmetric Suzuki–Miyaura coupling using (*P*)-**L1** and **L2**	S12
10.	Asymmetric Suzuki–Miyaura coupling (Figure 6)	S12
11.	Table S4. Scope of substrate for asymmetric Suzuki–Miyaura coupling using **L1** and **L2**	S15
12.	Figure S1 and S2, DFT calculation methods and data	S16
13.	Table S5. Cartesian coordinates	S19
14.	Chiral HPLC chromatograms	S34
15.	Figure S3 and S4, OPTEP drawings of compounds **L1** and **L2** with 50% ellipsoid probability	S43
16.	Figure S5, Isolation and X-ray structure of Pd(dba)[**L1**] **10**	S44
17.	Table S6. Crystallographic data collection and structural refinement information	S45

1. General information

All reagents and solvents were purchased from commercial sources and were used as received unless otherwise stated. Reagent grade solvents (acetonitrile, DMF, methanol, and toluene) were distilled prior to use, with the exception of tetrahydrofuran (dehydrated, Kanto Chemical Co., Inc.). Reactions were monitored by thin layer chromatography (TLC) (silica gel 60 F_{254}, 0.25 mm) analysis. Flash column chromatography was performed on flash silica gel 60N (spherical neutral, particle size 40−50 μm). HPLC analyses were conducted with a UV detector and a chiral column (4.6 mm × 250 mm). ^{1}H, ^{13}C, and ^{31}P NMR spectroscopy carried out in $CDCl_3$ at ambient temperature on a 400 or 500 MHz NMR spectrometer (400 or 500 MHz for ^{1}H NMR, 100 or 125 MHz for ^{13}C NMR, and 202 MHz for ^{31}P NMR). Chemical shifts were reported in parts per million (ppm) relative to tetramethylsilane (δ 0.00 ppm) or $CDCl_3$ (δ 7.26 ppm) for ^{1}H NMR and $CDCl_3$ (δ 77.0 ppm) for ^{13}C NMR. Coupling constants were reported as *J* values in Hertz (Hz). Splitting patterns are designated as s (singlet), d (doublet), t (triplet), dd (double doublet), ddd (doublet of doublet of doublets), br (broad), and m (multiplet). Infrared (IR) spectra of neat samples were recorded in ATR (attenuated total reflectance) mode using an FT-IR instrument. High-resolution mass spectrometry (HRMS) was carried out on an ESI-TOF spectrometer. Optical rotations were measured with a polarimeter using a 0.5 dm cell. CD measurements were performed on a spectropolarimeter with an optical path length of 1.0 mm.

2. Preparation of compounds L1 and L2

4-(2-Bromophenyl)-1,2-dihydrophenanthrene-3-carbaldehyde (5)

A suspension of 4-chloro-3-formyl-1,2-dihydrophenanthrene **3** (121 mg, 0.50 mmol), 2-bromophenylboronic acid **4** (200 mg, 1.00 mmol), tetrebytylammonium bromide (322 mg, 1.00 mmol), $Pd(OAc)_2$ (5.6 mg, 5 mol %) and K_2CO_3 (276 mg, 2.00 mmol) in 1.25 mL of degassed toluene/water mixture (4:1) was stirred at 60 °C under an argon atmosphere for 1 h. The organic layers were combined, washed with water and brine, dried over $MgSO_4$, filtered, and concentrated under reduced pressure. The residue was purified by flash column chromatography on silica gel eluting with hexane−EtOAc (95:5) to afford **5** (162 mg, 89%) as a pale yellow solid. mp = 125−128 °C; IR (neat, cm^{-1}) 2936, 1655, 1360, 1254, 1206, 1055, 1033, 826, 739, 667, 655; ^{1}H NMR (500 MHz, $CDCl_3$): δ 9.68 (s, 1H), 7.84 (d, *J* = 8.2 Hz, 1H), 7.77 (d, *J* = 8.1 Hz, 1H), 7.64 (dd, *J* = 8.0, 1.1 Hz, 1H), 7.44−7.23 (m, 6H), 7.08−7.04 (m, 1H), 2.99−2.96 (m, 2H), 2.78−2.62 (m, 2H); ^{13}C NMR (125 MHz, $CDCl_3$) : δ 192.9, 152.2, 140.5, 139.3, 136.4, 133.8, 133.5, 132.5, 131.5, 131.0, 130.5, 130.0, 128.9, 127.4, 126.3, 126.2, 124.8, 124.7, 124.3, 30.0, 19.9; HRMS (ESI, Pos) *m/z*: $[M + Na]^+$ calcd for $C_{21}H_{15}BrONa$ 385.0198, found 385.0195.

10-Bromo-3,4-dihydrodibenzo[*c*,*g*]phenanthrene (6)

To a stirred suspension of compound **5** (1.1 g, 3.03 mmol) and K_2CO_3 (781 mg, 5.65 mmol) in 30 mL of MeOH−THF (2:3) was added a solution of Ohira−Bestmann reagent (diethyl (1-diazo-2-oxopropyl)phosphonate) (1.0 g, 4.54 mmol) in MeOH (5.4 mL) under an argon atmosphere at 0 °C. The reaction mixture was allowed to warm to room temperature and stirred overnight at the same temperature. After removing the MeOH under reduced pressure, the residue was diluted with EtOAc and washed with water and brine, dried over $MgSO_4$, filtered, and concentrated under reduced pressure. The residue was dried in vacuo for 1 h, and then subjected to the next reaction without further purification. The residue was

dissolved in toluene (15 mL) and $PtCl_2$ (40 mg, 0.15 mmol) was added to this solution under an argon atmosphere at room temperature. The mixture was heated at 120 °C for 1 h. After cooling to room temperature, the solvent was evaporated and the residue was purified by flash column chromatography on silica gel with hexane−CH_2Cl_2 (98:2) to afford **6** (574 mg, 53%) as a pale yellow solid. mp: 169−173 °C; IR (neat, cm^{-1}) 2369, 1177, 1039, 835, 818, 742, 724, 655, 691; ^1H NMR (500 MHz, $CDCl_3$): δ 7.90 (dd, J = 8.1, 0.9 Hz, 1H), 7.86−7.79 (m, 3H), 7.61 (dd, J = 7.3, 1.2 Hz, 1H), 7.55−7.52 (m, 2H), 7.38−7.35 (m, 1H), 7.29 (t, J = 7.7 Hz, 1H), 7.14−7.07 (m, 2H), 3.04−2.92 (m, 3H), 2.83−2.76 (m, 1H); ^{13}C NMR (125 MHz, $CDCl_3$): δ 142.4, 136.0, 134.8, 132.8, 132.5, 132.2, 132.0, 131.1, 131.0, 128.3, 128.2, 127.7, 127.5, 126.4, 125.8, 125.5, 125.2, 124.5, 123.8, 120.3, 31.0, 30.6; HRMS (ESI, Pos) *m/z*: $[M + Na]^+$ calcd for $C_{22}H_{15}BrNa$ 381.0249, found 381.0235.

(3,4-Dihydrodibenzo[*c*,*g*]phenanthren-10-yl)diphenylphosphine oxide (7)

To a solution of **6** (359 mg, 1.00 mmol) in THF (12 mL) was added a solution of *sec*-BuLi (1.9 mL, 1.5 mmol, 0.78 M in cyclohexane/hexane) under an argon atmosphere at –78 °C over 5 min. The solution color was turned to dark green from orange by addition of *sec*-BuLi. The reaction mixture was stirred for 20 min, and then added a solution of freshly distilled chlorodiphenylphosphine (662 mg, 0.5 mL, 3.00 mmol) in THF (3.0 mL) at the same temperature. The reaction mixture was stirred for warmed to 0 °C over 6 h. The reaction was quenched with 30% H_2O_2 (170 mg, 0.56 mL, 5 mmol) at 0 °C and stirred at room temperature for 2 h. The reaction mixture was diluted with H_2O and extracted with EtOAc. The organic layers were combined; washed with saturated aqueous $Na_2S_2O_3$ and brine; dried over $MgSO_4$; filtered; and concentrated under reduced pressure. CH_2Cl_2 was then added to the crude mixture, and less soluble product was removed by filtration. After removal of the solvent under reduced pressure, the crude product was purified by flash column chromatography on silica gel with hexane−EtOAc (50:50) to afford **7** (304 mg, 63%) as a yellow foam. mp: 230−234 °C; IR (neat, cm^{-1}) 2937, 2746, 1437, 1219, 913, 772, 672, 657; ^1HNMR (500 MHz, $CDCl_3$) : δ 8.06 (m, 1H), 7.85 (dd, J = 8.0, 1.9 Hz, 1H), 7.72 (d, J = 7.2, 1H), 7.69 (d, J = 7.34 MHz, 1H), 7.53−7.48 (m, 2H), 7.41−7.37 (m, 1H), 7.27−7.21 (m, 5H), 7.14 (d, J = 8.1 Hz, 1H), 7.10−7.05 (m, 4H), 7.02 (ddd, J = 8.5, 6.7, 1.4 Hz, 1H), 6.74−6.69 (m, 3H), 2.78 (ddd, J = 15.8, 4.6, 1.9 Hz, 1H), 2.56 (ddd, 15.6, 15.6, 4.3 Hz, 1H), 2.48 (ddd, J = 14.1, 4.4, 1.8 Hz, 1H), 2.23 (ddd, J = 15.2, 15.2, 5.0 Hz, 1H); ^{13}C NMR (125 MHz, $CDCl_3$) : δ 141.7, 138.1, 138.0, 137.9, 136.2, 135.9, 135.4, 135.1, 133.6, 132.92, 132.86, 132.8, 132.6, 131.0, 130.96, 130.8, 130.28, 130.26, 130.2, 130.1, 130.0, 129.2, 128.6, 128.4, 128.2, 128.0, 127.9, 127.8, 127.6, 127.5, 126.5, 125.4, 125.3, 125.2, 124.4, 123.2, 123.1, 30.5, 30.1 (observed complexity due to C-P couplings); ^{31}P NMR (202 MHz, $CDCl_3$): δ 25.2; HRMS (ESI, Pos) *m/z*: $[M + Na]^+$ calcd for $C_{34}H_{25}NaOP$ 503.1535, found 503.1547; HPLC analysis (CHIRALPAK IA column, flow rate 1.0 mL/min, hexane−*i*-PrOH−EtOH (90:5:5), λ = 254 nm) t_P 10.1 min, t_M 11.5 min.

Resolution of 7 with (*S*,*S*)-spiro-TADDOL [(+)-8]

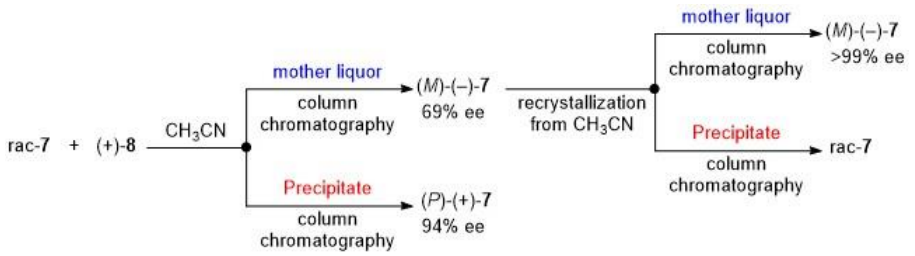

A mixture of rac-**7** (100 mg, 0.21 mmol) and (+)-**8** (63 mg, 0.13 mmol) in CH_3CN (6.9 mL, 0.03 M) was heated at 80 °C for several minutes. The resulting solution was allowed to cool to room temperature gradually, whereupon a pale yellow precipitate appeared. The resulting precipitate was collected by filtration and washed with CH_3CN and then dissolved in hexane−EtOAc (1:1). The solution was purified by flash column chromatography on silica gel with hexane−EtOAc (1:1) to afford (*P*)-(+)-**7** as a pale yellow solid (39 mg, 39%, 94% ee). The mother liquor was concentrated under reduced pressure and purified by flash column chromatography on silica gel with hexane−EtOAc (1:1) to afford (*M*)-(−)-**7** (61 mg, 61%, 69% ee). Phosphine oxide (*M*)-(−)-**7** was further purified by recrystallization from hot CH_3CN (1.3 mL, 0.1 M). The precipitate was collected by filtration and purified by flash column chromatography on silica gel with hexane−EtOAc (1:1) to afford rac-**7** as a pale yellow solid (16 mg). The mother liquor was concentrated under reduced pressure and purified over silica gel to give (*M*)-(−)-**7** (45 mg, >99% ee).

Resolution of 3 with (*R*,*R*)-spiro-TADDOL [(−)-8]

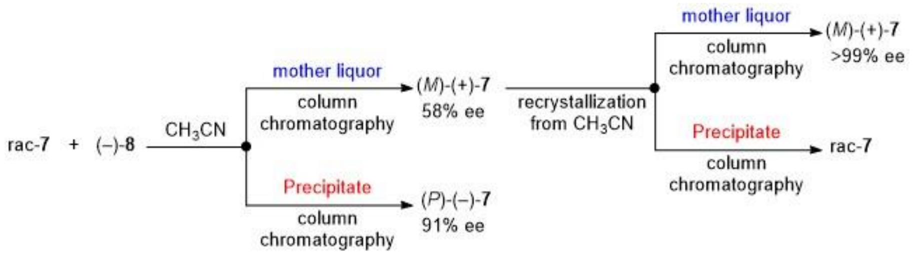

A mixture of rac-**7** (100 mg, 0.21 mmol) and (−)-**8** (65 mg, 0.13 mmol) in CH_3CN (7.0 mL, 0.03 M) was heated at 80 °C for several minutes. The resulting solution was allowed to cool to room temperature gradually, whereupon a pale yellow precipitate appeared. The resulting precipitate was collected by filtration and washed with CH_3CN and then dissolved in hexane−EtOAc (1:1). The solution was purified by flash column chromatography on silica gel with hexane−EtOAc (1:1) to afford (*M*)-(−)-**7** as a pale yellow solid (44 mg, 44%, 91% ee). The mother liquor was concentrated under reduced pressure and purified by flash column chromatography on silica gel with hexane−EtOAc (1:1) to afford (*P*)-(+)-**7** (55 mg, 54%, 58% ee). Phosphine oxide (*P*)-(+)-**7** was further purified by recrystallization from hot CH_3CN (1.4 mL, 0.1 M). The precipitate was collected by filtration and purified by flash column chromatography on silica gel with hexane−EtOAc (1:1) to afford rac-**7** as a pale yellow solid (16 mg). The mother liquor was concentrated under reduced pressure and purified over silica gel to give (*P*)-(+)-**7** (36 mg, >99% ee).

(3,4-Dihydrodibenzo[*c*,*g*]phenanthren-10-yl)diphenylphosphine ((*P*)-L1)

Triphenyl phosphite (189 mg, 1.14 mmol) and $HSiCl_3$ (464 mg, 3.43 mmol) was added to a solution of (*P*)-**7** (55 mg, 99% ee, 0.11 mmol) in 1.1 mL of toluene−THF (1:1) under an argon atmosphere. The mixture was heated at 100 °C for 24 h. After cooled to 0 °C, the mixture was diluted with Et_2O and added 1M NaOH solution. The resulting mixture was filtered through celite, and washed with water and brine, dried over $MgSO_4$, filtered, and concentrated under reduced pressure. The residue was purified by flash column chromatography on silica gel eluting with degassed hexane−EtOAc (99:1) to afford (*P*)-**L1** (37 mg, 72%, 99% ee) as a pale yellow solid. mp = 190−197 °C; IR (neat, cm^{-1}) 1434, 1088, 1039, 1027, 835, 810, 747, 666, 654; ^1H NMR (500 MHz, $CDCl_3$): δ 7.87 (d, J = 8.1 Hz, 2H), 7.84 (d, J = 8.1 Hz, 1H), 7.78 (d, J = 8.0 Hz, 1H), 7.42−7.36 (m, 3H), 7.31 (t, J = 7.4 Hz, 1H), 7.18−7.04 (m, 6H), 6.94−6.90 (m, 3H), 6.66 (t, J =

7.2 Hz, 2H), 6.27 (t, J = 7.6 Hz, 2H), 2.72−2.59 (m, 3H), 2.05−1.98 (m, 1H); ^{13}C NMR (125 MHz, $CDCl_3$): δ 141.0, 138.2, 138.0, 137.9, 137.8, 137.7, 136.9, 136.7, 134.1, 133.9, 133.8, 133.7, 133.5, 133.12, 133.09, 133.0, 132.91, 132.86, 132.72, 132.70, 132.40, 132.36, 131.59, 131.57, 129.1, 128.5, 128.04, 127.99, 127.9, 127.8, 127.5, 127.4, 125.7, 125.6, 125.5, 124.51, 124.48, 124.20, 124.16, 30.6, 30.3 (observed complexity due to C-P couplings); ^{31}P NMR (202 MHz, $CDCl_3$): δ −2.0; HRMS (ESI, Pos) m/z: $[M + Na]^+$ calcd for $C_{34}H_{25}Na$ 487.1586, found 487.1582; UV-Vis (CH_3CN) λmax (ε) 194 (82,764), 220 (82,867), 327 (11,537), 349 (11,314); $[\alpha]_D^{25}$ = +1750 (c = 3.2×10^{-3}, (P) : 99% ee, $CHCl_3$), $[\alpha]_D^{25}$ = −1667 (c = 3.0×10^{-3}, (M) : 99% ee, $CHCl_3$); HPLC analysis (CHIRALPAK IA column, flow rate 0.8 mL/min, hexane− i-PrOH (99:1), λ = 254 nm) t_P 4.2 min, t_M 5.3 min.

Dibenzo[*c*,*g*]phenanthren-10-yldiphenylphosphine oxide (9)

A solution of (P)-**7** (48 mg, 0.10 mmol) and DDQ (45 mg, 0.20 mmol) in toluene (0.5 mL) was stirred at 90 °C under an argon atmosphere for 9 h. The reaction flask was covered with foil to shield from light. After cooled to room temperature, formed precipitate was removed by filtration using celite and filter cake was washed with toluene thoroughly. The filtrate was washed with 1M NaOH solution, water, brine, and dried over $MgSO_4$, filtered, and concentrated under reduced pressure. The residue was purified by column chromatography on silica gel eluting with CH_2Cl_2−acetone (95:5) to afford (P)-**9** (48 mg, quant) as a pale yellow solid. mp = 252−260 °C; IR (neat, cm^{-1}) 3065, 2345, 2207, 1438, 1184, 1109, 842, 795, 723, 700; ^{1}H NMR (500 MHz, $CDCl_3$): δ 8.20 (dd, J = 7.6, 1.6 Hz, 1H), 7.97−7.93 (m, 2H), 7.86 (dd, J = 11.6, 8.3 Hz, 2H), 7.69−7.58 (m, 4H), 7.55–7.52 (m, 1H), 7.48 (d, J = 8.1, 1H), 7.37 (d, J = 8.5, 1H), 7.17−7.16 (m, 2H), 7.09−7.05 (m, 4H), 7.00−6.96 (m, 1H), 6.73−6.70 (m, 2H), 6.27−6.22 (m, 2H); ^{13}C NMR (125 MHz, $CDCl_3$): δ 136.7, 136.6, 133.3, 132.4, 131.6, 131.1, 130.7, 130.22, 130.16, 129,7, 129.4, 129.1, 128.74, 128.67, 128.6, 128.0, 127.6, 127.5, 127.4, 126.9, 126.8, 126.6, 125.6, 125.5, 125.2, 125.1, 124.4, 127.8, 127.6, 127.5, 126.5, 125.4, 125.3, 125.2, 124.4, 123.2, 123.1 (observed complexity due to C-P couplings); ^{31}P NMR (202 MHz, $CDCl_3$): δ 23.9; HRMS (ESI, Pos) m/z: $[M + Na]^+$ calcd for $C_{34}H_{23}NaOP$ 501.1379, found 501.1381

Dibenzo[*c*,*g*]phenanthren-10-yldiphenylphosphane ((*P*)-L2)

Triethyl phosphite (52 mg, 0.31 mmol) and $HSiCl_3$ (423 mg, 3.12 mmol) was added to a solution of (P)-**9** (30 mg, 99% ee, 0.06 mmol) in 0.8 mL of toluene−THF (1:1) under an argon atmosphere. The reaction flask was covered with foil to shield from light and stirred 100 °C for 25 h. After cooled to 0 °C, the mixture was diluted with Et_2O and added 1M NaOH solution. The resulting mixture was filtered through celite, and washed with water and brine, dried over $MgSO_4$, filtered, and concentrated under reduced pressure. The residue was purified by flash column chromatography on silica gel eluting with degassed hexane−EtAc (98:2) to afford (P)-**L2** (20 mg, 70%, 99% ee) as a pale yellow solid. mp = 202−208 °C; IR (neat, cm^{-1}) 1479, 1434, 1262, 1215, 1092, 1027, 908, 838, 793, 763, 744, 696; ^{1}H NMR (500 MHz, $CDCl_3$): δ 8.55−8.50 (m, 4H), 8.43 (dd, J = 8.3, 0.7 Hz, 1H), 8.35−8.25 (m, 4H), 8.07−8.01 (m, 2H), 7.79−7.76 (m, 1H), 7.74−7.70 (m, 3H), 7.64−7.61 (m, 1H), 7.44 (t, J = 7.7 Hz, 1H), 7.32−7.25 (m, 4H), 6.51−6.48 (m, 2H) ; ^{13}C NMR (125 MHz, $CDCl_3$): δ 138.4, 138.2, 137.9, 137.7, 137.6, 137.5, 134.5, 134.3, 134.0, 133.9, 132.7, 132.44, 132.41, 132.37, 132.34, 132.26, 132.25, 132.2, 132.0, 130.7, 128.6, 128.5, 128.3, 128.21, 128.20, 128.0, 127.7, 127.6, 127.5, 127.3, 127.2, 127.1, 126.8, 126.79, 126.0, 125.96, 125.9, 125.8, 125.7, 125.5, 125.3, 125.2 (observed complexity due to C-P couplings); ^{31}P NMR (202 MHz, $CDCl_3$): δ 1.6 ; HRMS (ESI, Pos) m/z: $[M + Na]^+$ calcd for $C_{34}H_{23}PNa$ 485.1430, found

485.1434; $[\alpha]_D^{25}$ = +920 (c = 5.0×10^{-3}, (P) : 99% ee, $CHCl_3$), $[\alpha]_D^{25}$ = −880 (c = 5.0×10^{-3}, (M) : 99% ee, $CHCl_3$); HPLC analysis (CHIRALCEL OD-H column, flow rate 0.5 mL/min, hexane− *i*-PrOH (99:1), λ = 254 nm) t_P 16.7 min, t_M 13.1 min.

3. Preparation of spiro-TADDOLs

(2*S*,3*S*)-Dimethyl 1,4-dioxaspiro[4.5]decane-2,3-dicarboxylate (S1)

A solution of (−)-dimethyl D-tartrate (5.46 g, 30.7 mmol), cyclohexanone (3.31 g, 33.7 mmol) and *p*-toluenesulfonic acid (570 mg, 3.0 mmol) in toluene was refluxed with azeotropic removal of H_2O for 5 h. After cooled to room temperature, the mixture was diluted with EtOAc and washed with saturated $NaHCO_3$. The organic layers were dried over anhydrous $MgSO_4$, filtered, and concentrated under reduced pressure to give the crude product as a brown oil. The residue was purified by flash column chromatography on silica gel with hexane−EtOAc (9:1) to afford **S1** (811 mg, 10%) as a white solid. ^{1}H NMR (400 MHz, $CDCl_3$): δ 4.81 (s, 2H), 3.82 (s, 6H), 1.72−1.61 (m, 8H), 1.43−1.41 (m, 2H). The ^{1}H NMR spectroscopic data are identical to those reported in the literature.[1]

(2S,3S)-1,4-Dioxaspiro[4.5]decane-2,3-diylbis(diphenylmethanol) [(+)-8]

The solution of phenylmagnesium bromide prepared from bromobenzene (2.43 g, 15.5 mmol) and magnesium (413 mg, 17.1 mmol) in THF (15.5 mL) was added to a solution of **S1** (811 mg, 3.1 mmol) in THF (0.75 mL) under an argon atmosphere at 0 °C. The reaction mixture was stirred at 70 °C for 1 h. After cooled to room temperature, the reaction mixture was treated with saturated aqueous NH_4Cl, 10% HCl, and extracted with EtOAc. The organic layers were washed with saturated $NaHCO_3$ and brine; dried over anhydrous $MgSO_4$; filtered; and concentrated under reduced pressure to give the crude product as a yellow oil. The crude product was crystallized from hexane−EtOAc (85:15) to afford (+)**-8** (1.14 g, 72%) as a white solid. ^{1}HNMR (500 MHz, $CDCl_3$): δ 7.53−7.51 (m, 4H), 7.39−7.37 (m, 4H), 7.32−7.24 (m, 12H), 4.57 (s, 2H), 3.89 (m, 4H), 1.44−1.41 (m, 4H), 1.26−1.11 (m, 6H). The ^{1}H NMR spectral data were matched with the literature data.[1]

(2*R*,3*R*)-Dimethyl 1,4-dioxaspiro[4.5]decane-2,3-dicarboxylate (S2)

A solution of (+)-dimethyl L-tartrate (3.56 g, 20.0 mmol), cyclohexanone (2.16 g, 22.0 mmol) and *p*-toluenesulfonic acid (380 mg, 2.2 mmol) in toluene was refluxed with azeotropic removal of H_2O for 2 h. After cooled to room temperature, the mixture was diluted with EtOAc and washed with saturated $NaHCO_3$. The organic layers were dried over anhydrous $MgSO_4$, filtered, and concentrated under reduced pressure to give the crude product as a brown oil. The residue was purified by flash column chromatography on silica gel with hexane−EtOAc (9:1) to afford **S1** (3.28 g, 64%) as a colorless oil. ^{1}H NMR (400 MHz, $CDCl_3$): δ 4.81 (s, 2H), 3.82 (s, 6H), 1.74−1.59 (m, 8H), 1.44−1.41 (m, 2H). The ^{1}H NMR spectral data were matched with the literature data.[1]

[1] S. Müller, M. C. Afraz, R. d. Gelder, G, J. A. Ariaans, B. Kaptein, Q. B. Broxterman, A. Bruggink, *Eur. J. Org. Chem.* **2005**, 1082.

(2*R*,3*R*)-1,4-Dioxaspiro[4.5]decane-2,3-diylbis(diphenylmethanol) [(−)-8]

The solution of phenylmagnesium bromide prepared from bromobenzene (3.94 g, 1.6 mL, 25.1 mmol) and magnesium (688 mg, 28.3 mmol) in THF (15.7 mL) was added to a solution of **S2** (1.29 g, 5.0 mmol) in THF (1.3 mL) under an argon atmosphere at 0 °C. The reaction mixture was stirred at 60 °C for 2 h. After cooled to room temperature, the reaction mixture was treated with saturated aqueous NH_4Cl, 10% HCl, and extracted with EtOAc. The organic layers were washed with saturated $NaHCO_3$ and brine; dried over anhydrous $MgSO_4$; filtered; and concentrated under reduced pressure to give the crude product as a yellow oil. The crude product was crystallized from CH_2Cl_2−hexane to afford (−)-**8** (2.02 g, 80%) as a white solid. ^{1}HNMR (400 MHz, $CDCl_3$): δ 7.52−7.51 (m, 4H), 7.40−7.37 (m, 4H), 7.35−7.24 (m, 12H), 4.57 (s, 2H), 3.89 (m, 4H), 1.46−1.39 (m, 4H), 1.27−1.10 (m, 6H). The ^{1}H NMR spectral data were matched with the literature data.[1]

4. General procedure for asymmetric allylic alkylation of 1,3-diphenylallyl acetate 11 with dimethyl malonate

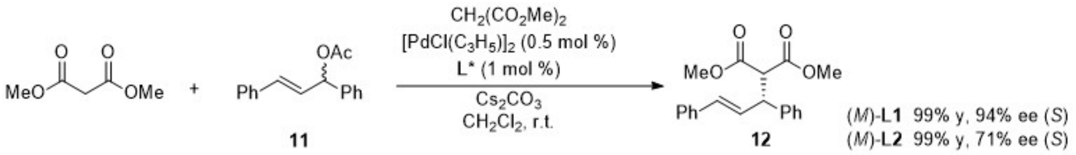

Oven-dried screw capped vessel was charged with $[PdCl(C_3H_5)]_2$ (0.5 mol %), ligand **L*** (1.0 mol %) was added CH_2Cl_2 (0.30 mL) under argon. The resulting mixture was stirred for 30 min at room temperature. Racemic 1,3-diphenylallyl acetate **11** (0.15 mmol) in CH_2Cl_2 (0.45 mL), Cs_2CO_3 (0.45 mmol), and dimethyl malonate (0.45 mmol) were added subsequently, and the reaction mixture was stirred at room temperature until starting material was consumed (**L1**: 1 h, **L2**: 2 h). The reaction mixture was diluted with ether, washed with water and brine, dried over Na_2SO_4, filtered, and concentrated under reduced pressure. The residue was purified by flash column chromatography on silica gel eluting with hexane−EtOAc (9:1) to afford the product **12** as a clear oil. ^{1}H NMR (500 MHz, $CDCl_3$): δ 7.33-7.20 (m, 10H), 6.48 (d, J = 15.8, 1H), 6.33 (dd, J = 15.7, 8.6 Hz, 1H), 4.26 (dd, J = 10.7, 8.7 Hz, 1H), 3.95 (d, J = 10.8 Hz, 1H), 3.70 (s, 3H), 3.52 (s, 3H); $[\alpha]_D^{25}$ = +16.2 (c = 0.8, 94% ee, $CHCl_3$); HPLC analysis (CHIRALCEL OD-H column, flow rate 0.5 mL/min, hexane−i-PrOH (95:5), λ = 254 nm). The ^{1}H NMR spectral data were matched with the literature data.[2]

[2] Z. Liu, H. Du, *Org. Lett.* **2010**, *12*, 3054.

5. Table S1. Optimizing the asymmetric allylation of indoles using (*M*)-L1[a]

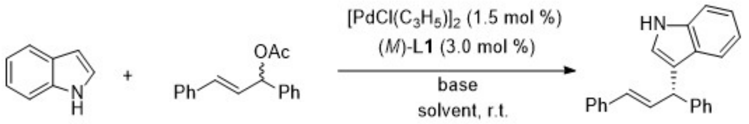

11 13a

entry	solvent	base	time (h)	yield[b] (%)	ee[c] (%)
1	toluene	Na_2CO_3	48	11	68
2	THF	Na_2CO_3	48	15	90
3	CH_3CN	Na_2CO_3	48	11	68
4	CH_2Cl_2	Na_2CO_3	48	73	98
5	CH_2Cl_2	K_2CO_3	12	67	98
6	CH_2Cl_2	Cs_2CO_3	1	65	96
7[d]	CH_2Cl_2	Cs_2CO_3	1	78	98
8[e]	CH_2Cl_2	Cs_2CO_3	1	97	99

[a]Conditions: **11** (0.18 mmol), indole (0.15 mmol), Pd/**L1** = 1/1 (3.0 mol % Pd), and base (0.3 mmol) in solvent (0.75 mL). [b]Isolated yields. [c]Determined by HPLC with a CHIRALPAK OD-H column. [d] Conditions: **11** (0.15 mmol), indole (0.18 mmol), Pd/**L1** = 1/1 (1.0 mol % Pd), and Cs_2CO_3 (0.3 mmol) in CH_2Cl_2 (0.75 mL). [e]2.0 equiv of indole (0.3 mmol) was used under similar conditions (entry 7).

6. Asymmetric allylation of indoles (Figure 1b)

(*S*,*E*)-3-(1,3-Diphenylallyl)-1*H*-indole (13a)

The reaction was conducted according to general procedure using **11** (33 mg) and indole (35 mg). Flash column chromatography with hexane−EtOAc (95:5) afforded **13a** (45 mg, 97%) as a white solid. ^{1}H NMR (500 MHz, $CDCl_3$): δ 8.00 (brs, 1H), 7.43 (d, *J* = 8.0 Hz, 1H), 7.37−7.16 (m, 12H), 7.02 (t, *J* = 7.5, 1H), 6.92 (m, 1H), 6.73 (dd, *J* = 15.8, 7.4 Hz, 1H), 6.44 (d, *J* = 15.8 Hz, 1H), 5.12 (d, *J* = 7.4 Hz, 1H) ; HRMS (ESI, Pos) *m/z*: $[M + Na]^+$ calcd for $C_{23}H_{19}NNa$ 332.1410, found 332.1421; $[\alpha]_D^{25}$ = +40 (*c* = 0.5, 99% ee, CH_2Cl_2); HPLC analysis (CHIRALCEL OD-H column, flow rate 0.5 mL/min, hexane−*i*-PrOH (90:10), λ = 254 nm). The ^{1}H NMR spectral data were matched with the literature data.[3]

(*S*,*E*)-3-(1,3-Diphenylallyl)-2-methyl-1*H*-indole (13b)

The reaction was conducted according to general procedure using **11** (33 mg) and 2-methylindole (39 mg). Flash column chromatography with hexane−EtOAc (95:5) afforded **13b** (49 mg, 99%) as an orange oil. ^{1}H NMR (500 MHz, $CDCl_3$): δ 7.80 (bs, 1H), 7.37−7.33 (m, 5H), 7.29−7.25 (m, 5H), 7.20−7.17 (m, 2H), 7.08 (t, *J* = 7.1 Hz, 1H), 6.96 (t, *J* = 8.0 Hz, 1H), 6.82 (dd, *J* = 15.8, 7.2 Hz, 1H), 6.41 (d, *J* = 15.9 Hz, 1H), 5.15 (d, *J* = 7.1 Hz, 1H), 2.38 (s, 3H); $[\alpha]_D^{25}$ = −3.6 (*c* = 0.5, 97% ee, CH_2Cl_2); HPLC analysis (CHIRALCEL OD-H column, flow rate 0.5 mL/min,

3 H. Y. Cheung, W. –Y. Yu, F. L. Lam, T. T. –L. Au-Yeung, Z. Zhou, T. H. Chan, S. S. C. Chan, *Org. Lett.* **2007**, *9*, 4295.

hexane−*i*-PrOH (90:10), λ = 254 nm). The ^{1}H NMR spectral data were matched with the literature data.[3]

(*S*,*E*)-3-(1,3-Diphenylallyl)-2-phenyl-1*H*-indole (13c)

The reaction was conducted according to general procedure using **11** (33 mg) and 2-Phenylindole (58 mg). Flash column chromatography with hexane−EtOAc (95:5) afforded **13c** (58 mg, 99%) as an orange oil. ^{1}H NMR (500 MHz, $CDCl_3$): *δ* 8.10 (bs, 1H), 7.55−7.53 (m, 2H), 7.47−7.33 (m, 9H), 7.28-7.25 (m, 4H), 7.20−7.16 (m, 3H), 7.00 (t, *J* = 7.1 Hz, 1H), 6.89 (dd, *J* = 15.8, 7.3 Hz, 1H), 6.41 (d, *J* = 15.8 Hz, 1H), 5.28 (d, *J* = 7.3 Hz, 1H); $[\alpha]_D^{25}$ = +66.5 (*c* = 0.4, 96% ee, CH_2Cl_2); HPLC analysis (CHIRALCEL OD-H column, flow rate 0.8 mL/min, hexane−*i*-PrOH (90:10), λ = 254 nm). The ^{1}H NMR spectral data were matched with the literature data.[3]

(*S*,*E*)-3-(1,3-Diphenylallyl)-5-methyl-1*H*-indole (13d)

The reaction was conducted according to general procedure using **11** (33 mg) and 5-methylindole (39 mg). Flash column chromatography with hexane−EtOAc (95:5) afforded **13d** (46 mg, 95%) as a pale pink solid. ^{1}H NMR (500 MHz, $CDCl_3$): *δ* 7.89 (bs, 1H), 7.37−7.18 (m, 12H), 7.00 (dd, *J* = 8.3, 1.4 Hz, 1H), 6.87 (m, 1H), 6.71 (dd, *J* = 15.8, 7.3 Hz, 1H), 6.42 (d, *J* = 15.9 Hz, 1H), 5.09 (d, *J* = 7.3 Hz, 1H), 2.37 (s, 3H); $[\alpha]_D^{25}$ = +50.7 (*c* = 0.7, 96% ee, CH_2Cl_2); HPLC analysis (CHIRALCEL OD-H column, flow rate 0.8 mL/min, hexane−*i*-PrOH (90:10), λ = 254 nm). The ^{1}H NMR spectral data were matched with the literature data.[3]

(*S*,*E*)-3-(1,3-Diphenylallyl)-5-methoxy-1*H*-indole (13e)

The reaction was conducted according to general procedure using **11** (33 mg) and 5-methoxyindole (44 mg). Flash column chromatography with hexane−EtOAc (95/5) afforded **13e** (51 mg, 99%) as a brown oil. ^{1}H NMR (500 MHz, $CDCl_3$): *δ* 7.89 (bs, 1H), 7.38−7.18 (m, 11H), 6.89-6.82 (m, 3H), 6.71 (dd, *J* = 15.8, 7.3 Hz, 1H), 6.44 (d, *J* = 15.7 Hz, 1H), 5.07 (d, *J* = 7.3 Hz, 1H), 3.71 (s, 3H); $[\alpha]_D^{25}$ = +36 (*c* = 0.4, 96% ee, CH_2Cl_2); HPLC analysis (CHIRALCEL OD-H column, flow rate 0.5 mL/min, hexane−*i*-PrOH (90:10), λ = 254 nm). The ^{1}H NMR spectral data were matched with the literature data.[3]

(*S*,*E*)-5-Bromo-3-(1,3-diphenylallyl)-1*H*-indole (13f)

The reaction was conducted according to general procedure using **11** (33 mg) and 5-bromolindole (59 mg). Flash column chromatography with hexane−EtOAc (95/5) afforded **13f** (59 mg, 99%) as a yellow oil. ^{1}H NMR (500 MHz, $CDCl_3$): *δ* 8.05 (bs, 1H), 7.54 (s, 1H), 7.37−7.19 (m, 12H), 6.93 (d, *J* = 1.5 Hz, 1H), 6.68 (dd, *J* = 15.8, 7.3 Hz, 1H), 6.41 (d, *J* = 15.9 Hz, 1H), 5.06 (d, *J* = 7.3 Hz, 1H); $[\alpha]_D^{25}$ = +33.6 (*c* = 0.3, 99% ee, CH_2Cl_2); HPLC analysis (CHIRALCEL OJ-H column, flow rate 1.0 mL/min, hexane−EtOH-*i*-PrOH (90:10:10), λ = 254 nm). The ^{1}H NMR spectral data were matched with the literature data.[3]

(*S*,*E*)-3-(1,3-Diphenylallyl)-7-methyl-1*H*-indole (13g)

The reaction was conducted according to general procedure using **11** (33 mg) and 7-methylindole (39 mg). Flash column chromatography with hexane−EtOAc (95:5) afforded **13g** (46 mg, 95%) as a pale orange solid. ^{1}H NMR (500 MHz, $CDCl_3$): *δ* 7.92 (bs, 1H), 7.37-7.18 (m, 11H), 6.99−6.92 (m, 3H), 6.73 (dd, *J* = 15.8, 7.4 Hz, 1H), 6.44

(d, J = 15.8 Hz, 1H), 5.11 (d, J = 7.4 Hz, 1H), 2.49 (s, 3H); $[\alpha]_D^{25}$ = +48.1 (c = 0.3, 96% ee, CH_2Cl_2); HPLC analysis (CHIRALCEL OD-H column, flow rate 0.5 mL/min, hexane−i-PrOH (90:10), λ = 254 nm). The ^{1}H NMR spectral data were matched with the literature data.[3]

7. Table S2. Optimizing the asymmetric allylic etherification using (*M*)-L1[a]

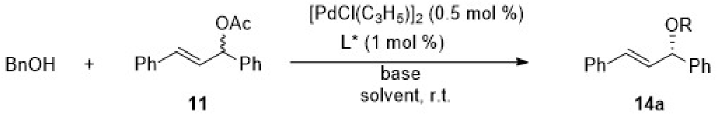

entry	solvent	base	Temp (°C)	time (h)	yield[b] (%)	ee[c] (%)
1	toluene	Cs_2CO_3	r.t.	2	87	90
2	THF	Cs_2CO_3	r.t.	24	58	92
3	CH_2Cl_2	Cs_2CO_3	r.t.	4	95	96
4	CH_3CN	Cs_2CO_3	r.t.	24	82	95
4	CH_2Cl_2	Cs_2CO_3	0	7	84	90
5	CH_2Cl_2	Cs_2CO_3	50	1	71	90
6	CH_2Cl_2	Na_2CO_3	r.t.	24	18	92
7	CH_2Cl_2	K_2CO_3	r.t.	24	84	94

[a]Conditions: **11** (0.15 mmol), benzyl alcohol (0.30 mmol), Pd/(*M*)-**L1** = 1/1 (1.0 mol % Pd), and base (0.45 mmol) in solvent (0.75 mL). [b]Isolated yields. [c]Determined by HPLC with a CHIRALPAK OD-H column.

8. Asymmetric allylic etherification (Figure 1c)

(*S*,*E*)-(3-(Benzyloxy)prop-1-ene-1,3-diyl)dibenzene (14a)

The reaction was conducted according to general procedure using **11** (38 mg) and benzyl alcohol (49 mg). Flash column chromatography with hexane−EtOAc (95:5) afforded **14a** (43 mg, 95%) as a clear oil. ^{1}H NMR (500 MHz, $CDCl_3$): δ 7.44−7.21 (m, 15H), 6.63 (d, J = 15.8 Hz, 1H), 6.34 (dd, J = 15.9, 7.0 Hz, 1H), 5.01 (d, J = 7.0 Hz, 1H), 4.58 (m, 2H); MS (ESI, Pos) m/z: $[M + Na]^+$ calcd for $C_{22}H_{20}NaO$ 323.1406, found 323.1431; $[\alpha]_D^{25}$ = +15.9 (c = 0.4, 96% ee, $CHCl_3$); HPLC analysis (CHIRALCEL OD-H column, flow rate 0.7 mL/min, hexane−i-PrOH (98:2), λ = 254 nm). The ^{1}H NMR spectral data were matched with the literature data.[4]

(*S*,*E*)-(3-methoxyprop-1-ene-1,3-diyl)dibenzene (14b)

The reaction was conducted according to general procedure using **11** (38 mg) and methanol (14 mg). Flash column chromatography with hexane−EtOAc (95:5) afforded **14b** (27 mg, 80%) as a clear oil. ^{1}H NMR (500 MHz, $CDCl_3$): δ 7.40−7.22 (m, 10H), 6.63 (d, J = 15.9 Hz, 1H), 6.28 (dd, J = 15.9, 6.9 Hz, 1H), 4.80 (d, J = 6.9 Hz, 1H), 3.38 (s, 3H); MS (ESI, Pos) m/z: $[M + Na]^+$ calcd for $C_{16}H_{16}NaO$ 247.1093, found 247.1122; $[\alpha]_D^{25}$ = −27.5 (c = 0.8, 93% ee, $CHCl_3$);

[4] Z. Liu, H. Du, *Org. Lett.* **2010**, *12*, 3054.

HPLC analysis (CHIRALCEL OD-H column, flow rate 1.0 mL/min, hexane−*i*-PrOH (98:2), λ = 254 nm). The ^{1}H NMR spectral data were matched with the literature data.[5]

(*S*,*E*)-(3-Ethoxyprop-1-ene-1,3-diyl)dibenzene (14c)

The reaction was conducted according to general procedure using **11** (33 mg) and ethanol (21mg). Flash column chromatography with hexane−EtOAc (95:5) afforded **14c (**30 mg, 84%) as a clear oil. ^{1}H NMR (500 MHz, $CDCl_3$): *δ* 7.40−7.21 (m, 10H), 6.60 (d, *J* = 15.8 Hz, 1H), 6.30 (dd, *J* = 15.9, 7.0 Hz, 1H), 4.92 (d, *J* = 7.1 Hz, 1H), 3.61−3.57 (m, 1H), 3.50−3.47 (m, 1H), 1.26 (t, *J* = 7.0 Hz, 3H) ; MS (ESI, Pos) *m/z*: $[M + Na]^+$ calcd for $C_{17}H_{18}NaO$ 261.1250, found 261.1273; $[\alpha]_D^{25}$ = −16.2 (*c* = 1.0, 90% ee, $CHCl_3$); HPLC analysis (CHIRAL Cellulose-SB column, flow rate 0.5 mL/min, hexane−*i*-PrOH (99:1), λ = 254 nm). The ^{1}H NMR spectral data were matched with the literature data.[5]

(*S*,*E*)-(3-(Allyloxy)prop-1-ene-1,3-diyl)dibenzene (14d)

The reaction was conducted according to general procedure using **11** (38 mg) and allyl alcohol (26mg). Flash column chromatography with hexane−EtOAc (95:5) afforded **14d** (23 mg, 61%) as a clear oil. ^{1}H NMR (500 MHz, $CDCl_3$): *δ* 7.41−7.34 (m, 6H), 7.31−7.25 (m, 3H), 7.24−7.21 (m, 1H), 6.62 (d, *J* = 15.9 Hz, 1H), 6.30 (dd, *J* = 15.9, 7.1 Hz, 1H), 5.97 (m, 1H), 5.30 (m, 1H), 5.19 (m, 1H), 4.98 (d, *J* = 7.0 Hz, 1H), 4.08−4.00 (m, 2H); MS (ESI, Pos) *m/z*: $[M + Na]^+$ calcd for $C_{18}H_{18}NaO$ 273.1250, found 273.1276; $[\alpha]_D^{25}$ = −4.7 (*c* = 0.6, 84% ee, $CHCl_3$); HPLC analysis (CHIRALCEL OD-H column, flow rate 0.5 mL/min, hexane−*i*-PrOH (98:2), λ = 254 nm). The ^{1}H NMR spectral data were matched with the literature data.[5]

(*S*,*E*)-2-(((1,3-Diphenylallyl)oxy)methyl)furan (14e)

The reaction was conducted according to general procedure using **11** (33 mg) and furfuryl alcohol (44mg). Flash column chromatography with hexane−EtOAc (95:5) afforded **14e** (40 mg, 92%) as a clear oil. ^{1}H NMR (500 MHz, $CDCl_3$): *δ* 7.42−7.22 (m, 11H), 6.61 (d, *J* =15.9 Hz, 1H), 6.34-6.29 (m, 3H), 5.02 (d, *J* = 7.0 Hz, 1H), 4.50 (s, 2H); MS (ESI, Pos) *m/z*: $[M + Na]^+$ calcd for $C_{20}H_{18}NaO_2$ 313.1199, found 313.1223; $[\alpha]_D^{25}$ = +16.0 (*c* = 0.8, 94% ee, $CHCl_3$); HPLC analysis (CHIRALCEL OD-H column, flow rate 0.5 mL/min, hexane−*i*-PrOH (99:1), λ = 254 nm). The ^{1}H NMR spectral data were matched with the literature data.[4]

5 Y. Li, W. Bao, *Adv. Synth. Catal.* **2009**, *351*, 865.

9. Table S3. Optimizing the asymmetric Suzuki–Miyaura coupling using (*P*)-L1 and -L2[a]

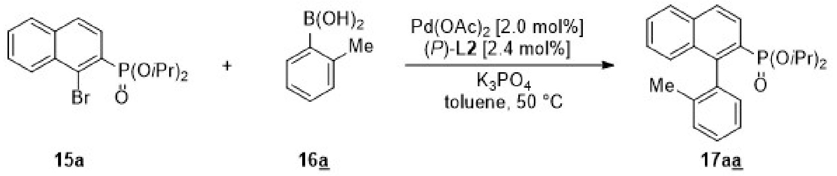

entry	L*	Pd	solvent	time (h)	yield[b] (%)	ee[c] (%)
1	**L1**	$Pd_2(dba)_3$	THF	48	30	83 (*R*)
2	**L1**	$Pd_2(dba)_3$	DCE	48	15	76 (*R*)
3	**L1**	$Pd_2(dba)_3$	DMF	48	trace[d]	—
4	**L1**	$Pd_2(dba)_3$	toluene	17	63	81 (*R*)
5	**L1**	$Pd(OAc)_2$	toluene	8	87	81 (*R*)
6	**L1**	$PdCl_2$	toluene	48	—[e]	—
7	**L1**	$Pd(PPh_3)_4$	toluene	48	trace[d]	—
8	**L2**	$Pd(OAc)_2$	toluene	4	97	95 (*R*)
9	**L2**	$Pd_2(dba)_3$	toluene	17	98	94 (*R*)
10[f]	**L2**	$Pd(OAc)_2$	toluene	84	98	96 (*R*)

[a] Conditions: Aryl bromide **15a** (0.1 mmol), arylboronic acid **16a** (0.2 mmol), (*P*)-**L***/Pd = 1.2 (2 mol% Pd), and K_3PO_4 (0.3 mmol) in solvent (0.5 mL), 50 °C. [b]Yields were determined by ^1H NMR analysis of the reaction mixture containing the dehalogenated product following purification by column chromatography. [c]Determined by HPLC using a CHIRALPAK IA column. [d]Major product was dehalogenated-**15a**. [e]No desired coupling product was detected. [f]Reaction carried out at 30 °C.

10. Asymmetric Suzuki–Miyaura coupling (Figure 6)

Diisopropyl (1-(*o*-tolyl)naphthalen-2-yl)phosphonate (17aa)

The reaction was conducted according to general procedure using **15a** (37 mg) and **16a** (27 mg). Flash column chromatography with hexane−EtOAc (7:3) afforded **17aa** as a pale yellow oil. IR (neat, cm^{-1}) 2977, 1559, 1489, 1453, 1384, 1373, 1243, 1171, 1141, 1107, 1006, 981, 890, 824, 756, 728; ^1H NMR (500 MHz, $CDCl_3$): δ 8.13 (dd, J = 12.4, 8.6 Hz, 1H), 7.92 (dd, J = 4.8, 3.7 Hz, 1H), 7.90 (d, J = 8.2 Hz, 1H), 7.55−7.52 (m, 1H), 7.38−7.33 (m, 2H), 7.30−7.20 (m, 4H), 4.65−4.54 (m, 2H), 1.90 (s, 3H), 1.24 (d, J = 6.2 Hz, 3H), 1.20 (d, J = 6.2 Hz, 3H), 1.13 (d, J = 6.2 Hz, 3H), 1.03 (d, J = 6.2 Hz, 3H); ^{13}C NMR (125 MHz, $CDCl_3$): δ 144.7, 144.6, 138.09, 138.05, 137.7, 134.89, 134.87, 132.6, 132.4, 130.9, 129.2, 128.2, 128.1, 128.0, 127.8, 127.6, 127.2, 127.1, 127.0, 126.9, 126.6, 125.4, 124.7, 70.54, 70.49, 70.3, 70.2, 24.1, 24.02, 23.96, 23.9, 23.83, 23.79, 23.52, 23.48, 20.1 (observed complexity due to C-P couplings); ^{31}P NMR (202 MHz, $CDCl_3$): δ 16.1; HRMS (ESI, Pos) *m/z*: $[M + Na]^+$ calcd for $C_{23}H_{27}NaO_3P$ 405.1590, found 405.1576; $[\alpha]_D^{25}$ = +3.4 (*c* = 2.8, 95% ee, $CHCl_3$); HPLC analysis (CHIRALPAK IA column, flow rate 1.0 mL/min, hexane− *i*-PrOH (99:1), λ = 254 nm).

Dimethyl (1-(*o*-tolyl)naphthalen-2-yl)phosphonate (17ba)

The reaction was conducted according to general procedure using **15b** (33 mg) and **16a** (27 mg). Flash column chromatography with hexane−EtOAc (7:3) afforded **17ba** as a pale yellow oil. ^{1}H NMR (500 MHz, $CDCl_3$): *δ* 8.06 (dd, *J* = 12.0, 8.5 H z, 1H), 7.94−7.90 (m, 2H), 7.58−7.55 (m, 1H), 7.41−7.29 (m, 5H), 7.22−7.20 (m, 1H), 3.51 (d, *J* = 12.0 Hz, 3H), 3.50 (d, *J* = 11.0 Hz, 3H), 1.92 (s, 3H); ^{31}P NMR (202 MHz, $CDCl_3$): *δ* 20.4; $[\alpha]_D^{25}$ = +22.0 (*c* = 0.1, 90% ee, $CHCl_3$); HPLC analysis (CHIRAL Cellulose-SB column, flow rate 1.0 mL/min, hexane−*i*-PrOH (90:10), λ = 254 nm). The ^{1}H NMR spectral data were matched with the literature data.[6]

Dibutyl (1-(*o*-tolyl)naphthalen-2-yl)phosphonate (17ca)

The reaction was conducted according to general procedure using **15c** (40 mg) and **16a** (27 mg). Flash column chromatography with hexane−EtOAc (7:3) afforded **17ca** as a pale yellow oil. IR (neat, cm^{-1}) 2956, 2340, 2323, 1739, 1378, 1231, 1065, 1024, 979, 913, 823, 748 ; ^{1}H NMR (500 MHz, $CDCl_3$): *δ* 8.08 (dd, *J* = 12.1, 8.6 Hz, 1H), 7.93−7.89 (m, 2H), 7.56−7.53 (m, 1H), 7.39−7.34 (m, 2H), 7.31−7.27 (m, 3H), 7.22−7.20 (m, 1H), 3.92−3.65 (m, 4H), 1.91 (s, 3H), 1.55−1.43 (m, 4H), 1.33−1.25 (m, 4H) , 0.90−0.86 (m, 6H); ^{13}C NMR (125 MHz, $CDCl_3$): *δ* 144.9, 144.8, 137.9, 137.6, 134.97, 134.95, 132.5, 132.4, 130.6, 129.3, 128.33, 128,25, 127.99, 127.96, 127.7, 127.3, 127.2, 127.1, 126.7, 125.8, 124.9, 65.62, 65.57, 65.52, 65.47, 32.50, 32.45, 32.42, 32.37, 18.8, 18.7, 13.6 (observed complexity due to C-P couplings); ^{31}P NMR (202 MHz, $CDCl_3$): *δ* 18.0; HRMS (ESI, Pos) *m/z*: $[M + Na]^+$ calcd for $C_{25}H_{31}NaO_3P$ 433.1903, found 433.1888; $[\alpha]_D^{25}$ = +18.0 (*c* = 0.1, 85% ee, $CHCl_3$); HPLC analysis (CHIRALCEL OD-H column, flow rate 0.5 mL/min, hexane−*i*-PrOH (95:5), λ = 254 nm).

Diphenyl (1-(*o*-tolyl)naphthalen-2-yl)phosphonate (17da)

The reaction was conducted according to general procedure using **15d** (44 mg) and **16a** (27 mg). Flash column chromatography with hexane−EtOAc (7:3) afforded **17da** as a pale yellow oil. ^{1}H NMR (500 MHz, $CDCl_3$): *δ* 8.33 (dd, *J* = 12.8, 8.6 Hz, 1H), 8.03 (dd, *J* = 8.5, 4.2 Hz, 1H), 7.95 (d, *J* = 8.2 Hz, 1H), 7.62−7.59 (m, 1H), 7.44−7.41 (m, 2H), 7.38−7.22 (m, 4H), 7.21−7.17 (m, 4H), 7.09−7.04 (m, 2H), 6.91−6.84 (m, 4H), 1.89 (s, 3H); ^{31}P NMR (202 MHz, $CDCl_3$): *δ* 10.7; $[\alpha]_D^{25}$ = −24.0 (*c* = 0.1, 83% ee, $CHCl_3$); HPLC analysis (CHIRALPAK IA column, flow rate 1.0 mL/min, hexane−*i*-PrOH (90:10), λ = 254 nm). The ^{1}H NMR spectral data were matched with the literature data.[7]

Diisopropyl (1-(2,4-dimethylphenyl)naphthalen-2-yl)phosphonate (17ab)

The reaction was conducted according to general procedure using **15a** (37 mg) and **16b** (30 mg). Flash column chromatography with hexane−EtOAc (7:3) afforded **17ab** as a pale yellow oil. IR (neat, cm^{-1}) 2976, 2918, 1739, 1498, 1453, 1374, 1241, 1175, 1141, 1106, 1006, 981, 889, 824, 770, 749, 684; ^{1}H NMR (500 MHz, $CDCl_3$): *δ* 8.09 (dd, *J* = 12.3, 8.5 Hz, 1H), 7.91−7.87 (m, 2H), 7.54−7.51 (m, 1H), 7.38−7.32 (m, 2H), 7.10−7.07 (m, 3H), 4.65−4.54 (m, 2H), 2.41 (s, 3H), 1.86 (s, 3H) 1.25 (d, *J* = 6.2 Hz, 3H), 1.20 (d, *J* = 6.2 Hz, 3H), 1.13 (d, *J* = 6.2 Hz, 3H), 1.05 (d, *J* = 6.2 Hz, 3H); ^{13}C NMR (125 MHz, $CDCl_3$): *δ* 144.94, 144.86, 137.4, 137.3, 135.09, 135.05, 134.87, 134.85, 132.8, 132.7, 130.8, 130.0, 128.2, 128.1, 127.9, 127.6, 127.2, 126.9, 126.5, 125.4, 70.5, 70.4, 70.3, 70.2, 24.1, 24.04, 23.96, 23.92, 23.81,

6 J. Yin, S. L. Buchwald, *J. Am. Chem. Soc.* **2000**, *122*, 12051.
7 X. Shen, G. O. Jones, D. A. Watson, B. Bhayana, S. L. Buchwald, *J. Am. Chem. Soc.* **2010**, *132*, 11278.

23.78, 23.51, 23.48, 21.3, 20.1 (observed complexity due to C-P couplings); ^{31}P NMR (202 MHz, $CDCl_3$): δ 16.1; HRMS (ESI, Pos) *m/z*: $[M + Na]^+$ calcd for $C_{24}H_{29}NaO_3P$ 419.1747, found 419.1743; $[\alpha]_D^{25}$ = −8.0 (*c* = 0.1, 89% ee, $CHCl_3$); HPLC analysis (CHIRALCEL OD-H column, flow rate 0.3 mL/min, hexane−*i*-PrOH (99:1), λ = 254 nm).

Diisopropyl (1-(2,5-dimethylphenyl)naphthalen-2-yl)phosphonate (17ac)

The reaction was conducted according to general procedure using **15a** (37 mg) and **16c** (30 mg). Flash column chromatography with hexane−EtOAc (7:3) afforded **17ac** as a pale yellow oil. IR (neat, cm^{-1}) 2978, 2367, 2341, 1789, 1499, 1456, 1373, 1234, 1167, 1141, 1107, 1012, 985, 909, 823, 731; ^{1}H NMR (500 MHz, $CDCl_3$): δ 8.13 (dd, *J* = 12.5, 8.6 Hz, 1H), 7.92−7.88 (m, 2H), 7.55−7.51 (m, 1H), 7.38−7.31 (m, 2H), 7.15−7.14 (m, 2H), 7.01 (s, 1H), 4.67−4.52 (m, 2H), 2.34 (s, 3H), 1.85 (s, 3H) 1.24 (d, *J* = 6.2 Hz, 3H), 1.18 (d, *J* = 6.2 Hz, 3H), 1.15 (d, *J* = 6.2 Hz, 3H), 1.02 (d, *J* = 6.2 Hz, 3H); ^{13}C NMR (125 MHz, $CDCl_3$): δ 144.9, 144.8, 137.9, 137.8, 134.88, 134.86, 133.7, 132.6, 132.4, 131.7, 129.1, 128.4, 128.3, 128.2, 127.9, 127.6, 127.2, 127.0, 126.9, 126.8, 126.5, 125.3, 70.44, 70.40, 70.14, 70.08, 24.08, 24.05, 24.0, 23.92, 23.89, 23.85, 23.5, 23.43, 21.0, 19.6 (observed complexity due to C-P couplings); ^{31}P NMR (202 MHz, $CDCl_3$): δ 16.2; HRMS (ESI, Pos) *m/z*: $[M + Na]^+$ calcd for $C_{24}H_{29}NaO_3P$ 419.1747, found 419.1747; $[\alpha]_D^{25}$ = +0.8 (*c* = 2.4, 93% ee $CHCl_3$); HPLC analysis (CHIRALCEL OD-H column, flow rate 0.5 mL/min, hexane−*i*-PrOH (99:1), λ = 254 nm).

Diisopropyl(1-(4-chloro-2-methylphenyl)naphthalen-2-yl)phosphonate (17ad)

The reaction was conducted for according to general procedure using **15ad** (37 mg) and **16d** (34 mg). Flash column chromatography with hexane−EtOAc (7:3) afforded **17ad** as a pale yellow oil. IR (neat, cm^{-1}) 2974, 1738, 1594, 1374, 1241, 1103, 1005, 981, 913, 892, 873, 824, 748 ; ^{1}H NMR (500 MHz, $CDCl_3$): δ 8.10 (dd, *J* = 12.4, 8.6 Hz, 1H), 7.94−7.89 (m, 2H), 7.57−7.54 (m, 1H), 7.40−7.37 (m, 1H), 7.30−7.24 (m, 3H), 7.14 (d, *J* = 8.1 Hz, 1H), 4.68−4.57 (m, 2H), 1.89 (s, 3H), 1.26 (d, *J* = 6.2 Hz, 3H), 1.21 (d, *J* = 6.2 Hz, 3H), 1.14 (d, *J* = 6.2 Hz, 3H), 1.08 (d, *J* = 6.2 Hz, 3H); ^{13}C NMR (125 MHz, $CDCl_3$): δ 143.23, 143.15 , 139.9, 136.7, 134.9, 133.5, 132.2, 129.2, 128.12, 128.09, 128.0, 127.8, 127.5, 127.4, 126.9, 126.8, 124.9, 70.73, 70.68, 70.5, 70.4, 24.1, 24.03, 23.97, 23.9, 23.83, 23.80, 23.54, 23.50, 20.0 (observed complexity due to C-P couplings); ^{31}P NMR (202 MHz, $CDCl_3$): δ 15.8; HRMS (ESI, Pos) *m/z*: $[M + Na]^+$ calcd for $C_{23}H_{26}NaClO_3P$ 439.1200, found 439.1200; $[\alpha]_D^{25}$ = −3.0 (*c* = 0.4, 95% ee, $CHCl_3$); HPLC analysis (CHIRALCEL OD-H column, flow rate 0.5 mL/min, hexane−*i*-PrOH (99:1), λ = 254 nm).

Diisopropyl(1-(4-methoxy-2-methylphenyl)naphthalen-2-yl)phosphonate (17ae)

The reaction was conducted according to general procedure using **15a** (37 mg) and **16e** (33 mg). Flash column chromatography with hexane−EtOAc (7:3) afforded **17ae** as a pale yellow oil. IR (neat, cm^{-1}) 2981, 1739, 1608, 1497, 1465, 1373, 1239, 1163, 1141, 1106, 1006, 982, 913, 889, 823, 748, 733, 685; ^{1}H NMR (500 MHz, $CDCl_3$): δ 8.10 (dd, *J* = 12.4, 8.6 Hz, 1H), 7.91−7.87 (m, 2H), 7.55−7.51 (m, 1H), 7.39−7.33 (m, 2H), 7.11 (d, 8.3 Hz, 1H), 6.85-6.80 (m, 2H), 4.64−4.57 (m, 2H), 3.88 (s, 3H), 1.88 (s, 3H), 1.26 (d, *J* = 6.2 Hz, 3H), 1.21 (d, *J* = 6.2 Hz, 3H), 1.14 (d, *J* = 6.2 Hz, 3H), 1.08 (d, *J* = 6.2 Hz, 3H); ^{13}C NMR (125 MHz, $CDCl_3$): δ 159.2, 144.6, 144.5, 139.2, 134.91, 134.88, 133.1, 133.0, 131.9, 130.50, 130.46, 128.2, 128.1, 127.9, 127.5, 127.1, 127.0, 126.6, 125.9, 114.8, 109.9, 70.5, 70.4, 70.3, 70.2, 24.10, 24.07, 23.99, 23.95, 23.9, 23.8, 23.7, 23.6, 20.4 (observed complexity due to C-P couplings); ^{31}P NMR (202 MHz, $CDCl_3$): δ 16.2; HRMS (ESI, Pos) *m/z*: $[M + Na]^+$ calcd for $C_{24}H_{29}NaO_4P$ 435.1696, found 435.1694; $[\alpha]_D^{25}$ = −9.9 (*c* = 2.8, 87% ee,

CHCl$_3$); HPLC analysis (CHIRAL ART Amylose-SA column, flow rate 0.5 mL/min, hexane−*i*-PrOH (97:3), λ = 254 nm).

Diisopropyl (1-(2-ethylphenyl)naphthalen-2-yl)phosphonate (17af)

The reaction was conducted according to general procedure using **15a** (37 mg) and **16f** (30 mg). Flash column chromatography with hexane−EtOAc (7:3) afforded **17af** as a pale yellow oil. IR (neat, cm^{-1}) 2976, 1739, 1456, 1373, 1241, 1176, 1141, 1107, 1006, 980, 913, 891, 824, 770, 749 ;^{1}H NMR (500 MHz, CDCl$_3$): δ 8.12 (dd, *J* = 12.4, 8.6 Hz, 1H), 7.93−7.88 (m, 2H), 7.55−7.51 (m, 1H), 7.42-7.26 (m, 4H), 7.25−7.23 (m, 1H), 7.19−7.17 (m, 1H), 4.64−4.54 (m, 2H), 2.35−2.30 (m, 1H) 2.14−2.09 (m, 1H), 1.24 (d, *J* = 6.2 Hz, 3H), 1.18 (d, *J* = 6.2 Hz, 3H), 1.12 (d, *J* = 6.2 Hz, 3H), 1.02 (d, *J* = 6.2 Hz, 3H), 0.98 (t, *J* = 7.6 Hz, 3H); ^{13}C NMR (125 MHz, CDCl$_3$): δ 144.5, 143.1, 137.61, 137.58, 134.83, 134.81, 133.0, 132.8, 130.8, 128.2, 128.1, 127.96, 127.87, 127.6, 127.5, 127.1, 126.9, 126.4, 125.5, 124.6, 70.47, 70.42, 70.2, 70.1, 26.0, 24.03, 24.00, 23.97, 23.9, 23.82, 23.78, 23.53, 23.50, 13.9 (observed complexity due to C-P couplings); ^{31}P NMR (202 MHz, CDCl$_3$): δ 16.2; HRMS (ESI, Pos) *m/z*: [M + Na]$^+$ calcd for $C_{24}H_{29}NaO_3P$ 419.1747, found 419.1739; $[\alpha]_D^{25}$ = −3.5 (*c* = 0.5, 99% ee, CHCl$_3$); HPLC analysis (CHIRALPAK IA column, flow rate 1.0 mL/min, hexane−*i*-PrOH (99:1), λ = 254 nm).

11. Table S4. Scope of substrate for asymmetric Suzuki–Miyaura coupling using L1 and L2[a]

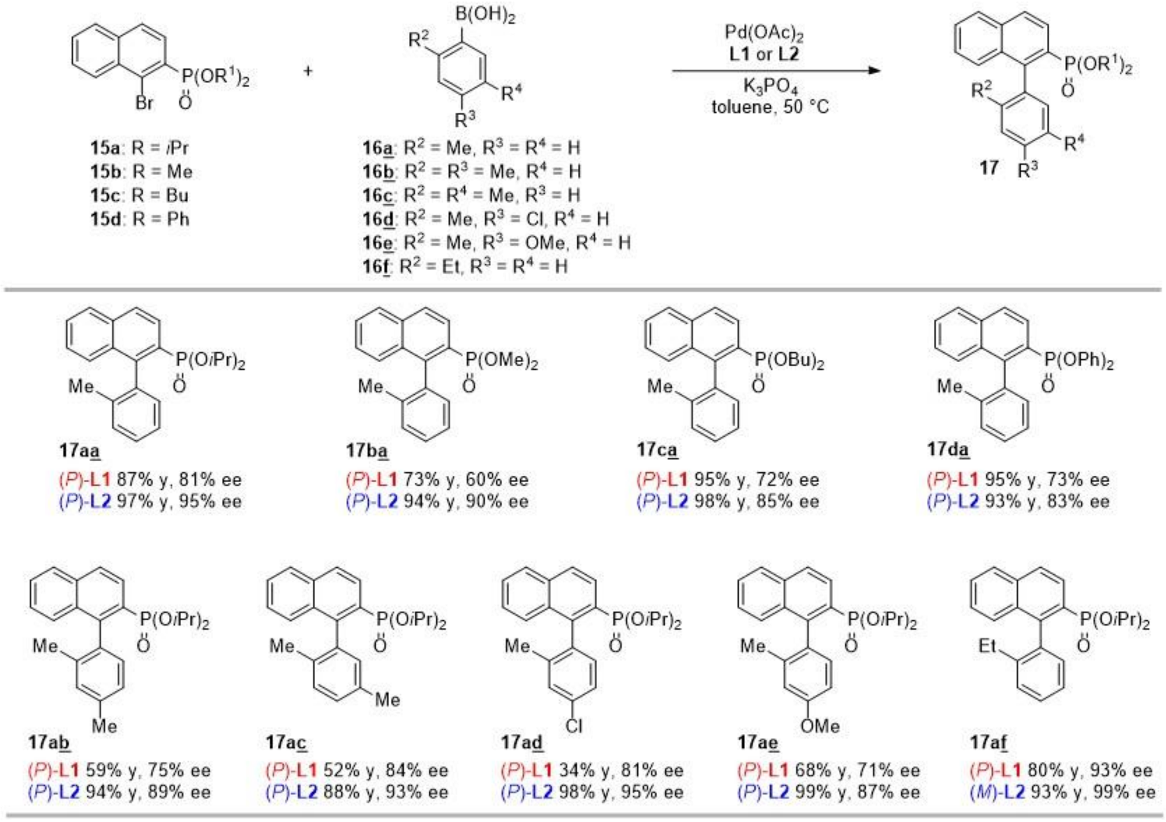

[a]Conditions: Aryl bromide (0.1 mmol), aryl boronic acid (0.2 mmol), **L***/Pd = 1.2 (2 mol % Pd), and K_3PO_4 (0.3 mmol) in toluene (0.5 mL) at 50 °C.

12. DFT calculation methods and data

All calculations were carried out with the D.01 revision of the Gaussian 09 program package.[8, 9]

The three dimensional structures presented in Figure S1 and S2 were visualized utilizing CYLview.[10]

12-1. Computational methods for (*π*-ally)palladium intermediates with (*M*)-L1

The geometries of all intermediates (**IM-*endo*** and ***-exo***) were optimized at the DFT level by using the B3PW91. The standard 6-31G* basis set has been applied for atoms except Pd, which has been described by the LANL2DZ basis set.[11] Frequencies were analytically computed at the same level of theory to give gas-phase free energies and to confirm whether the structures are minima (no imaginary frequencies) or transition states (only one imaginary frequency). The Gibbs free energies were in all cases computed by adding to the single-point energies both zero-point vibrational energies and thermal corrections (298 K, 1 atm).

12-2. Computational methods for transitions states (TSs) for the reductive elimination steps

The geometries of all transition states were optimized at the DFT level by using the dispersion-corrected functional B97-D. The standard 6-31G* basis set has been applied for atoms except Pd, which has been described by the LANL2DZ basis set. Frequencies were analytically computed at the same level of theory to give gas-phase free energies and to confirm whether the structures are minima (no imaginary frequencies) or transition states (only one imaginary frequency). All transition state structures were confirmed to connect the proposed reactants and products by intrinsic reaction coordinate (IRC) calculations.[12] The Gibbs free energies were in all cases computed by adding to the single-point energies both zero-point vibrational energies and thermal corrections (298K, 1 atm) obtained at the level of theory employed in the geometry optimization. The single-point calculations with the SMD solvation model (toluene) were carried out at the same level theory on the previously optimized structures.

---

[8] Gaussian 09, Revision **D.01**, M. J. Frisch, G. W. Trucks, H. B. Schlegel, G. E. Scuseria, M. A. Robb, J. R. Cheeseman, G. Scalmani, V. Barone, B. Mennucci, G. A. Petersson, H. Nakatsuji, M. Caricato, X. Li, H. P. Hratchian, A. F. Izmaylov, J. Bloino, G. Zheng, J. L. Sonnenberg, M. Hada, M. Ehara, K. Toyota, R. Fukuda, J. Hasegawa, M. Ishida, T. Nakajima, Y. Honda, O. Kitao, H. Nakai, T. Vreven, J. A. Montgomery, Jr., J. E. Peralta, F. Ogliaro, M. Bearpark, J. J. Heyd, E. Brothers, K. N. Kudin, V. N. Staroverov, R. Kobayashi, J. Normand, K. Raghavachari, A. Rendell, J. C. Burant, S. S. Iyengar, J. Tomasi, M. Cossi, N. Rega, J. M. Millam, M. Klene, J. E. Knox, J. B. Cross, V. Bakken, C. Adamo, J. Jaramillo, R. Gomperts, R. E. Stratmann, O. Yazyev, A. J. Austin, R. Cammi, C. Pomelli, J. W. Ochterski, R. L. Martin, K. Morokuma, V. G. Zakrzewski, G. A. Voth, P. Salvador, J. J. Dannenberg, S. Dapprich, A. D. Daniels, Ö. Farkas, J. B. Foresman, J. V. Ortiz, J. Cioslowski, and D. J. Fox, Gaussian, Inc., Wallingford CT, 2009.

[9] Calculations were performed with the Gaussian 09 program on a TATARA system at Kyushu University.

[10] C. Y. Legault, CYLview, version 1.0b; Universitéde Sherbrooke: Sherbrooke, Canada, 2009; http://www.cylview.org.

[11] a) P. J. Hay, W. R. Wadt, *J. Chem. Phys.* **1985**, *82*, 270; b) W. R. Wadt, P. J. Hay, *J. Chem. Phys.* **1985**, *82*, 284. c) P. J. Hay, W. R. Wadt, *J. Chem. Phys.* **1985**, *82*, 299.

[12] a) C. Gonzalez, H. B. Schlegel, *J. Chem. Phys.* **1989**, *90*, 2154; b) C. Gonzalez, H. B. Schlegel, *J. Phys. Chem.* **1990**, *94*, 5523.

12-3. Computational data for transitions states (TSs)

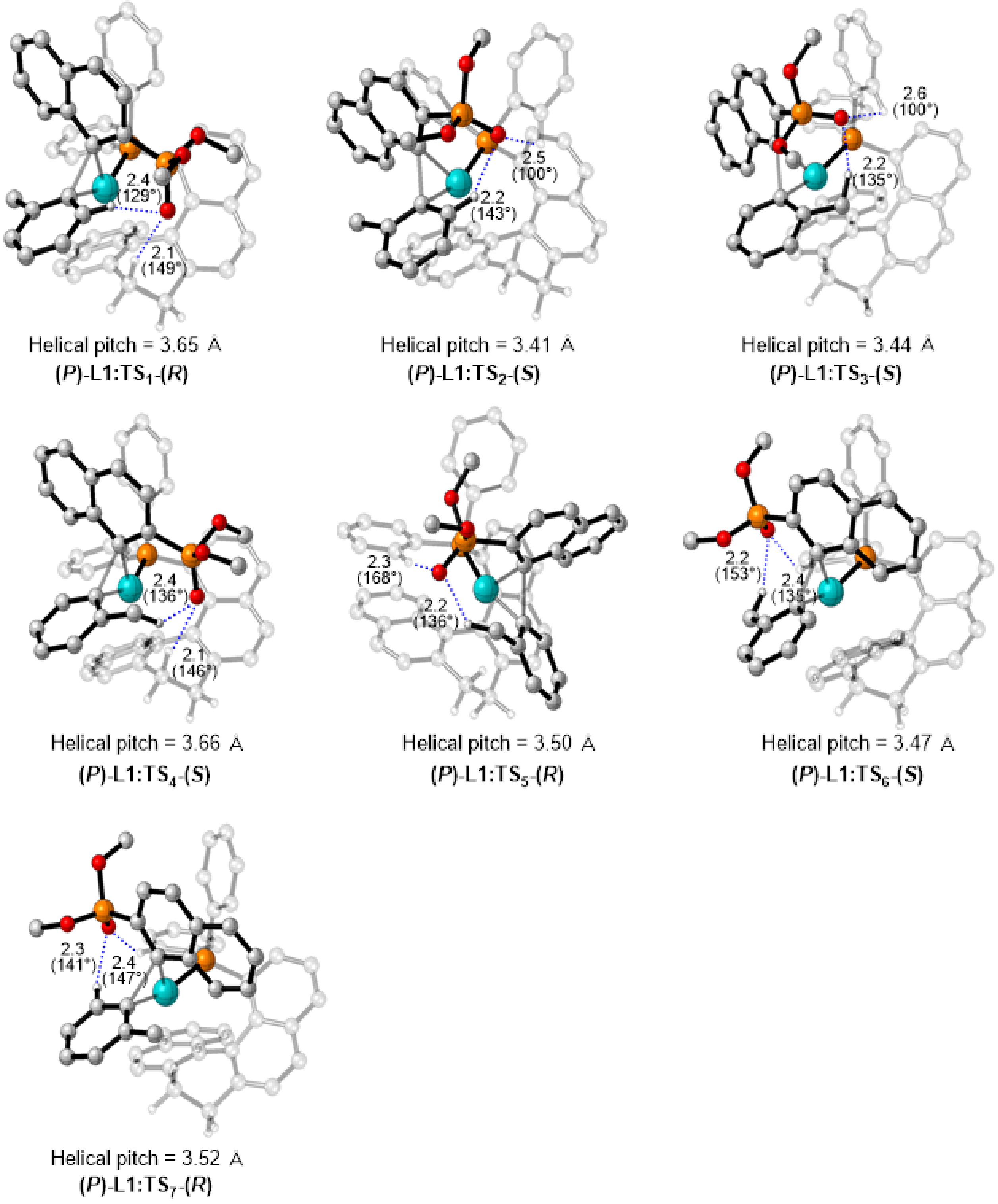

Figure S1. The optimized structures of the transition states (**TS**$_{1\text{-}7}$) for reductive elimination of axially chiral biaryls from Pd(**L1**) complexes. Most of the hydrogen atoms have been omitted for clarity. Hydrogen-bonding interactions are designated by dashed blue lines, with distances in Å and H–O–P bond angles in degrees shown.

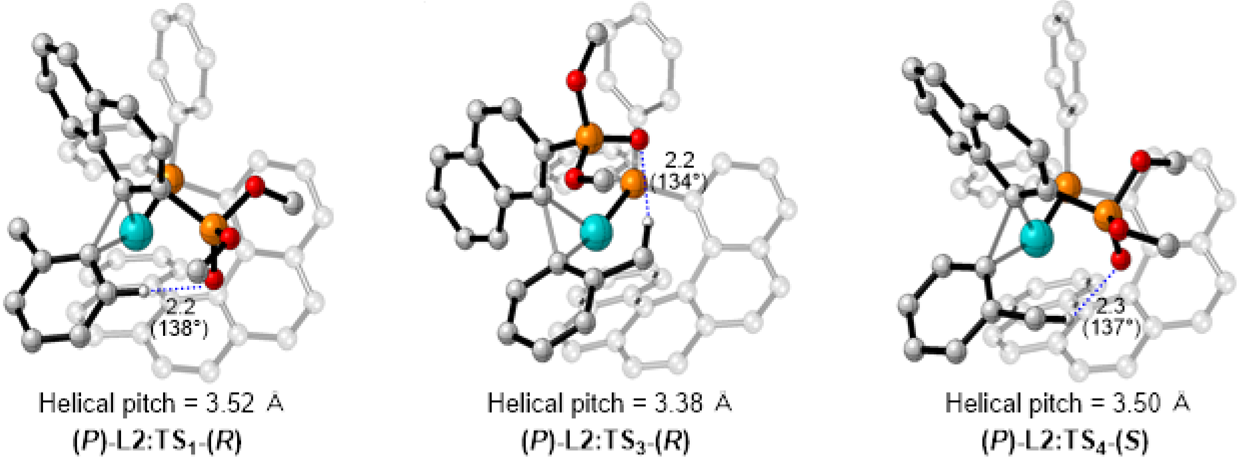

Figure continued on the next page.

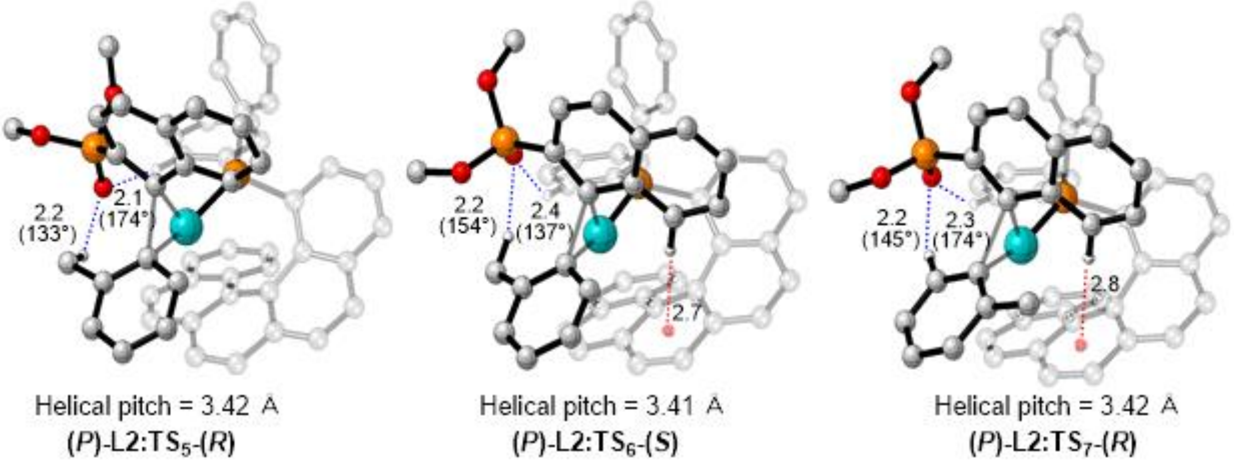

Figure S2. The optimized structures of the transition states (**TS**$_{1\text{-}7}$) for reductive elimination of axially chiral biaryls from Pd(**L2**) complexes. Most of the hydrogen atoms have been omitted for clarity. Hydrogen-bonding interactions are designated by dashed blue lines, with distances in Å and H–O–P bond angles in degrees shown.CH/π-interactions are designated by dashed red lines.

13. Table S5. Cartesian coordinates

Im-*endo*

E_{gas}	-2357.331373	a.u.
H_{gas}	-2356.56505	a.u.
G_{gas}	-2356.685076	a.u.

Symbol	X	Y	Z
P	-0.115341	1.723609	-3.846677
C	0.582522	1.263324	-2.208614
C	-0.307041	1.061118	-1.14086
H	-1.376687	1.191701	-1.294293
C	0.169865	0.715709	0.11981
H	-0.527599	0.570548	0.940537
C	1.543007	0.564207	0.331119
H	1.915711	0.298284	1.316515
C	2.430381	0.759258	-0.723987
H	3.499552	0.645778	-0.565847
C	1.955303	1.103299	-1.991751
H	2.657428	1.250759	-2.806247
C	-0.800273	3.401816	-3.489975
C	-0.068839	4.170158	-2.592915
H	0.878233	3.794895	-2.216185
C	-0.531511	5.40696	-2.107452
H	0.088212	5.987606	-1.430679
C	-1.793877	5.825032	-2.444063
H	-2.204848	6.740146	-2.024683
C	-2.604883	5.05797	-3.315377
C	-3.944871	5.453931	-3.54924
H	-4.30978	6.362279	-3.076579
C	-4.780859	4.676554	-4.307845
H	-5.827892	4.944155	-4.42535
C	-4.281386	3.536809	-4.971719
C	-5.199172	2.636329	-5.759937
H	-6.230921	2.764243	-5.414063
H	-5.186709	2.893539	-6.829726
C	-4.739657	1.186261	-5.56734
H	-5.394395	0.485677	-6.093504
H	-4.792507	0.943503	-4.499527
C	-3.33283	1.09298	-6.122386
C	-3.027373	0.180229	-7.178991
H	-3.699089	-0.658433	-7.344018
C	-1.948974	0.383455	-7.997652
H	-1.73055	-0.315991	-8.801397
C	-1.165038	1.568388	-7.889274
C	-0.191544	1.878042	-8.865294
H	0.005873	1.154879	-9.653154
C	0.464006	3.09066	-8.850139
H	1.194797	3.328241	-9.617845
C	0.161932	4.030409	-7.849567
H	0.650621	5.000638	-7.855083
C	-0.753149	3.735244	-6.856813
H	-0.973697	4.479798	-6.10082
C	-1.428285	2.495067	-6.833653
C	-2.459366	2.173813	-5.854195
C	-2.931369	3.194721	-4.8776
C	-2.088671	3.86424	-3.930106
C	1.361032	1.931648	-4.912016
C	1.805774	0.821272	-5.640284
H	1.234698	-0.1019	-5.620544

Symbol	X	Y	Z
C	2.985732	0.88943	−6.378513
H	3.322936	0.018337	−6.933431
C	3.727912	2.068583	−6.403181
H	4.648621	2.121917	−6.978006
C	3.284175	3.182482	−5.690427
H	3.855005	4.107003	−5.711929
C	2.107185	3.117812	−4.948513
H	1.769653	3.996064	−4.407498
Pd	−1.734162	0.191984	−4.571834
C	−2.187362	−2.016507	−4.21714
H	−2.046964	−2.662576	−5.079742
C	−3.465648	−1.650311	−3.84294
H	−3.568946	−1.09022	−2.912063
C	−1.023731	−1.53901	−3.518401
C	0.306476	−2.104606	−3.803483
C	1.251041	−2.183902	−2.767226
C	0.655534	−2.621963	−5.065442
C	2.497519	−2.766447	−2.980128
H	0.996683	−1.797533	−1.783903
C	1.900004	−3.204455	−5.275848
C	2.826704	−3.279892	−4.233222
H	3.209958	−2.825606	−2.162009
H	2.149029	−3.604594	−6.255135
H	3.796918	−3.740142	−4.398336
H	−1.170561	−1.245186	−2.478022
C	−4.71527	−2.074335	−4.452588
C	−5.919888	−1.742415	−3.804136
C	−4.779972	−2.825025	−5.643703
C	−7.14606	−2.142404	−4.323949
H	−5.885814	−1.174111	−2.877132
C	−6.005186	−3.220092	−6.163166
H	−3.868502	−3.107423	−6.162844
C	−7.192113	−2.87955	−5.506862
H	−8.064734	−1.88357	−3.80538
H	−6.039974	−3.802096	−7.079861
H	−8.147961	−3.195051	−5.915535
H	−0.045358	−2.558071	−5.894641

Im-*exo*

E_{gas}	-2357.326077	a.u.
H_{gas}	-2356.560117	a.u.
G_{gas}	-2356.68077	a.u.

Symbol	X	Y	Z
P	-0.228099	1.62424	-4.26077
C	0.971048	1.0811	-2.978883
C	0.661456	1.261563	-1.621647
H	-0.28183	1.713887	-1.330425
C	1.570211	0.887285	-0.636962
H	1.321767	1.0446	0.408648
C	2.791487	0.311998	-0.989813
H	3.503466	0.027965	-0.219563
C	3.094324	0.103577	-2.333082
H	4.044047	-0.341973	-2.617043
C	2.190671	0.484958	-3.325524
H	2.454037	0.339633	-4.368593
C	-0.638662	3.325547	-3.687431
C	0.367268	4.0473	-3.059626
H	1.382643	3.662083	-3.055201
C	0.103197	5.236606	-2.352489
H	0.922039	5.787471	-1.899389
C	-1.198808	5.630694	-2.166929
H	-1.433517	6.489129	-1.542137
C	-2.266377	4.916799	-2.765751
C	-3.610014	5.274577	-2.491746
H	-3.803046	6.107582	-1.820534
C	-4.647309	4.553622	-3.027066
H	-5.674904	4.787974	-2.760474
C	-4.396165	3.538159	-3.97467
C	-5.527336	2.745329	-4.57982
H	-6.382158	2.737928	-3.894112
H	-5.874404	3.209174	-5.515403
C	-5.047128	1.316875	-4.86239
H	-5.844155	0.706054	-5.2973
H	-4.758092	0.851533	-3.909338
C	-3.884768	1.413591	-5.831819
C	-3.953719	0.7726	-7.10489
H	-4.692597	-0.009028	-7.251566
C	-3.141836	1.158627	-8.13448
H	-3.20788	0.66312	-9.100345
C	-2.275306	2.278052	-7.998629
C	-1.578624	2.78201	-9.122149
H	-1.644294	2.237872	-10.061377
C	-0.869828	3.959525	-9.045756
H	-0.355859	4.34892	-9.919987
C	-0.845197	4.674903	-7.834466
H	-0.32735	5.62869	-7.781254
C	-1.478909	4.183678	-6.711207
H	-1.453419	4.766002	-5.79904
C	-2.188521	2.959293	-6.744231
C	-2.935696	2.439255	-5.607337
C	-3.088092	3.24381	-4.362342
C	-1.989825	3.811543	-3.638409
C	0.804315	1.699862	-5.775915
C	0.823209	0.58378	-6.622797
H	0.15597	-0.2517	-6.423246
C	1.690349	0.536475	-7.712997
H	1.698966	-0.336737	-8.359809
C	2.541103	1.609494	-7.972266
H	3.218585	1.574696	-8.821095
C	2.517415	2.730626	-7.143013
H	3.173264	3.572792	-7.346485
C	1.655664	2.777897	-6.049777
H	1.643089	3.659316	-5.416474
Pd	-2.093611	0.271267	-4.667841
C	-2.823104	-1.804507	-4.073608
H	-3.624707	-1.821004	-3.338515
C	-3.098196	-2.072469	-5.398874
H	-2.247788	-2.136592	-6.079317
C	-1.492114	-1.487114	-3.614821
C	-1.226077	-1.4795	-2.164152
C	-0.104136	-2.159947	-1.66875
C	-2.118036	-0.897807	-1.24612
C	0.105189	-2.282982	-0.298013
H	0.597247	-2.613523	-2.364624
C	-1.903812	-1.012921	0.122603
C	-0.795128	-1.714322	0.601697
H	0.971702	-2.826944	0.06733
H	-2.603864	-0.559153	0.819139
H	-0.634949	-1.814701	1.671748
H	-0.672714	-1.880924	-4.219574
C	-4.377744	-2.452386	-5.975184
C	-4.408345	-2.842423	-7.327676
C	-5.579903	-2.48091	-5.241151
C	-5.59532	-3.247175	-7.92853
H	-3.48611	-2.832283	-7.904294
C	-6.764274	-2.88349	-5.8434
H	-5.588769	-2.193591	-4.193691
C	-6.776946	-3.266983	-7.187921
H	-5.598919	-3.551298	-8.971321
H	-7.683103	-2.906003	-5.264234
H	-7.705834	-3.585045	-7.652896
H	-2.979252	-0.341339	-1.610569

(*P*)-L1:TS$_1$-(*R*)

$E_{toluene}$	-3079.941221	a.u.
$H_{toluene}$	-3079.084426	a.u.
$G_{toluene}$	-3079.220552	a.u.

Symbol	X	Y	Z	Symbol	X	Y	Z
P	0.960457	-0.717562	-1.041283	C	2.934296	-4.013506	0.442538
C	-0.171818	-1.492521	-2.272094	H	2.877472	-4.620648	1.348397
C	-1.059342	-0.680167	-3.005361	C	3.808721	-4.370264	-0.593444
H	-0.981171	0.400827	-2.947105	H	4.428158	-5.265314	-0.503819
C	-2.06233	-1.257512	-3.794565	C	3.893569	-3.567565	-1.744796
H	-2.748844	-0.609707	-4.343089	H	4.579956	-3.836566	-2.550827
C	-2.197523	-2.652756	-3.85826	C	3.093906	-2.424138	-1.864046
H	-2.989923	-3.102594	-4.460559	H	3.160214	-1.804007	-2.758541
C	-1.315789	-3.467565	-3.13301	Pd	-0.11972	0.081149	0.841076
H	-1.418275	-4.553875	-3.165257	C	-3.321359	-4.461537	-0.227294
C	-0.308247	-2.894675	-2.343572	C	-4.127759	-3.42474	-0.674144
H	0.356498	-3.540973	-1.772294	C	-3.737251	-2.063595	-0.523371
C	1.786478	0.670576	-1.946204	C	-2.51613	-1.747426	0.176697
C	1.944007	0.534456	-3.326687	C	-1.690008	-2.838724	0.548272
H	1.677695	-0.41166	-3.798493	C	-2.069853	-4.162141	0.361541
C	2.363181	1.608335	-4.149904	H	-5.418377	-1.254113	-1.649788
H	2.494605	1.453162	-5.222438	H	-3.631777	-5.499916	-0.364876
C	2.53059	2.859372	-3.586953	H	-5.068188	-3.635607	-1.189834
H	2.776898	3.724561	-4.207763	C	-4.488645	-1.016718	-1.128734
C	2.346685	3.065301	-2.18903	C	-2.108228	-0.356302	0.404162
C	2.388018	4.388909	-1.666091	H	-0.703191	-2.613356	0.93415
H	2.596187	5.214786	-2.350569	H	-1.387453	-4.966479	0.645496
C	2.107661	4.62257	-0.333839	C	-2.790557	0.613089	-0.420167
H	2.058198	5.642552	0.054125	C	-3.9762	0.262849	-1.138882
C	1.923302	3.541281	0.562251	H	-4.488769	1.03713	-1.711117
C	1.626432	3.807098	2.027752	P	-2.234272	2.309935	-0.739678
H	1.168936	4.803839	2.128608	O	-3.613704	3.188478	-0.996548
H	2.567381	3.813426	2.611213	O	-1.699772	2.265899	-2.282384
C	0.682419	2.718036	2.583074	O	-1.293244	2.983864	0.214464
H	0.493053	2.872429	3.656386	C	-4.414894	3.488241	0.162356
H	-0.270977	2.773425	2.047334	H	-3.819179	4.022465	0.920913
C	1.370732	1.377812	2.381143	H	-5.23642	4.130126	-0.184608
C	1.538662	0.485846	3.48624	H	-4.827718	2.562602	0.598822
H	0.898912	0.630948	4.358267	C	-1.027441	3.445547	-2.792488
C	2.475646	-0.523746	3.45618	H	-0.476549	3.115704	-3.68252
H	2.587985	-1.206116	4.302132	H	-1.774933	4.208491	-3.06113
C	3.370519	-0.646725	2.350615	H	-0.324171	3.843454	-2.048777
C	4.437611	-1.585142	2.382521	C	-2.615754	1.286669	2.512738
H	4.517037	-2.248887	3.24738	C	-1.895106	0.099528	2.243192
C	5.366326	-1.650571	1.354608	C	-1.641528	-0.763355	3.363975
H	6.178676	-2.378889	1.390505	C	-2.15397	-0.403139	4.630969
C	5.258733	-0.759325	0.261342	C	-2.878437	0.771365	4.857015
H	5.997969	-0.791091	-0.541169	C	-3.093402	1.636862	3.777448
C	4.212273	0.152221	0.190586	H	-2.813421	1.973495	1.702619
H	4.152641	0.824445	-0.661208	H	-1.944354	-1.067956	5.47384
C	3.221044	0.221336	1.2076	H	-3.256514	1.004631	5.854799
C	2.118028	1.173709	1.183345	H	-3.638654	2.574926	3.907139
C	2.01765	2.213976	0.110962	C	-0.781212	-2.016633	3.36104
C	2.061296	1.945943	-1.311219	H	-0.40894	-2.213148	4.379123
C	2.204775	-2.067096	-0.831119	H	-1.348889	-2.900842	3.035464
C	2.13828	-2.863215	0.324955	H	0.095835	-1.902242	2.712328
H	1.469972	-2.568764	1.134373				

(*P*)-L1:TS$_2$-(*S*)

$E_{toluene}$	-3079.935723	a.u.
$H_{toluene}$	-3079.080054	a.u.
$G_{toluene}$	-3079.213637	a.u.

Symbol	X	Y	Z	Symbol	X	Y	Z
P	0.853273	0.768504	1.166102	C	0.784297	4.270776	-0.984231
C	-0.213041	1.415716	2.538599	H	0.011688	4.945287	-1.355161
C	-0.91749	0.457511	3.293166	C	2.13157	4.485255	-1.315343
H	-0.909452	-0.585821	2.982544	H	2.413345	5.339088	-1.935111
C	-1.653711	0.846255	4.421042	C	3.111752	3.590763	-0.858865
H	-2.203317	0.093763	4.989367	H	4.161365	3.745822	-1.119136
C	-1.708702	2.198626	4.796577	C	2.75098	2.491295	-0.066469
H	-2.292305	2.502861	5.668307	H	3.5146	1.796271	0.278693
C	-1.018195	3.160156	4.040462	Pd	-0.207316	-0.723202	-0.258395
H	-1.057465	4.214375	4.324525	C	0.523603	3.008273	-4.177196
C	-0.264083	2.770319	2.922456	C	-0.804076	3.24729	-3.86384
H	0.285334	3.520869	2.353859	C	-1.495009	2.44103	-2.910935
C	2.305307	0.288203	2.235001	C	-0.8265	1.308054	-2.31435
C	2.926788	1.305774	2.957825	C	0.550367	1.124827	-2.643394
H	2.742287	2.344066	2.678382	C	1.213879	1.9469	-3.538307
C	3.725611	1.033503	4.095465	H	-3.314509	3.634754	-2.943652
H	4.215928	1.852805	4.624702	H	1.043111	3.64743	-4.894935
C	3.783003	-0.259845	4.585652	H	-1.341698	4.084285	-4.317326
H	4.292665	-0.479743	5.52684	C	-2.810745	2.777473	-2.491283
C	3.150698	-1.331892	3.892171	C	-1.528867	0.447587	-1.360923
C	3.109517	-2.637376	4.457959	H	1.096465	0.305631	-2.175831
H	3.582021	-2.804875	5.428529	H	2.271338	1.772418	-3.742023
C	2.430166	-3.653539	3.811214	C	-2.790025	0.90847	-0.898115
H	2.31782	-4.633367	4.281333	C	-3.411757	2.057465	-1.477921
C	1.924254	-3.464147	2.500128	H	-4.389787	2.366296	-1.107336
C	1.196992	-4.593085	1.791541	P	-3.73803	0.113902	0.440491
H	0.687721	-5.221174	2.540379	O	-4.897939	-0.815874	-0.232487
H	1.909787	-5.243427	1.249289	O	-4.673638	1.327526	1.036711
C	0.185899	-3.994033	0.79174	O	-2.962674	-0.616135	1.493698
H	-0.376087	-4.772883	0.257588	C	-5.180588	-0.765634	-1.648086
H	-0.540702	-3.372422	1.341915	H	-5.938136	-1.540561	-1.825099
C	0.979558	-3.151347	-0.183527	H	-5.580482	0.219697	-1.935248
C	0.914837	-3.40634	-1.582427	H	-4.2773	-0.988472	-2.234822
H	0.152615	-4.09396	-1.948963	C	-3.988084	2.320547	1.838189
C	1.746004	-2.742535	-2.457101	H	-3.315068	2.924079	1.206391
H	1.661167	-2.901645	-3.53331	H	-4.77199	2.96329	2.261649
C	2.734447	-1.837258	-1.971559	H	-3.410905	1.845251	2.643133
C	3.606979	-1.172317	-2.880225	C	-2.306756	-2.433725	-0.92758
H	3.44595	-1.320978	-3.950813	C	-1.575238	-1.550217	-1.76539
C	4.649664	-0.380174	-2.424486	C	-1.449125	-1.897468	-3.146259
H	5.313737	0.121356	-3.131396	C	-2.050257	-3.096621	-3.595168
C	4.875547	-0.267847	-1.030059	C	-2.735804	-3.972913	-2.745548
H	5.732654	0.300189	-0.661969	C	-2.856578	-3.635291	-1.392684
C	4.020759	-0.876692	-0.118905	H	-2.436773	-2.201296	0.126041
H	4.23586	-0.783322	0.940844	H	-1.961508	-3.346991	-4.656139
C	2.887174	-1.626909	-0.54778	H	-3.173166	-4.893068	-3.139914
C	1.925787	-2.245866	0.35509	H	-3.383155	-4.287965	-0.692458
C	2.08608	-2.22907	1.840647	C	-0.719251	-1.096933	-4.204968
C	2.511939	-1.085805	2.614511	H	-0.744933	-1.634914	-5.166173
C	1.401967	2.271981	0.272182	H	-1.170751	-0.105828	-4.358827
C	0.41861	3.1659	-0.206757	H	0.33209	-0.926836	-3.940642
H	-0.633234	2.987233	0.016812				

(*P*)-L1:TS$_3$-(*R*)

$E_{toluene}$	-3079.930705	a.u.
$H_{toluene}$	-3079.074245	a.u.
$G_{toluene}$	-3079.213777	a.u.

Symbol	X	Y	Z	Symbol	X	Y	Z
P	1.111783	-0.988995	-0.624367	C	2.659999	-3.214304	2.453869
C	-0.011533	-2.280957	-1.340704	H	2.412567	-3.350152	3.508733
C	-0.825481	-1.940114	-2.439561	C	3.655052	-3.994634	1.846874
H	-0.762699	-0.946617	-2.878623	H	4.192147	-4.74917	2.426274
C	-1.73493	-2.867145	-2.96245	C	3.962851	-3.800322	0.489308
H	-2.358984	-2.579942	-3.809859	H	4.744	-4.397774	0.013475
C	-1.862627	-4.140126	-2.382211	C	3.266233	-2.842983	-0.261101
H	-2.574688	-4.861567	-2.790146	H	3.508489	-2.694852	-1.313754
C	-1.075077	-4.47656	-1.270367	Pd	-0.128872	0.662841	0.419495
H	-1.170434	-5.460544	-0.805605	C	-0.376781	-1.911468	4.920306
C	-0.14932	-3.55594	-0.754109	C	-1.389826	-2.526167	4.203927
H	0.46411	-3.836035	0.101712	C	-1.929991	-1.934301	3.022523
C	1.995695	-0.357315	-2.137069	C	-1.398208	-0.682826	2.535806
C	2.194994	-1.262743	-3.181168	C	-0.369941	-0.072687	3.323477
H	2.013143	-2.323217	-3.004366	C	0.128543	-0.661695	4.476748
C	2.523448	-0.847634	-4.495832	H	-3.398433	-3.50246	2.688278
H	2.681878	-1.592631	-5.277734	H	0.023037	-2.377991	5.823545
C	2.538317	0.50241	-4.787375	H	-1.802255	-3.483491	4.532732
H	2.681666	0.854529	-5.811843	C	-2.984558	-2.564795	2.311409
C	2.343981	1.473413	-3.761382	C	-1.85741	-0.129274	1.257895
C	2.279316	2.853435	-4.104653	H	0.053528	0.874233	2.996479
H	2.372162	3.134386	-5.15627	H	0.920641	-0.159342	5.035434
C	2.071207	3.810599	-3.12977	C	-2.958419	-0.766984	0.63259
H	1.965016	4.864399	-3.397665	C	-3.498414	-1.975425	1.176676
C	2.062754	3.447549	-1.761341	H	-4.351115	-2.439835	0.686479
C	1.933986	4.515912	-0.690914	P	-3.832663	-0.149401	-0.843321
H	1.428917	5.400413	-1.11044	O	-4.546572	1.185118	-0.247477
H	2.941572	4.838189	-0.364803	O	-5.157512	-1.120046	-1.018264
C	1.163656	3.972518	0.528896	O	-3.096984	0.000502	-2.141201
H	1.146788	4.719815	1.337359	C	-5.20586	2.078466	-1.170145
H	0.11845	3.774713	0.258535	H	-6.163391	1.642075	-1.496963
C	1.861691	2.720334	1.019106	H	-5.378315	3.008947	-0.615149
C	2.019	2.476399	2.40873	H	-4.566221	2.275369	-2.043797
H	1.484309	3.120564	3.11049	C	-5.054316	-2.228302	-1.939216
C	2.819314	1.445677	2.866746	H	-4.329367	-2.974796	-1.576736
H	2.912428	1.242626	3.935975	H	-6.058118	-2.673082	-1.990652
C	3.615318	0.697289	1.95412	H	-4.738776	-1.878452	-2.933036
C	4.584466	-0.236102	2.419746	C	-2.155228	2.345548	2.390865
H	4.631442	-0.444622	3.49108	C	-1.787904	1.903325	1.098421
C	5.471572	-0.839104	1.544323	C	-2.053594	2.781511	-0.006129
H	6.212909	-1.549383	1.913796	C	-2.65221	4.03367	0.253776
C	5.423183	-0.516182	0.164678	C	-3.010997	4.447423	1.541189
H	6.150299	-0.955826	-0.520883	C	-2.752549	3.59134	2.619968
C	4.448981	0.338651	-0.328262	H	-2.010859	1.6927	3.248413
H	4.429161	0.569182	-1.390477	H	-2.822894	4.702733	-0.595029
C	3.4911	0.941733	0.537345	H	-3.484428	5.419555	1.697196
C	2.474924	1.863576	0.074401	H	-3.026619	3.877605	3.638226
C	2.218748	2.102689	-1.371714	C	-1.605723	2.547386	-1.442713
C	2.190678	1.060403	-2.379505	H	-2.283419	3.064669	-2.141904
C	2.261589	-2.062861	0.341085	H	-0.593499	2.953387	-1.603702
C	1.969582	-2.248063	1.705249	H	-1.587636	1.487846	-1.715831
H	1.195117	-1.646128	2.177391				

(*P*)-L1:TS$_4$-(*S*)

$E_{toluene}$ -3079.937940 a.u.
$H_{toluene}$ -3079.082095 a.u.
$G_{toluene}$ -3079.217599 a.u.

Symbol	X	Y	Z	Symbol	X	Y	Z
P	-0.888846	-0.158253	1.283343	C	-2.654282	-3.841877	1.759858
C	0.299371	-0.148689	2.696842	H	-2.555884	-4.825247	1.295441
C	1.332952	0.80829	2.667739	C	-3.503576	-3.662921	2.861117
H	1.361128	1.553785	1.882446	H	-4.059723	-4.510374	3.267774
C	2.362017	0.784264	3.61511	C	-3.649495	-2.386245	3.431478
H	3.15944	1.526371	3.553991	H	-4.320355	-2.238592	4.280788
C	2.379977	-0.209682	4.606767	C	-2.929346	-1.300725	2.916151
H	3.191367	-0.244784	5.337019	H	-3.039146	-0.312189	3.364066
C	1.356607	-1.167901	4.644507	Pd	0.161937	-0.421536	-0.753608
H	1.366093	-1.950889	5.405947	C	3.639558	-3.622597	2.387049
C	0.320782	-1.141925	3.696865	C	4.448364	-2.541588	2.069921
H	-0.453777	-1.906532	3.733131	C	3.979613	-1.48684	1.235511
C	-1.772881	1.466383	1.40661	C	2.668118	-1.574608	0.641515
C	-1.874868	2.04722	2.672627	C	1.850004	-2.661924	1.052301
H	-1.561656	1.47283	3.545552	C	2.311167	-3.66166	1.898565
C	-2.297895	3.386802	2.857308	H	5.752851	-0.24497	1.490933
H	-2.382035	3.797806	3.865108	H	4.009502	-4.415507	3.041039
C	-2.521895	4.178145	1.746476	H	5.454504	-2.458677	2.489229
H	-2.766313	5.237928	1.854578	C	4.754794	-0.305183	1.051591
C	-2.402628	3.646198	0.429565	C	2.18423	-0.536017	-0.271584
C	-2.51022	4.519705	-0.689289	H	0.805798	-2.657609	0.752199
H	-2.7172	5.577299	-0.508798	H	1.634705	-4.461112	2.208707
C	-2.30071	4.043648	-1.969171	C	2.922396	0.691261	-0.239837
H	-2.306715	4.724291	-2.823758	C	4.198946	0.776677	0.406131
C	-2.115874	2.658884	-2.199773	H	4.746856	1.718675	0.36487
C	-1.876374	2.14308	-3.608084	P	2.27137	2.297531	-0.762094
H	-1.479328	2.959771	-4.231673	O	3.588097	2.967017	-1.484614
H	-2.829291	1.811748	-4.063933	O	2.180662	3.195382	0.623953
C	-0.886124	0.95831	-3.565176	O	1.004907	2.393211	-1.563461
H	-0.710743	0.550076	-4.572017	C	3.4672	4.338664	-1.913052
H	0.062917	1.319031	-3.157591	H	4.412414	4.588381	-2.413401
C	-1.502763	-0.112571	-2.682545	H	2.6277	4.453786	-2.617618
C	-1.617104	-1.456216	-3.150238	H	3.318694	5.002993	-1.045234
H	-0.990725	-1.761736	-3.989353	C	0.993856	3.981119	0.909409
C	-2.485937	-2.34579	-2.555026	H	0.36697	4.103304	0.017293
H	-2.55915	-3.375998	-2.911717	H	0.408606	3.479125	1.693287
C	-3.365149	-1.911815	-1.518213	H	1.342189	4.959353	1.273099
C	-4.377131	-2.773497	-1.013494	C	1.257989	-2.695437	-2.023162
H	-4.426973	-3.795016	-1.399084	C	1.674921	-1.336187	-2.044336
C	-5.289097	-2.331614	-0.06722	C	2.29408	-0.863076	-3.241804
H	-6.058073	-3.005875	0.314507	C	2.393066	-1.740383	-4.346629
C	-5.223763	-0.99518	0.394619	C	1.918152	-3.056173	-4.320537
H	-5.953613	-0.635023	1.122171	C	1.351552	-3.539621	-3.134846
C	-4.230336	-0.137344	-0.059399	H	0.817326	-3.11617	-1.126466
H	-4.199885	0.883699	0.312801	H	2.865976	-1.361995	-5.257234
C	-3.256201	-0.568175	-1.002408	H	2.012646	-3.694481	-5.201998
C	-2.215912	0.303209	-1.523016	H	0.983853	-4.5663	-3.064281
C	-2.13756	1.744464	-1.132833	C	2.899109	0.507096	-3.424925
C	-2.118778	2.237744	0.227346	H	3.672453	0.697788	-2.666538
C	-2.058397	-1.478911	1.822751	H	3.371523	0.5872	-4.4167
C	-1.938308	-2.752332	1.239993	H	2.157416	1.309051	-3.337584
H	-1.293842	-2.876199	0.368224				

(*P*)-L1:TS$_5$-(*R*)

$E_{toluene}$	-3079.935940	a.u.
$H_{toluene}$	-3079.080150	a.u.
$G_{toluene}$	-3079.214570	a.u.

Symbol	X	Y	Z	Symbol	X	Y	Z
P	-0.870119	-0.888656	0.78998	C	-2.885825	-3.375231	-1.745074
C	0.100853	-2.090676	1.822038	H	-2.740847	-3.732745	-2.766794
C	1.202828	-1.606941	2.559532	C	-3.953561	-3.856051	-0.972188
H	1.445886	-0.548435	2.540854	H	-4.647583	-4.588419	-1.391343
C	1.982659	-2.473591	3.336501	C	-4.137518	-3.391069	0.342031
H	2.830813	-2.072416	3.895351	H	-4.978586	-3.751191	0.937697
C	1.673975	-3.843527	3.391216	C	-3.249719	-2.454559	0.885601
H	2.278507	-4.521011	3.998449	H	-3.390598	-2.084095	1.90148
C	0.584638	-4.334496	2.656052	Pd	0.360155	0.442786	-0.658554
H	0.335618	-5.397859	2.687973	C	4.223312	1.711249	3.288259
C	-0.19536	-3.4678	1.87194	C	4.950801	0.931166	2.401335
H	-1.032162	-3.86865	1.301821	C	4.360858	0.425032	1.205922
C	-1.529493	0.29522	2.075848	C	3.006272	0.791313	0.87531
C	-1.456829	-0.06172	3.42129	C	2.279483	1.555893	1.832799
H	-1.337645	-1.111184	3.691349	C	2.861972	1.999477	3.01337
C	-1.405537	0.910969	4.456438	H	6.093619	-0.754432	0.60633
H	-1.357378	0.587921	5.4981	H	4.68605	2.074072	4.208806
C	-1.29568	2.248938	4.126281	H	5.984678	0.654882	2.62458
H	-1.124331	3.005736	4.895763	C	5.054773	-0.506123	0.378384
C	-1.390368	2.671161	2.765429	C	2.387404	0.298146	-0.351689
C	-1.18705	4.03451	2.405016	H	1.212595	1.708591	1.673466
H	-0.950169	4.754614	3.191788	H	2.258094	2.551832	3.736173
C	-1.253649	4.428447	1.078018	C	3.035984	-0.802578	-0.985045
H	-1.028901	5.459066	0.793902	C	4.37693	-1.159087	-0.631012
C	-1.682085	3.521083	0.078818	H	4.869993	-1.959663	-1.182111
C	-1.836869	3.95605	-1.366213	P	2.110115	-2.001547	-1.995559
H	-1.15783	4.796992	-1.571175	O	1.674038	-3.223345	-0.99103
H	-2.871837	4.308774	-1.539629	O	3.325057	-2.638827	-2.907761
C	-1.538623	2.785447	-2.329733	O	0.896564	-1.614886	-2.789691
H	-1.76115	3.083861	-3.366192	C	2.627165	-3.781278	-0.060029
H	-0.467743	2.546647	-2.287277	H	2.843947	-3.059757	0.739198
C	-2.417462	1.603362	-1.954232	H	2.14417	-4.666295	0.369446
C	-3.036483	0.813214	-2.956299	H	3.55783	-4.067669	-0.577173
H	-2.721139	0.945633	-3.993766	C	2.945192	-3.648165	-3.869824
C	-4.043234	-0.080378	-2.636675	H	3.865359	-3.924302	-4.402253
H	-4.516287	-0.6889	-3.410415	H	2.52679	-4.532494	-3.361179
C	-4.546827	-0.149174	-1.307677	H	2.203107	-3.245394	-4.575827
C	-5.728276	-0.887275	-1.013708	C	1.546593	3.067786	-0.746508
H	-6.191544	-1.461915	-1.818741	C	1.796674	1.857123	-1.452128
C	-6.296195	-0.853406	0.24875	C	2.267418	1.968741	-2.798499
H	-7.213141	-1.408902	0.456333	C	2.420045	3.25897	-3.357979
C	-5.683029	-0.084845	1.270877	C	2.129342	4.432821	-2.655559
H	-6.142797	-0.031568	2.260241	C	1.687086	4.330338	-1.330309
C	-4.507264	0.611034	1.027981	H	1.187646	3.040431	0.273974
H	-4.071096	1.207519	1.825145	H	2.778175	3.328168	-4.388695
C	-3.890907	0.598436	-0.259429	H	2.258251	5.406943	-3.132838
C	-2.731567	1.395269	-0.596523	H	1.449107	5.220986	-0.743926
C	-1.999444	2.194175	0.420484	C	2.615969	0.804654	-3.692154
C	-1.668214	1.699494	1.733696	H	2.967046	1.165985	-4.671622
C	-2.179699	-1.972116	0.108548	H	3.419012	0.19301	-3.250188
C	-1.995283	-2.430866	-1.2097	H	1.756507	0.140959	-3.84417
H	-1.153262	-2.063214	-1.798808				

(*P*)-L1:TS$_6$-(*S*)

$E_{toluene}$ -3079.941532 a.u.
$H_{toluene}$ -3079.086259 a.u.
$G_{toluene}$ -3079.222710 a.u.

Symbol	X	Y	Z	Symbol	X	Y	Z
P	0.960781	0.505109	-1.111397	C	1.474038	-2.428262	-3.925443
C	-0.177942	1.657679	-2.006169	H	0.98618	-3.378936	-4.151204
C	-0.678006	2.785756	-1.32003	C	2.486072	-1.936039	-4.763441
H	-0.344193	3.001471	-0.306427	H	2.792597	-2.502882	-5.645644
C	-1.596852	3.641814	-1.938952	C	3.108355	-0.712663	-4.463699
H	-1.980647	4.500985	-1.386171	H	3.904055	-0.329612	-5.106968
C	-2.018759	3.394541	-3.256041	C	2.710471	0.021748	-3.337889
H	-2.728422	4.068193	-3.741872	H	3.200946	0.966812	-3.104245
C	-1.522754	2.275938	-3.943369	Pd	-0.162384	-0.635179	0.55179
H	-1.844987	2.073859	-4.967646	C	-1.403243	4.236375	2.418862
C	-0.615775	1.403348	-3.320458	C	-2.567853	3.947724	1.726595
H	-0.250736	0.528723	-3.857583	C	-2.816457	2.646077	1.197515
C	2.245459	1.71146	-0.490068	C	-1.849509	1.594165	1.393904
C	2.513163	2.81643	-1.30111	C	-0.682835	1.930126	2.14866
H	2.125925	2.832313	-2.320285	C	-0.448623	3.209305	2.633588
C	3.179805	3.970549	-0.819127	H	-4.734584	3.175505	0.315966
H	3.379078	4.804098	-1.495154	H	-1.221289	5.244843	2.797428
C	3.47514	4.063227	0.527584	H	-3.315932	4.724709	1.548339
H	3.887817	4.982332	0.950727	C	-3.979421	2.393886	0.421409
C	3.223247	2.968241	1.404137	C	-2.032597	0.269255	0.773328
C	3.442019	3.115245	2.803522	H	0.069785	1.163343	2.324512
H	3.804707	4.075664	3.177244	H	0.477632	3.414064	3.172539
C	3.160747	2.075414	3.671481	C	-3.128924	0.156623	-0.129563
H	3.265161	2.206691	4.751084	C	-4.103932	1.194914	-0.245908
C	2.798654	0.802066	3.167482	H	-4.968846	1.030833	-0.887639
C	2.54144	-0.361805	4.10796	P	-3.333941	-1.201439	-1.302284
H	2.26953	0.020281	5.104792	O	-4.516782	-0.733017	-2.350225
H	3.460281	-0.967261	4.225824	O	-4.167315	-2.325859	-0.462572
C	1.417505	-1.249438	3.534338	O	-2.105103	-1.676963	-2.024233
H	1.227685	-2.119503	4.180804	C	-4.117863	0.130315	-3.439815
H	0.482397	-0.673081	3.491895	H	-4.990906	0.208198	-4.103003
C	1.838991	-1.718502	2.154507	H	-3.840896	1.125549	-3.059605
C	1.653123	-3.065338	1.742389	H	-3.262462	-0.299302	-3.981629
H	1.040863	-3.715825	2.369205	C	-4.508791	-3.55248	-1.147128
C	2.218436	-3.536602	0.570149	H	-4.932206	-4.218648	-0.383736
H	2.04121	-4.56296	0.240752	H	-5.254587	-3.3531	-1.932156
C	3.10395	-2.716101	-0.183566	H	-3.6116	-4.011469	-1.590766
C	3.81413	-3.234983	-1.303727	C	-2.015418	-0.692612	3.281847
H	3.596759	-4.255293	-1.628059	C	-1.765979	-1.21413	1.983901
C	4.777237	-2.478794	-1.949645	C	-1.92285	-2.631668	1.815193
H	5.315049	-2.88782	-2.806507	C	-2.323514	-3.410178	2.924365
C	5.073799	-1.172309	-1.483223	C	-2.575811	-2.866164	4.186433
H	5.861685	-0.590535	-1.965722	C	-2.409125	-1.486312	4.363259
C	4.361984	-0.618164	-0.43089	H	-1.928814	0.375937	3.457991
H	4.602537	0.386354	-0.091102	H	-2.417837	-4.490669	2.777752
C	3.331671	-1.350951	0.227493	H	-2.896218	-3.506181	5.011646
C	2.570405	-0.824536	1.337287	H	-2.598718	-1.018092	5.332054
C	2.682889	0.587502	1.781571	C	-1.550043	-3.445044	0.585211
C	2.716263	1.715727	0.879209	H	-2.325911	-4.199034	0.381189
C	1.690728	-0.464987	-2.498584	H	-1.40081	-2.848026	-0.318603
C	1.080569	-1.700472	-2.791271	H	-0.609075	-3.98363	0.77864
H	0.274069	-2.062394	-2.153806				

(*P*)-L1:TS$_7$-(*R*)

E_{toluene}	-3079.943331	a.u.
H_{toluene}	-3079.087645	a.u.
G_{toluene}	-3079.224171	a.u.

Symbol	X	Y	Z	Symbol	X	Y	Z
P	-0.982903	0.61319	1.067987	C	-1.47421	-1.947808	4.229177
C	0.078755	1.901694	1.875556	H	-0.957887	-2.838299	4.594251
C	0.624811	2.927333	1.073546	C	-2.525579	-1.386245	4.969711
H	0.369326	2.986549	0.017664	H	-2.833834	-1.838293	5.915359
C	1.482916	3.883335	1.630723	C	-3.185178	-0.242332	4.49006
H	1.905323	4.658833	0.98933	H	-4.011245	0.193178	5.057107
C	1.794553	3.842915	3.000471	C	-2.78545	0.345098	3.281689
H	2.458563	4.593099	3.435853	H	-3.30168	1.230842	2.910741
C	1.245731	2.833202	3.805628	Pd	0.208433	-0.674128	-0.453384
H	1.479003	2.793294	4.872293	C	1.51587	3.992842	-2.788768
C	0.399706	1.860994	3.246874	C	2.678953	3.759418	-2.073407
H	-0.010382	1.075183	3.880626	C	2.89405	2.526163	-1.38755
C	-2.286182	1.688265	0.272266	C	1.901874	1.479682	-1.473131
C	-2.598947	2.887554	0.915624	C	0.727952	1.760335	-2.238108
H	-2.233011	3.058125	1.92847	C	0.524097	2.979068	-2.865866
C	-3.290943	3.940795	0.266937	H	4.80091	3.123733	-0.527196
H	-3.525174	4.854715	0.815962	H	1.359267	4.952367	-3.286926
C	-3.568258	3.833307	-1.082494	H	3.446456	4.533803	-1.99283
H	-4.001263	4.670344	-1.635693	C	4.036653	2.344333	-0.562548
C	-3.270603	2.634001	-1.792638	C	2.053872	0.247183	-0.703997
C	-3.468084	2.575492	-3.201839	H	-0.046678	0.995511	-2.302958
H	-3.854312	3.461038	-3.712148	H	-0.404007	3.152039	-3.413267
C	-3.138105	1.434264	-3.911091	C	3.138406	0.197763	0.207612
H	-3.225759	1.410171	-4.999842	C	4.131212	1.223491	0.23706
C	-2.743242	0.257924	-3.228074	H	4.975345	1.117347	0.91867
C	-2.41567	-1.01523	-3.987757	P	3.323424	-1.102649	1.455309
H	-2.149706	-0.768708	-5.027941	O	4.299942	-0.461431	2.614299
H	-3.298554	-1.681506	-4.020696	O	4.372121	-2.146062	0.769136
C	-1.253347	-1.742721	-3.280175	O	2.071274	-1.708135	2.023282
H	-0.98975	-2.678553	-3.794905	C	3.687697	0.506997	3.498764
H	-0.363271	-1.101239	-3.301363	H	3.406484	1.41342	2.94179
C	-1.675477	-2.040681	-1.852021	H	2.793034	0.083348	3.979436
C	-1.421592	-3.302328	-1.245659	H	4.445402	0.751001	4.256391
H	-0.735215	-3.986311	-1.746051	C	4.492434	-3.464427	1.345614
C	-2.006772	-3.637978	-0.037579	H	5.135527	-4.034337	0.662482
H	-1.784184	-4.594603	0.440661	H	4.956536	-3.40834	2.343475
C	-2.97257	-2.779379	0.561697	H	3.50496	-3.944631	1.42134
C	-3.704259	-3.182724	1.714584	C	1.899896	-2.773439	-0.964428
H	-3.450108	-4.136434	2.182806	C	1.79026	-1.542473	-1.666925
C	-4.730926	-2.401119	2.217396	C	2.146895	-1.531017	-3.050119
H	-5.285169	-2.72331	3.10052	C	2.533169	-2.750494	-3.653215
C	-5.069094	-1.183648	1.572663	C	2.576696	-3.964961	-2.959223
H	-5.902666	-0.58559	1.946389	C	2.25673	-3.971697	-1.596565
C	-4.339829	-0.735465	0.482234	H	1.672174	-2.815172	0.098941
H	-4.61026	0.203582	0.00427	H	2.810609	-2.733646	-4.710882
C	-3.252858	-1.496611	-0.037133	H	2.874088	-4.881714	-3.473634
C	-2.480109	-1.086173	-1.186464	H	2.286825	-4.897507	-1.016203
C	-2.645549	0.244122	-1.824451	C	2.183412	-0.310659	-3.950838
C	-2.735681	1.484749	-1.089468	H	2.533269	-0.596992	-4.955074
C	-1.726706	-0.211103	2.539451	H	1.201339	0.170966	-4.065627
C	-1.076871	-1.36821	3.013622	H	2.862299	0.461044	-3.559605
H	-0.241961	-1.786251	2.450662				

(*P*)-L2:TS$_1$-(*R*)

$E_{toluene}$	-3078.728448	a.u.
$H_{toluene}$	-3077.896063	a.u.
$G_{toluene}$	-3078.030789	a.u.

Symbol	X	Y	Z	Symbol	X	Y	Z
P	-1.648867	-0.322305	-0.198893	C	-5.078725	2.756535	0.451163
C	-1.173291	0.328522	-1.864956	H	-5.845497	3.528412	0.546536
C	-0.064574	-0.277338	-2.488038	C	-5.317361	1.620284	-0.341131
H	0.412798	-1.13805	-2.031336	H	-6.272251	1.50373	-0.858646
C	0.464166	0.245347	-3.673314	C	-4.329128	0.635857	-0.476301
H	1.336539	-0.231685	-4.12291	H	-4.517154	-0.240218	-1.097567
C	-0.105144	1.392099	-4.251061	Pd	0.178308	-0.323808	1.222284
H	0.3174	1.815848	-5.164758	C	1.63978	4.30526	-1.643214
C	-1.210882	2.000323	-3.639114	C	2.686277	3.448889	-1.95008
H	-1.655936	2.896732	-4.076926	C	2.760649	2.141667	-1.388303
C	-1.745422	1.474975	-2.451402	C	1.790937	1.731612	-0.398485
H	-2.590819	1.973644	-1.979457	C	0.705714	2.617836	-0.164817
C	-2.320574	-2.019272	-0.560835	C	0.617071	3.86551	-0.768102
C	-2.893503	-2.220393	-1.824045	H	4.455278	1.511209	-2.605072
H	-3.063639	-1.359757	-2.471971	H	1.582904	5.294115	-2.104002
C	-3.191435	-3.509348	-2.317923	H	3.44994	3.741939	-2.67524
H	-3.66782	-3.623369	-3.293519	C	3.709653	1.197575	-1.871334
C	-2.786064	-4.619088	-1.591276	C	1.863363	0.404703	0.223015
H	-2.902539	-5.626506	-1.998457	H	-0.101024	2.27084	0.471316
C	-2.149765	-4.470318	-0.330798	H	-0.254962	4.495058	-0.578603
C	-1.52953	-5.608146	0.285806	C	2.671042	-0.547275	-0.489571
H	-1.637298	-6.582431	-0.196379	C	3.594104	-0.124377	-1.495618
C	-0.719789	-5.445483	1.381636	H	4.225445	-0.87442	-1.974484
H	-0.133416	-6.278347	1.775717	P	2.505955	-2.354658	-0.384837
C	-0.645995	-4.183776	2.055029	O	4.025889	-2.940762	-0.65066
C	0.263284	-3.998837	3.142959	O	1.850002	-2.754353	-1.829231
H	0.933605	-4.820939	3.399799	O	1.813067	-2.96005	0.798901
C	0.367276	-2.780626	3.769297	C	5.013674	-2.658996	0.361679
H	1.143234	-2.588109	4.511489	H	5.134269	-1.571098	0.498738
C	-0.610576	-1.767473	3.525463	H	4.731388	-3.122337	1.321645
C	-0.645487	-0.577807	4.333571	H	5.955874	-3.095155	0.002783
H	0.217847	-0.364106	4.965324	C	0.901714	-3.848718	-1.916005
C	-1.734584	0.256659	4.311141	H	1.38343	-4.683147	-2.448537
H	-1.75709	1.160324	4.924392	H	0.572633	-4.174527	-0.921162
C	-2.903538	-0.09295	3.556808	H	0.037978	-3.480664	-2.487436
C	-4.108745	0.640789	3.707286	C	2.98554	-0.640296	2.53868
H	-4.103735	1.51928	4.357102	C	2.067563	0.361511	2.14062
C	-5.274571	0.246098	3.064842	C	1.887855	1.466179	3.037194
H	-6.193247	0.822077	3.187993	C	2.657643	1.51571	4.221671
C	-5.266903	-0.916806	2.261711	C	3.566733	0.515251	4.580647
H	-6.187822	-1.254195	1.782485	C	3.712724	-0.589047	3.730238
C	-4.092507	-1.635336	2.07022	H	3.112105	-1.512544	1.913558
H	-4.110656	-2.531083	1.454075	H	2.509402	2.366893	4.892122
C	-2.873496	-1.230828	2.676002	H	4.139106	0.595447	5.507482
C	-1.633425	-1.986748	2.538143	H	4.39247	-1.406891	3.982447
C	-1.465068	-3.098345	1.617173	C	0.868608	2.577769	2.874792
C	-2.022461	-3.162636	0.273752	H	0.734977	3.108485	3.831063
C	-3.090628	0.772287	0.181967	H	1.178381	3.313807	2.119448
C	-2.874722	1.894394	1.000129	H	-0.106519	2.172314	2.577092
H	-1.933739	1.978423	1.542503				
C	-3.858166	2.887598	1.128503				
H	-3.672358	3.754214	1.766661				

(*P*)-L2:TS$_3$-(*R*)

$E_{toluene}$	-3078.725790	a.u.
$H_{toluene}$	-3077.893053	a.u.
$G_{toluene}$	-3078.026505	a.u.

Symbol	X	Y	Z	Symbol	X	Y	Z
P	-1.107586	1.047368	-0.605089	C	-3.924964	3.71551	0.920217
C	-0.053441	2.408342	-1.297441	H	-4.728273	4.353763	0.54473
C	0.567826	2.252791	-2.553005	C	-3.246994	2.863764	0.038066
H	0.404026	1.345827	-3.131803	H	-3.533663	2.839732	-1.012836
C	1.404468	3.255431	-3.05898	Pd	0.163953	-0.661155	0.305195
H	1.869957	3.120607	-4.037341	C	0.625926	1.529761	5.113192
C	1.661251	4.412338	-2.305198	C	1.689003	2.116645	4.447798
H	2.316809	5.191979	-2.700345	C	2.152388	1.61332	3.194663
C	1.077816	4.555927	-1.036427	C	1.495645	0.480873	2.585643
H	1.275911	5.447199	-0.436709	C	0.414725	-0.101958	3.319131
C	0.219278	3.56421	-0.536687	C	-0.012207	0.399273	4.540553
H	-0.244009	3.698219	0.441093	H	3.767828	3.057292	3.006055
C	-2.111999	0.531981	-2.089424	H	0.285573	1.928599	6.071522
C	-2.445801	1.526931	-3.017452	H	2.19951	2.985208	4.871741
H	-2.250093	2.569871	-2.769463	C	3.250943	2.220531	2.531303
C	-2.936458	1.223133	-4.30609	C	1.894916	0.00794	1.254611
H	-3.210452	2.028728	-4.989848	H	-0.106699	-0.955123	2.891617
C	-2.958653	-0.099394	-4.720296	H	-0.851701	-0.076732	5.051703
H	-3.214591	-0.359319	-5.750229	C	2.998381	0.660874	0.647412
C	-2.596446	-1.145033	-3.829786	C	3.662122	1.743456	1.305386
C	-2.4349	-2.478427	-4.334898	H	4.527698	2.197748	0.827404
H	-2.640306	-2.660326	-5.39214	P	3.667874	0.250773	-0.991942
C	-1.940271	-3.470962	-3.528641	O	4.450725	-1.145294	-0.689665
H	-1.706496	-4.459157	-3.931182	O	4.961142	1.252195	-1.219895
C	-1.77591	-3.254743	-2.121653	O	2.746126	0.273053	-2.173408
C	-1.256077	-4.299136	-1.295511	C	5.143056	-1.793073	-1.778149
C	-1.110029	-4.11509	0.059626	H	5.286774	-2.836525	-1.46761
C	-1.700033	-2.979893	0.691284	H	4.545374	-1.757473	-2.702283
C	-1.730532	-2.8959	2.123442	H	6.116067	-1.304729	-1.943587
H	-1.146016	-3.622956	2.690013	C	4.706057	2.502288	-1.901048
C	-2.467053	-1.928526	2.758065	H	5.687798	2.976071	-2.041831
H	-2.475728	-1.857966	3.848154	H	4.220979	2.32274	-2.870953
C	-3.31553	-1.049948	2.007265	H	4.056321	3.15427	-1.295227
C	-4.231043	-0.19373	2.673029	C	2.017891	-2.528718	2.296892
H	-4.192869	-0.141215	3.763636	C	1.77531	-1.997532	1.006296
C	-5.176772	0.533703	1.963788	C	2.027284	-2.854428	-0.115159
H	-5.873766	1.190125	2.486776	C	2.475687	-4.173029	0.126965
C	-5.24368	0.398693	0.559005	C	2.697438	-4.677815	1.41065
H	-6.016552	0.926218	-0.002792	C	2.463724	-3.838449	2.508298
C	-4.324314	-0.391914	-0.12104	H	1.887981	-1.904133	3.175857
H	-4.402658	-0.483785	-1.201007	H	2.633491	-4.820765	-0.741122
C	-3.30261	-1.096556	0.568985	H	3.052381	-5.701698	1.550132
C	-2.333442	-1.960203	-0.103095	H	2.64194	-4.186953	3.528504
C	-2.138414	-1.993905	-1.543308	C	1.750331	-2.546084	-1.57797
C	-2.306585	-0.855502	-2.443378	H	0.897325	-3.148898	-1.920043
C	-2.212917	2.029711	0.507583	H	1.525549	-1.494642	-1.775845
C	-1.877619	2.059292	1.872503	H	2.619021	-2.825835	-2.194857
H	-1.083367	1.41536	2.2424	H	-0.610242	-4.859247	0.680797
C	-2.548168	2.921262	2.754944	H	-0.922584	-5.221809	-1.775398
H	-2.264358	2.932098	3.809325				
C	-3.571741	3.753317	2.280577				
H	-4.096076	4.426048	2.96323				

(*P*)-L2:TS$_4$-(*S*)

$E_{toluene}$	-3078.728458	a.u.
$H_{toluene}$	-3077.897289	a.u.
$G_{toluene}$	-3078.030365	a.u.

Symbol	X	Y	Z	Symbol	X	Y	Z
P	-1.031246	0.046691	1.192278	C	-4.166509	-1.948679	3.05366
C	-0.032979	0.154235	2.755646	H	-5.003821	-1.705729	3.711723
C	1.261775	0.696141	2.63883	C	-3.373	-0.917602	2.528515
H	1.619558	1.030091	1.671732	H	-3.590033	0.121904	2.777867
C	2.116419	0.763332	3.745756	Pd	0.28756	-0.527204	-0.629204
H	3.118858	1.17623	3.620157	C	3.51461	-3.623763	2.847308
C	1.691157	0.273716	4.990997	C	4.446874	-2.745218	2.315593
H	2.360053	0.308138	5.85383	C	4.072149	-1.767356	1.349405
C	0.406629	-0.278276	5.117335	C	2.720243	-1.745216	0.845262
H	0.071201	-0.672306	6.079516	C	1.786262	-2.62335	1.460282
C	-0.452606	-0.339718	4.008215	C	2.16036	-3.534414	2.439267
H	-1.438795	-0.78907	4.121131	H	6.01493	-0.777551	1.307851
C	-1.867912	1.71072	1.099661	H	3.813274	-4.352081	3.604619
C	-2.077939	2.393642	2.304774	H	5.482456	-2.755107	2.665414
H	-1.949543	1.861276	3.248279	C	4.983457	-0.746474	0.949949
C	-2.364232	3.777236	2.342499	C	2.316926	-0.774946	-0.16995
H	-2.54807	4.269448	3.299481	H	0.736935	-2.506542	1.201916
C	-2.304194	4.511821	1.16724	H	1.40222	-4.164153	2.909718
H	-2.402669	5.600161	1.183232	C	3.193304	0.340662	-0.326434
C	-2.058794	3.873274	-0.077672	C	4.520113	0.322869	0.214484
C	-1.795286	4.661766	-1.247666	H	5.174474	1.174028	0.023432
H	-1.856219	5.749432	-1.164006	P	2.624902	1.975486	-0.854185
C	-1.373407	4.063011	-2.408827	O	3.938821	2.545832	-1.659401
H	-1.049317	4.661648	-3.263167	O	2.669885	2.867574	0.538229
C	-1.379476	2.637608	-2.539979	O	1.319139	2.142535	-1.571414
C	-0.859047	2.009299	-3.71449	C	3.887576	3.924237	-2.085094
C	-0.809347	0.638121	-3.811946	H	3.008015	4.100017	-2.725303
C	-1.514697	-0.172962	-2.869103	H	3.85367	4.59526	-1.210895
C	-1.641279	-1.591586	-3.064942	H	4.808362	4.103892	-2.655998
H	-0.995035	-2.068166	-3.803549	C	1.495752	3.645505	0.887747
C	-2.547676	-2.321459	-2.337151	H	1.730708	4.111424	1.854031
H	-2.635723	-3.400659	-2.481177	H	1.294537	4.415911	0.128655
C	-3.461811	-1.668116	-1.445905	H	0.609062	3.005871	0.987091
C	-4.55371	-2.374933	-0.876376	C	1.196042	-3.035001	-1.614729
H	-4.635515	-3.447095	-1.069185	C	1.617185	-1.694613	-1.82343
C	-5.511555	-1.720644	-0.114947	C	2.10022	-1.34382	-3.120297
H	-6.347651	-2.27676	0.312692	C	2.076666	-2.324612	-4.136977
C	-5.408819	-0.323658	0.087449	C	1.605937	-3.626329	-3.924328
H	-6.183269	0.204173	0.647675	C	1.167544	-3.984635	-2.643065
C	-4.325374	0.383301	-0.416854	H	0.876129	-3.353471	-0.628299
H	-4.266045	1.457458	-0.257133	H	2.438679	-2.040477	-5.129167
C	-3.304818	-0.26688	-1.163074	H	1.602048	-4.350572	-4.74217
C	-2.192333	0.452355	-1.767712	H	0.812261	-4.996664	-2.434265
C	-1.873387	1.833891	-1.468247	C	2.62486	0.018346	-3.491637
C	-1.978447	2.432963	-0.14973	H	3.52084	0.266294	-2.900843
C	-2.288525	-1.214683	1.681626	H	2.906326	0.045693	-4.556554
C	-2.04297	-2.555948	1.329493	H	1.881084	0.80082	-3.300217
H	-1.244585	-2.777649	0.619272	H	-0.300691	0.145957	-4.642106
C	-2.830161	-3.587978	1.859951	H	-0.420233	2.641888	-4.488627
H	-2.625398	-4.623919	1.5808				
C	-3.886234	-3.286925	2.734123				
H	-4.499508	-4.089004	3.150613				

(*P*)-L2:TS$_5$-(*R*)

$E_{toluene}$	-3078.730872	a.u.
$H_{toluene}$	-3077.898721	a.u.
$G_{toluene}$	-3078.032223	a.u.

Symbol	X	Y	Z	Symbol	X	Y	Z
P	-0.908802	-0.75795	1.069815	C	-3.43988	-4.045589	1.372453
C	0.360754	-1.517859	2.189151	H	-4.128291	-4.473249	2.105187
C	1.02861	-0.732147	3.150738	C	-2.830041	-2.811341	1.631598
H	0.717173	0.295457	3.320513	H	-3.054363	-2.285822	2.559291
C	2.090847	-1.258547	3.896756	Pd	0.030162	0.594261	-0.611417
H	2.607479	-0.620647	4.616136	C	4.173057	1.973546	2.944472
C	2.491961	-2.590197	3.707307	C	4.852646	1.19377	2.019138
H	3.318059	-3.004754	4.289315	C	4.191072	0.653289	0.878941
C	1.820638	-3.38562	2.765704	C	2.799444	0.951529	0.65672
H	2.116332	-4.426791	2.618695	C	2.129933	1.715625	1.651922
C	0.767603	-2.855328	2.004338	C	2.790126	2.220622	2.766504
H	0.271092	-3.478849	1.262811	H	5.936753	-0.408623	0.112818
C	-1.871326	0.32391	2.244958	H	4.695572	2.374571	3.816015
C	-2.011311	-0.109678	3.56901	H	5.910489	0.958468	2.162277
H	-1.699768	-1.120671	3.830872	C	4.867641	-0.22735	-0.016278
C	-2.448232	0.751631	4.600476	C	2.102353	0.448985	-0.536424
H	-2.571086	0.367971	5.615037	H	1.051257	1.852422	1.555184
C	-2.612304	2.101243	4.327226	H	2.231422	2.78692	3.515068
H	-2.831414	2.811645	5.128142	C	2.751465	-0.650967	-1.19428
C	-2.446653	2.5997	3.007444	C	4.140643	-0.915594	-0.965233
C	-2.417574	4.015744	2.774605	H	4.632046	-1.682914	-1.564031
H	-2.578705	4.684415	3.623249	P	1.849024	-1.970511	-2.037339
C	-2.098151	4.509918	1.534418	O	1.78968	-3.258268	-1.010303
H	-1.963586	5.582258	1.374887	O	2.940741	-2.442653	-3.179652
C	-1.985533	3.635936	0.40378	O	0.456746	-1.770243	-2.557424
C	-1.624408	4.152134	-0.879929	C	2.97981	-3.78143	-0.390733
C	-1.497729	3.317463	-1.964134	H	3.753254	-3.995265	-1.146811
C	-1.938425	1.960279	-1.885456	H	3.368975	-3.075985	0.357095
C	-1.974033	1.133112	-3.062876	H	2.677868	-4.714137	0.104576
H	-1.46086	1.494358	-3.955083	C	2.529023	-3.514401	-4.057177
C	-2.630211	-0.070234	-3.056652	H	1.597312	-3.246841	-4.578667
H	-2.628516	-0.711826	-3.939584	H	3.344372	-3.647897	-4.780853
C	-3.4083	-0.469275	-1.919148	H	2.375845	-4.44655	-3.488206
C	-4.271359	-1.592863	-1.99421	C	1.527645	3.186178	-0.931697
H	-4.254435	-2.199964	-2.902033	C	1.480367	1.926971	-1.592151
C	-5.135875	-1.903797	-0.953071	C	1.626887	1.946699	-3.018152
H	-5.791449	-2.773156	-1.02279	C	1.804581	3.183999	-3.675467
C	-5.171997	-1.07625	0.19133	C	1.847336	4.406034	-2.995627
H	-5.879422	-1.288086	0.995255	C	1.705116	4.398438	-1.602801
C	-4.302886	0.002921	0.310945	H	1.424034	3.233202	0.145088
H	-4.350532	0.628596	1.198922	H	1.909925	3.171563	-4.76387
C	-3.36974	0.311761	-0.71269	H	1.99328	5.339576	-3.543865
C	-2.454797	1.449301	-0.640635	H	1.723754	5.328817	-1.030285
C	-2.23043	2.236158	0.563992	C	1.596636	0.721082	-3.889282
C	-2.215538	1.686498	1.912473	H	1.593915	1.004175	-4.954217
C	-1.943318	-2.245135	0.69384	H	2.491082	0.099844	-3.713714
C	-1.688239	-2.920896	-0.513635	H	0.723411	0.094018	-3.679765
H	-1.008502	-2.486296	-1.246781	H	-1.09091	3.681666	-2.907707
C	-2.290981	-4.165761	-0.762253	H	-1.377312	5.212068	-0.962556
H	-2.075382	-4.684989	-1.698726				
C	-3.162583	-4.732476	0.17764				
H	-3.629674	-5.700824	-0.017396				

(*P*)-L2:TS$_6$-(*S*)

$E_{toluene}$	-3078.734510	a.u.
$H_{toluene}$	-3077.902306	a.u.
$G_{toluene}$	-3078.037146	a.u.

Symbol	X	Y	Z	Symbol	X	Y	Z
P	1.005472	1.104465	0.51488	C	3.279963	4.368454	-0.708907
C	-0.118469	2.064665	1.628904	H	4.085346	4.993892	-0.316664
C	-0.743039	1.380448	2.694224	C	2.834692	3.263782	0.030715
H	-0.506905	0.334823	2.882528	H	3.297176	3.030608	0.989882
C	-1.665091	2.039074	3.516744	Pd	-0.149902	-0.559623	-0.605848
H	-2.146923	1.487406	4.325793	C	-1.740572	-2.310063	4.238141
C	-1.967103	3.393997	3.296472	C	-2.879779	-1.624358	3.850013
H	-2.682573	3.909569	3.941268	C	-3.033182	-1.130012	2.520291
C	-1.34418	4.080949	2.242895	C	-1.996313	-1.35745	1.544001
H	-1.571884	5.134559	2.064285	C	-0.857409	-2.101054	1.9821
C	-0.430037	3.420116	1.405973	C	-0.716073	-2.551121	3.287292
H	0.037049	3.959135	0.582276	H	-4.984358	-0.234949	2.884322
C	2.253478	0.464598	1.75262	H	-1.631374	-2.662095	5.266529
C	2.538401	1.281756	2.854006	H	-3.680342	-1.423932	4.567007
H	2.186189	2.313825	2.854591	C	-4.172024	-0.358618	2.165062
C	3.185422	0.78864	4.009455	C	-2.078742	-0.771398	0.195024
H	3.415348	1.46495	4.834898	H	-0.05962	-2.300398	1.271012
C	3.415778	-0.574334	4.125134	H	0.192365	-3.084379	3.570899
H	3.793394	-1.001068	5.05749	C	-3.149757	0.146059	-0.012338
C	3.10891	-1.452723	3.052469	C	-4.200575	0.285624	0.947925
C	3.143083	-2.874188	3.252553	H	-5.041701	0.936113	0.710297
H	3.473085	-3.254013	4.222175	P	-3.225178	1.35165	-1.358438
C	2.663783	-3.723928	2.28586	O	-4.342553	2.472376	-0.89971
H	2.565057	-4.795007	2.476742	O	-4.082738	0.60023	-2.526648
C	2.327836	-3.231363	0.98161	O	-1.936532	1.996338	-1.782565
C	1.764488	-4.096745	-0.008524	C	-3.89887	3.4821	0.037506
C	1.441256	-3.615489	-1.256664	H	-4.718371	4.210686	0.111651
C	1.87683	-2.314675	-1.659065	H	-3.703249	3.032481	1.023137
C	1.698544	-1.871623	-3.012849	H	-2.983031	3.971491	-0.324274
H	1.092717	-2.492689	-3.674164	C	-4.31309	1.331033	-3.753286
C	2.261718	-0.699695	-3.454096	H	-3.360577	1.686176	-4.176328
H	2.100279	-0.355066	-4.477906	H	-4.790377	0.623245	-4.443989
C	3.148787	0.04625	-2.610813	H	-4.981457	2.185834	-3.566344
C	3.892488	1.139939	-3.128436	C	-1.847189	-3.323936	-0.692384
H	3.697909	1.460948	-4.154215	C	-1.678699	-2.020029	-1.230491
C	4.866999	1.766698	-2.365361	C	-1.785466	-1.8906	-2.656334
H	5.43208	2.605267	-2.775069	C	-2.073865	-3.040547	-3.426168
C	5.137433	1.297479	-1.058426	C	-2.252772	-4.308033	-2.8661
H	5.936231	1.753634	-0.47053	C	-2.12655	-4.445885	-1.477179
C	4.38751	0.265467	-0.509165	H	-1.784339	-3.4766	0.380214
H	4.615312	-0.083384	0.49519	H	-2.134999	-2.920582	-4.512366
C	3.344229	-0.359261	-1.244817	H	-2.484606	-5.166677	-3.500385
C	2.552085	-1.463852	-0.718456	H	-2.255264	-5.418359	-0.996026
C	2.533939	-1.848692	0.677196	C	-1.466947	-0.656271	-3.485423
C	2.663093	-0.918736	1.787562	H	-2.277974	-0.461124	-4.20363
C	1.800754	2.447498	-0.465965	H	-1.299105	0.247367	-2.894386
C	1.225405	2.741425	-1.717733	H	-0.548483	-0.842003	-4.064964
H	0.407528	2.123951	-2.088075	H	0.896838	-4.228776	-1.975379
C	1.667329	3.853448	-2.4516	H	1.538244	-5.128301	0.269652
H	1.205845	4.080499	-3.415084				
C	2.692662	4.669176	-1.949097				
H	3.035649	5.535002	-2.520318				

(***P***)-L2:TS$_7$-(***R***)

E_{toluene}	-3078.737518	a.u.
H_{toluene}	-3077.903671	a.u.
G_{toluene}	-3078.040476	a.u.

Symbol	X	Y	Z
P	-1.053524	0.657949	1.014879
C	-0.038036	2.003823	1.786377
C	0.539915	2.975452	0.940899
H	0.324502	2.965019	-0.125553
C	1.386874	3.959537	1.465488
H	1.835203	4.691489	0.791399
C	1.659028	3.997487	2.843883
H	2.317612	4.766903	3.253211
C	1.078527	3.041477	3.691505
H	1.28079	3.064196	4.765012
C	0.239173	2.044446	3.167337
H	-0.197027	1.301067	3.834316
C	-2.351653	1.662049	0.12319
C	-2.733937	2.880419	0.698375
H	-2.42589	3.104881	1.720031
C	-3.425434	3.872116	-0.033482
H	-3.730873	4.797655	0.45816
C	-3.603545	3.703112	-1.398917
H	-4.014194	4.50806	-2.013306
C	-3.201912	2.499718	-2.037067
C	-3.187319	2.411327	-3.470335
H	-3.550263	3.265569	-4.046519
C	-2.628568	1.322878	-4.094989
H	-2.498496	1.302669	-5.179425
C	-2.248243	0.163237	-3.341545
C	-1.613946	-0.951976	-3.974851
C	-1.234277	-2.0524	-3.239353
C	-1.676007	-2.203284	-1.887267
C	-1.411469	-3.412127	-1.156791
H	-0.726199	-4.135845	-1.598926
C	-1.983126	-3.631356	0.072153
H	-1.758482	-4.539206	0.636421
C	-2.96408	-2.724856	0.594179
C	-3.721059	-3.058173	1.748549
H	-3.470769	-3.97448	2.287634
C	-4.77657	-2.260153	2.16605
H	-5.352482	-2.529981	3.052611
C	-5.11465	-1.104192	1.423442
H	-5.973704	-0.498787	1.71913
C	-4.354625	-0.724508	0.324518
H	-4.632138	0.165848	-0.235333
C	-3.237338	-1.494603	-0.097671
C	-2.439005	-1.155216	-1.268388
C	-2.486666	0.131658	-1.931512
C	-2.709721	1.395798	-1.248663
C	-1.826949	-0.106593	2.503803
C	-1.166034	-1.221351	3.057811
H	-0.303688	-1.646003	2.543078
C	-1.591355	-1.749828	4.286927
H	-1.066491	-2.607197	4.713969
C	-2.681692	-1.179686	4.962383
H	-3.011502	-1.591875	5.918898
C	-3.351428	-0.078851	4.403426
H	-4.207222	0.3628	4.919314
C	-2.923866	0.45808	3.180669
H	-3.44883	1.30994	2.748074
Pd	0.213711	-0.669891	-0.410977
C	1.662839	3.888174	-2.904802
C	2.805515	3.660236	-2.155643
C	2.982502	2.448028	-1.42284
C	1.975354	1.41485	-1.496835
C	0.823701	1.690358	-2.294948
C	0.655158	2.889296	-2.968686
H	4.877483	3.043804	-0.535296
H	1.534709	4.832018	-3.43984
H	3.584794	4.423626	-2.083848
C	4.099931	2.277239	-0.562335
C	2.085834	0.207765	-0.68243
H	0.045086	0.93232	-2.35595
H	-0.256965	3.055424	-3.544089
C	3.141504	0.176982	0.26318
C	4.151856	1.186918	0.281398
H	4.973403	1.095306	0.992128
P	3.266973	-1.057581	1.583493
O	4.191307	-0.356247	2.749455
O	4.339604	-2.135875	0.994862
O	1.992638	-1.635536	2.130646
C	3.539588	0.655412	3.555632
H	3.285068	1.533627	2.94282
H	2.623495	0.255198	4.015524
H	4.26244	0.937415	4.333718
C	4.447973	-3.415947	1.654887
H	5.091893	-4.032194	1.014021
H	4.906697	-3.298392	2.649848
H	3.456981	-3.884662	1.756172
C	1.882473	-2.803868	-0.812208
C	1.764061	-1.610961	-1.575394
C	2.044695	-1.682273	-2.974042
C	2.380375	-2.940633	-3.525724
C	2.440464	-4.11636	-2.767896
C	2.188383	-4.041851	-1.393377
H	1.695411	-2.786594	0.259048
H	2.598981	-2.987039	-4.596332
H	2.697291	-5.065386	-3.244246
H	2.231792	-4.934626	-0.764225
C	2.030451	-0.519468	-3.944008
H	2.352979	-0.85847	-4.941309
H	1.028158	-0.081248	-4.045926
H	2.70377	0.287929	-3.623351
H	-0.637616	-2.849554	-3.683394
H	-1.380046	-0.882523	-5.039262

14. Chiral HPLC chromatograms

14-1. Asymmetric allylic alkylation of 1,3-diphenylallyl acetate 11 with dimethyl malonate.

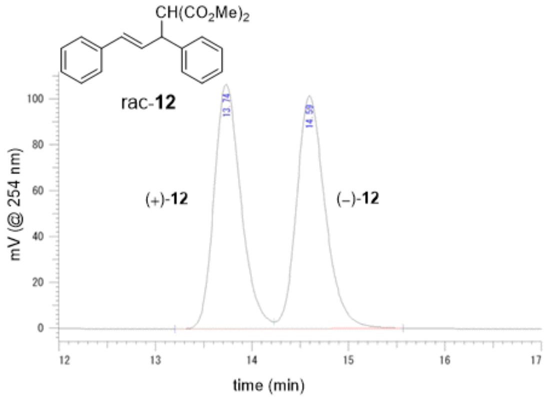

	RT	Area	Ratio
(+)-**12**	13.74	2077382	50
(–)-**12**	14.59	2078295	50

12 (using (*M*)-**L1** as a chiral ligand)

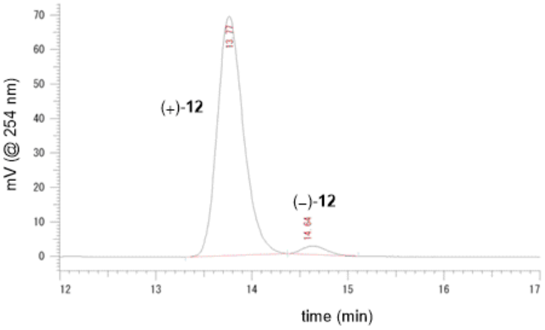

	RT	Area	Ratio
(+)-**12**	13.77	1305038	97
(–)-**12**	14.64	42749	3

14-2. Asymmetric allylation of indoles.

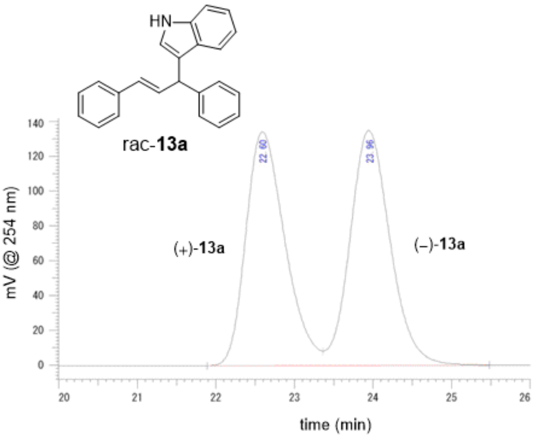

	RT	Area	Ratio
(+)-**13a**	22.60	4787480	50
(–)-**13a**	23.96	4694939	50

13a (using (*M*)-**L1** as a chiral ligand)

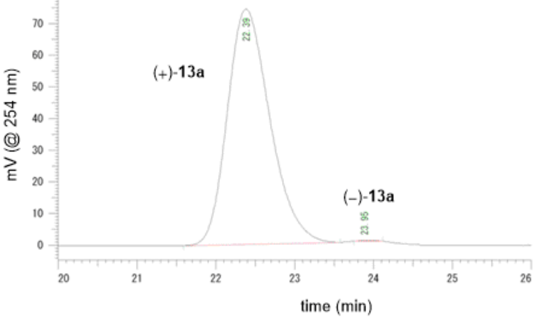

	RT	Area	Ratio
(+)-**13a**	22.39	2775373	99.8
(–)-**13a**	23.95	4574	0.2

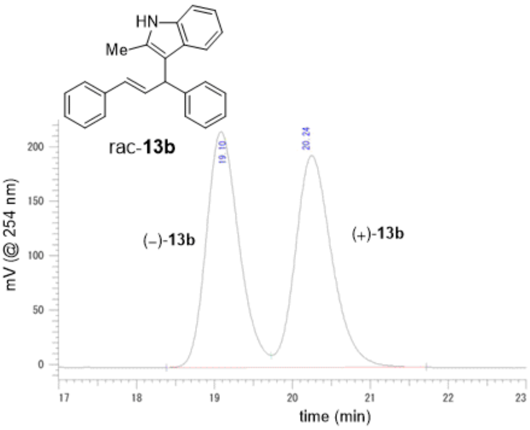

	RT	Area	Ratio
(–)-**13b**	19.10	6384294	50
(+)-**13b**	20.24	6319892	50

13b (using (*M*)-**L1** as a chiral ligand)

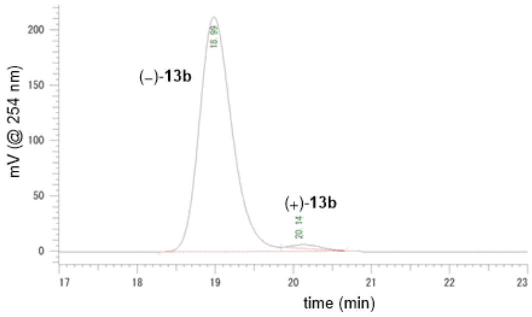

	RT	Area	Ratio
(–)-**13b**	18.99	6246331	98.7
(+)-**13b**	20.14	83034	1.3

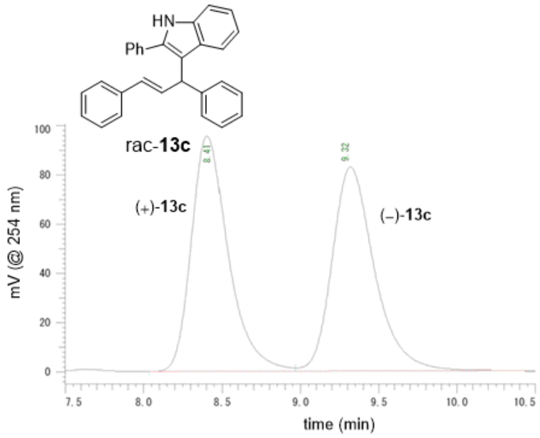

13c (using (*M*)-**L1** as a chiral ligand)

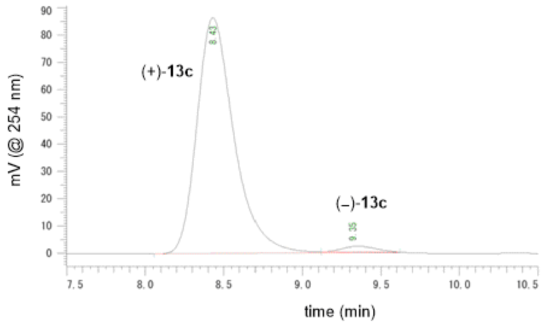

	RT	Area	Ratio
(+)-**13c**	8.41	1546340	50
(–)-**13c**	9.32	1561456	50

	RT	Area	Ratio
(+)-**13c**	8.43	1418032	97.8
(–)-**13c**	9.35	31740	2.2

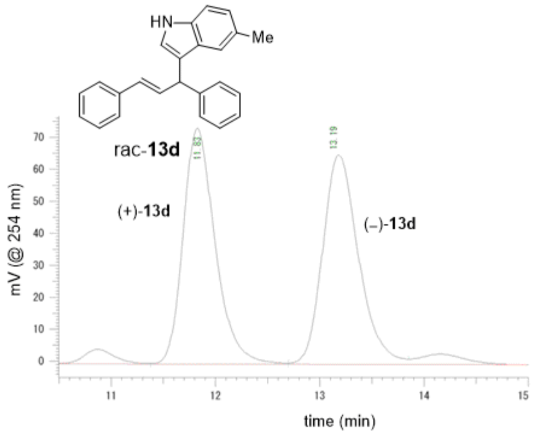

13d (using (*M*)-**L1** as a chiral ligand)

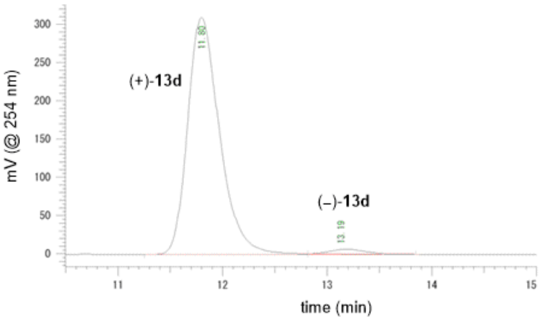

	RT	Area	Ratio
(+)-**13d**	11.83	1571146	50
(–)-**13d**	13.19	1563354	50

	RT	Area	Ratio
(+)-**13d**	11.80	6590233	97.9
(–)-**13d**	13.19	140476	2.1

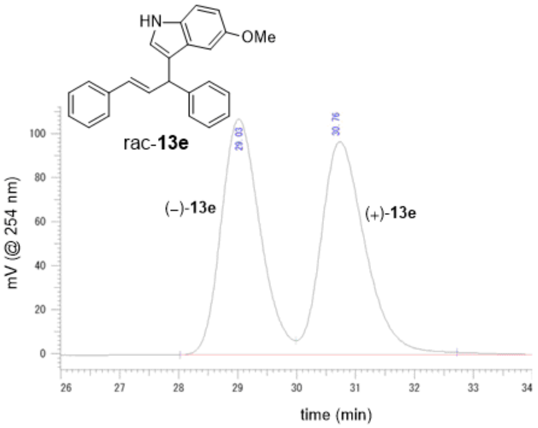

13e (using (*M*)-**L1** as a chiral ligand)

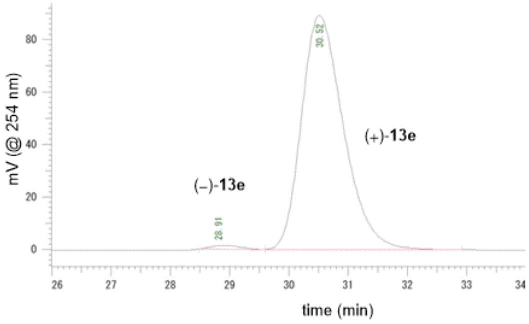

	RT	Area	Ratio
(–)-**13e**	29.03	4967456	50
(+)-**13e**	30.76	5041746	50

	RT	Area	Ratio
(–)-**13e**	28.91	49709	1.1
(+)-**13e**	30.52	4389112	98.9

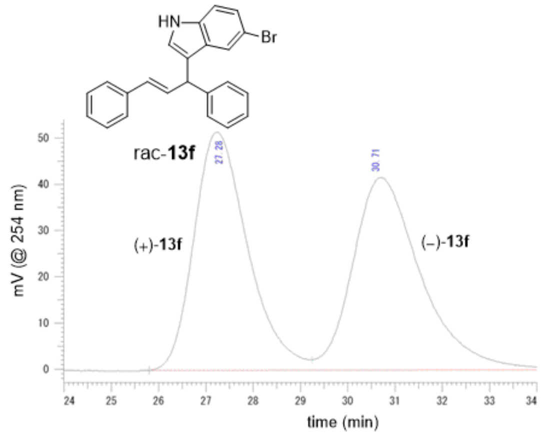

13f (using (*M*)-**L1** as a chiral ligand)

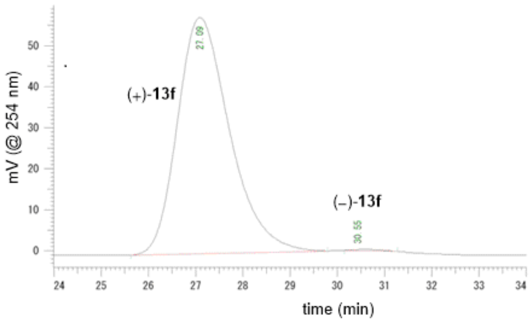

	RT	Area	Ratio
(+)-**13f**	27.28	4121468	50
(–)-**13f**	30.71	4158639	50

	RT	Area	Ratio
(+)-**13f**	27.09	4388028	99.7
(–)-**13f**	30.55	14483	0.3

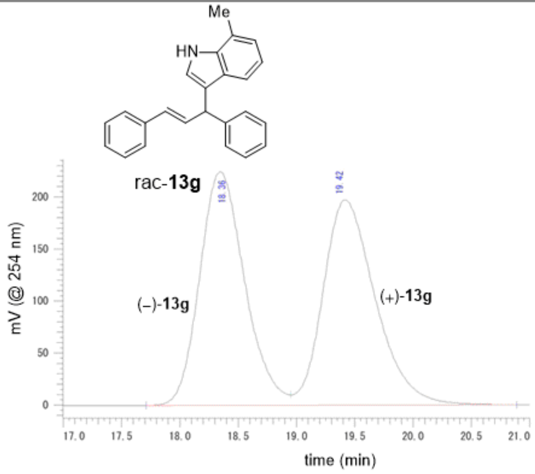

13g (using (*M*)-**L1** as a chiral ligand)

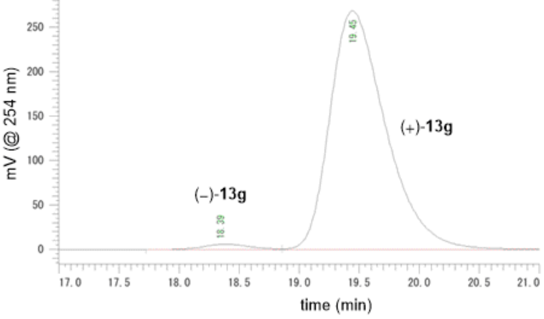

	RT	Area	Ratio
(–)-**13g**	18.36	6091088	50
(+)-**13g**	19.42	6223048	50

	RT	Area	Ratio
(–)-**13g**	18.39	163324	1.9
(+)-**13g**	19.45	8649496	98.1

14-3. Asymmetric etherification of alcohols.

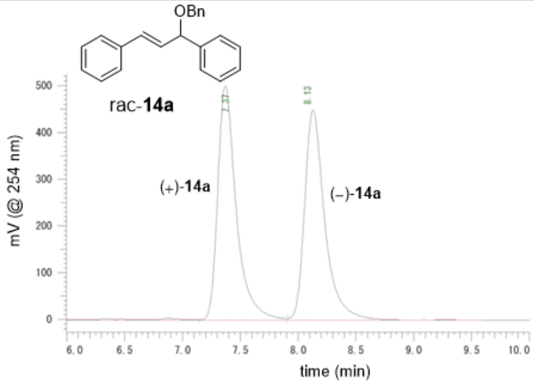

12a (using (*M*)-**L1** as a chiral ligand)

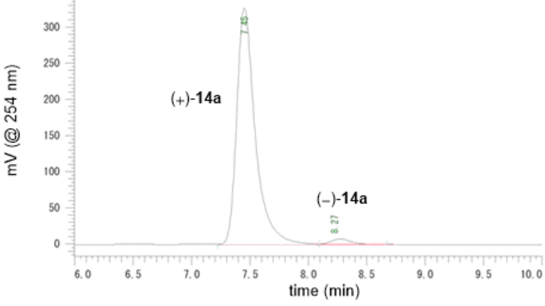

	RT	Area	Ratio
(+)-**14a**	7.37	5516662	50
(–)-**14a**	8.13	5401074	50

	RT	Area	Ratio
(+)-**14a**	7.45	3578692	98
(–)-**14a**	8.27	81014	2

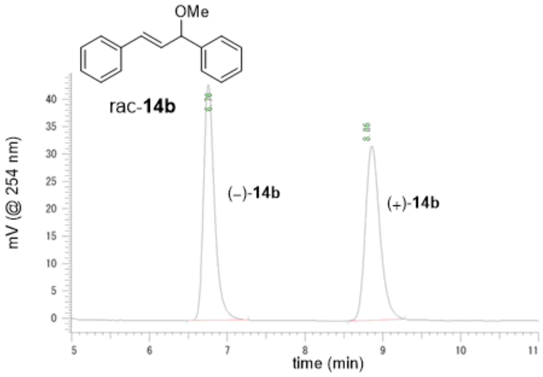

12b (using (M)-L1 as a chiral ligand)

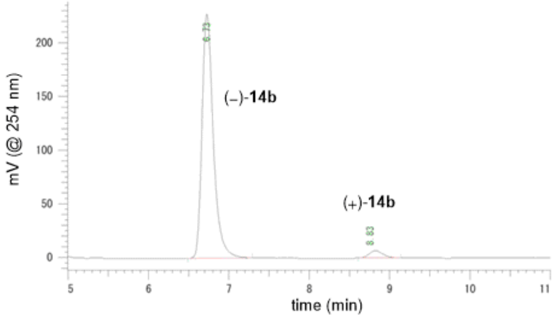

	RT	Area	Ratio
(–)-**14b**	6.76	408914	50
(+)-**14b**	8.86	401630	50

	RT	Area	Ratio
(–)-**14b**	6.73	2145995	96
(+)-**14b**	8.83	78682	4

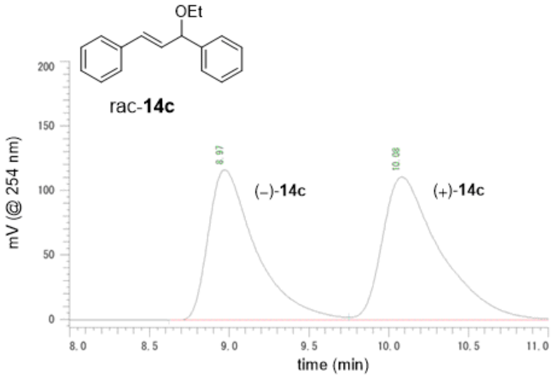

14c (using (M)-**L1** as a chiral ligand)

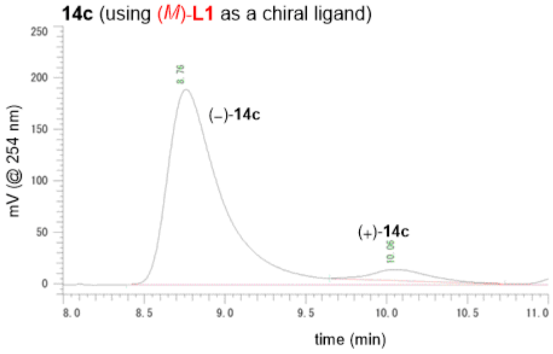

	RT	Area	Ratio
(–)-**14c**	8.97	2412870	46
(+)-**14c**	10.08	2793953	54

	RT	Area	Ratio
(–)-**14c**	8.76	4886896	95
(+)-**14c**	10.06	265256	5

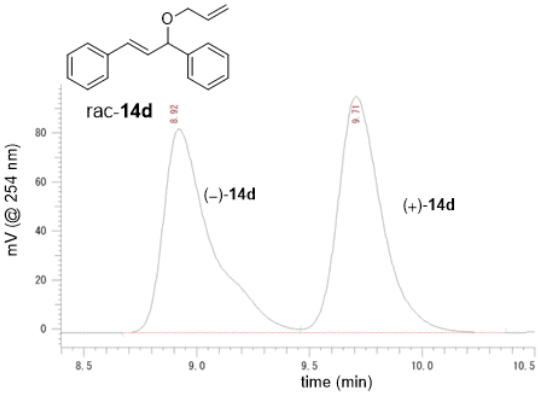

14d (using (M)-**L1** as a chiral ligand)

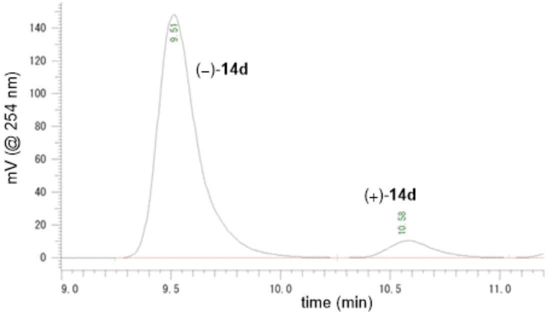

	RT	Area	Ratio
(–)-**14d**	8.92	1227887	49
(+)-**14d**	9.71	1294912	51

	RT	Area	Ratio
(–)-**14d**	9.51	1890432	92
(+)-**14d**	10.58	159205	8

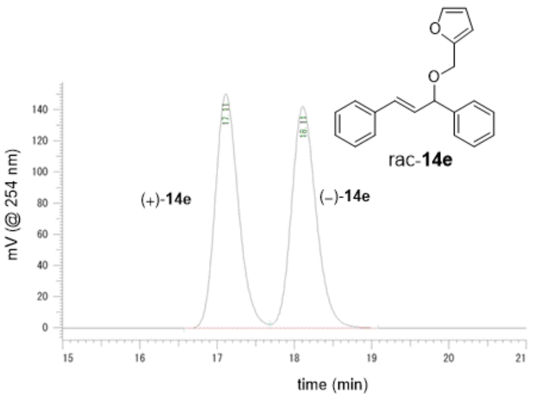

	RT	Area	Ratio
(+)-**14e**	17.11	3113070	50
(–)-**14e**	18.11	3133390	50

14e (using (*M*)-**L1** as a chiral ligand)

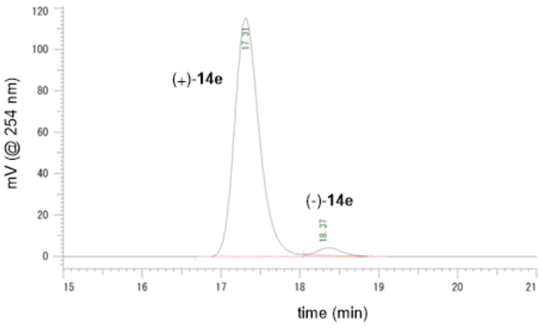

	RT	Area	Ratio
(+)-**14e**	17.31	2524138	97
(–)-**14e**	18.37	75549	3

14-4. Asymmetric Suzuki−Miyaura coupling.

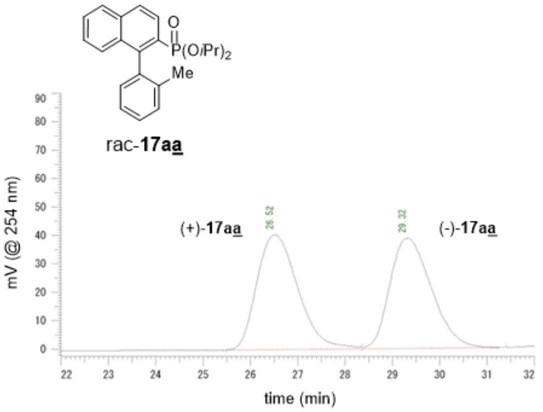

	RT	Area	Ratio
(+)-**17aa**	26.52	2403638	50
(-)-**17aa**	29.32	2337416	50

17aa (using (*P*)-**L1** as a chiral ligand)

	RT	Area	Ratio
(+)-**17aa**	25.21	2733654	90.7
(-)-**17aa**	28.29	279888	9.3

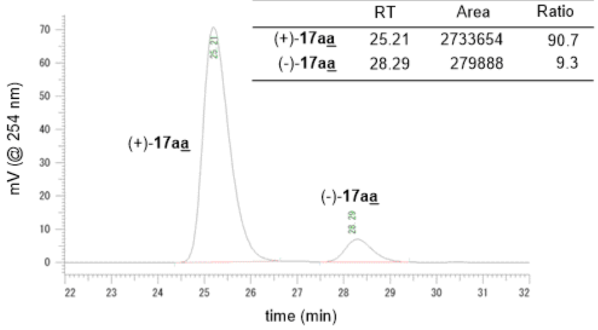

17aa (using (*P*)-**L2** as a chiral ligand)

	RT	Area	Ratio
(+)-**17aa**	25.56	2330117	97.6
(-)-**17aa**	28.76	56458	2.4

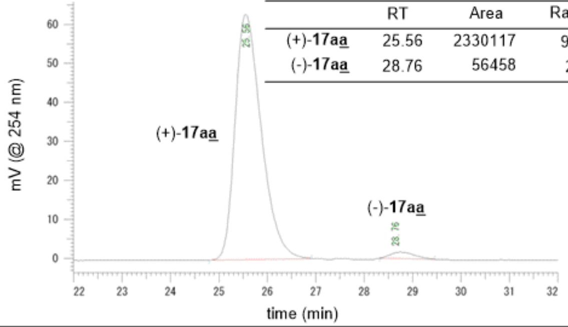

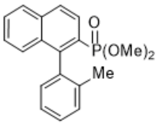

rac-**17ba**

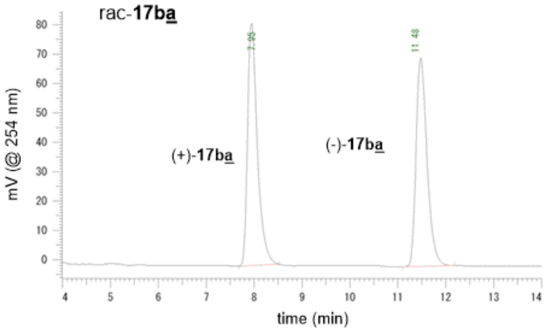

	RT	Area	Ratio
(+)-**17ba**	7.95	1115783	50
(-)-**17ba**	11.48	1094188	50

17ba (using (*P*)-**L1** as a chiral ligand)

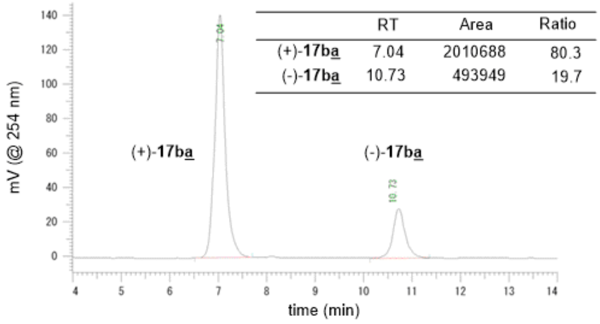

	RT	Area	Ratio
(+)-**17ba**	7.04	2010688	80.3
(-)-**17ba**	10.73	493949	19.7

11ba (using (*P*)-**L2** as a chiral ligand)

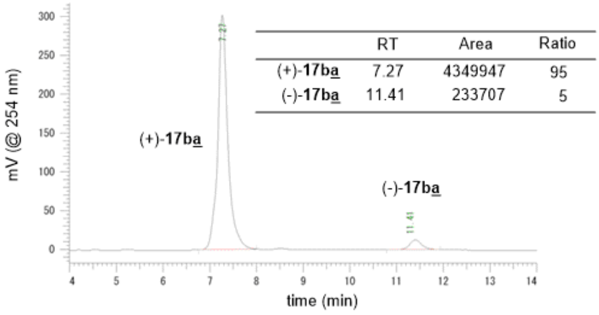

	RT	Area	Ratio
(+)-**17ba**	7.27	4349947	95
(-)-**17ba**	11.41	233707	5

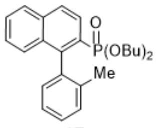

rac-**17ca**

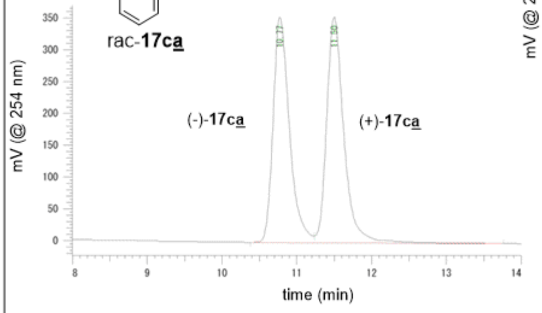

	RT	Area	Ratio
(-)-**17ca**	10.77	5334412	50
(+)-**17ca**	11.50	5656594	50

17ca (using (*P*)-**L1** as a chiral ligand)

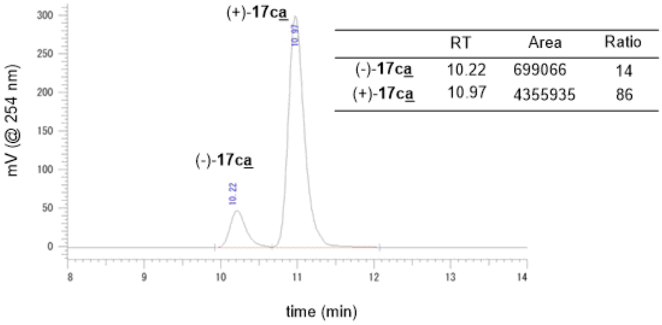

	RT	Area	Ratio
(-)-**17ca**	10.22	699066	14
(+)-**17ca**	10.97	4355935	86

17ca (using (*P*)-**L2** as a chiral ligand)

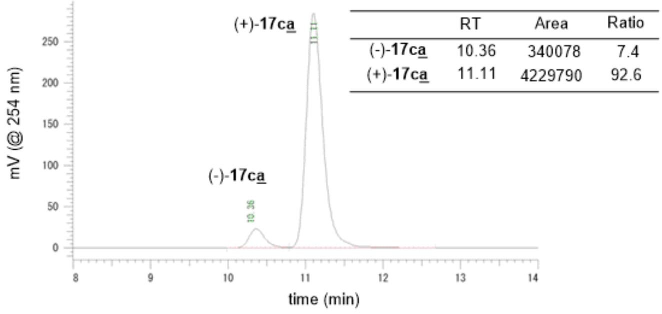

	RT	Area	Ratio
(-)-**17ca**	10.36	340078	7.4
(+)-**17ca**	11.11	4229790	92.6

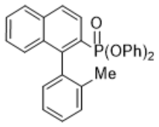

rac-**17da**

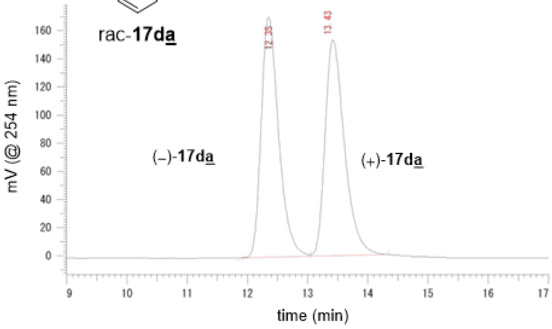

	RT	Area	Ratio
(–)-**17da**	12.35	3386011	50
(+)-**17da**	13.43	3390144	50

17da (using (*P*)-**L1** as a chiral ligand)

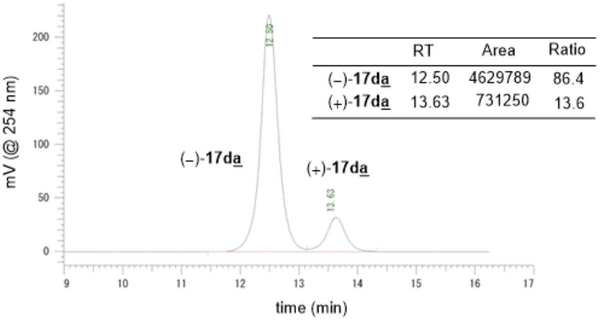

	RT	Area	Ratio
(–)-**17da**	12.50	4629789	86.4
(+)-**17da**	13.63	731250	13.6

17da (using (*P*)-**L2** as a chiral ligand)

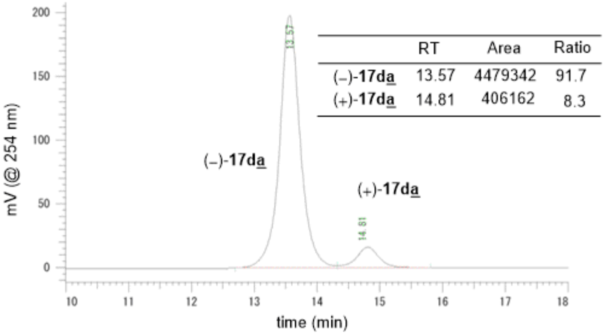

	RT	Area	Ratio
(–)-**17da**	13.57	4479342	91.7
(+)-**17da**	14.81	406162	8.3

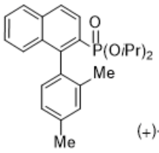

rac-**17ab**

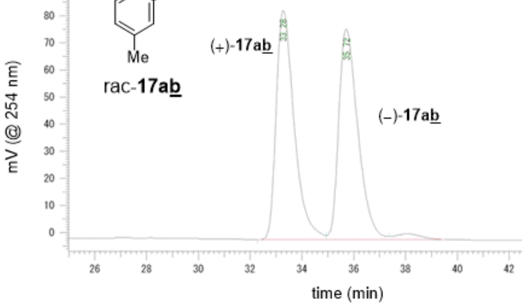

	RT	Area	Ratio
(+)-**17ab**	33.28	4025356	50
(–)-**17ab**	35.72	3930206	50

17ab (using (*P*)-**L1** as a chiral ligand)

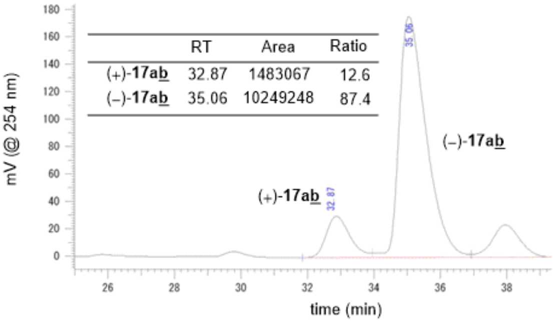

	RT	Area	Ratio
(+)-**17ab**	32.87	1483067	12.6
(–)-**17ab**	35.06	10249248	87.4

17ab (using (*P*)-**L2** as a chiral ligand)

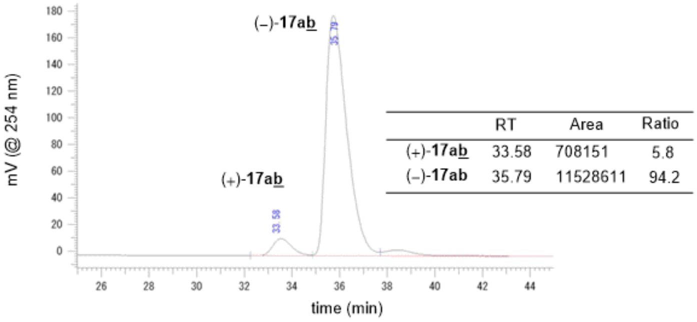

	RT	Area	Ratio
(+)-**17ab**	33.58	708151	5.8
(–)-**17ab**	35.79	11528611	94.2

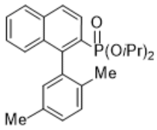

rac-**17ac**

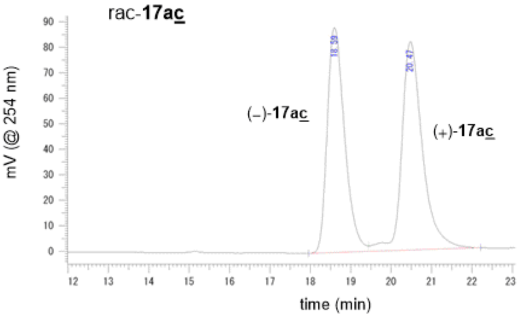

	RT	Area	Ratio
(–)-**17ac**	18.59	2654957	50
(+)-**17ac**	20.47	2772766	50

17ac (using (*P*)-**L1** as a chiral ligand)

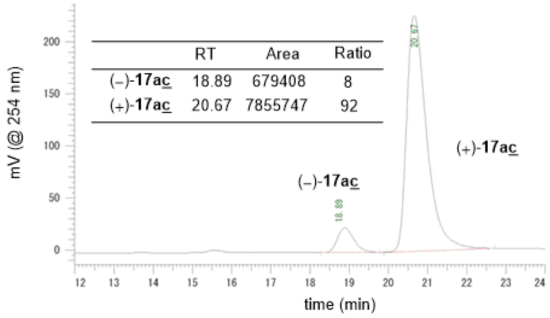

	RT	Area	Ratio
(–)-**17ac**	18.89	679408	8
(+)-**17ac**	20.67	7855747	92

17ac (using (*P*)-**L2** as a chiral ligand)

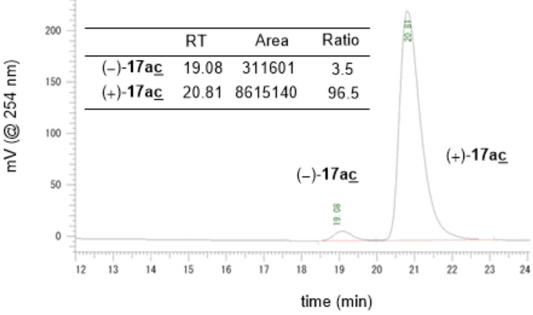

	RT	Area	Ratio
(–)-**17ac**	19.08	311601	3.5
(+)-**17ac**	20.81	8615140	96.5

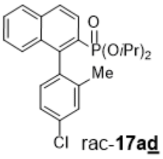

rac-**17ad**

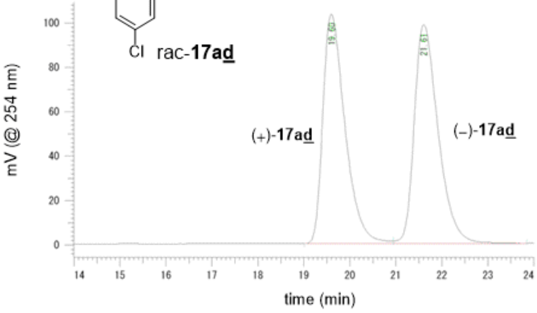

	RT	Area	Ratio
(+)-**17ad**	19.60	3409460	50
(–)-**17ad**	21.61	3503268	50

17ad (using (*P*)-**L1** as a chiral ligand)

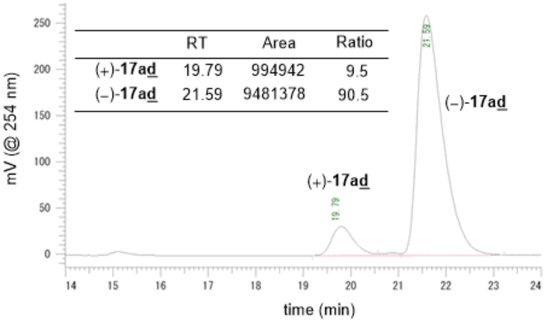

	RT	Area	Ratio
(+)-**17ad**	19.79	994942	9.5
(–)-**17ad**	21.59	9481378	90.5

17ad (using (*P*)-**L2** as a chiral ligand)

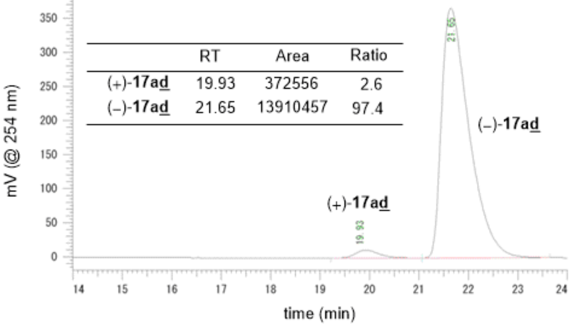

	RT	Area	Ratio
(+)-**17ad**	19.93	372556	2.6
(–)-**17ad**	21.65	13910457	97.4

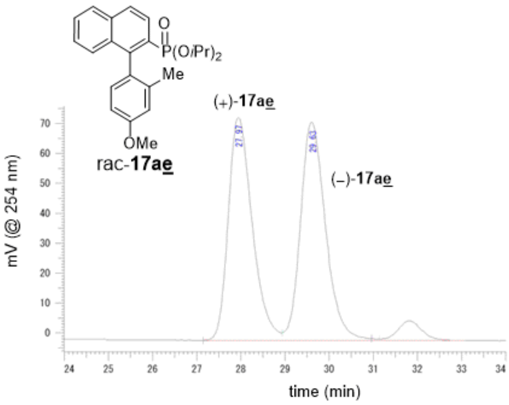

	RT	Area	Ratio
(+)-**17ae**	27.97	2832179	50
(–)-**17ae**	29.63	2850571	50

17ae (using (*P*)-**L1** as a chiral ligand)

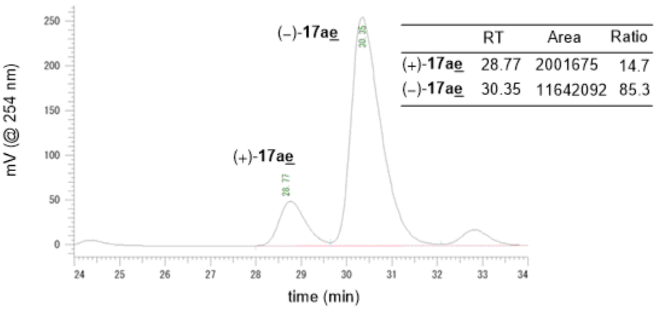

	RT	Area	Ratio
(+)-**17ae**	28.77	2001675	14.7
(–)-**17ae**	30.35	11642092	85.3

17ae (using (*P*)-**L2** as a chiral ligand)

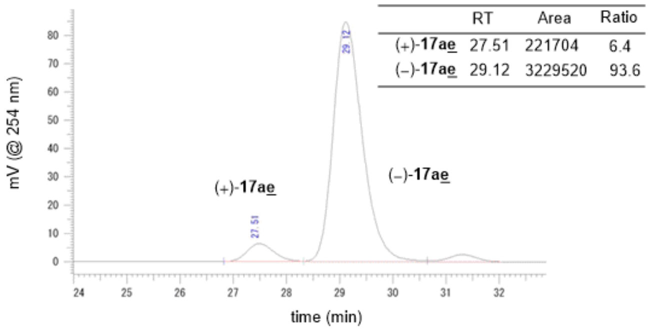

	RT	Area	Ratio
(+)-**17ae**	27.51	221704	6.4
(–)-**17ae**	29.12	3229520	93.6

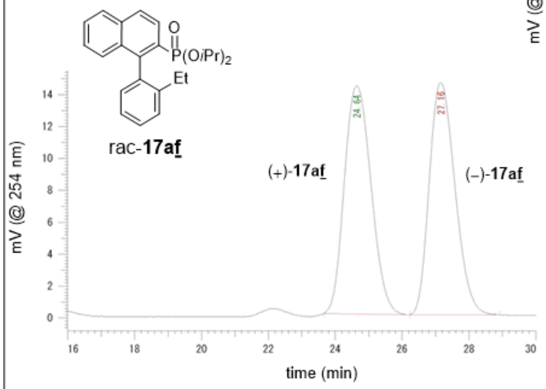

	RT	Area	Ratio
(+)-**17af**	24.64	782951	50
(–)-**17af**	27.16	767124	50

17af (using (*P*)-**L1** as a chiral ligand)

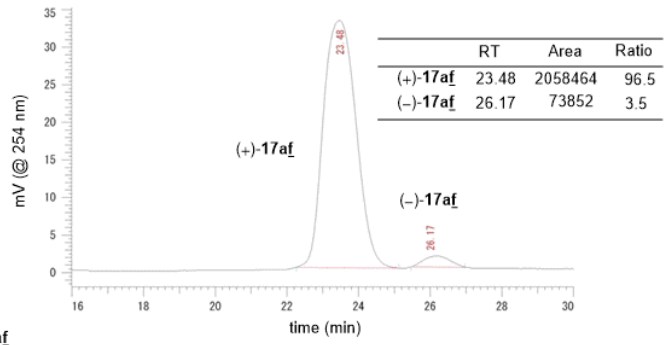

	RT	Area	Ratio
(+)-**17af**	23.48	2058464	96.5
(–)-**17af**	26.17	73852	3.5

17af (using (*M*)-**L2** as a chiral ligand)

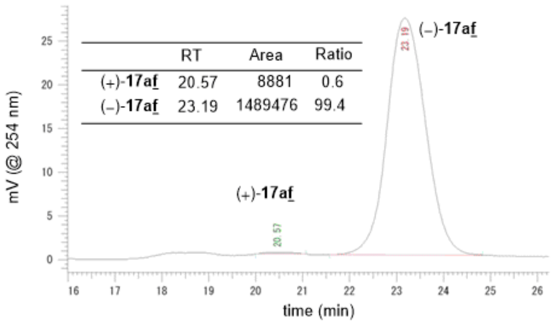

	RT	Area	Ratio
(+)-**17af**	20.57	8881	0.6
(–)-**17af**	23.19	1489476	99.4

15. OPTEP drawings of compounds L1 and L2 with 50% ellipsoid probability. The hydrogen atoms have been omitted clarity[13]

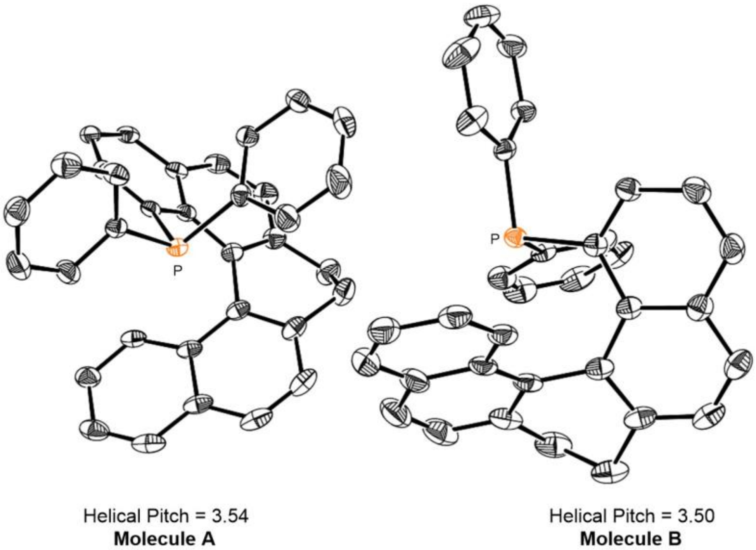

Figure S3. X-ray structure of **L1**

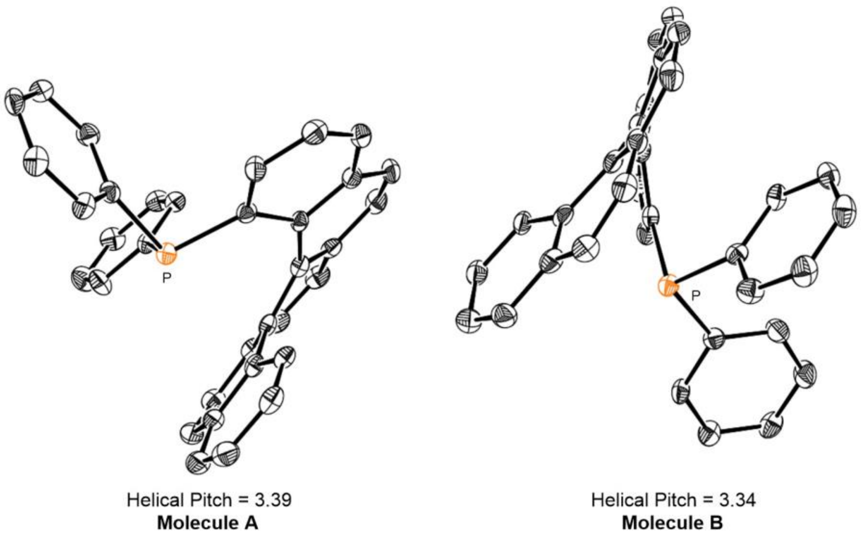

Figure S4. X-ray structure of **L2**

[13] The helical pitch P_h [Å], obtained by total least-squares method with the program HELFIT. Hence, pitch lengths calculated by using the inner carbon atoms (C14, C14a, C14b, C14c, C14d, and C1), see: P. Enkhbayar, S. Damdinsuren, M. Osaki, N. Matsushima, *Comput. Biol. Chem.* **2008**, *32*, 307.

16. Isolation and X-ray structure of Pd(dba)[L1] 10

A solution of **L1** (2.2 mg, 0.005 mmol) and $Pd_2(dba)_3$•$CHCl_3$ (2.0 mg, 0.002 mmol, [Pd]:**L1** = 1:1) in THF was stirred for 1h at 50 ºC under an argon atmosphere. After cooling to room temperature, the solvent was removed under reduced pressure. The residue was purified by flash column chromatography on silica gel eluting with hexane−EtOAc (1:1), followed by recrystallized from acetone to afford complex **10** as a red X-ray quality single crystals (Figure S5).

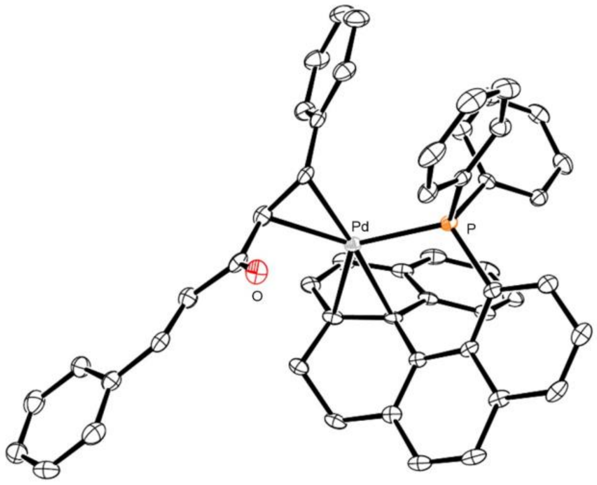

Figure S5. X-ray structure of Pd(dba)[**L1**] **10**

17. Table S6. Crystallographic data collection and structural refinement information

Compound	**L1**	**L2**	**10**
Empirical formula	$C_{34}H_{25}P$	$C_{34}H_{23}P$	$C_{51}H_{39}OPPd$
Formula weight	464.55	462.53	805.25
Temperature/K	120	123	90
Crystal system	triclinic	triclinic	monoclinic
Space group	*P*-1 (#2)	*P*-1 (#2)	$P2_1$/c (#14)
a/Å	9.7790(6)	9.630(3)	9.946(3)
b/Å	15.3746(9)	11.883(4)	15.338(4)
c/Å	17.8375(10)	21.146(6)	24.727(6)
α/°	68.4830(10)	82.777(10)	90
β/°	85.5650(10)	80.433(8)	96.359(3)
γ/°	87.1020(10)	87.857(10)	90
Volume/Å^3	2486.8(3)	2366.8(13)	3748.9(15)
Z	4	4	4
Density (calculated) Mg/cm^3	1.241	1.298	1.427
Absorption coefficient/mm^{-1}	0.131	0.137	0.578
Crystal size/mm^3	0.5 x 0.3 x 0.3	0.2 x 0.2 x 0.15	0.5 x 0.3 x 0.3
F(000)	976.0	968.0	1656.0
Radiation	Mo-K_α	Mo-K_α	Mo-K_α
R_1 (I＞2σ(I))[a]	0.0364	0.0881	0.0440
wR_2[a]	0.0820	0.2161	0.1128
GOF	1.034	1.071	0.998
CCDC No	**1431191**	**1431193**	**1458065**
