# Supplementary material for: Rational Design and Synthesis of [5]Helicene-Derived Phosphine Ligands and Their Application in Pd-Catalyzed Asymmetric Reactions
Source: Sci Rep. 2016 Nov 8;6:36211. doi: 10.1038/srep36211 (PMC5099951; doi:10.1038/srep36211)

Copies of NMR spectra

^{1}H NMR (500 MHz, $CDCl_3$) spectrum of compound **5**

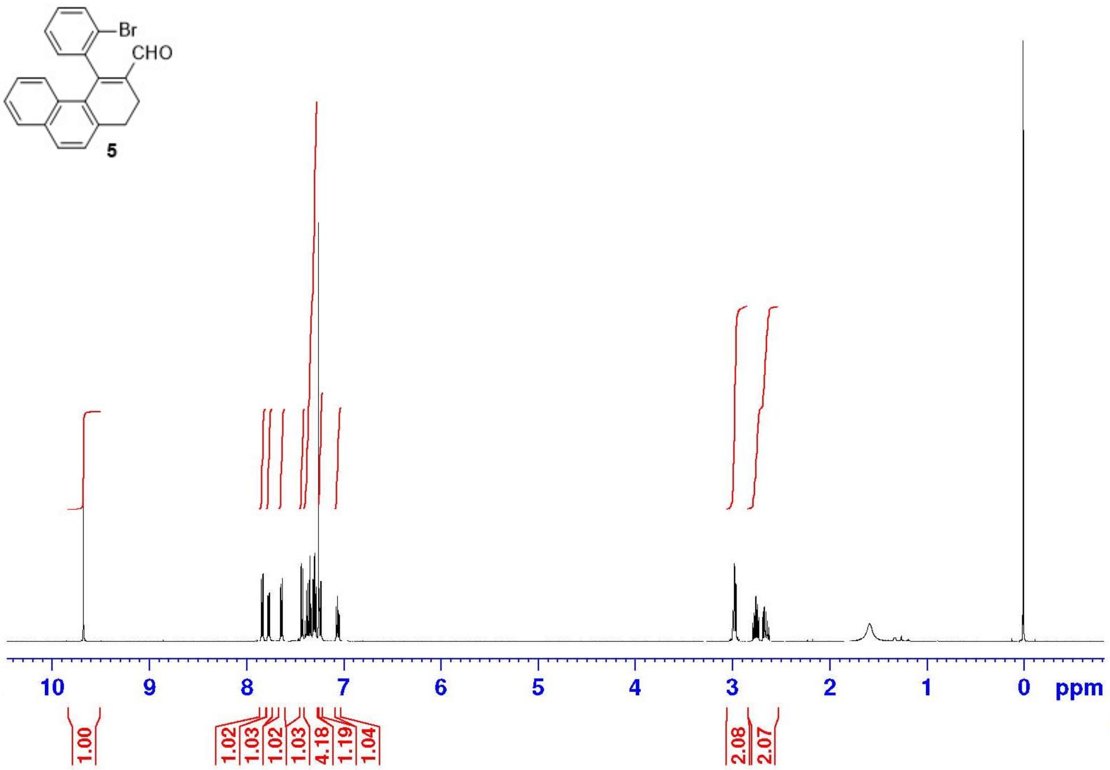

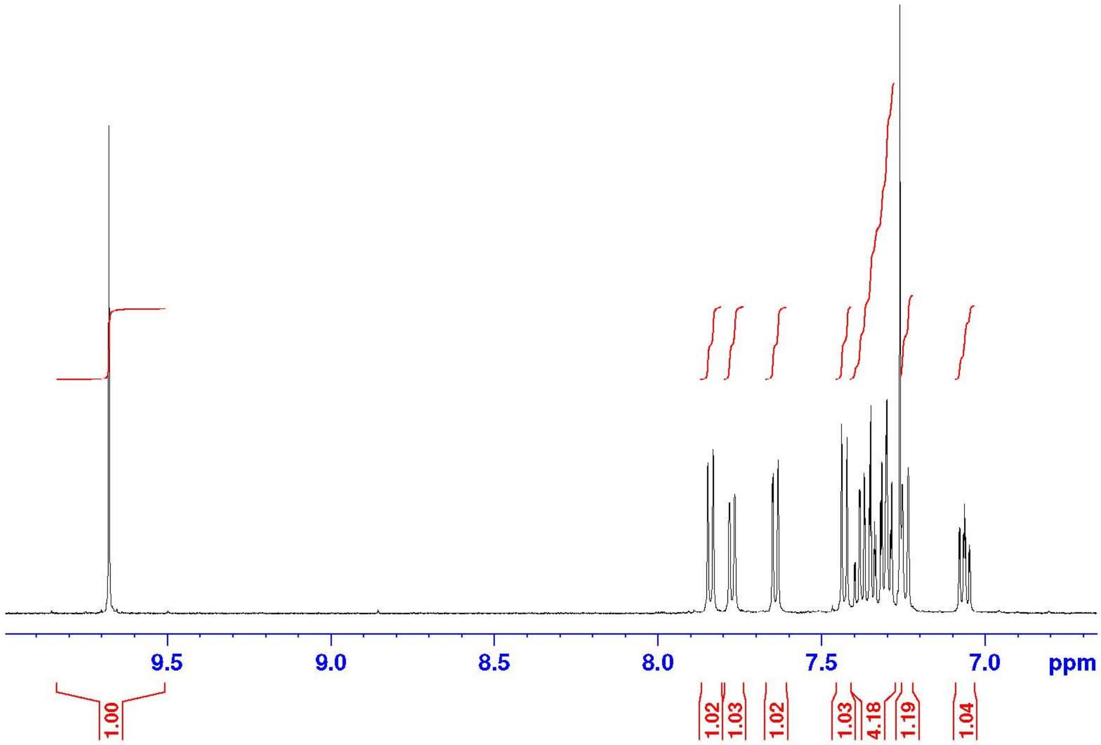

^{13}C NMR (125 MHz, $CDCl_3$) spectrum of compound **5**

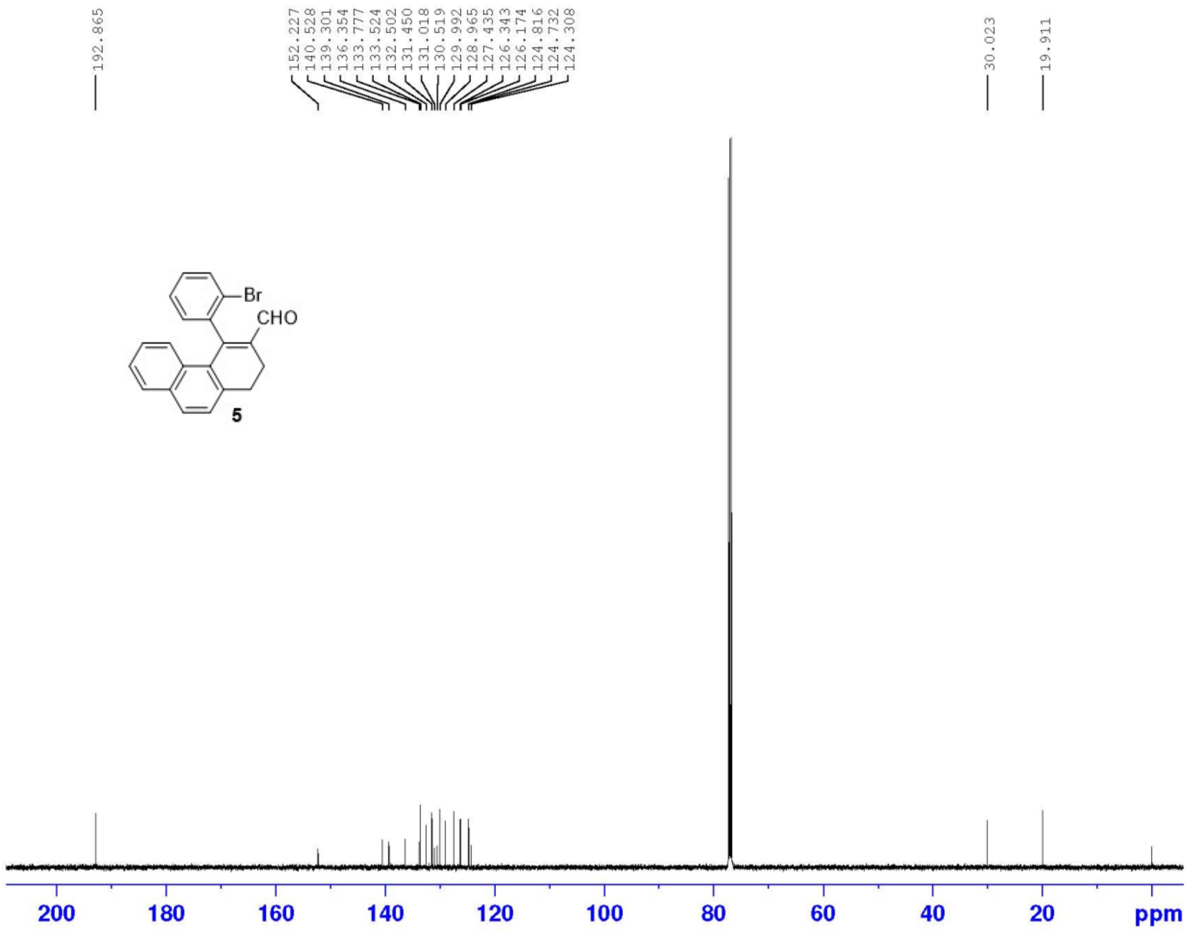

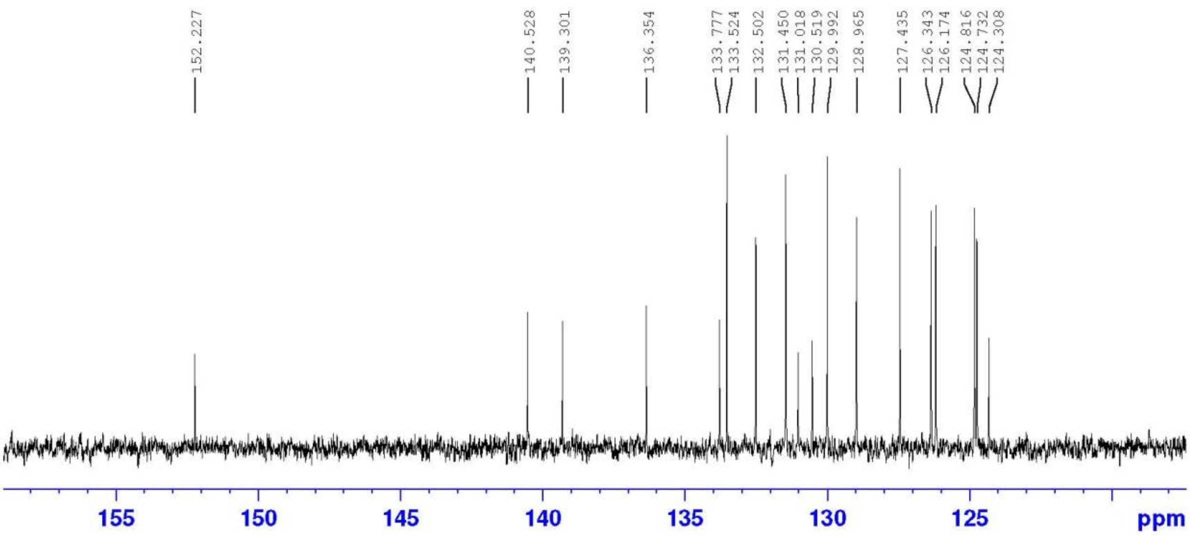

DEPT (125 MHz, $CDCl_3$) spectrum of compound **5**

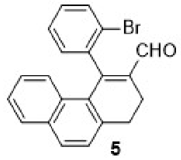

5

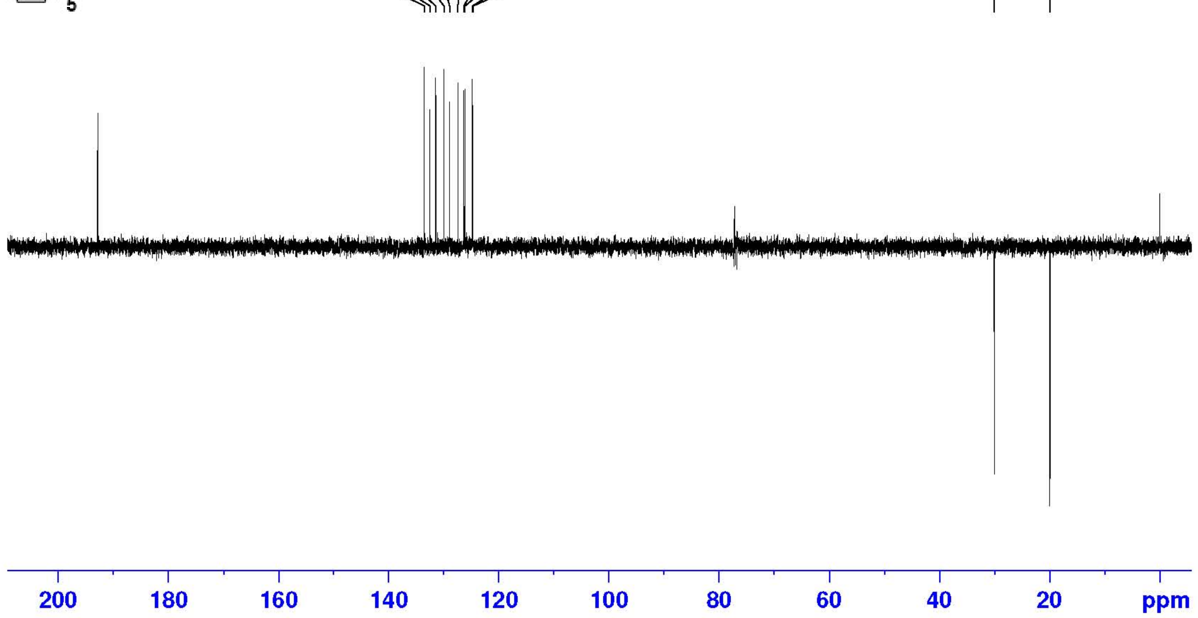

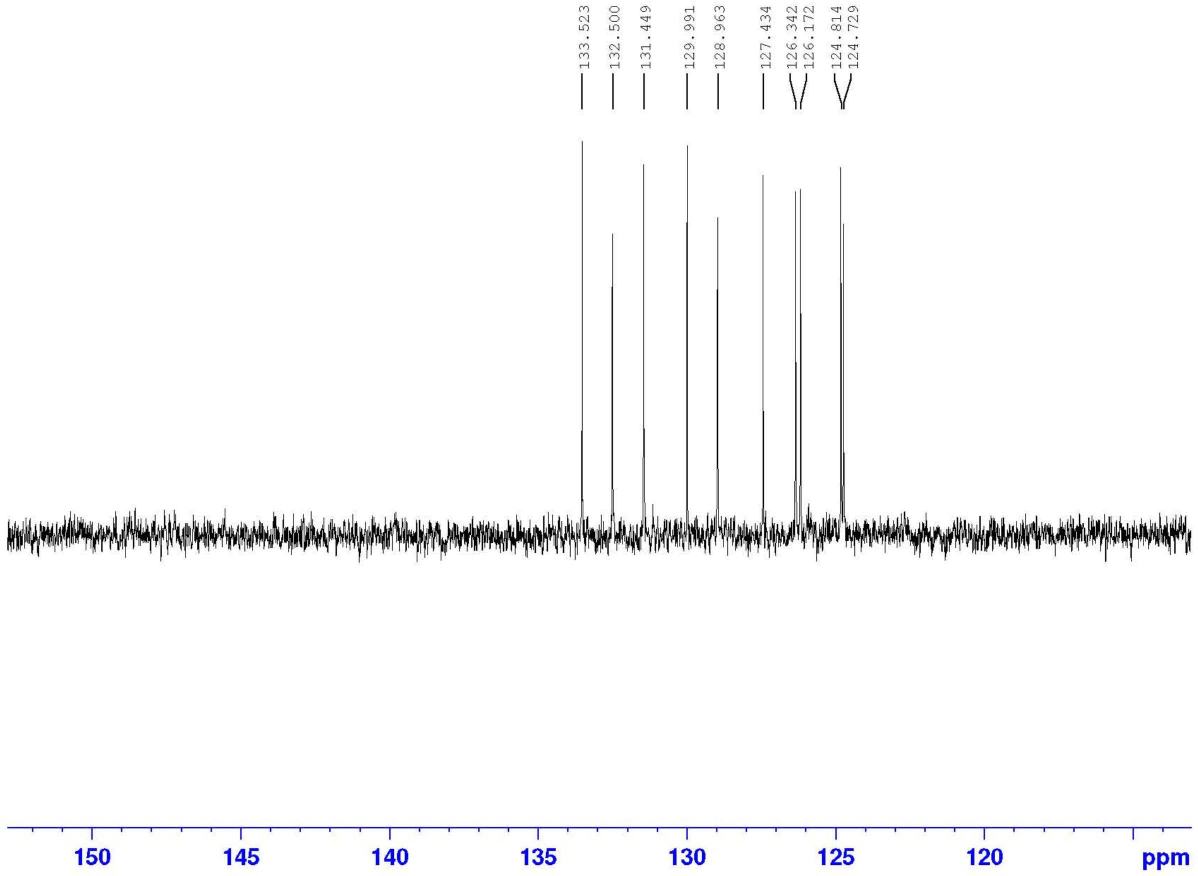

^{1}H NMR (500 MHz, $CDCl_3$) spectrum of compound **6**

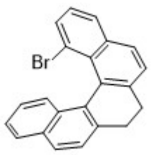

6

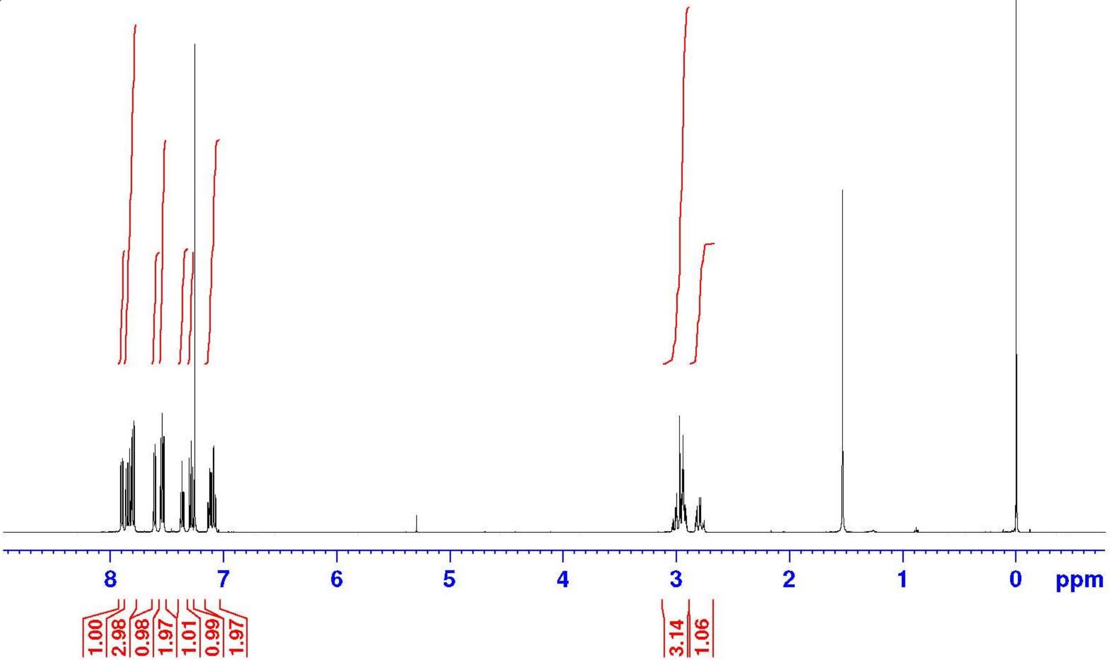

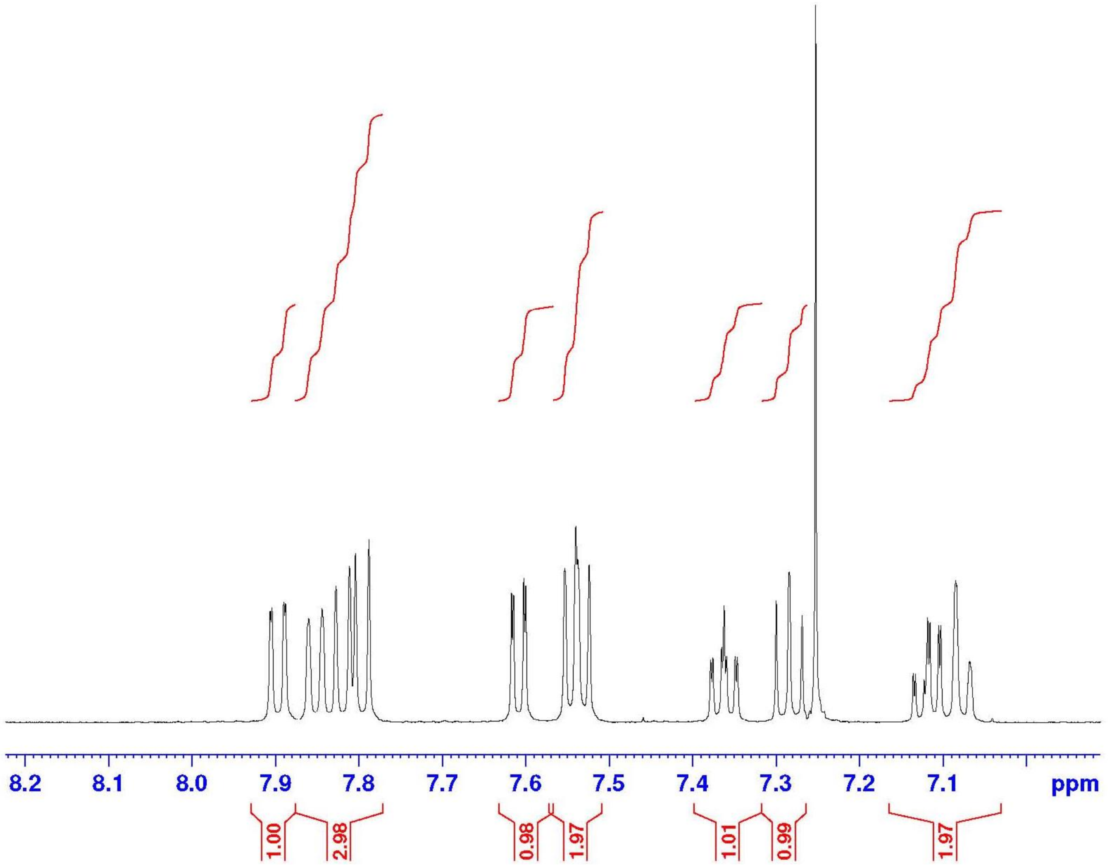

^{13}C NMR (125 MHz, $CDCl_3$) spectrum of compound **6**

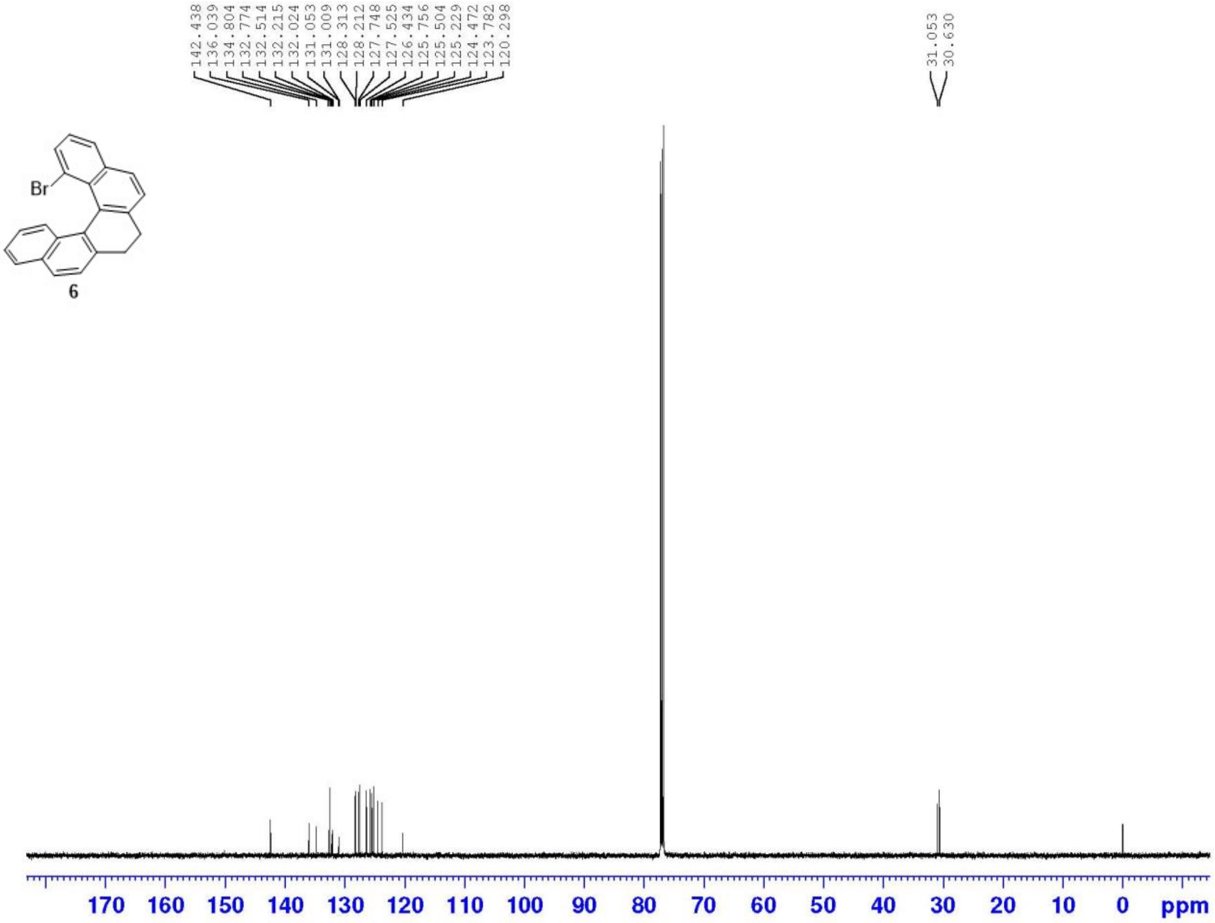

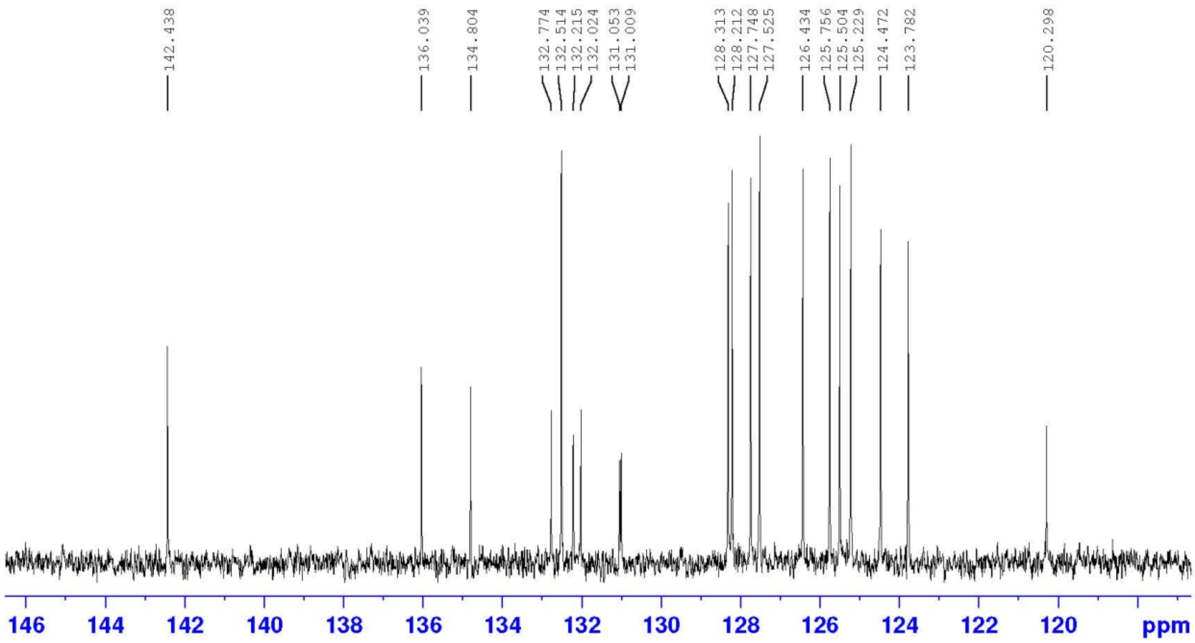

DEPT (125 MHz, $CDCl_3$) spectrum of compound **6**

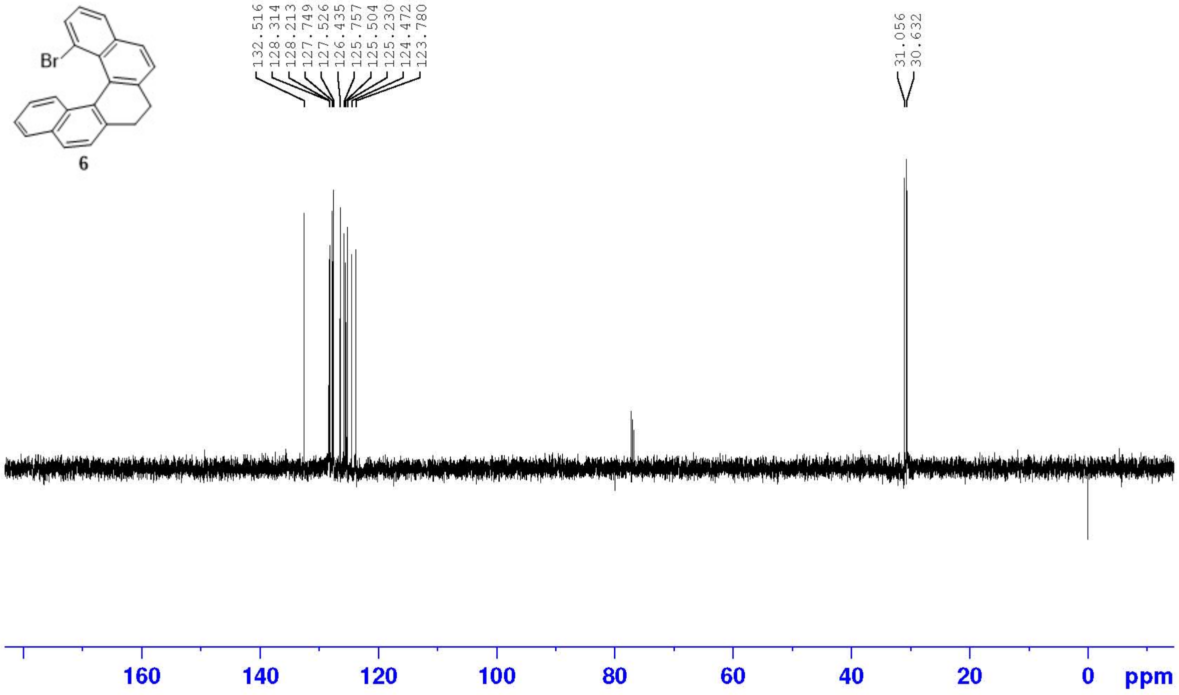

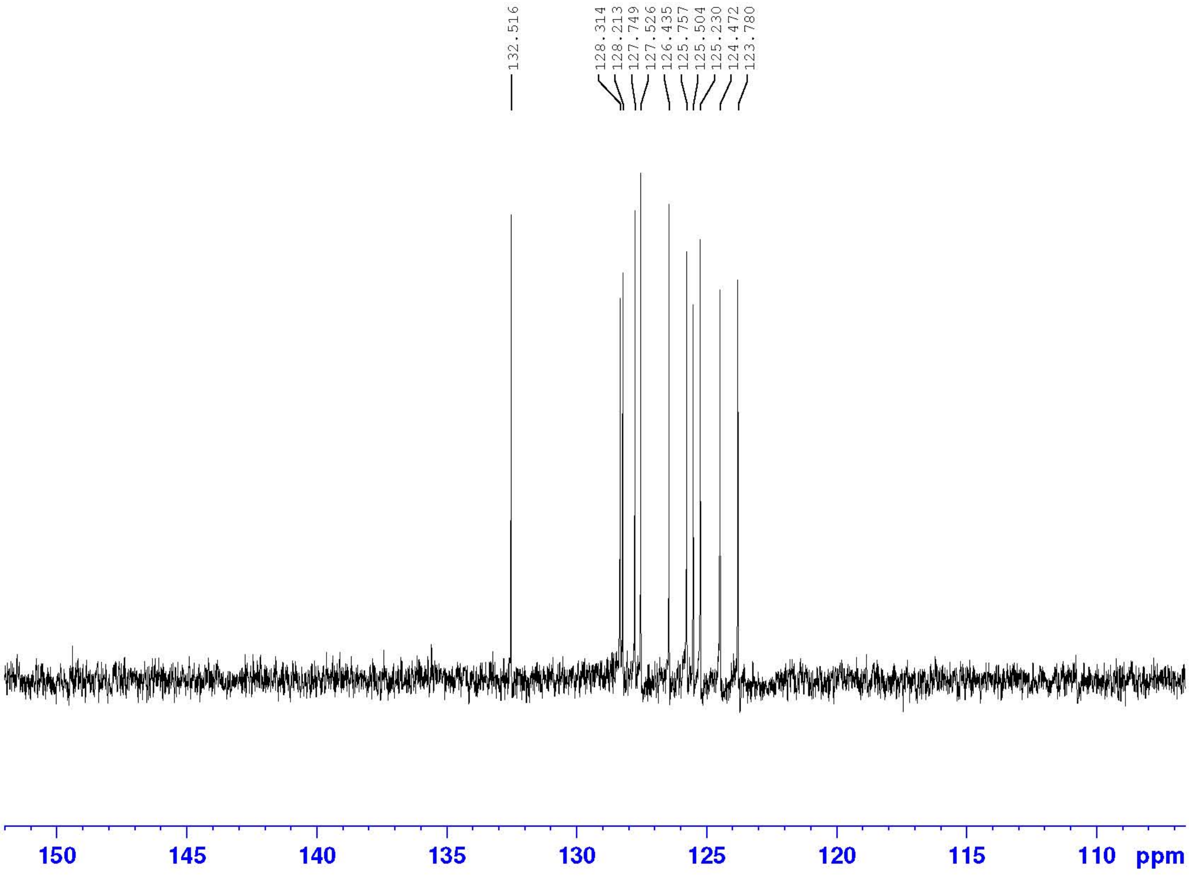

^{1}H NMR (500 MHz, $CDCl_3$) spectrum of compound **7**

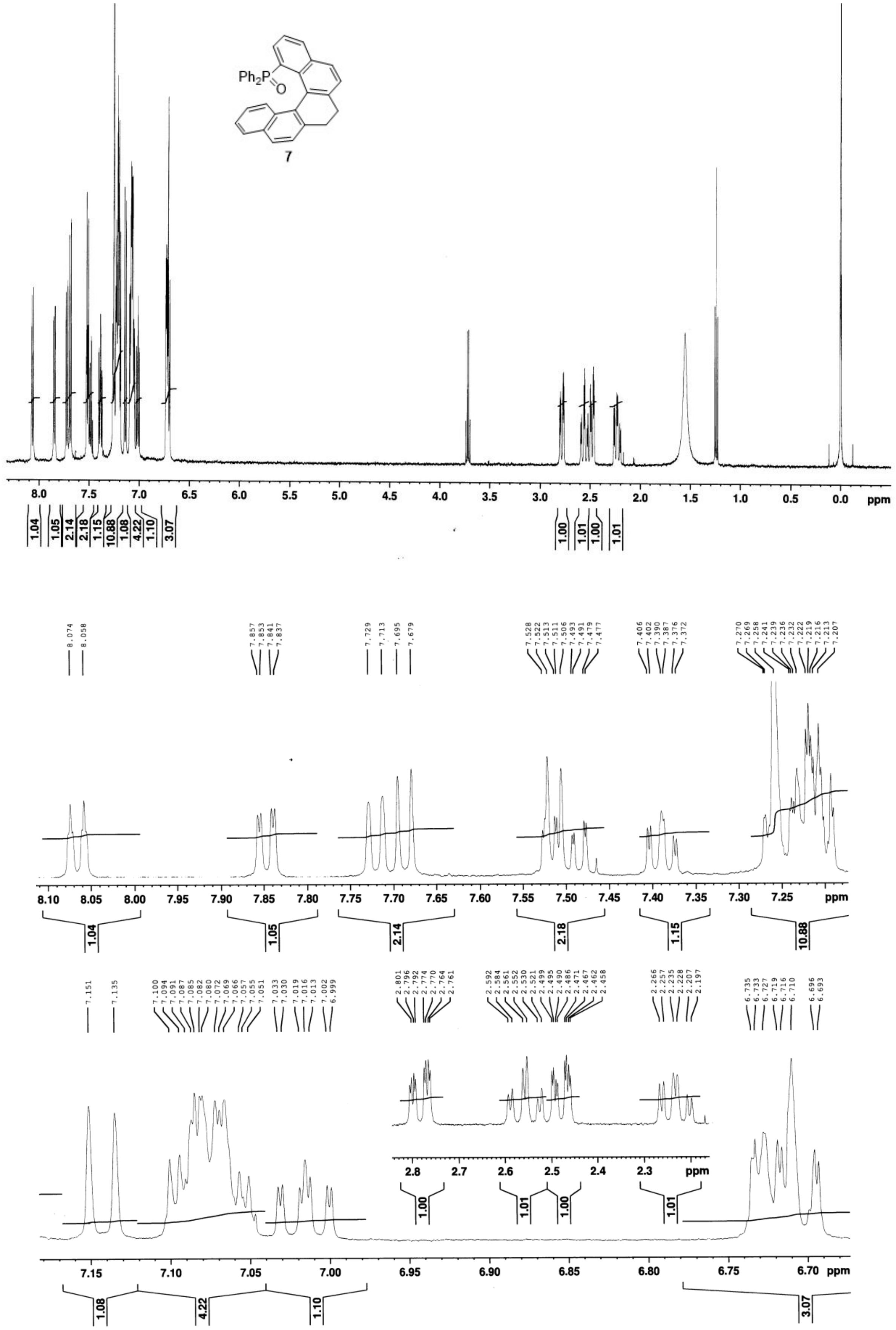

^{13}C NMR (125 MHz, $CDCl_3$) spectrum of compound **7**

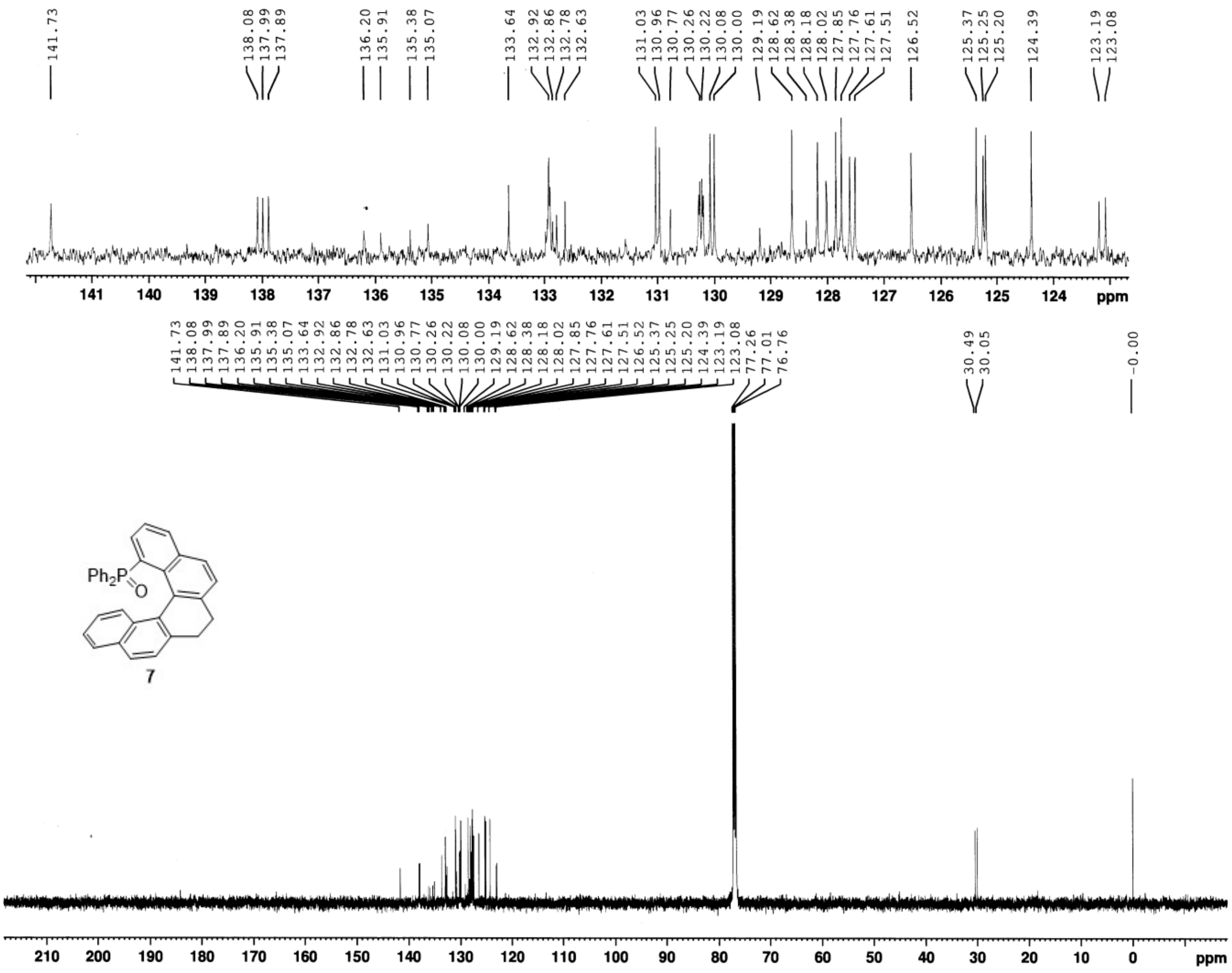

DEPT (125 MHz, $CDCl_3$) spectrum of compound **7**

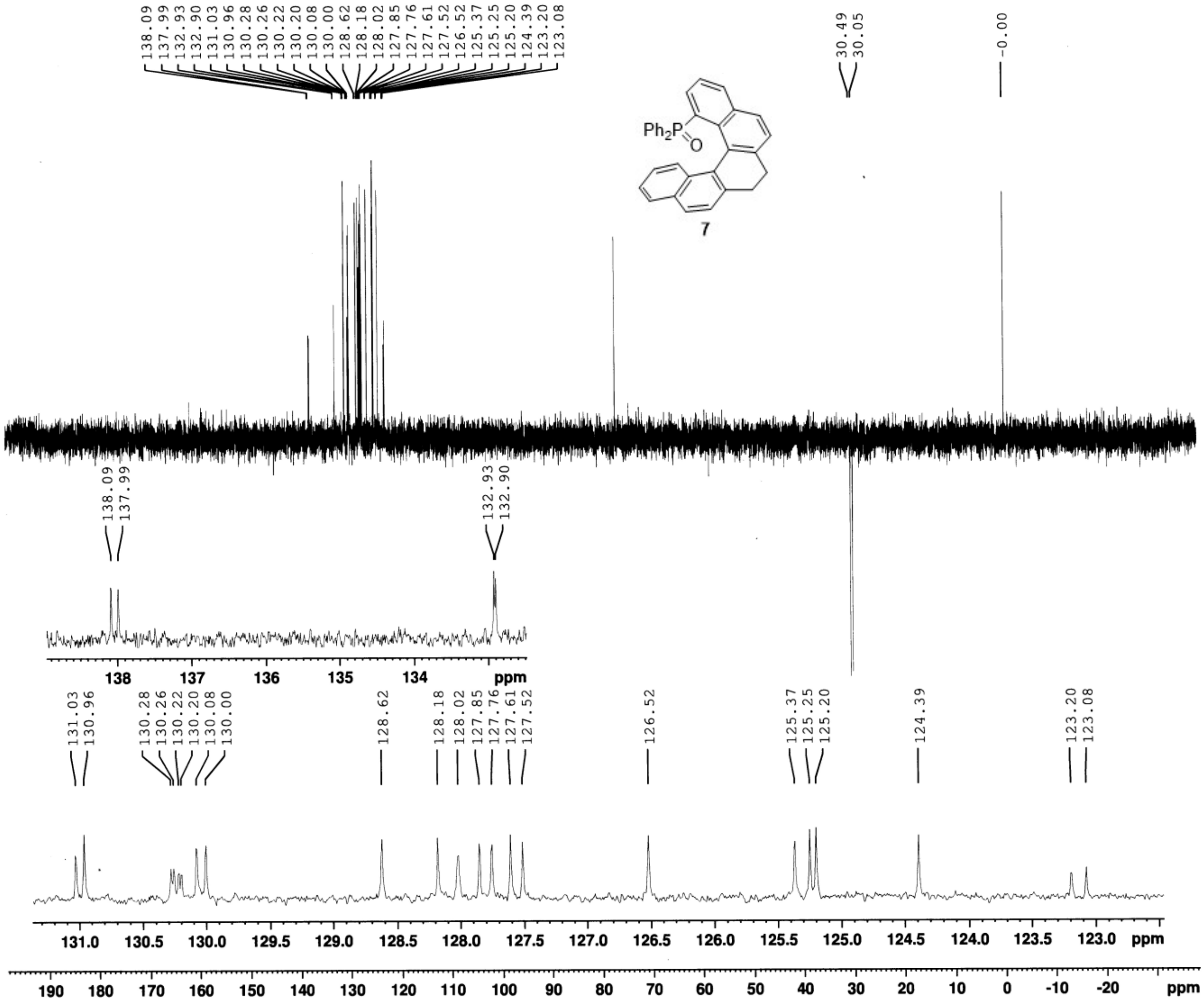

^{31}P NMR (202 MHz, $CDCl_3$) spectrum of compound **7**

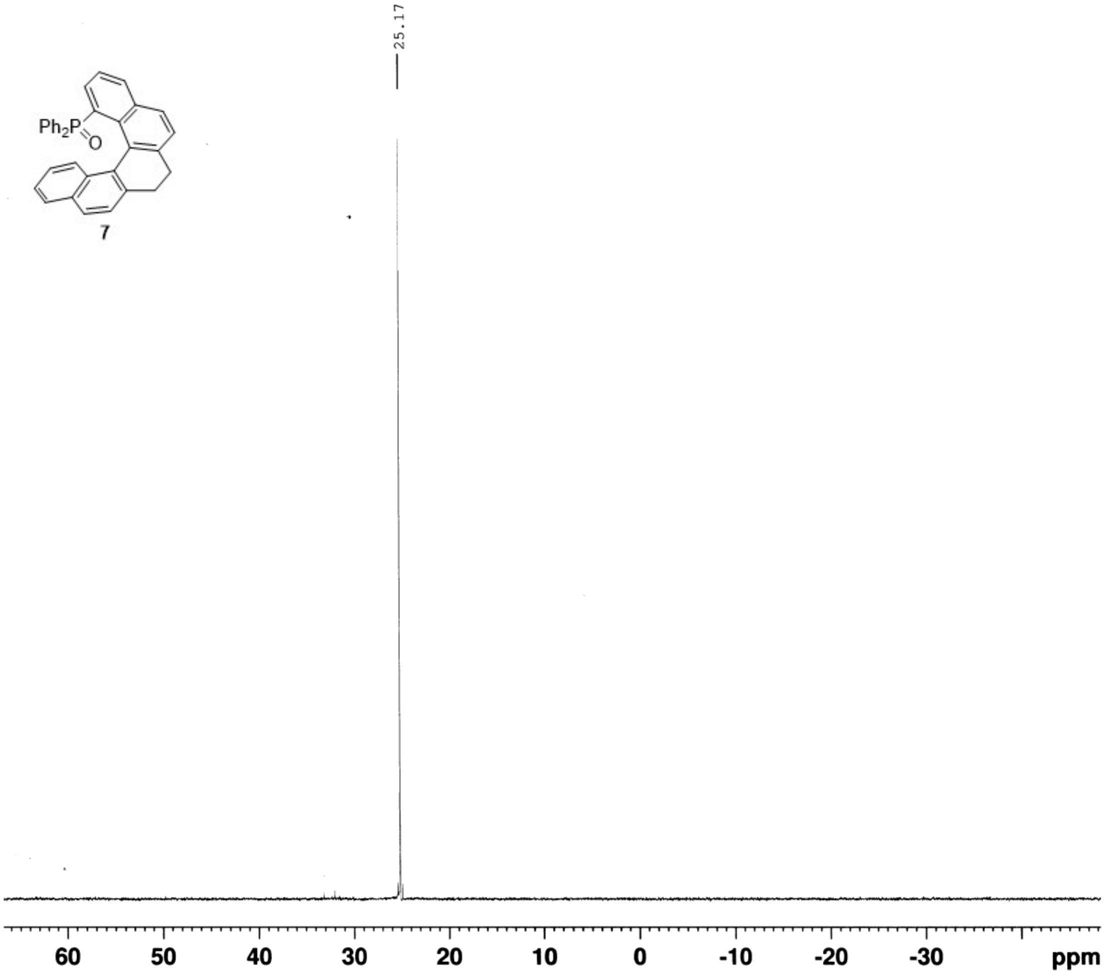

^{1}H NMR (500 MHz, $CDCl_3$) spectrum of compound **L1**

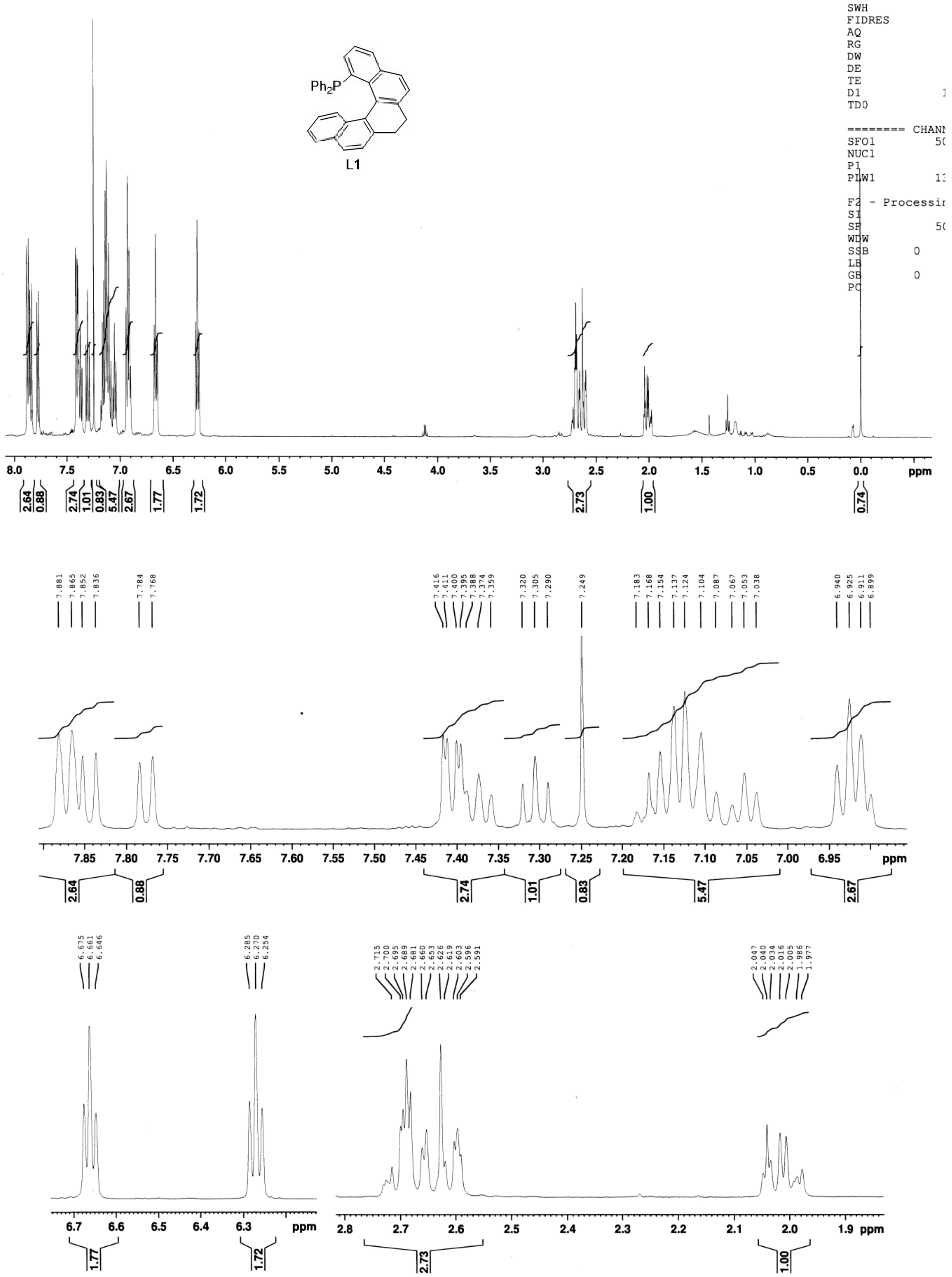

^{13}C NMR (125 MHz, $CDCl_3$) spectrum of compound **L1**

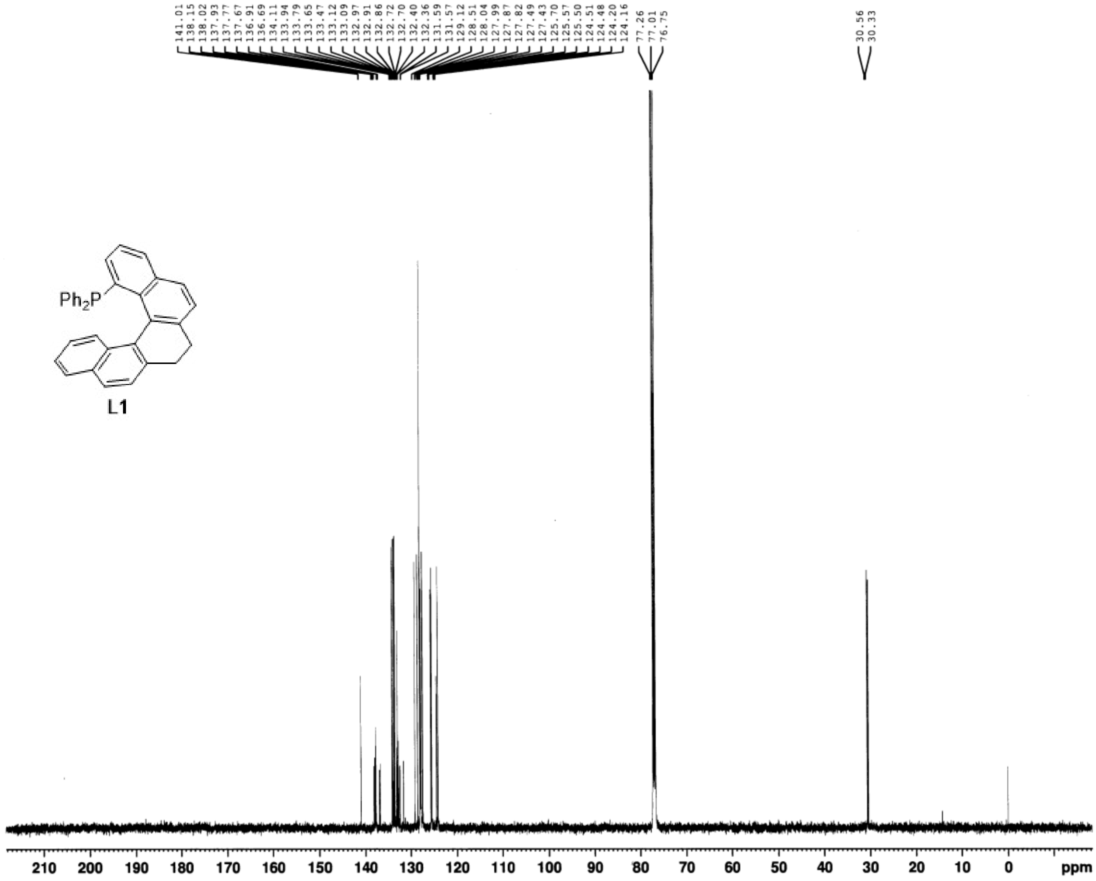

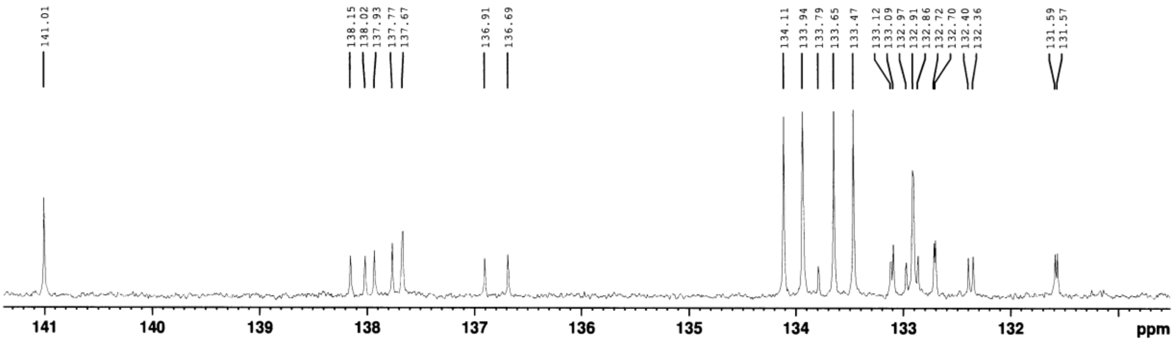

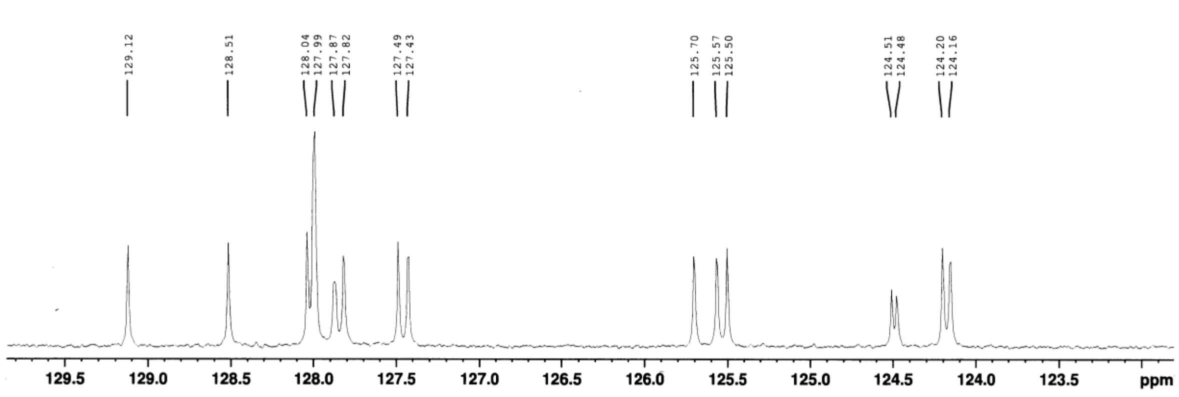

DEPT (125 MHz, $CDCl_3$) spectrum of compound **L1**

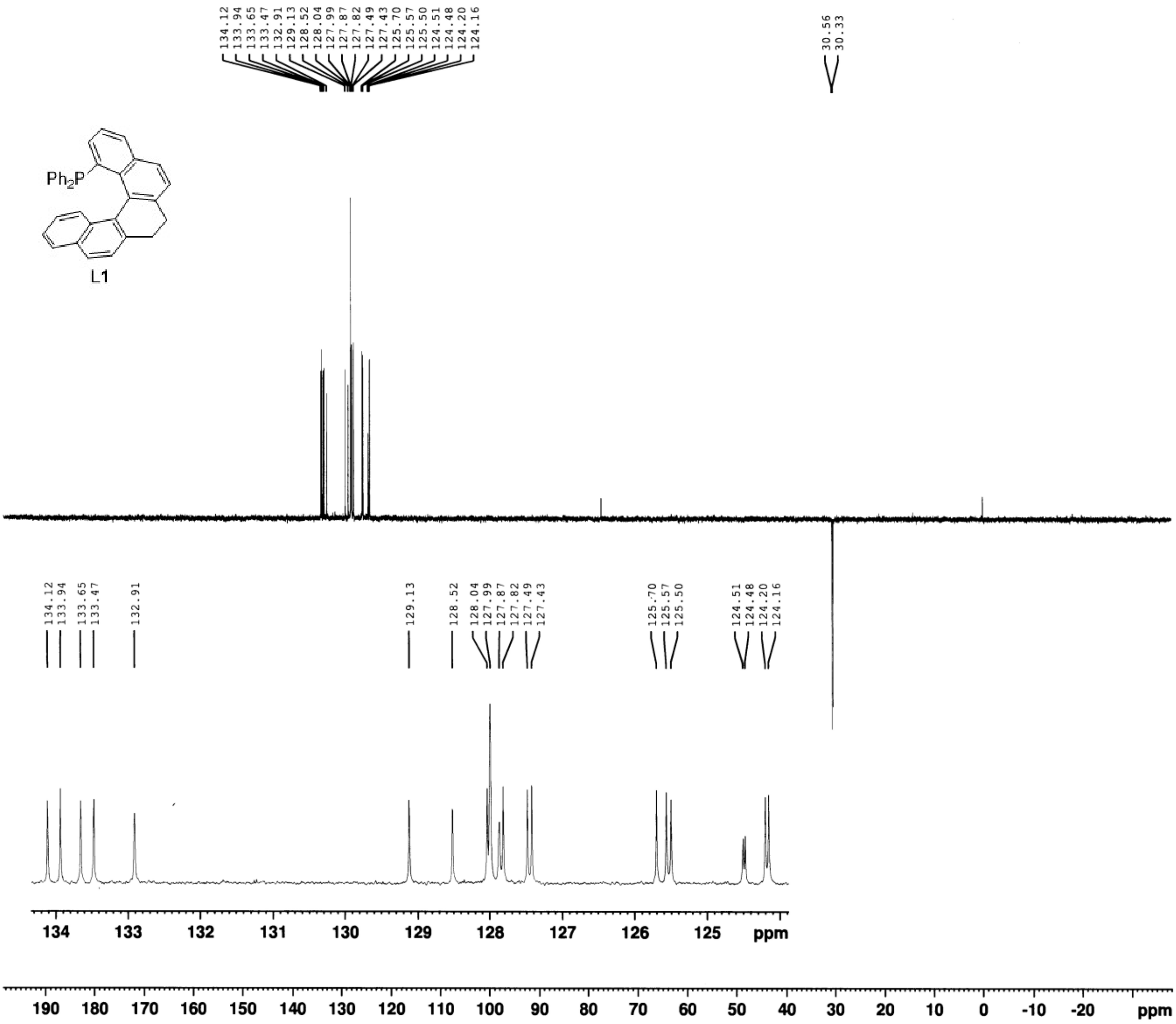

^{31}P NMR (202 MHz, $CDCl_3$) spectrum of compound **L1**

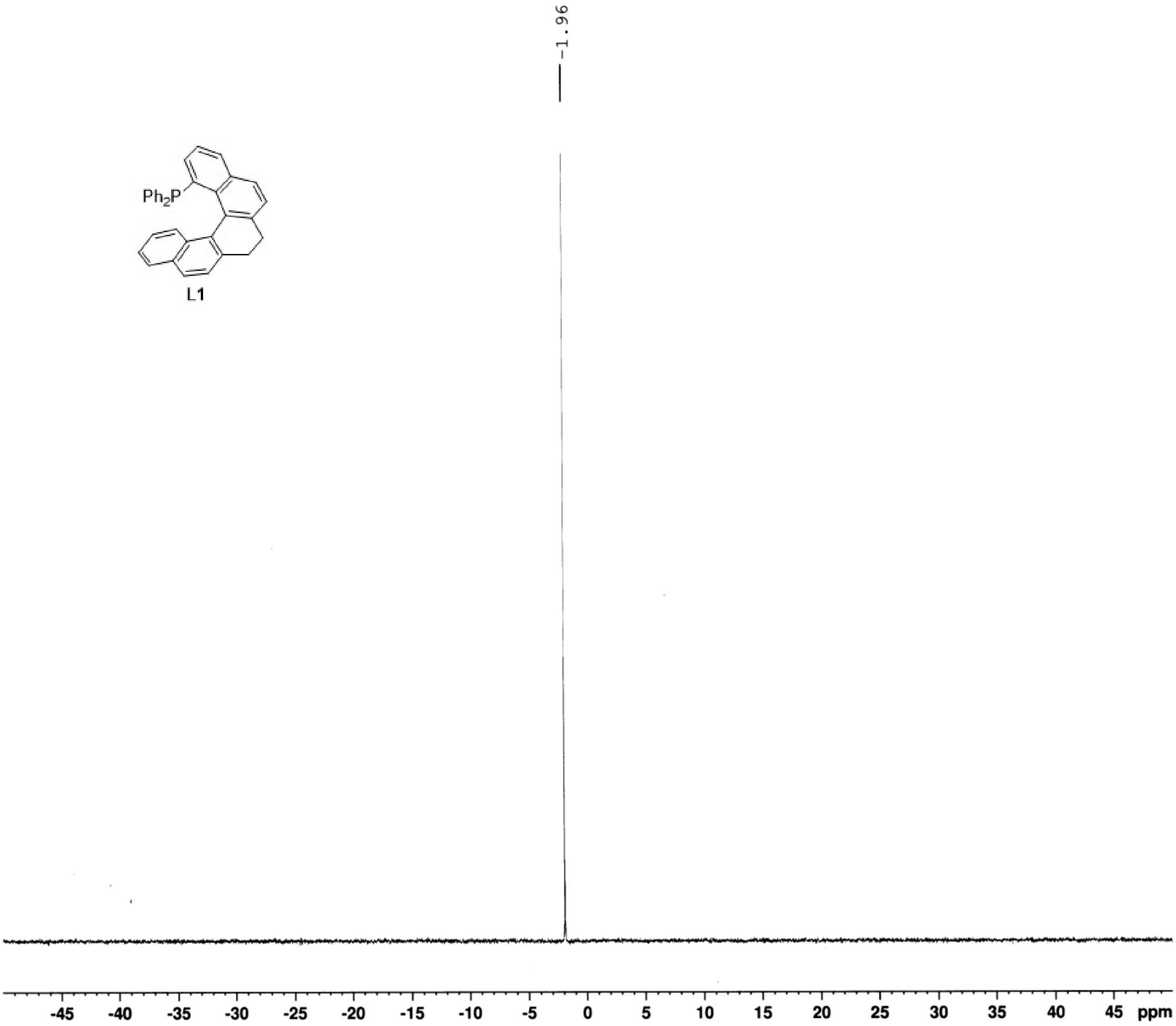

^{1}H NMR (500 MHz, $CDCl_3$) spectrum of compound **9**

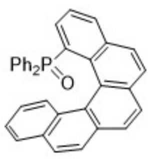

9

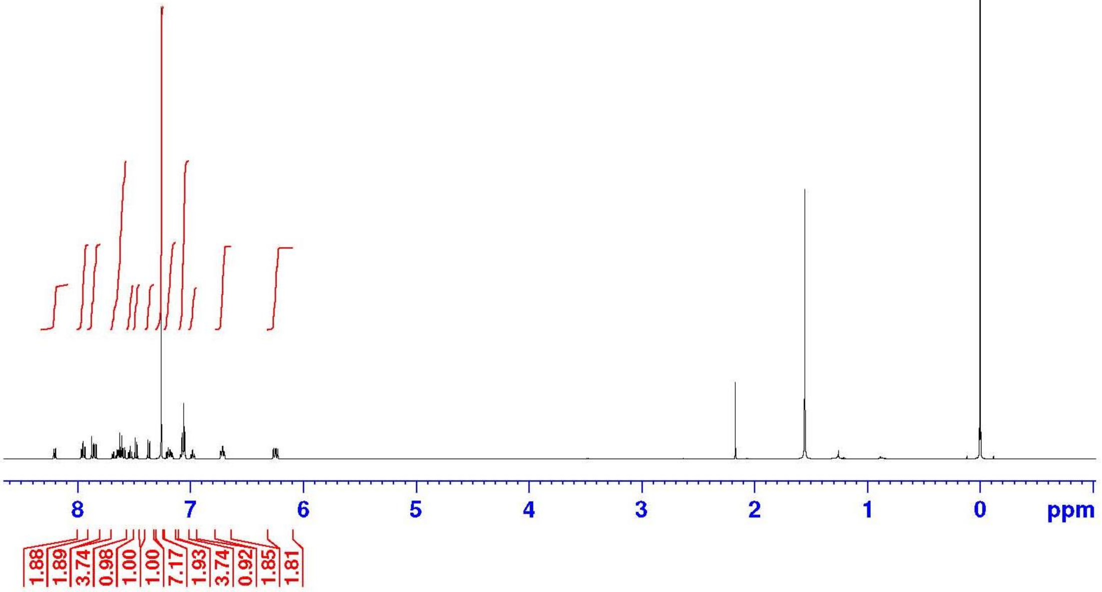

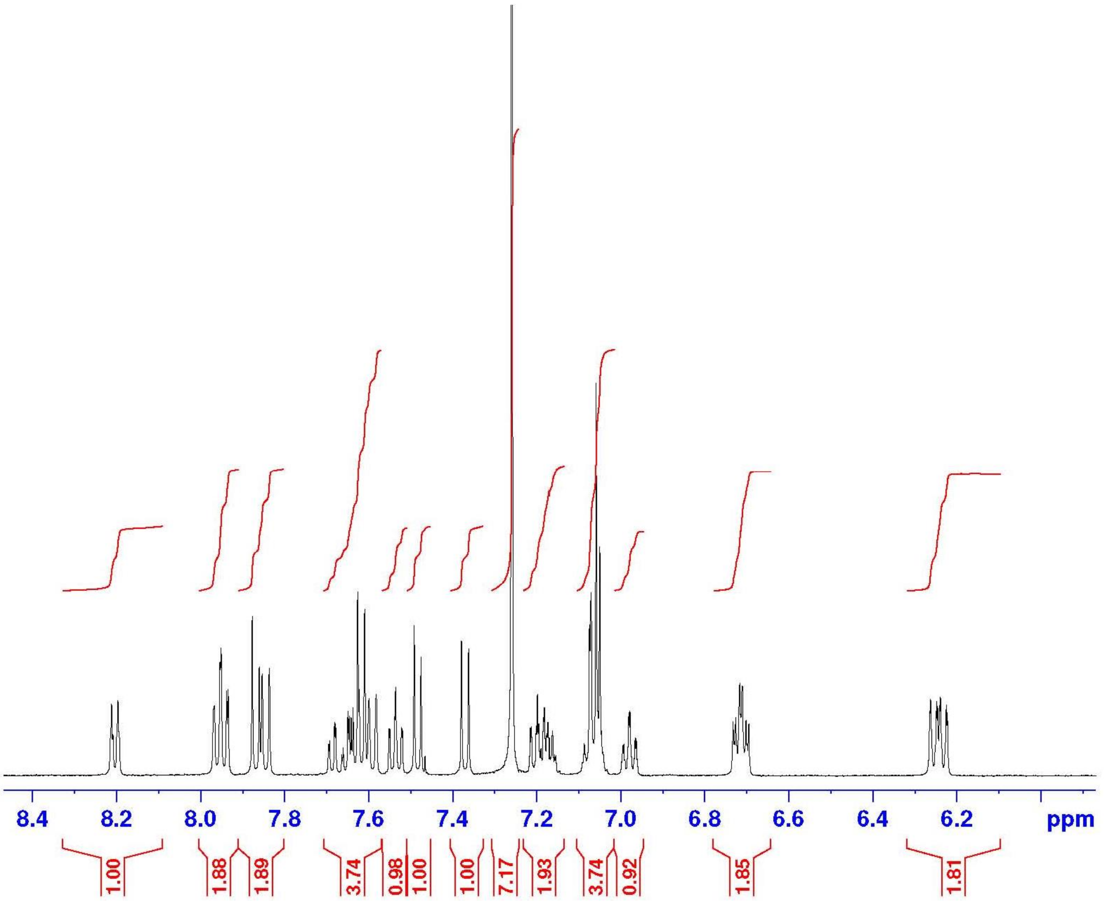

^{13}C NMR (125 MHz, $CDCl_3$) spectrum of compound **9**

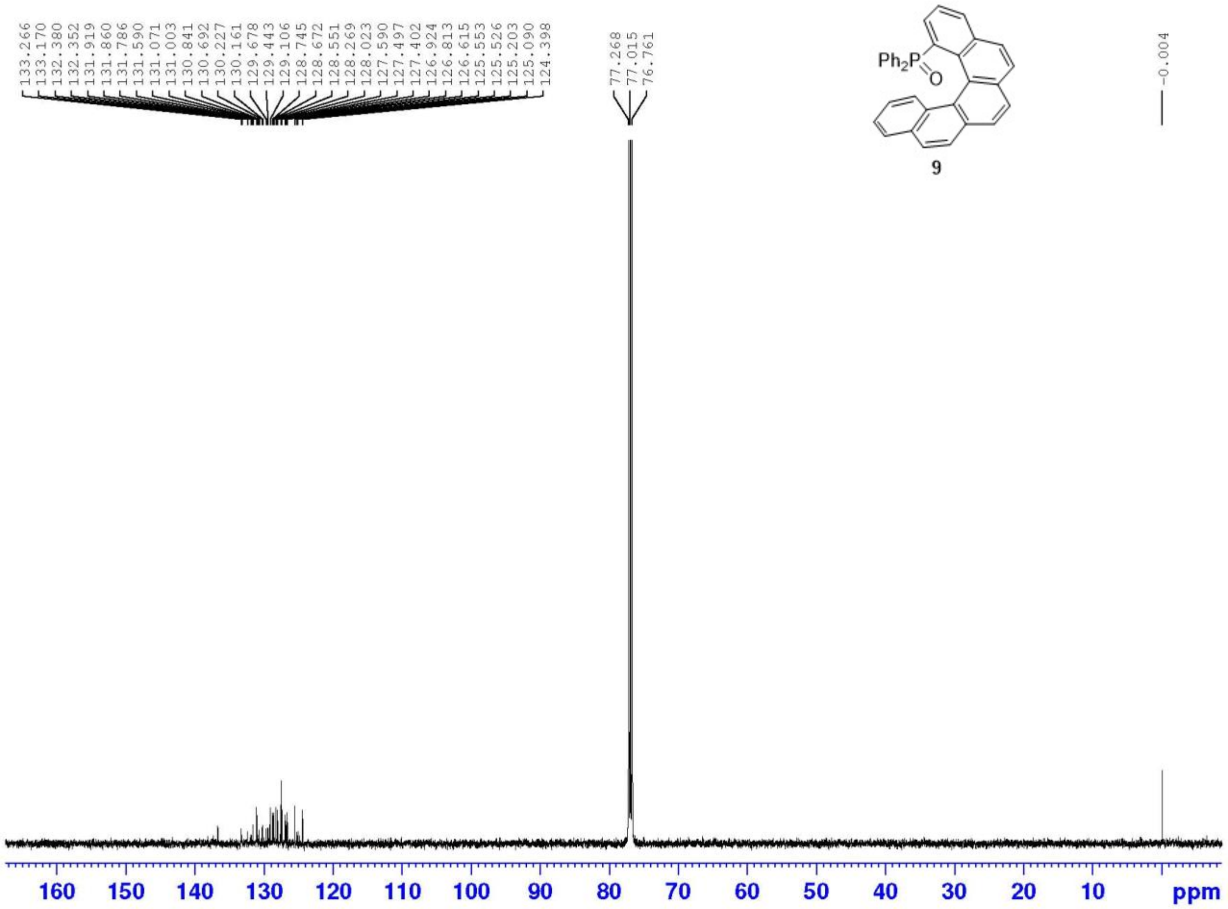

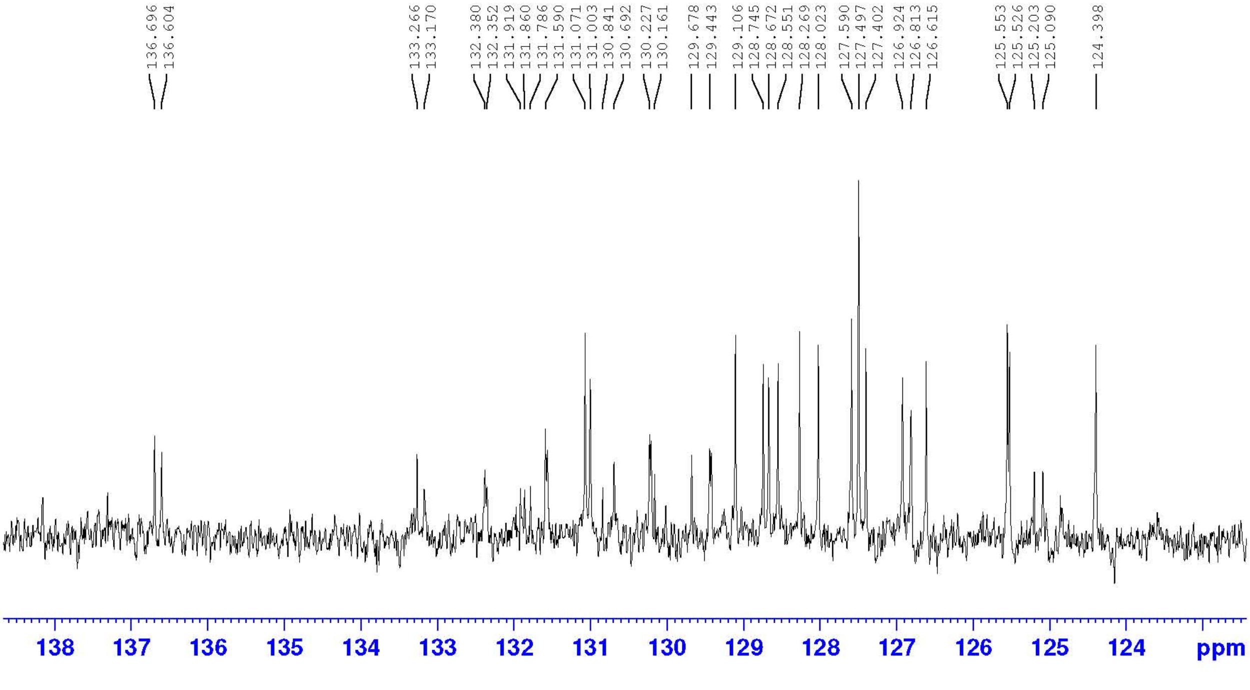

DEPT (125 MHz, $CDCl_3$) spectrum of compound **9**

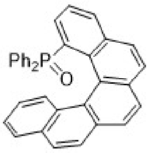

9

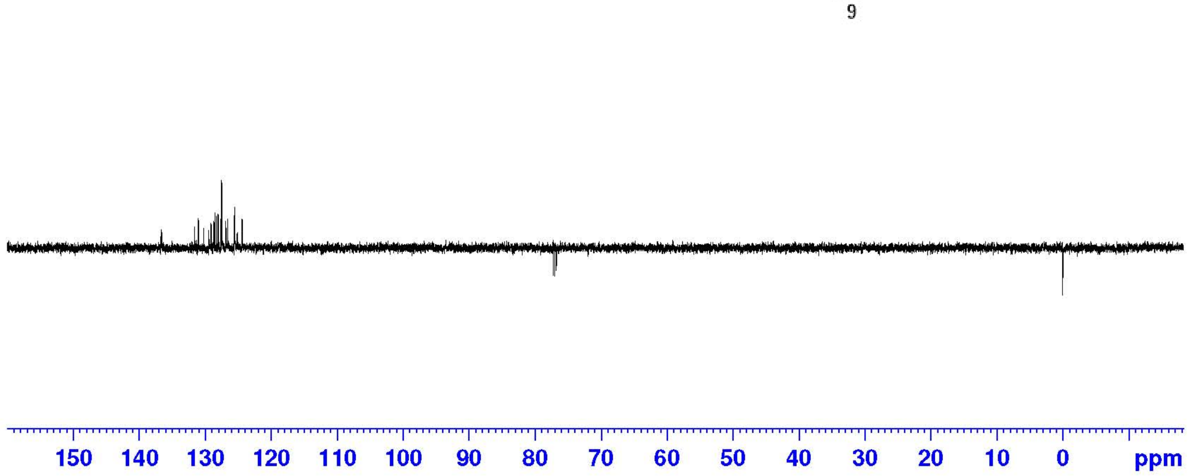

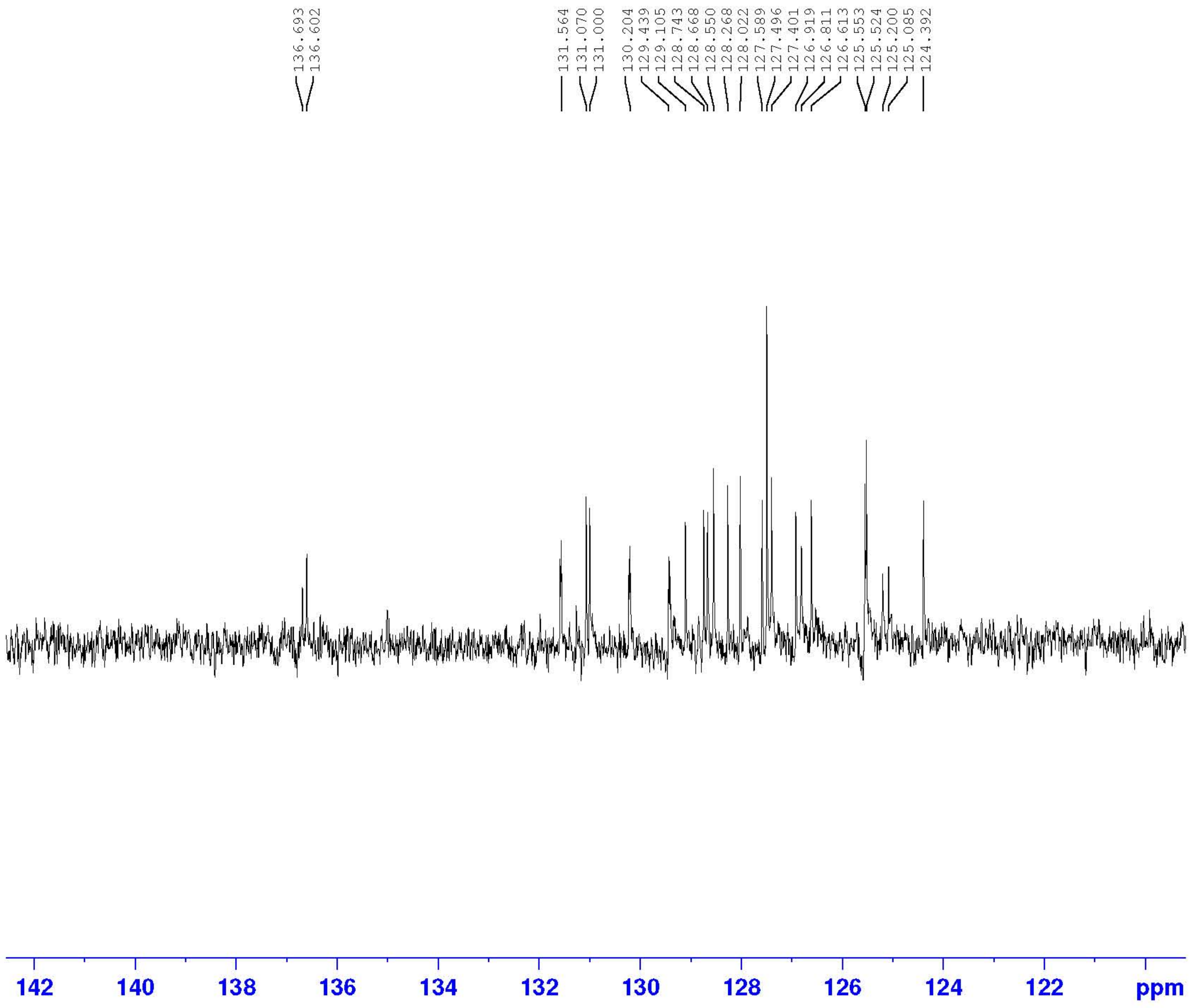

^{31}P NMR (202 MHz, $CDCl_3$) spectrum of compound **9**

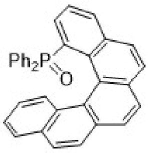

9

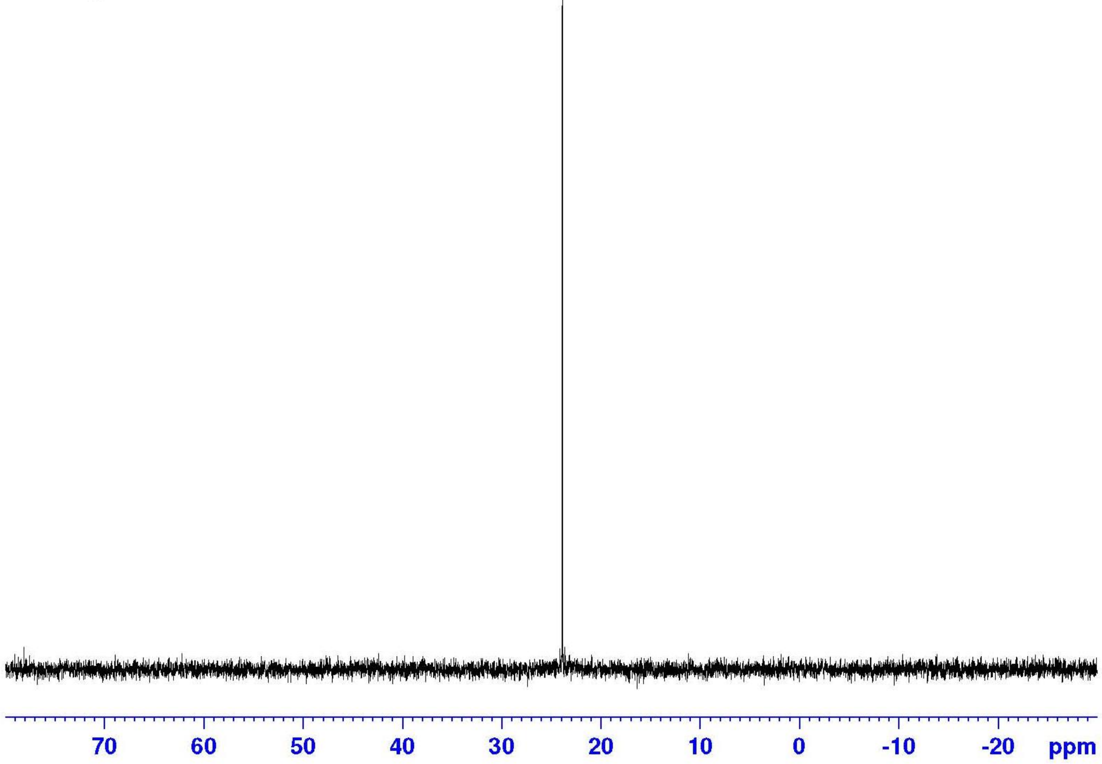

^{1}H NMR (500 MHz, $CDCl_3$) spectrum of compound **L2**

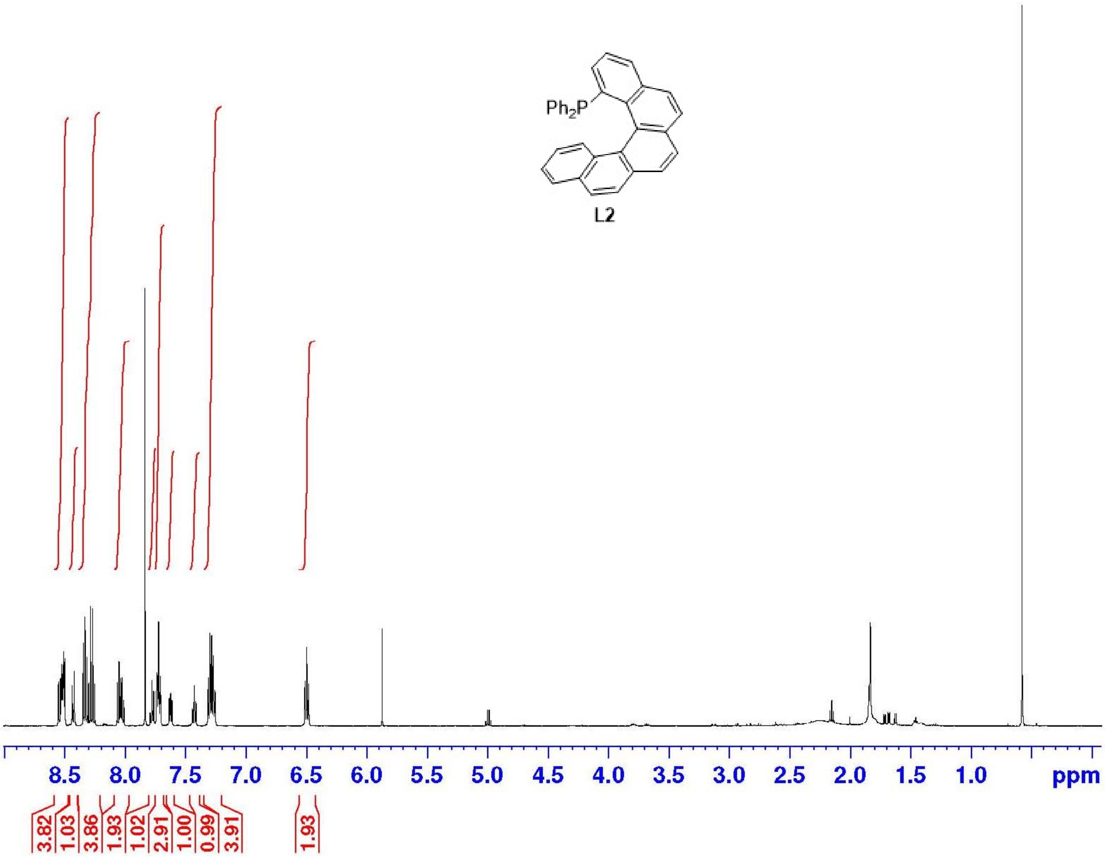

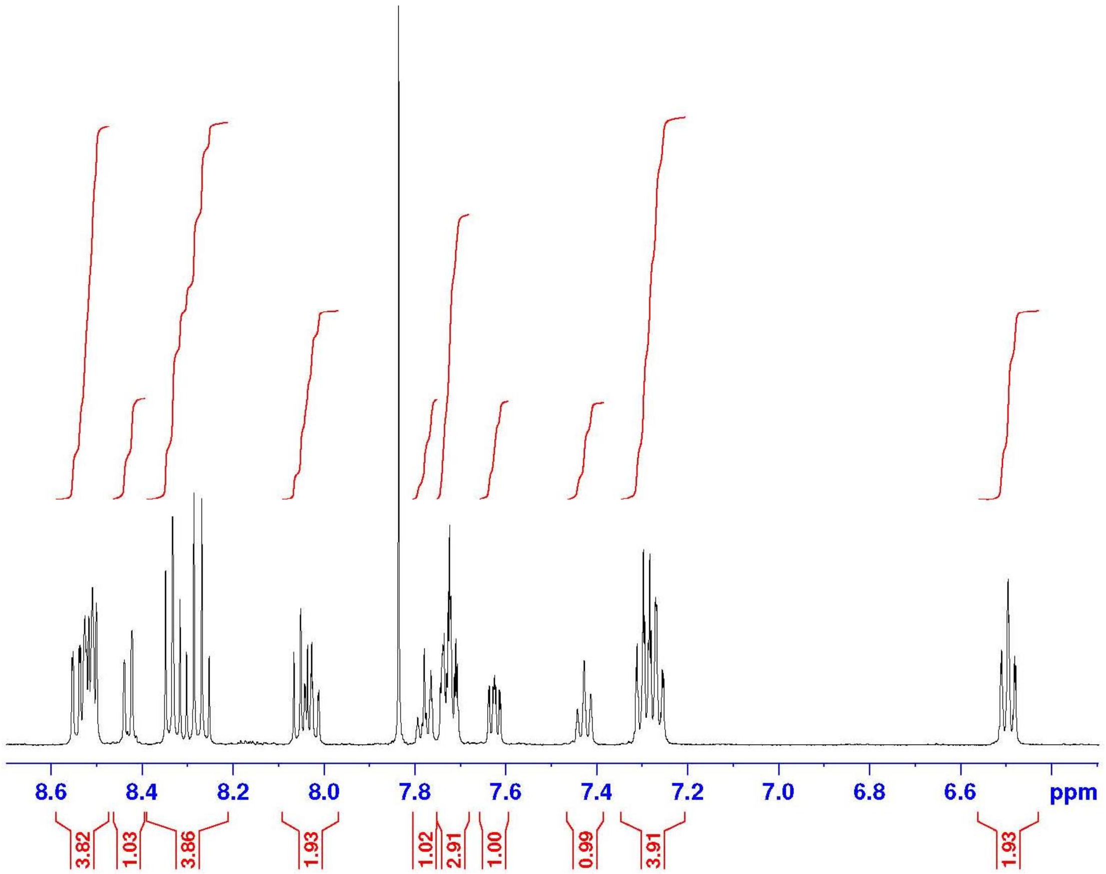

^{13}C NMR (125 MHz, $CDCl_3$) spectrum of compound **L2**

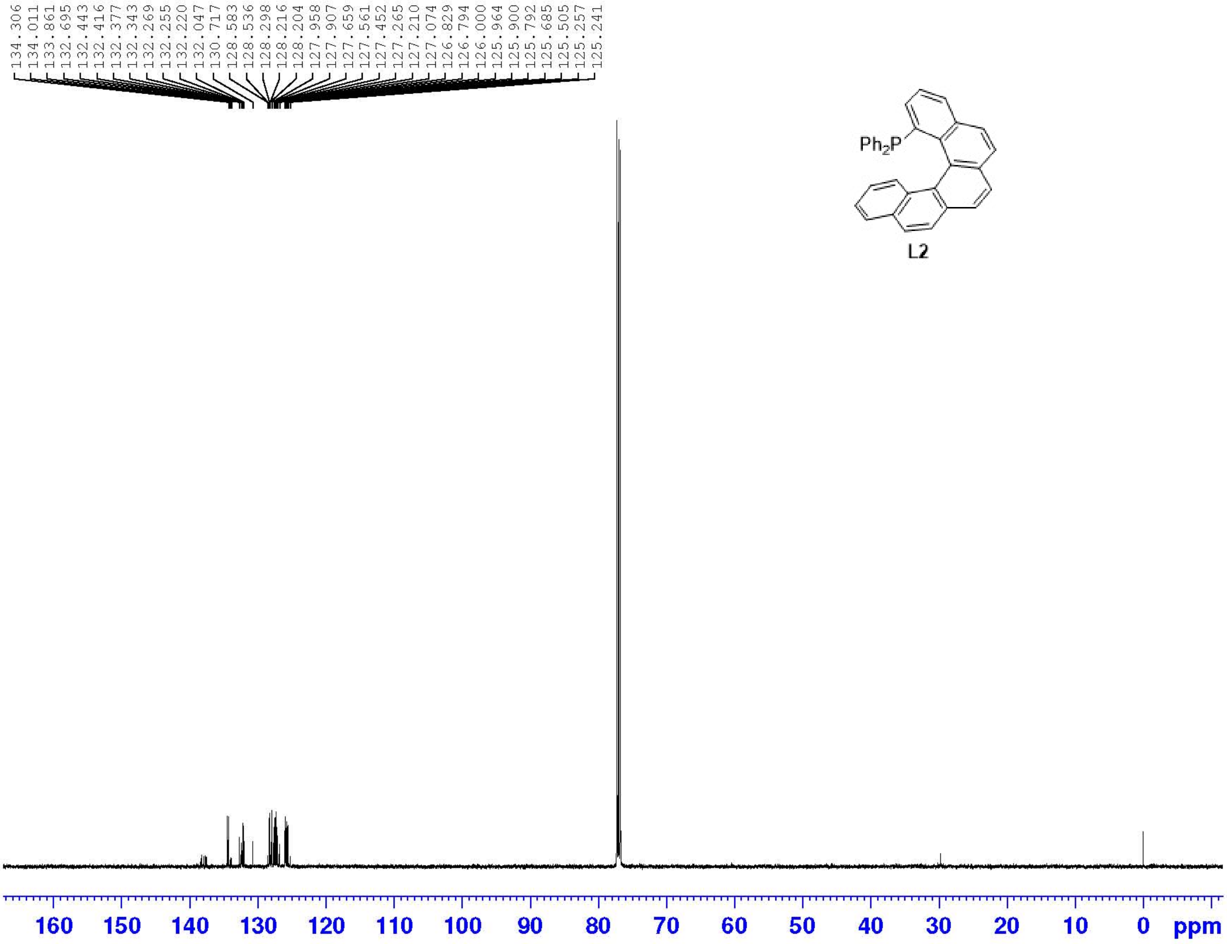

L2

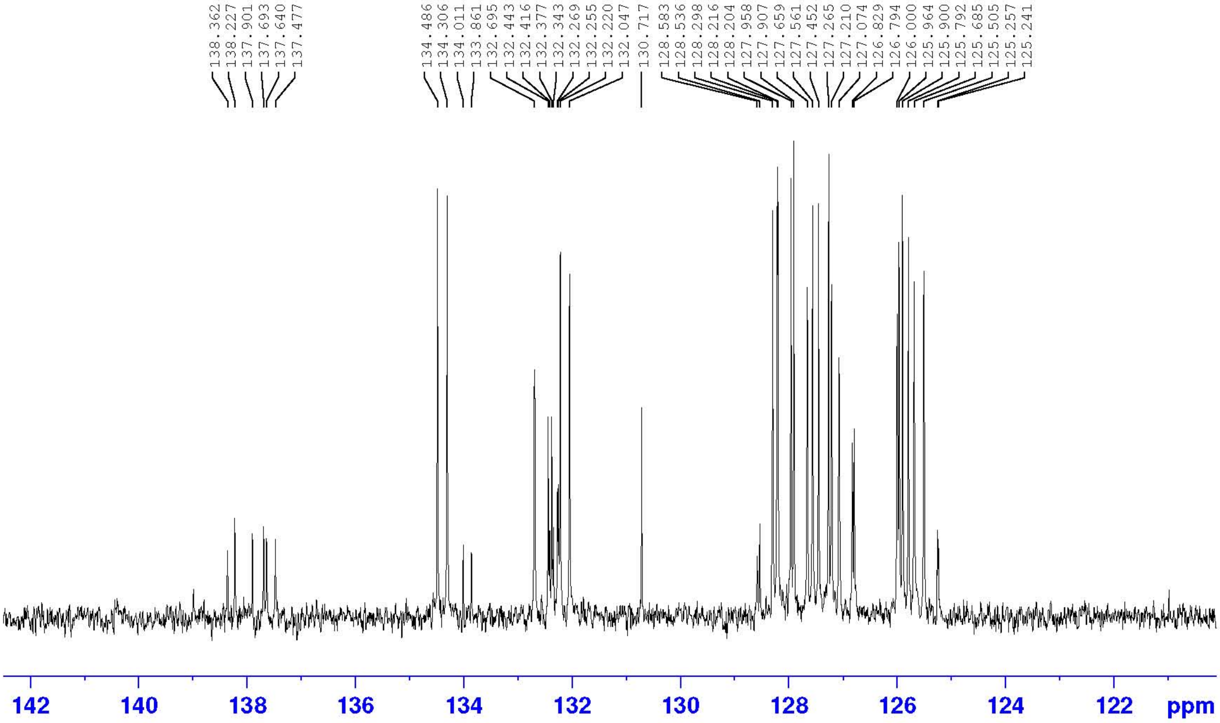

DEPT (125 MHz, $CDCl_3$) spectrum of compound **L2**

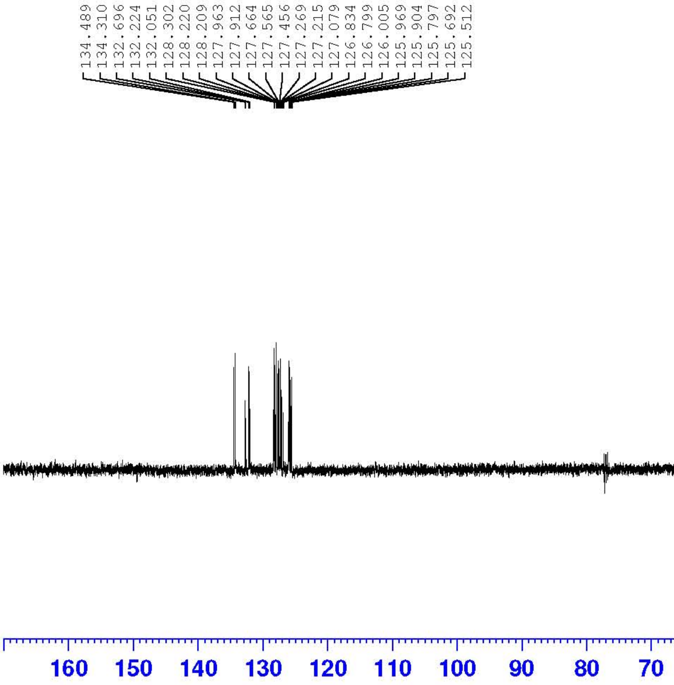

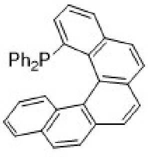

L2

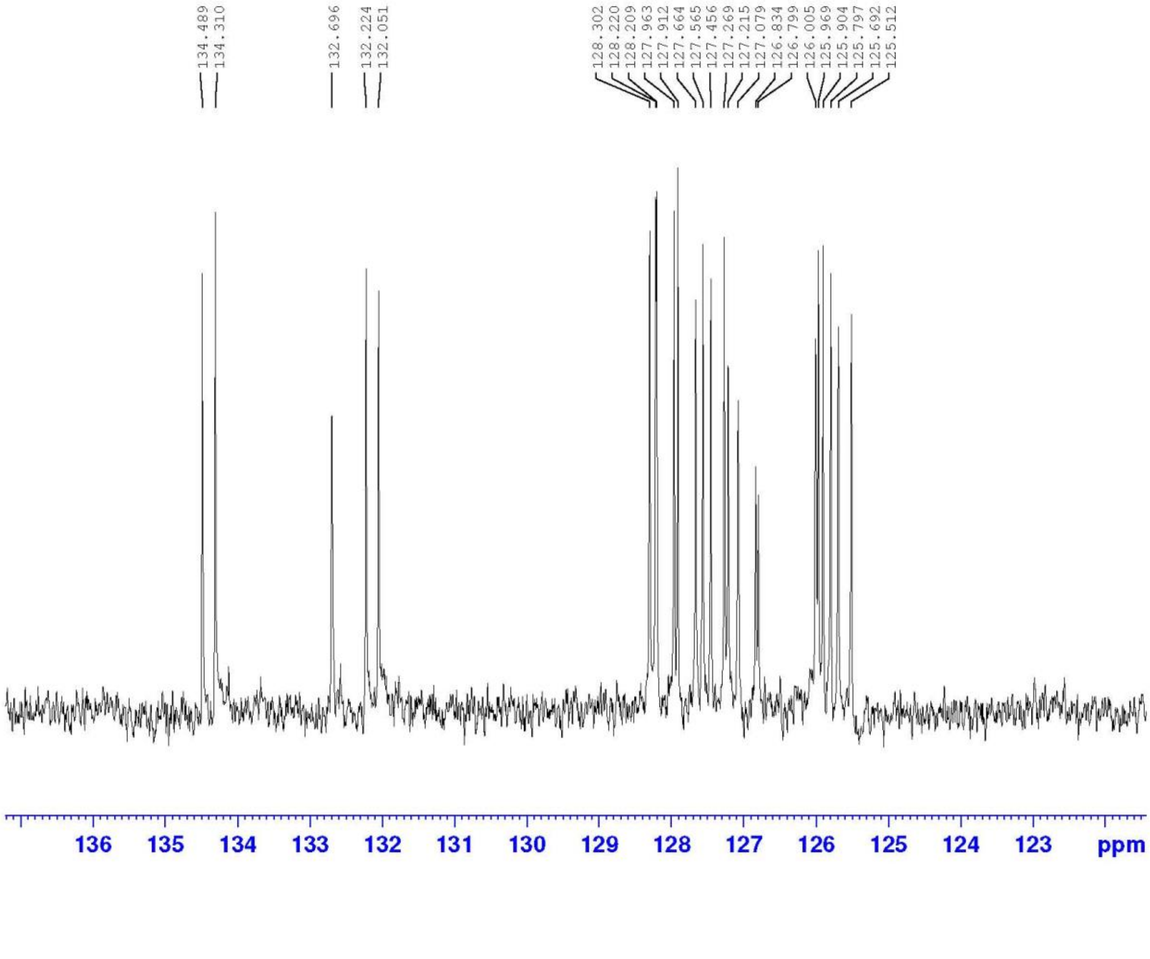

^{31}P NMR (202 MHz, $CDCl_3$) spectrum of compound **L2**

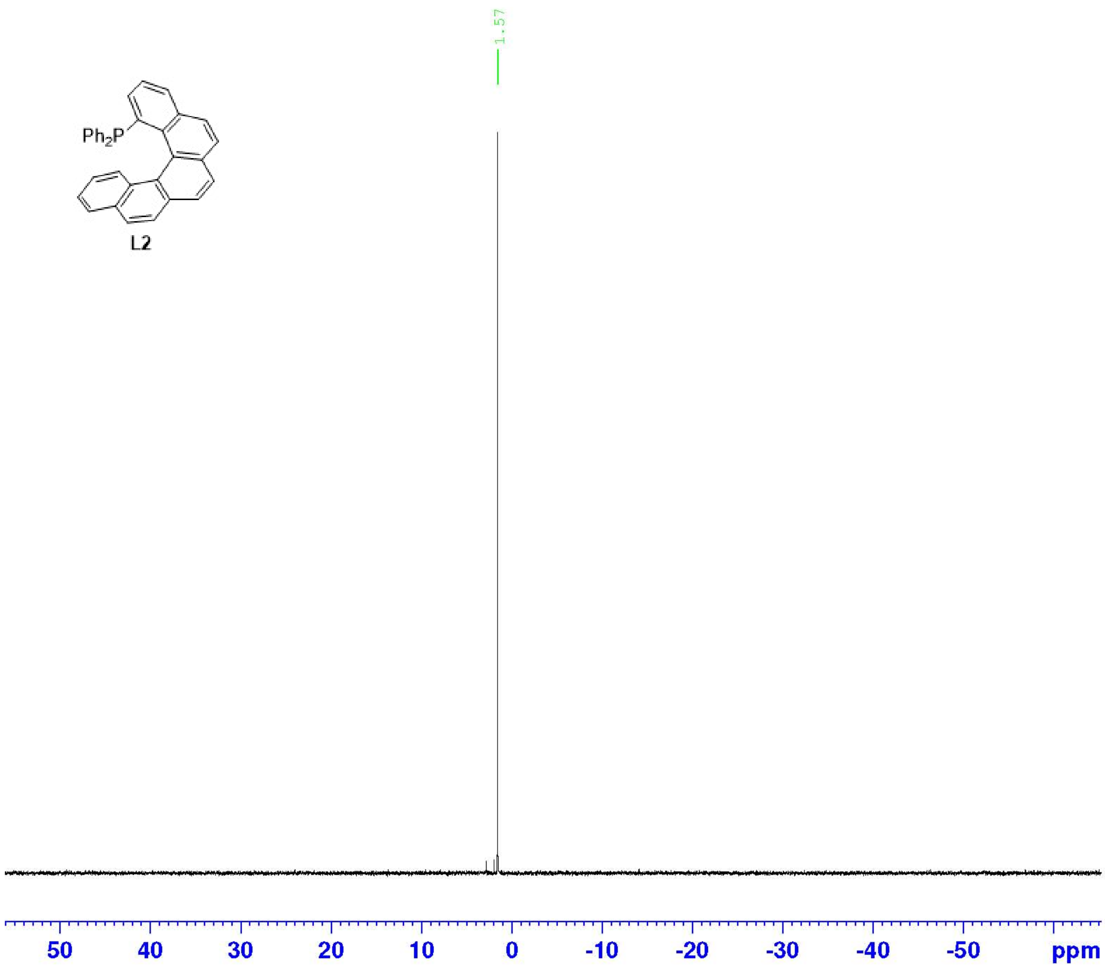

^1H NMR (400 MHz, $CDCl_3$) spectrum of compound **S1**

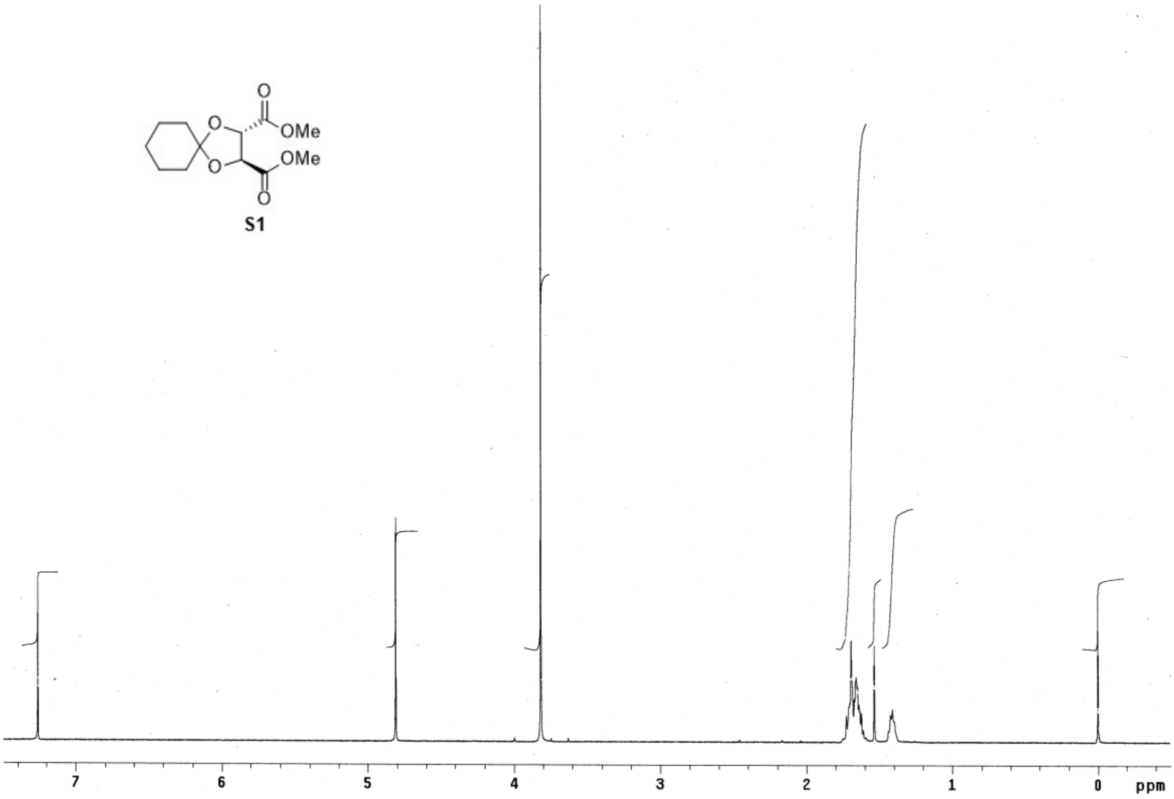

^1H NMR (400 MHz, $CDCl_3$) spectrum of compound **S2**

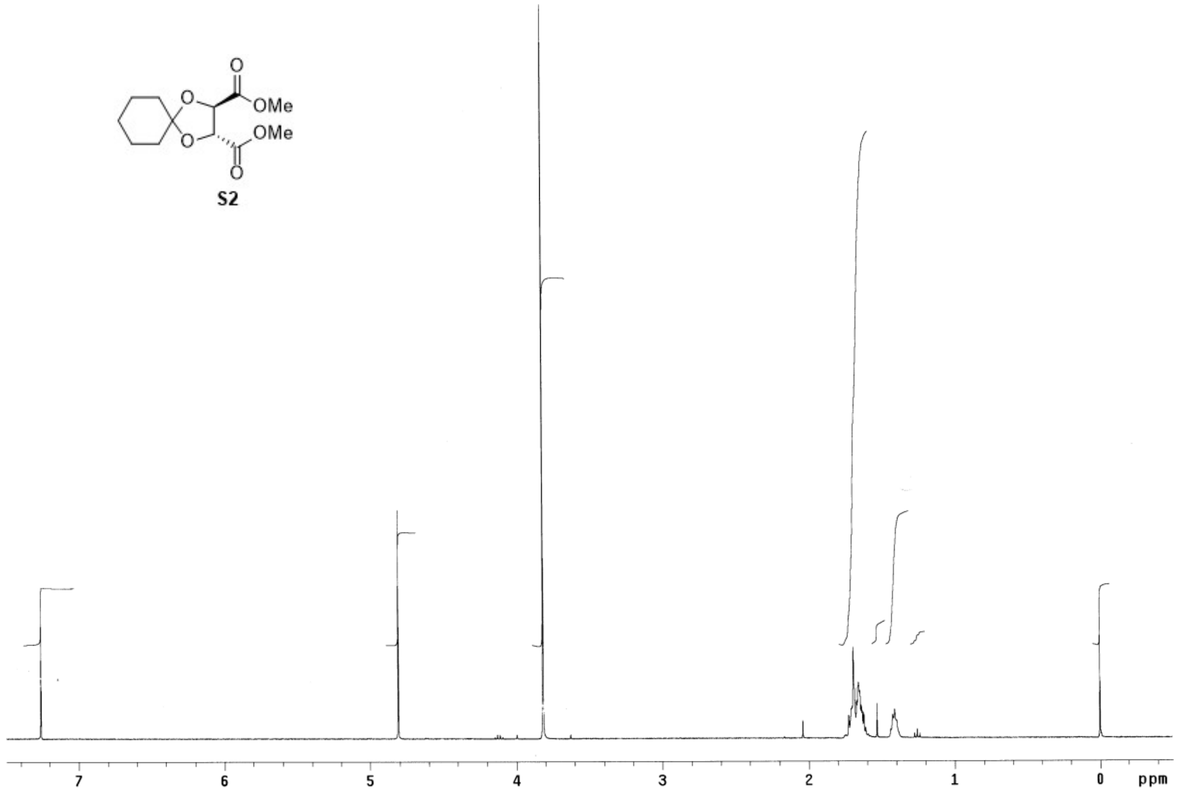

^{1}H NMR (500 MHz, $CDCl_3$) spectrum of compound (+)-**8**

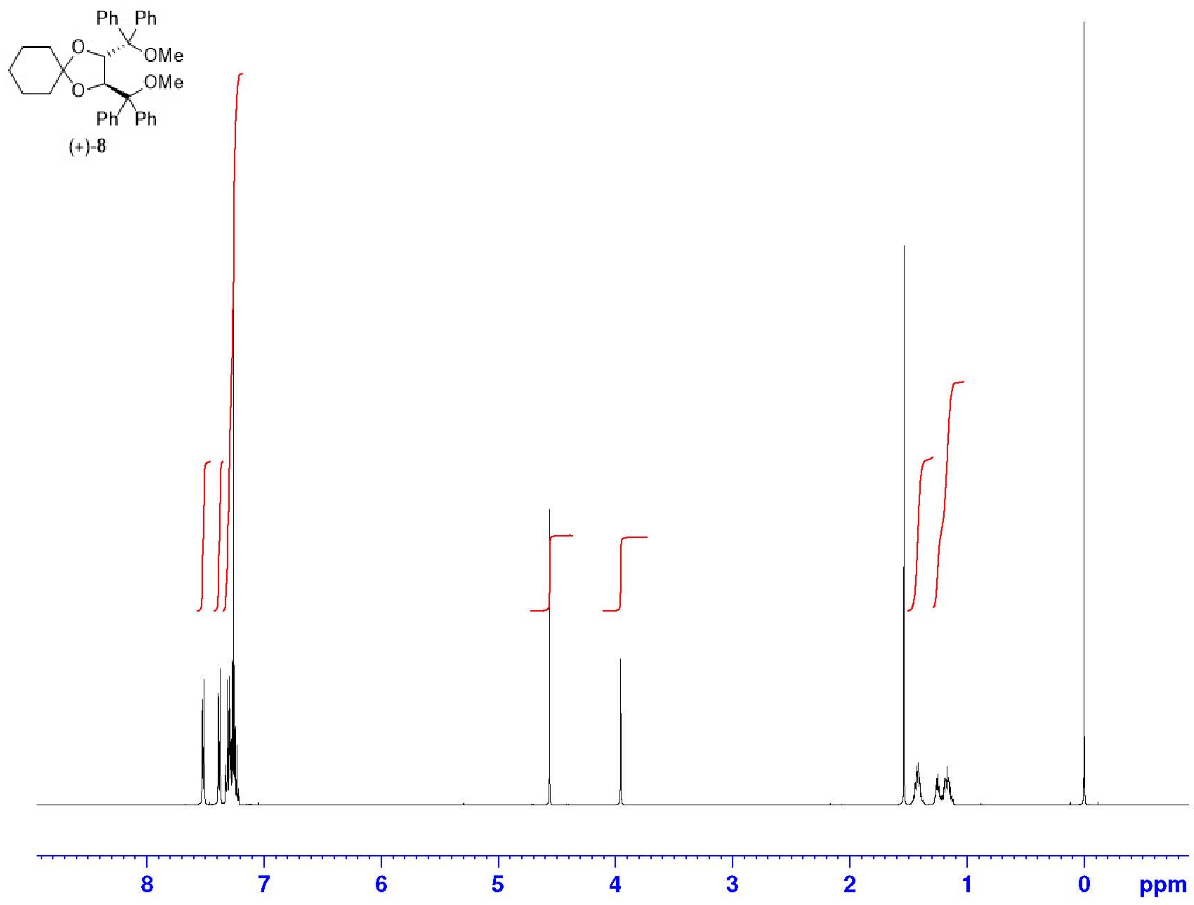

^{1}H NMR (400 MHz, $CDCl_3$) spectrum of compound (−)-**8**

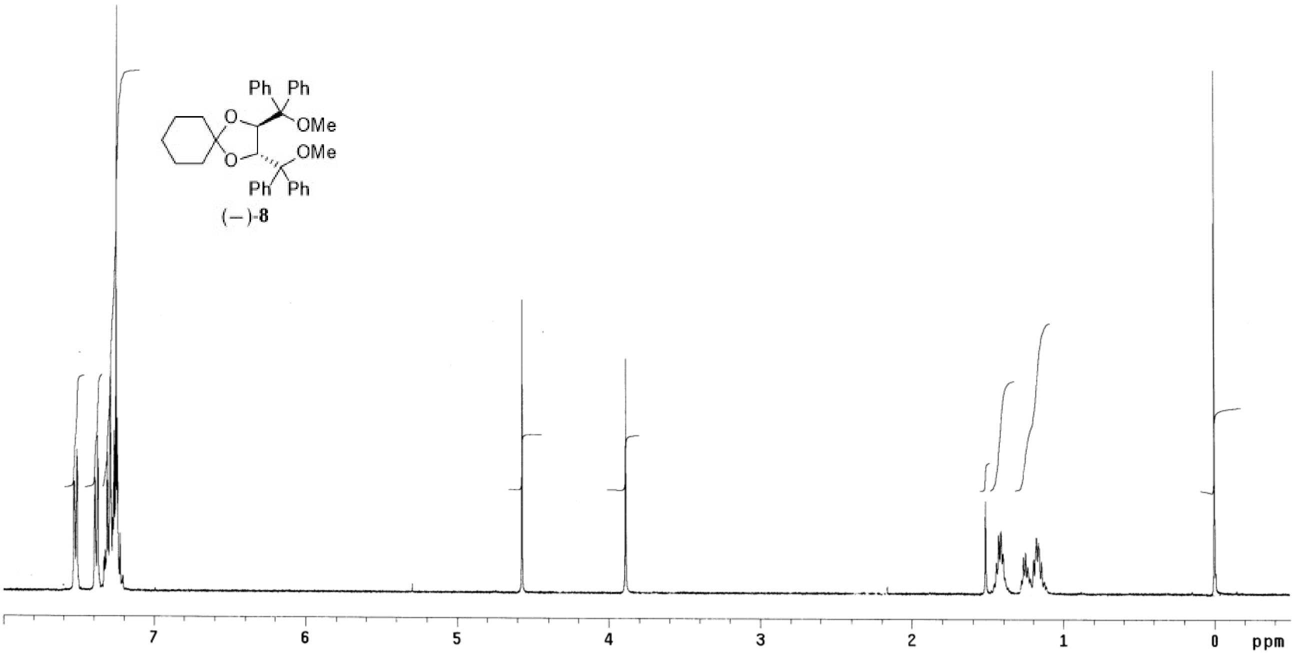

Supplement: Supplementary Information 2 [file srep36211-s2.pdf]
