# Supplementary material for: Rational Design and Synthesis of [5]Helicene-Derived Phosphine Ligands and Their Application in Pd-Catalyzed Asymmetric Reactions
Source: Sci Rep. 2016 Nov 8;6:36211. doi: 10.1038/srep36211 (PMC5099951; doi:10.1038/srep36211)

Copies of NMR spectra

^{1}H NMR (500 MHz, $CDCl_3$) spectrum of compound **12**

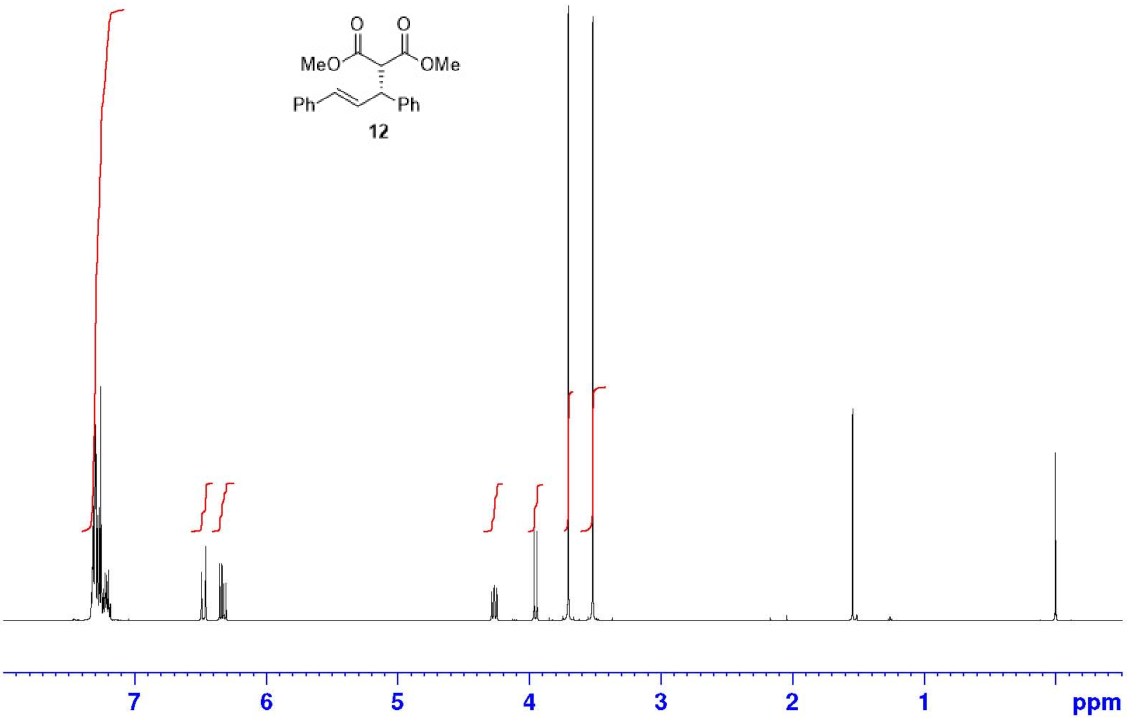

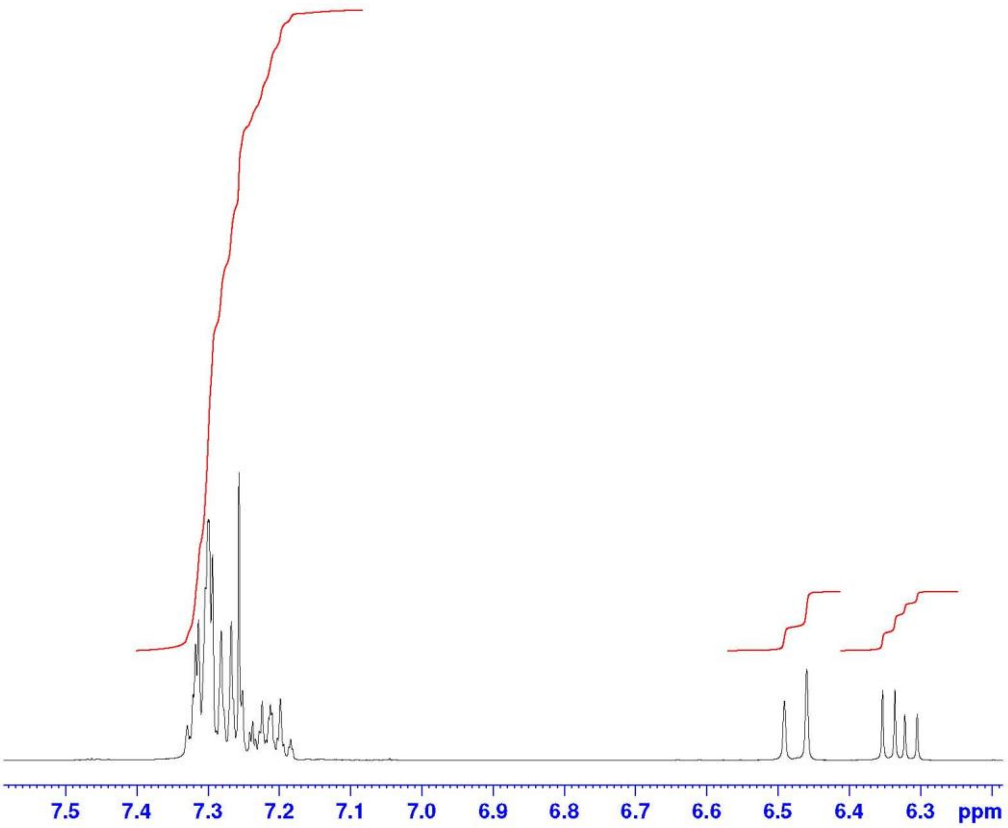

^{1}H NMR (500 MHz, $CDCl_3$) spectrum of compound **13a**

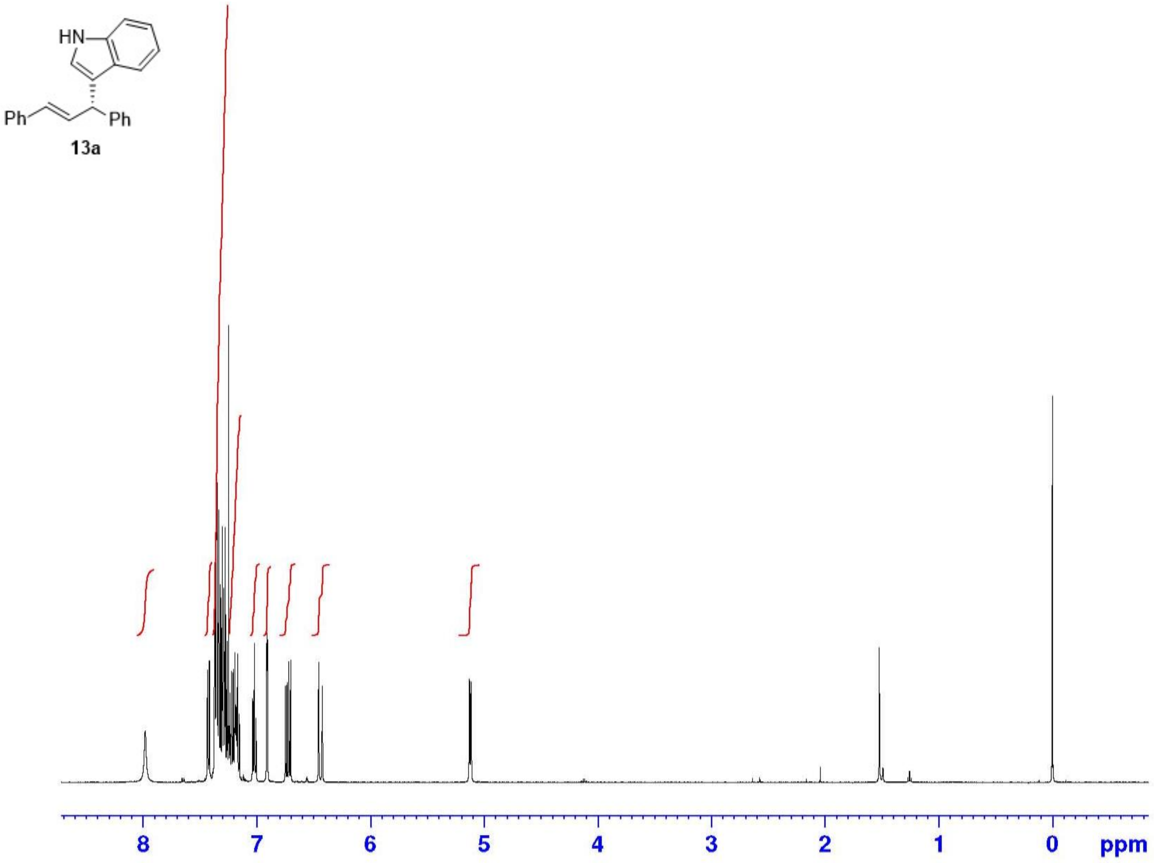

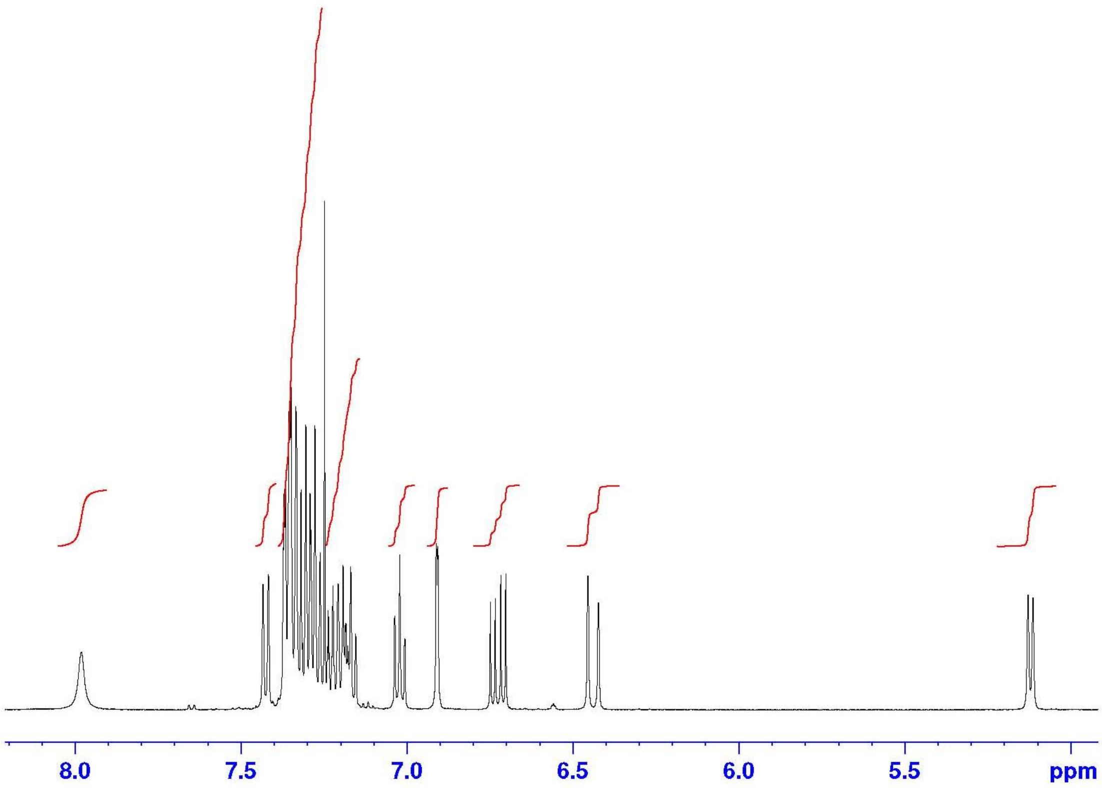

^{1}H NMR (500 MHz, $CDCl_3$) spectrum of compound **13b**

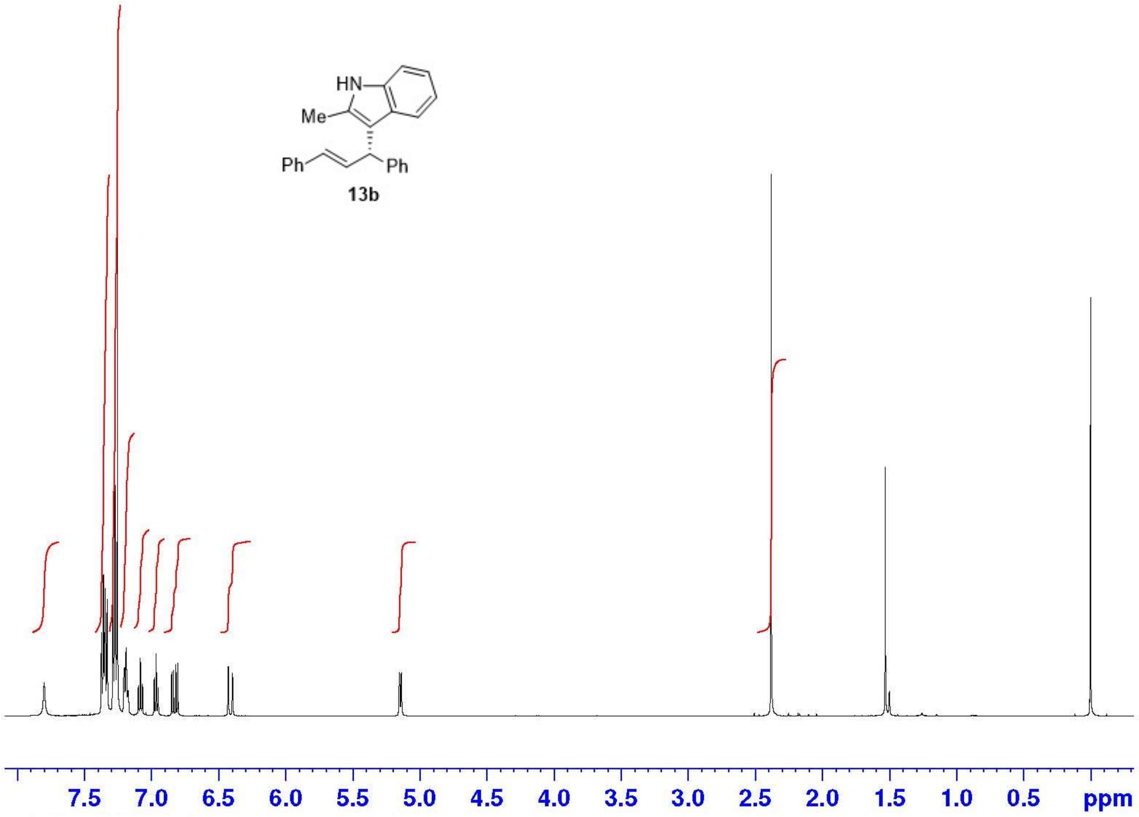

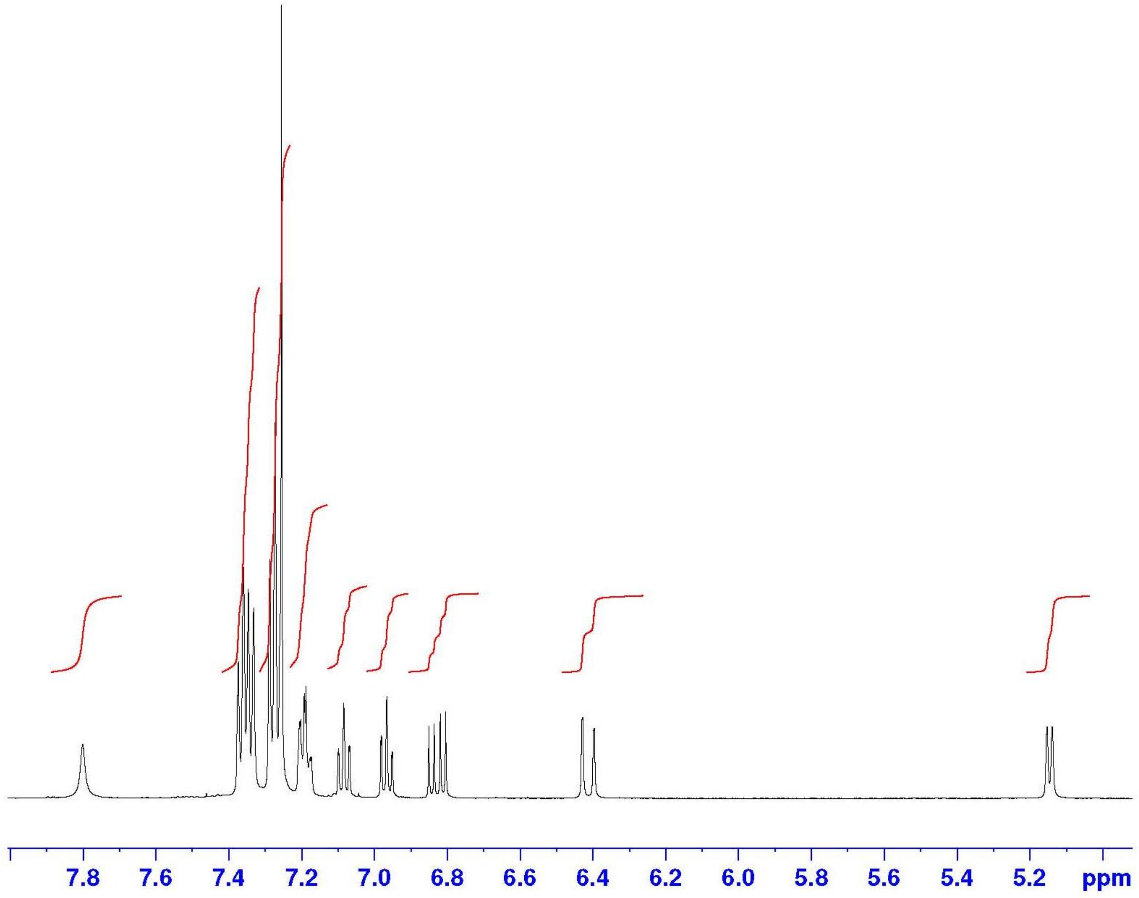

^{1}H NMR (500 MHz, $CDCl_3$) spectrum of compound **13c**

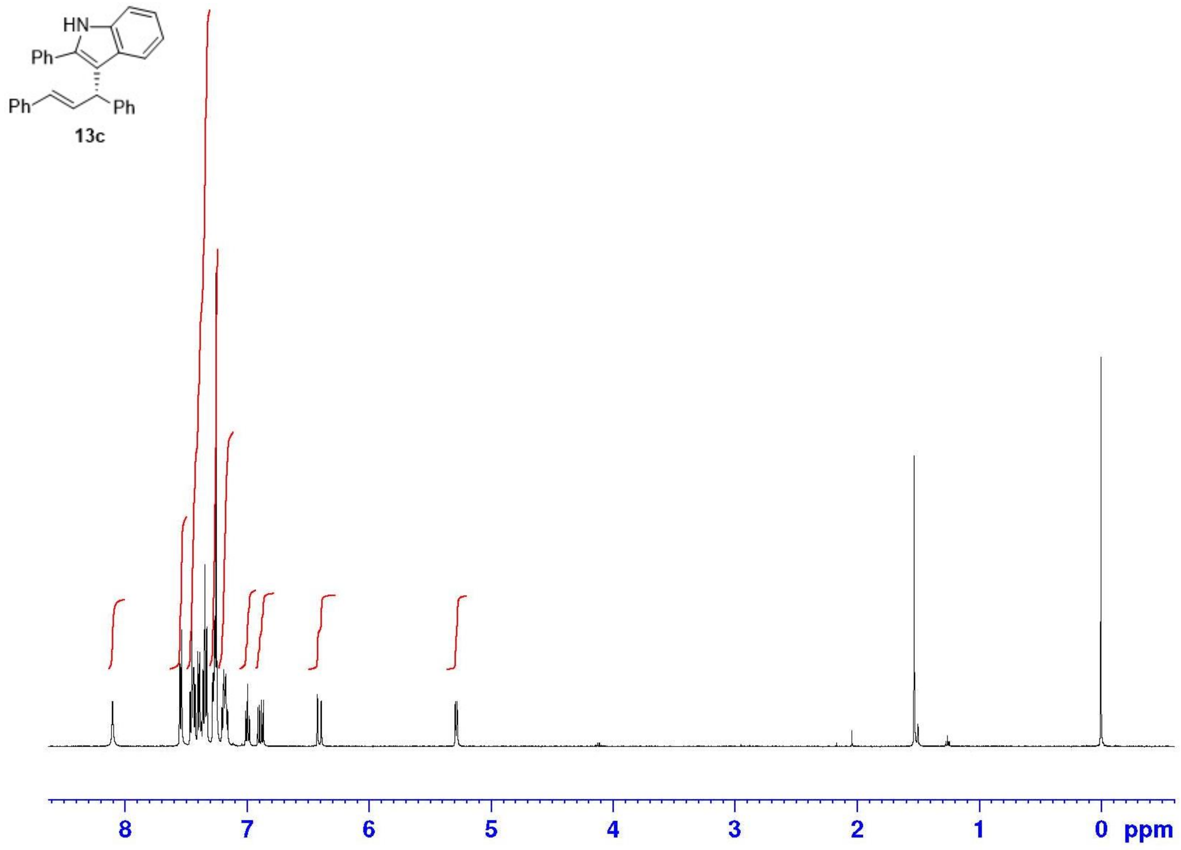

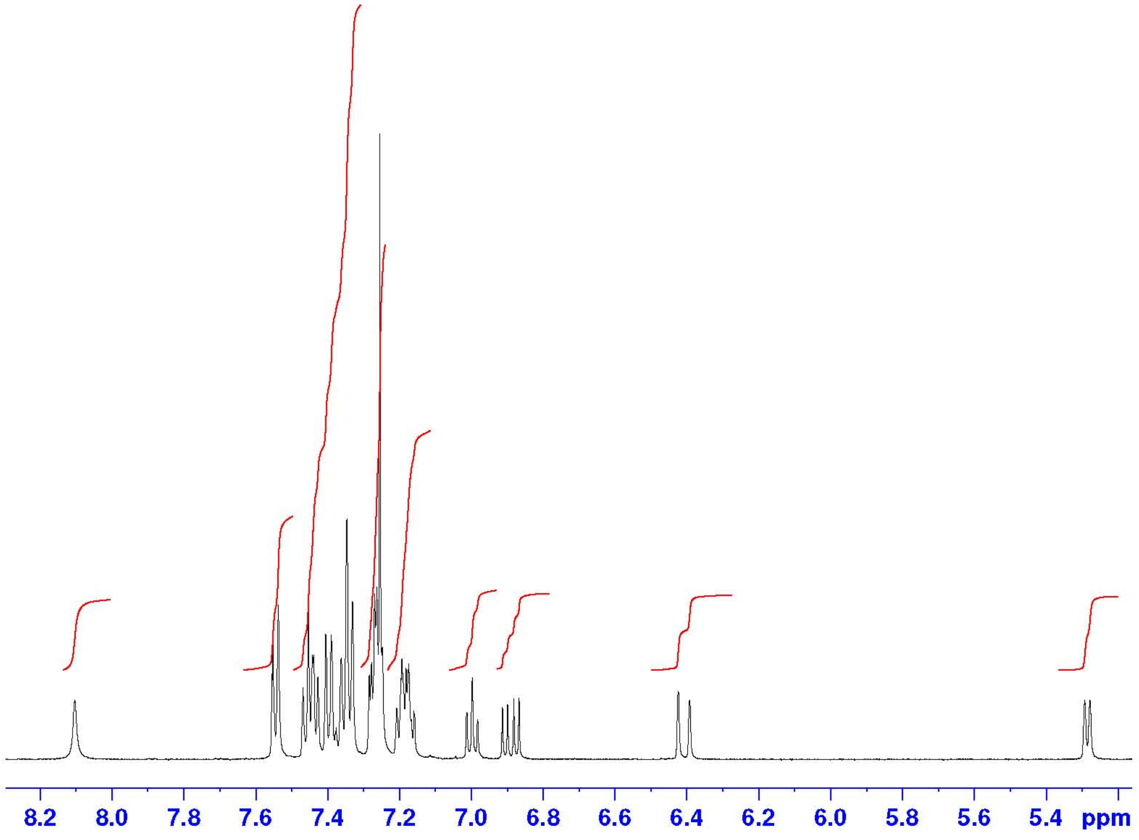

^{1}H NMR (500 MHz, $CDCl_3$) spectrum of compound **13d**

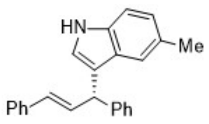

13d

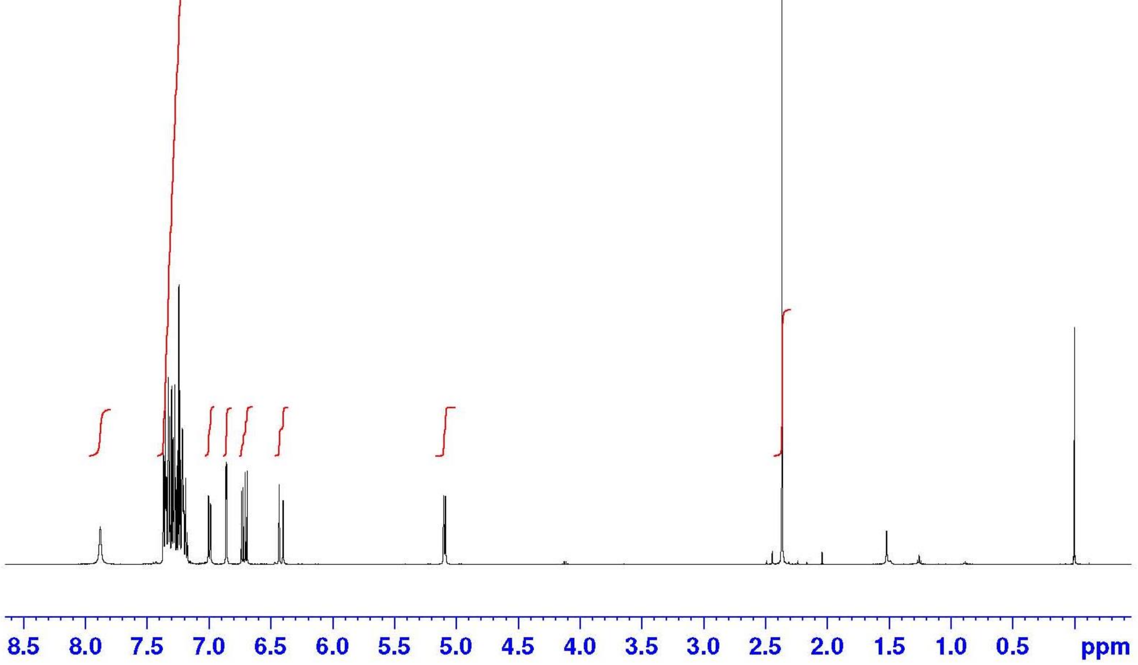

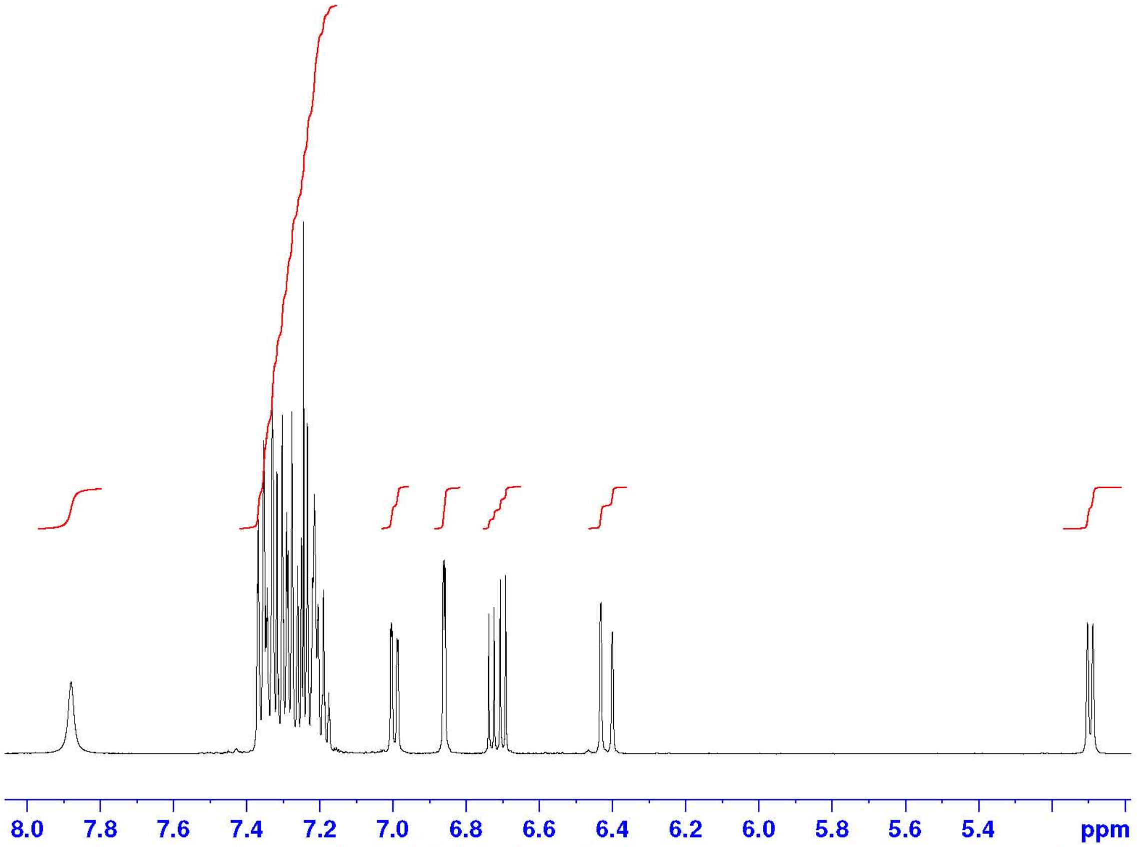

^{1}H NMR (500 MHz, $CDCl_3$) spectrum of compound **13e**

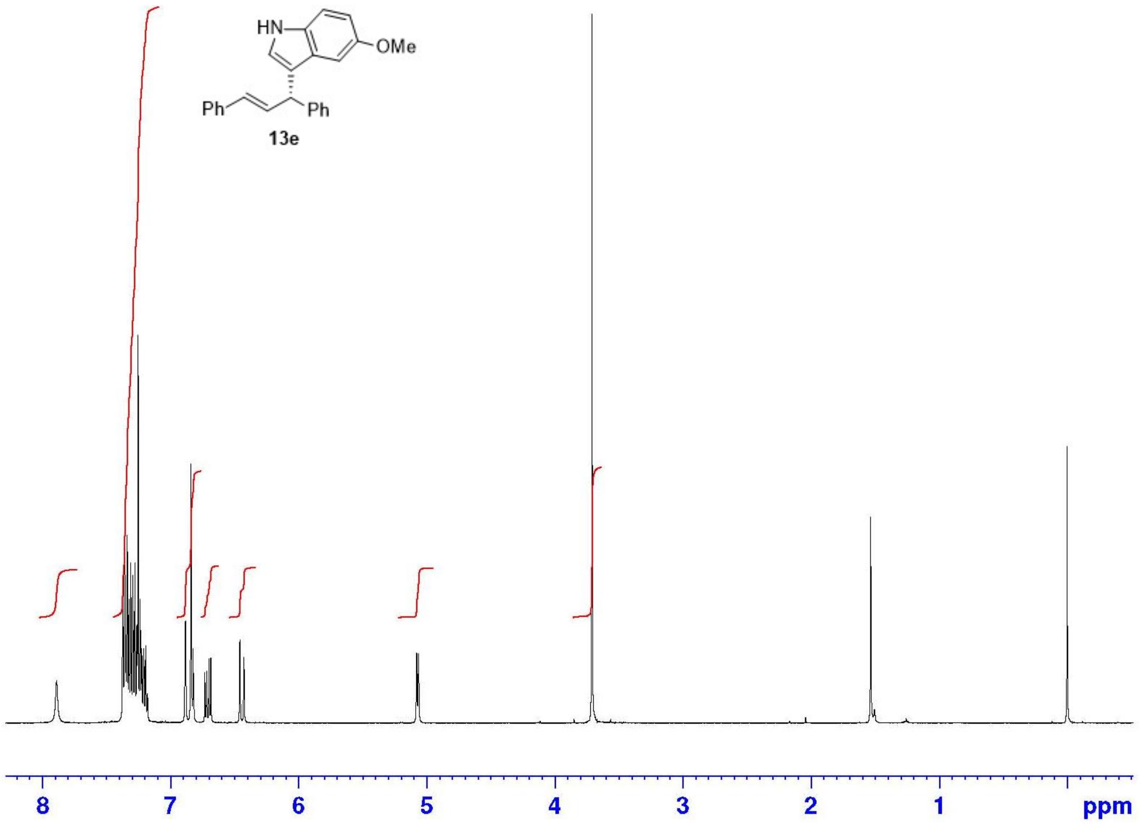

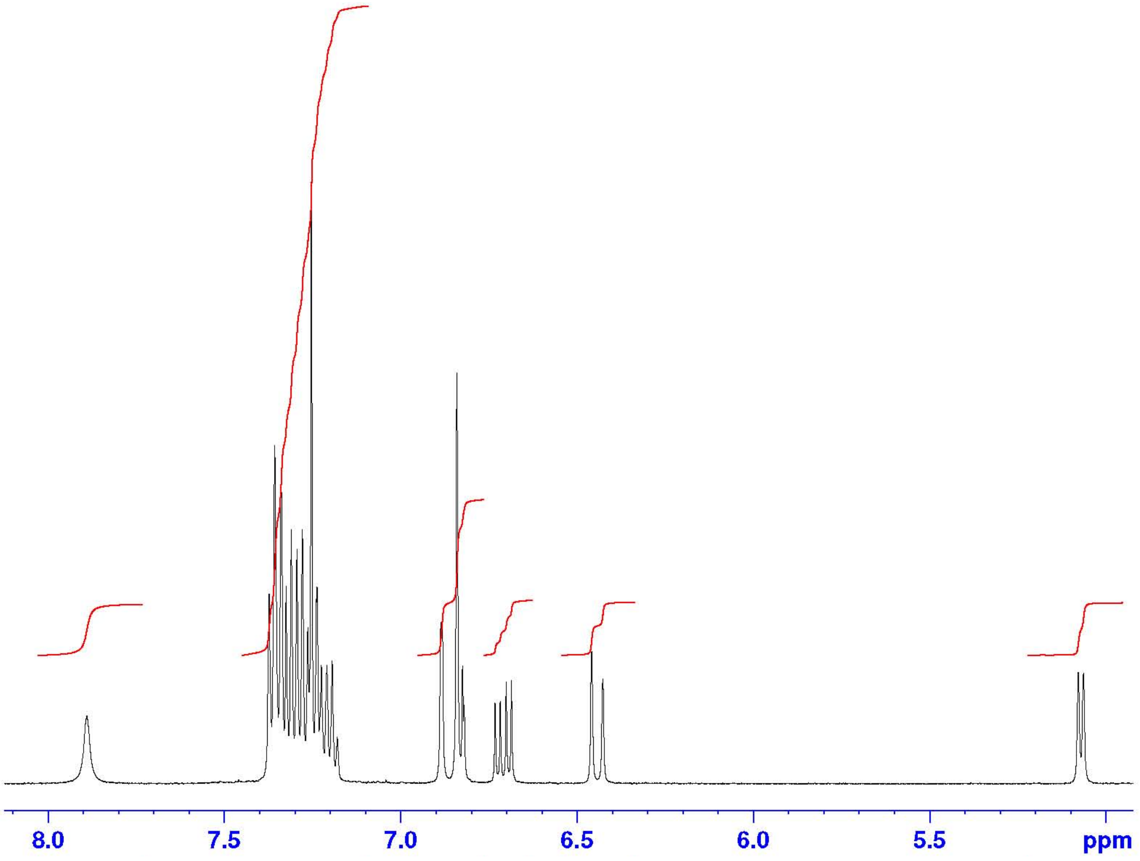

^{1}H NMR (500 MHz, $CDCl_3$) spectrum of compound **13f**

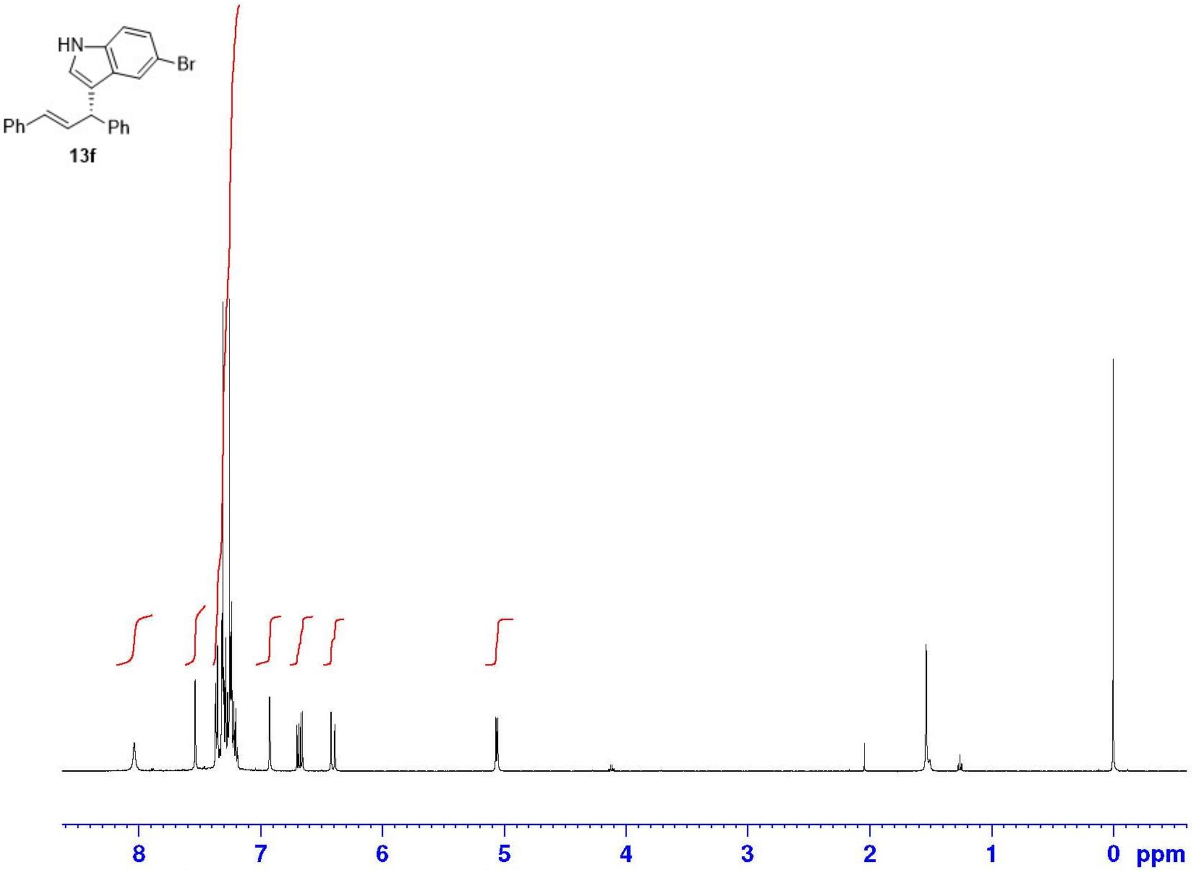

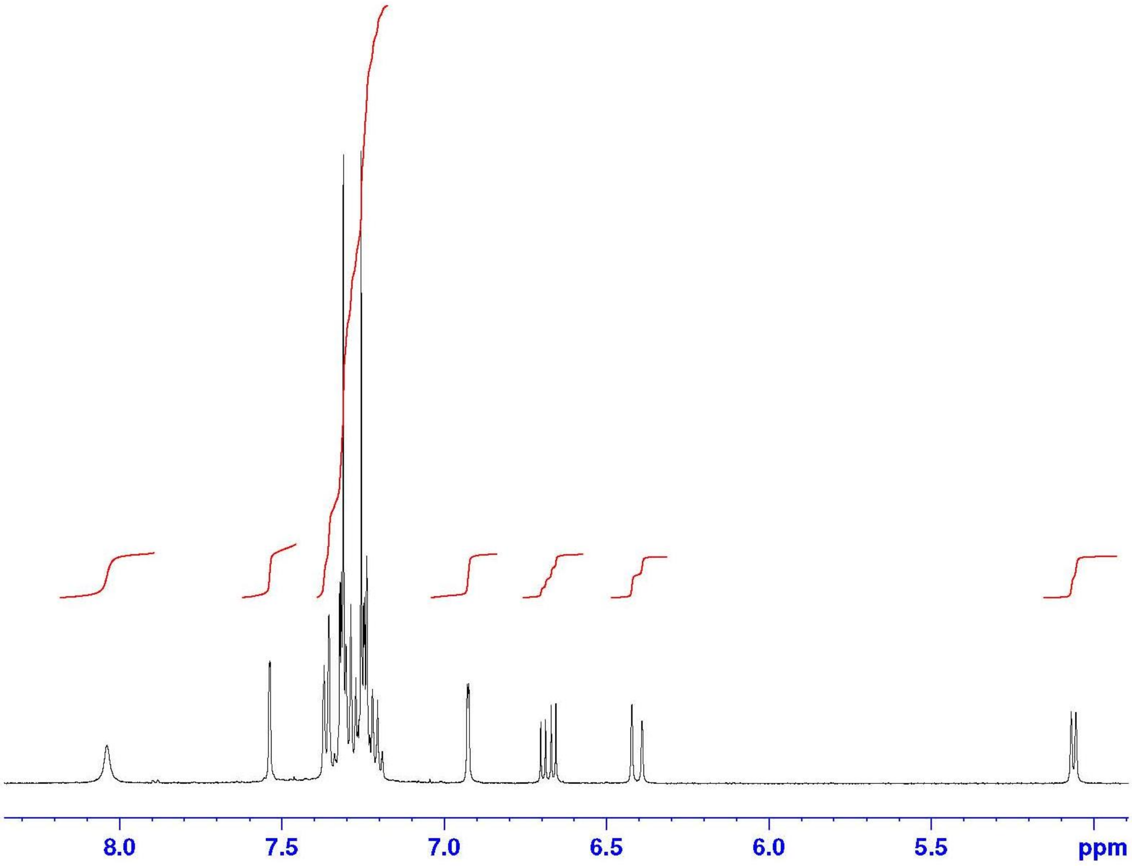

^{1}H NMR (500 MHz, $CDCl_3$) spectrum of compound **13g**

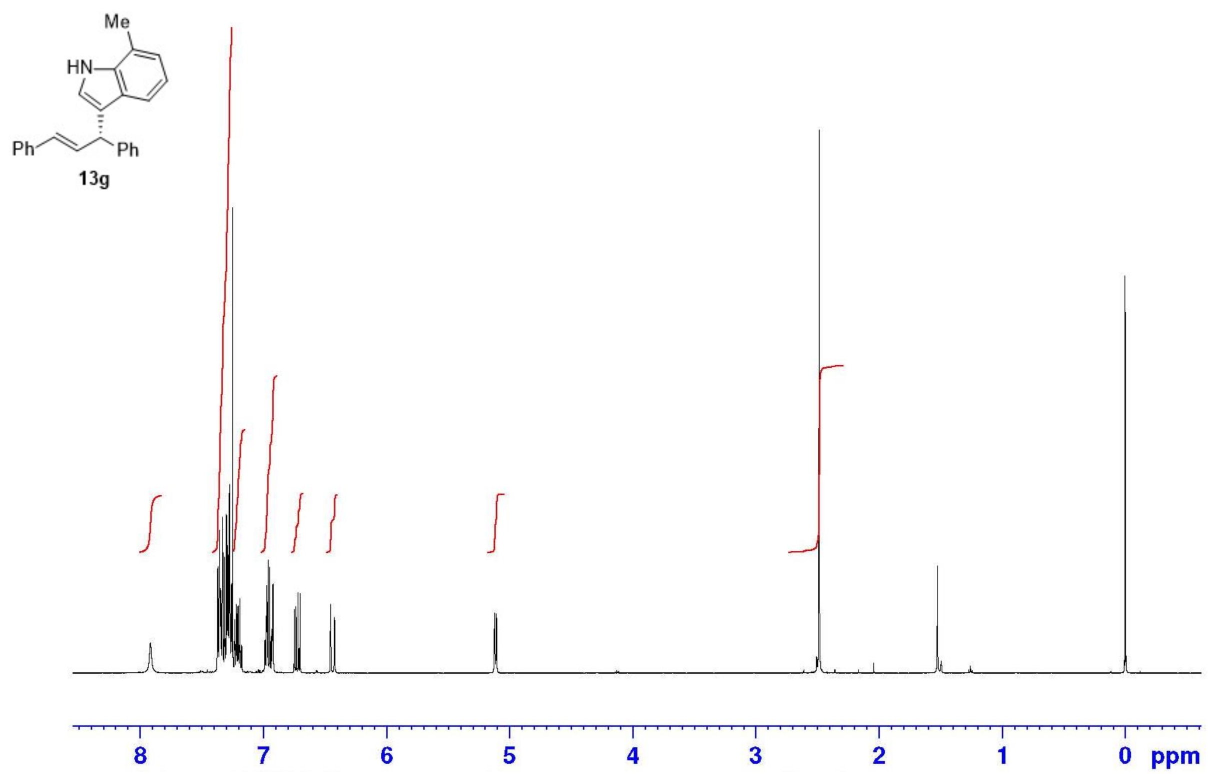

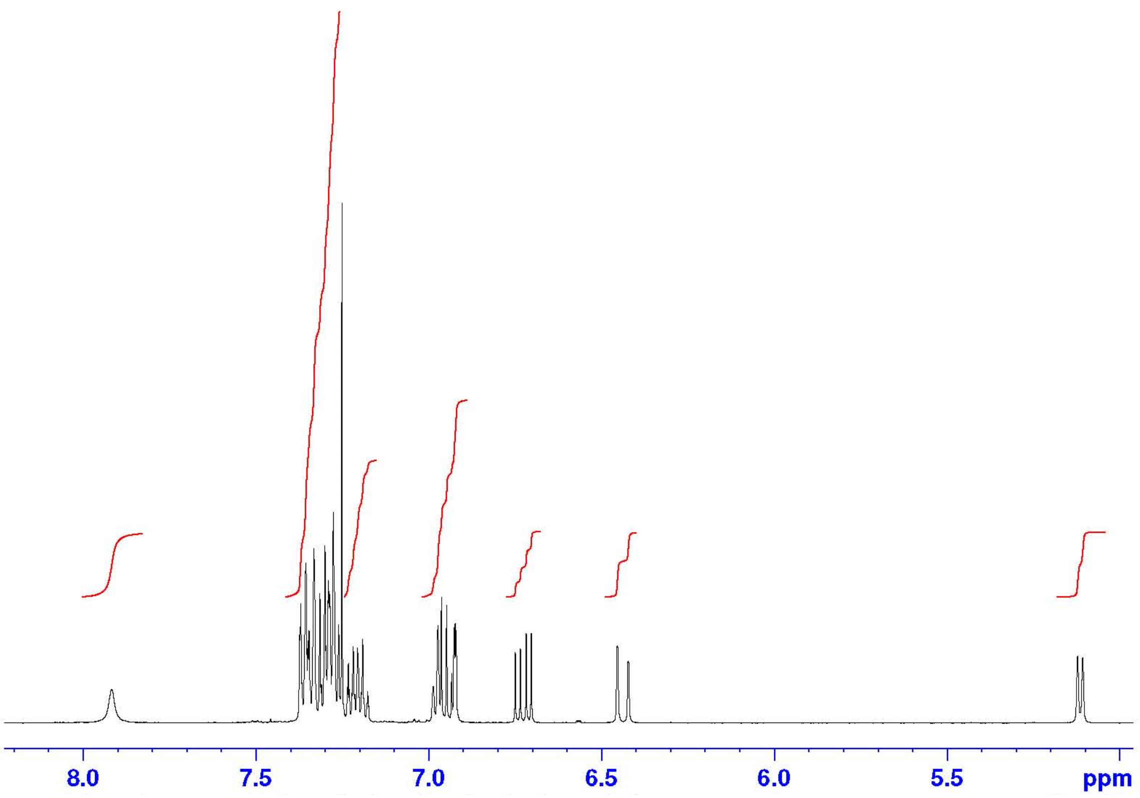

^{1}H NMR (500 MHz, $CDCl_3$) spectrum of compound **14a**

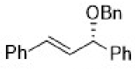

14a

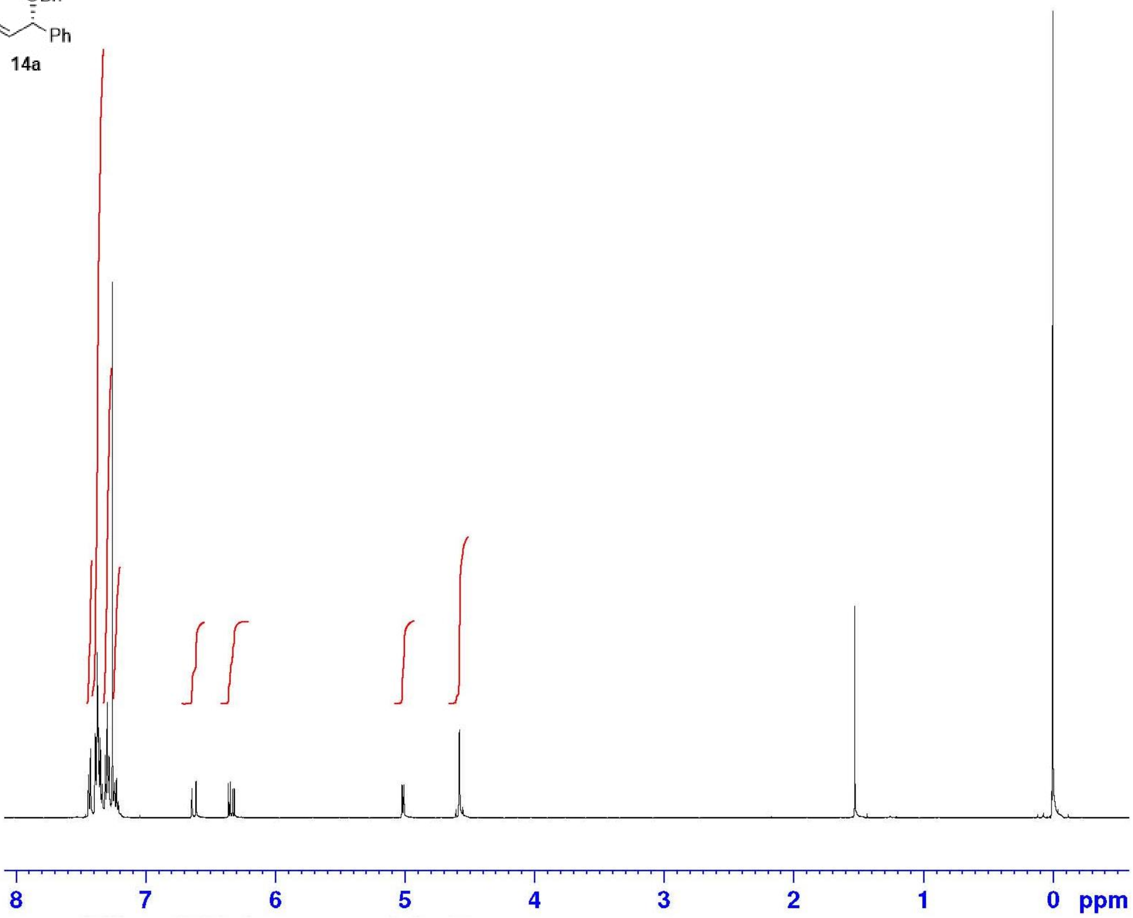

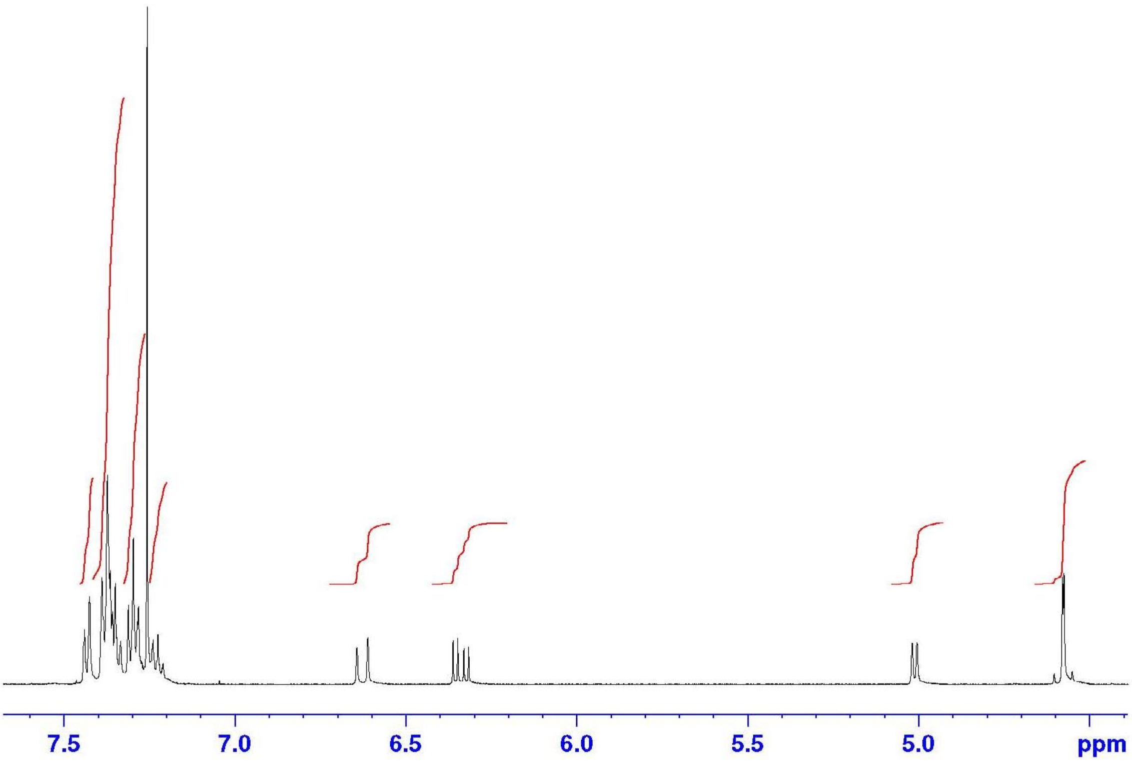

^{1}H NMR (500 MHz, $CDCl_3$) spectrum of compound **14b**

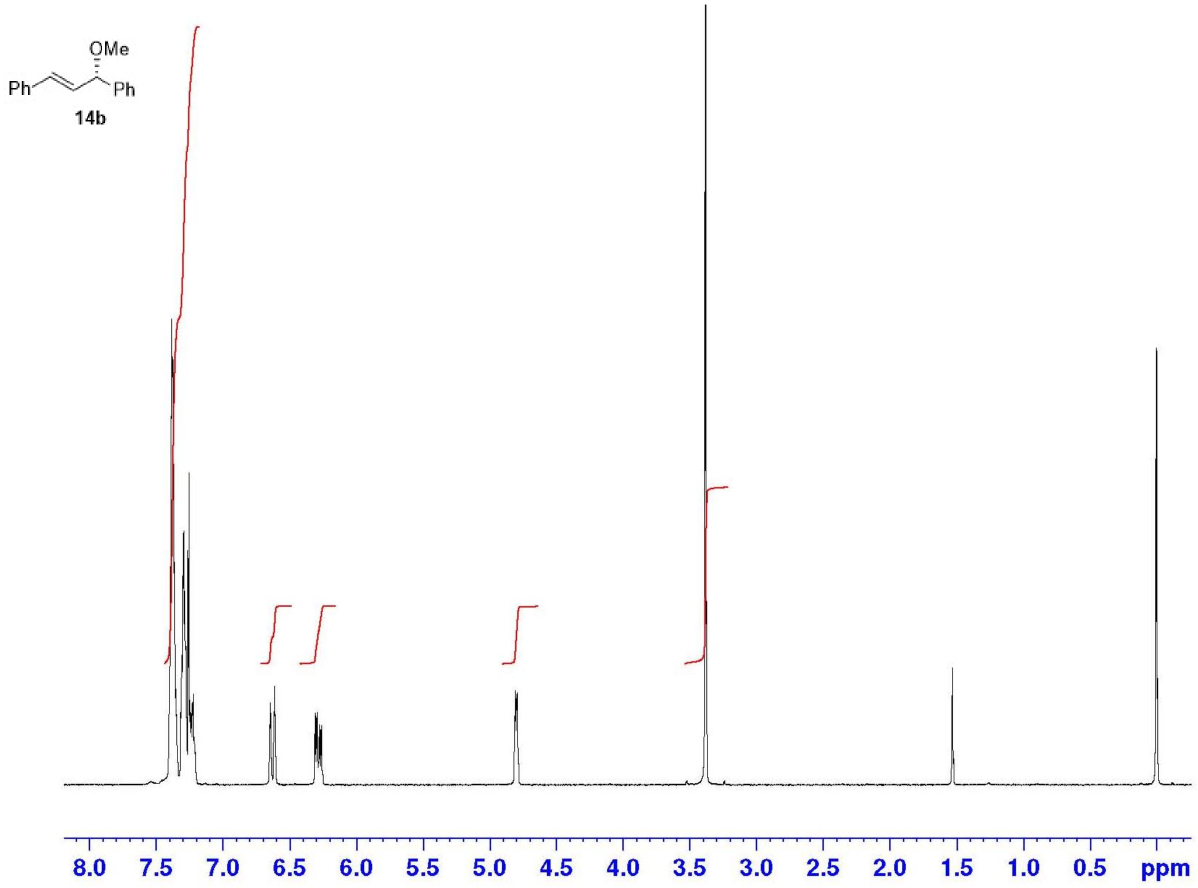

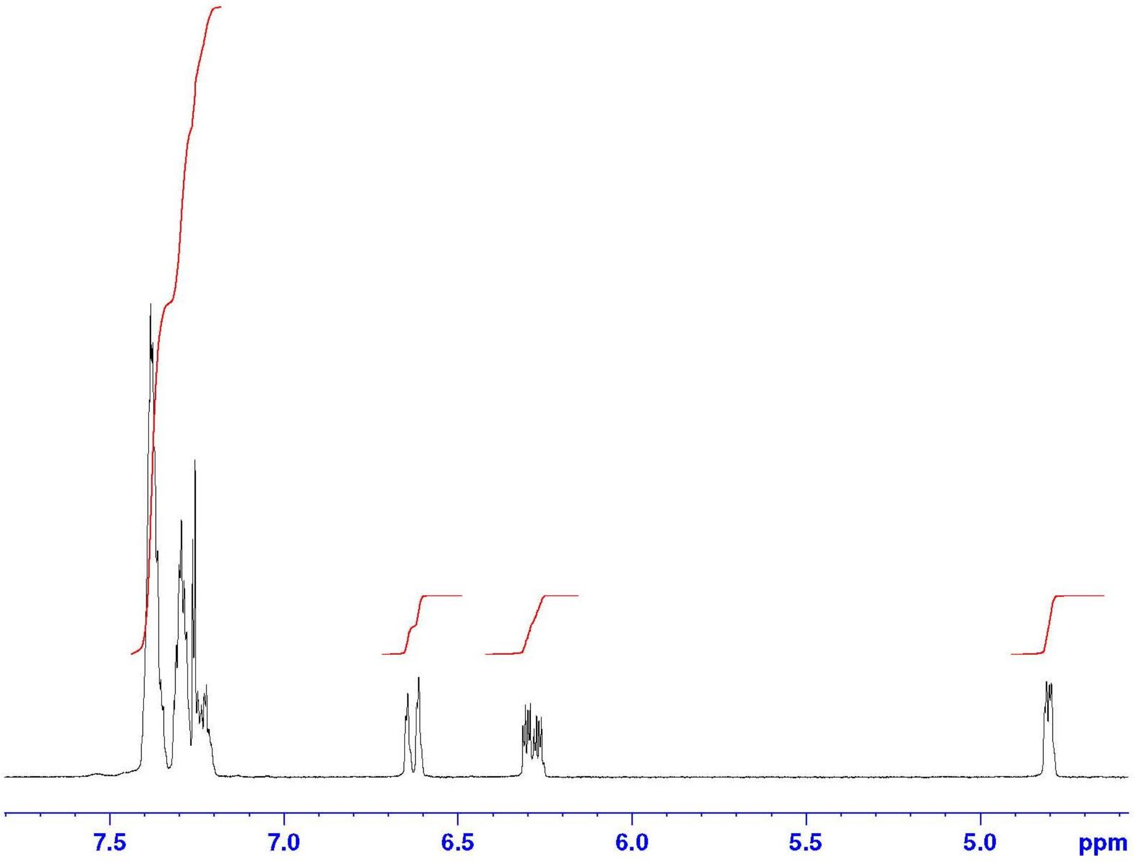

^{1}H NMR (500 MHz, $CDCl_3$) spectrum of compound **14c**

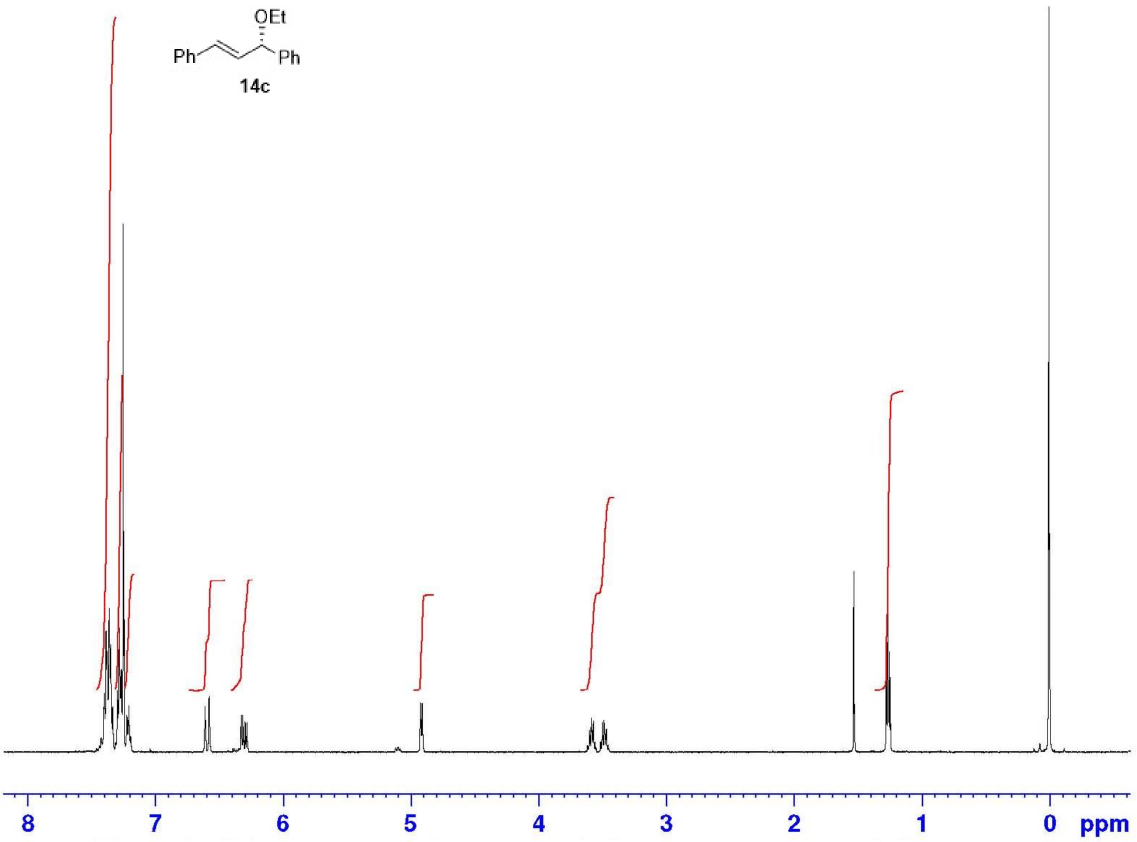

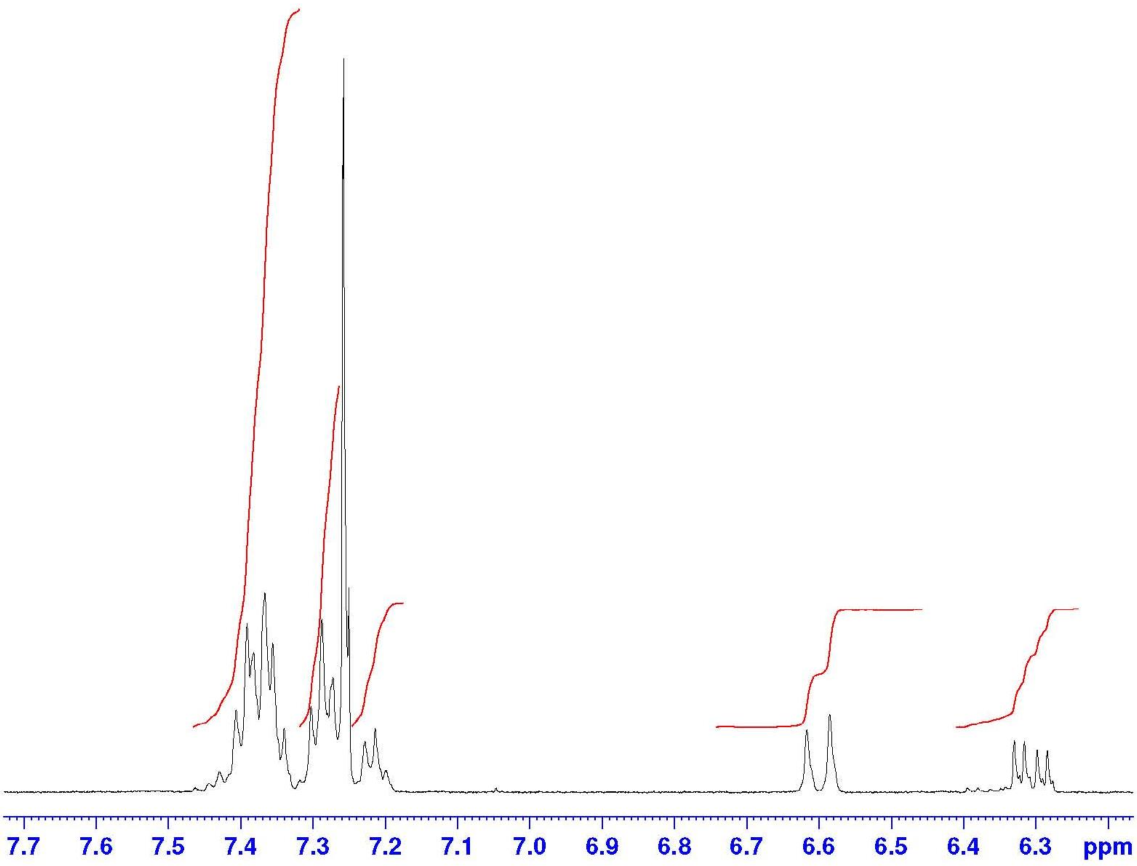

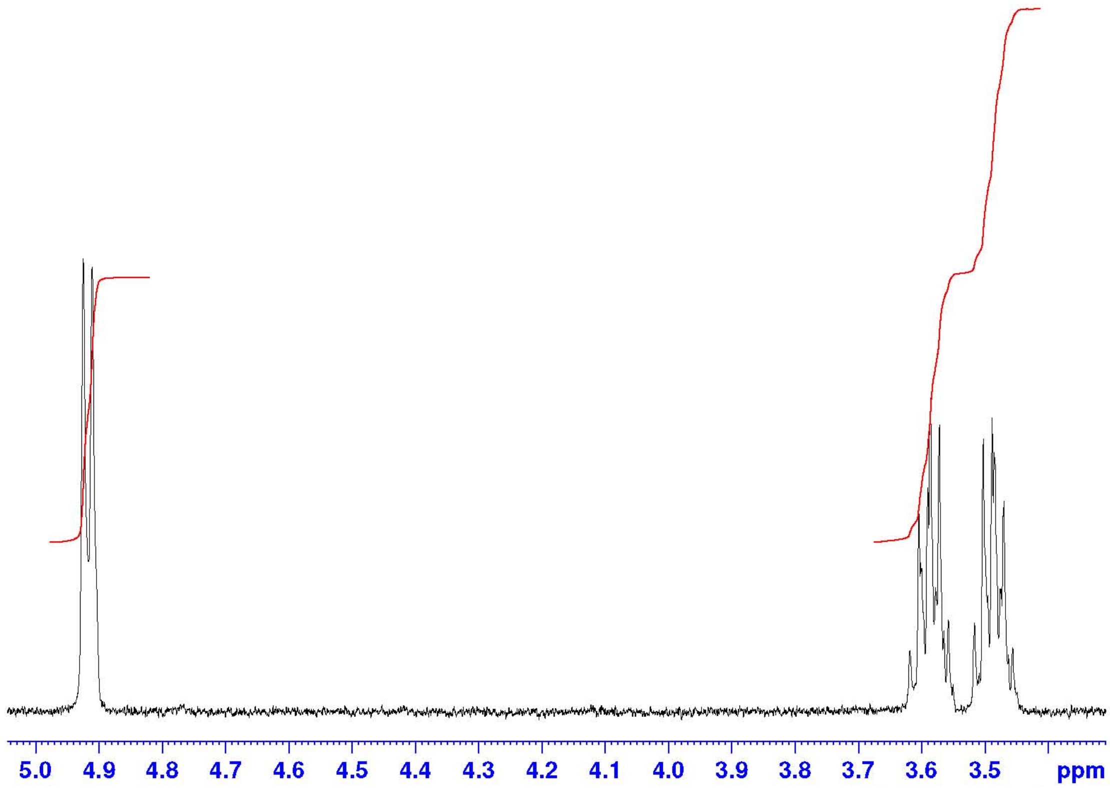

^{1}H NMR (500 MHz, $CDCl_3$) spectrum of compound **14d**

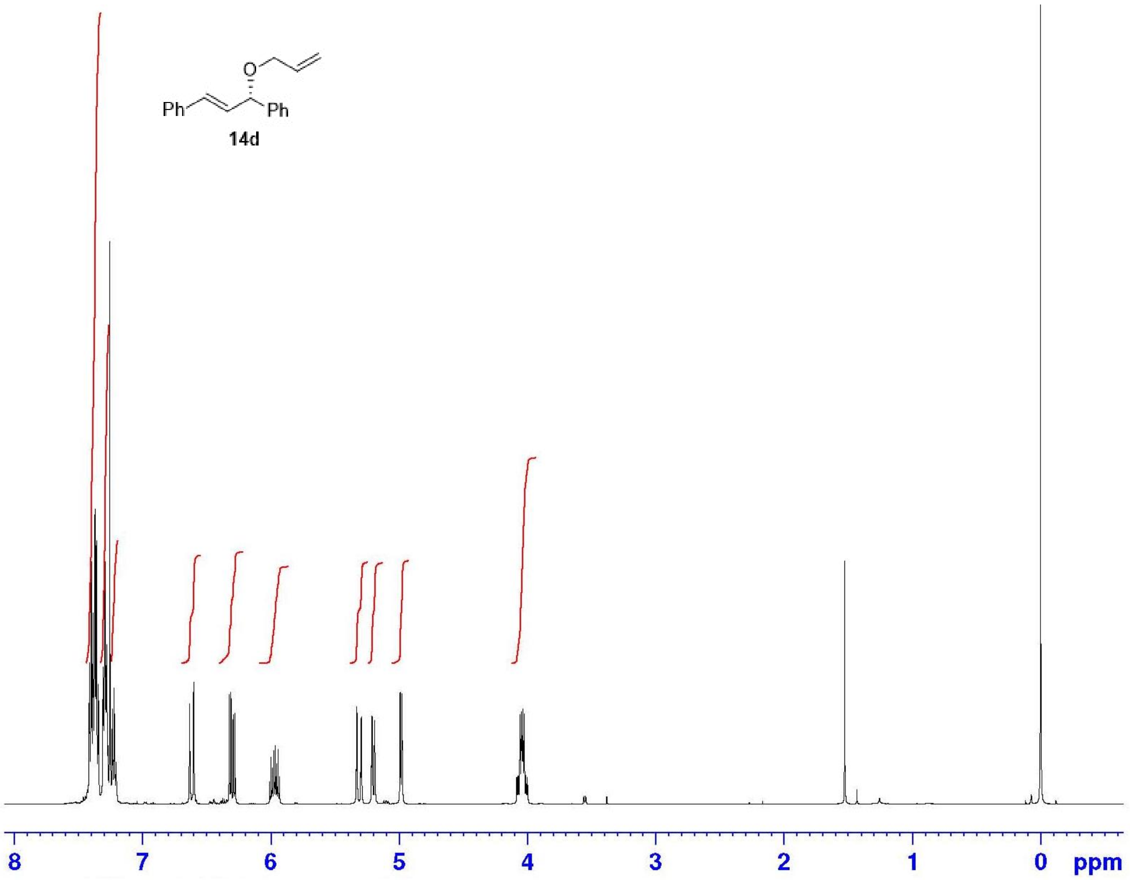

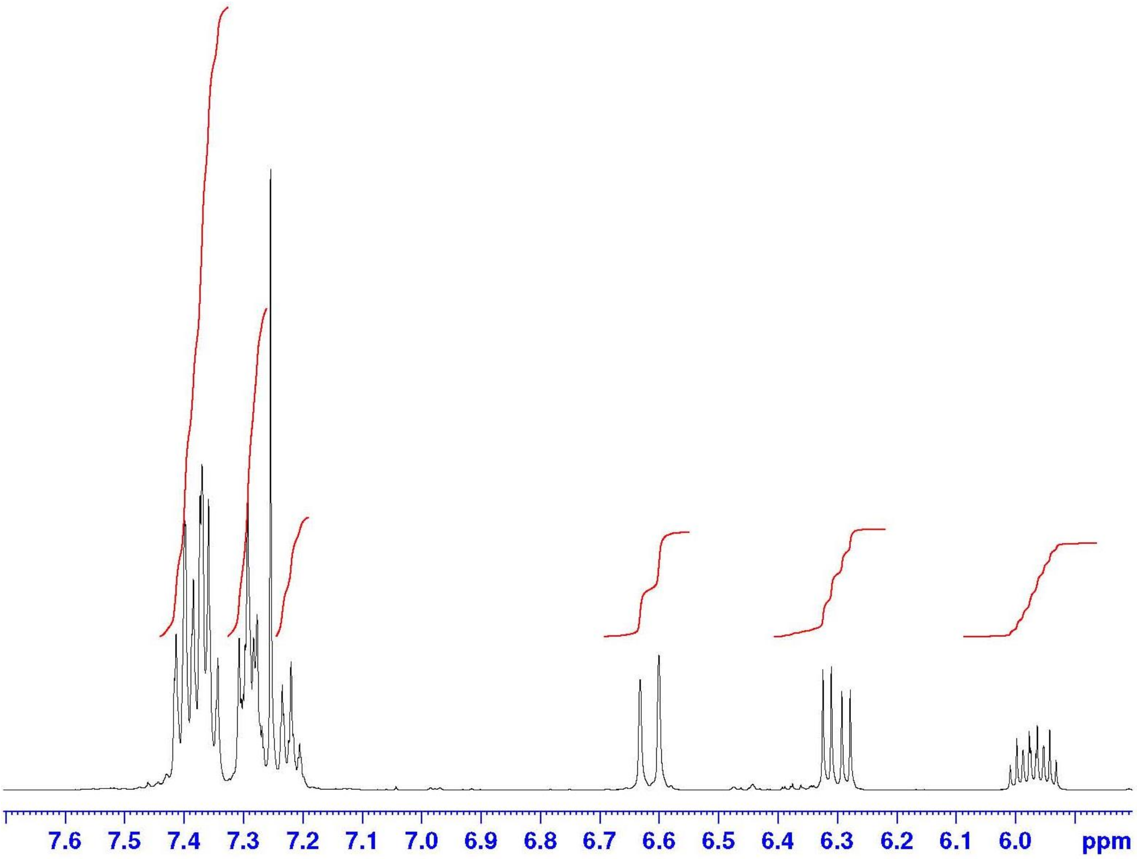
7.6 7.5 7.4 7.3 7.2 7.1 7.0 6.9 6.8 6.7 6.6 6.5 6.4 6.3 6.2 6.1 6.0 ppm

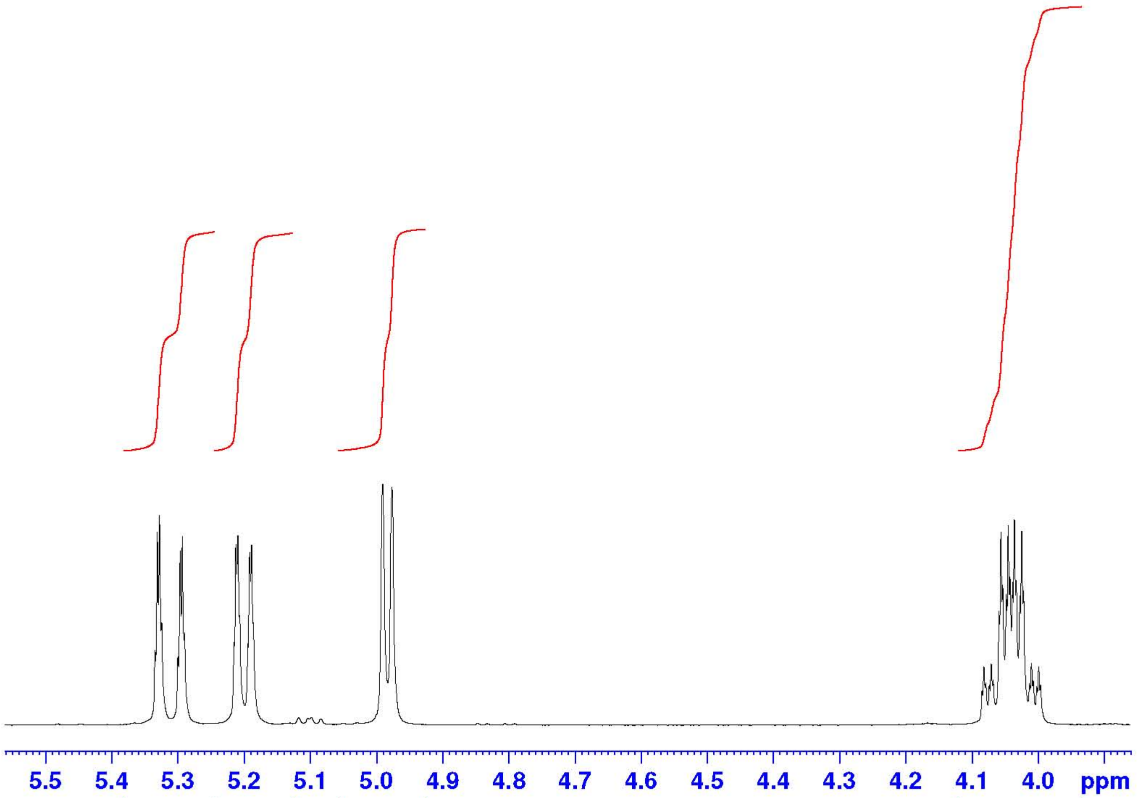
5.5 5.4 5.3 5.2 5.1 5.0 4.9 4.8 4.7 4.6 4.5 4.4 4.3 4.2 4.1 4.0 ppm

^{1}H NMR (500 MHz, $CDCl_3$) spectrum of compound **14e**

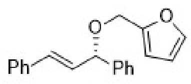

14e

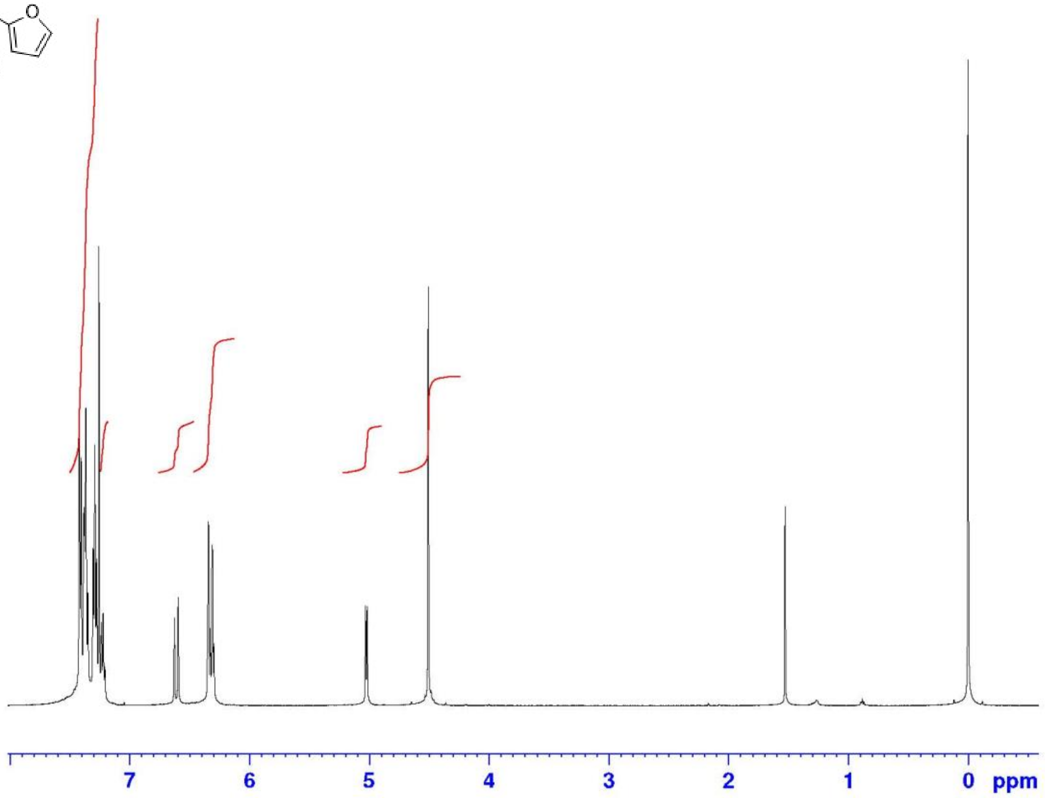

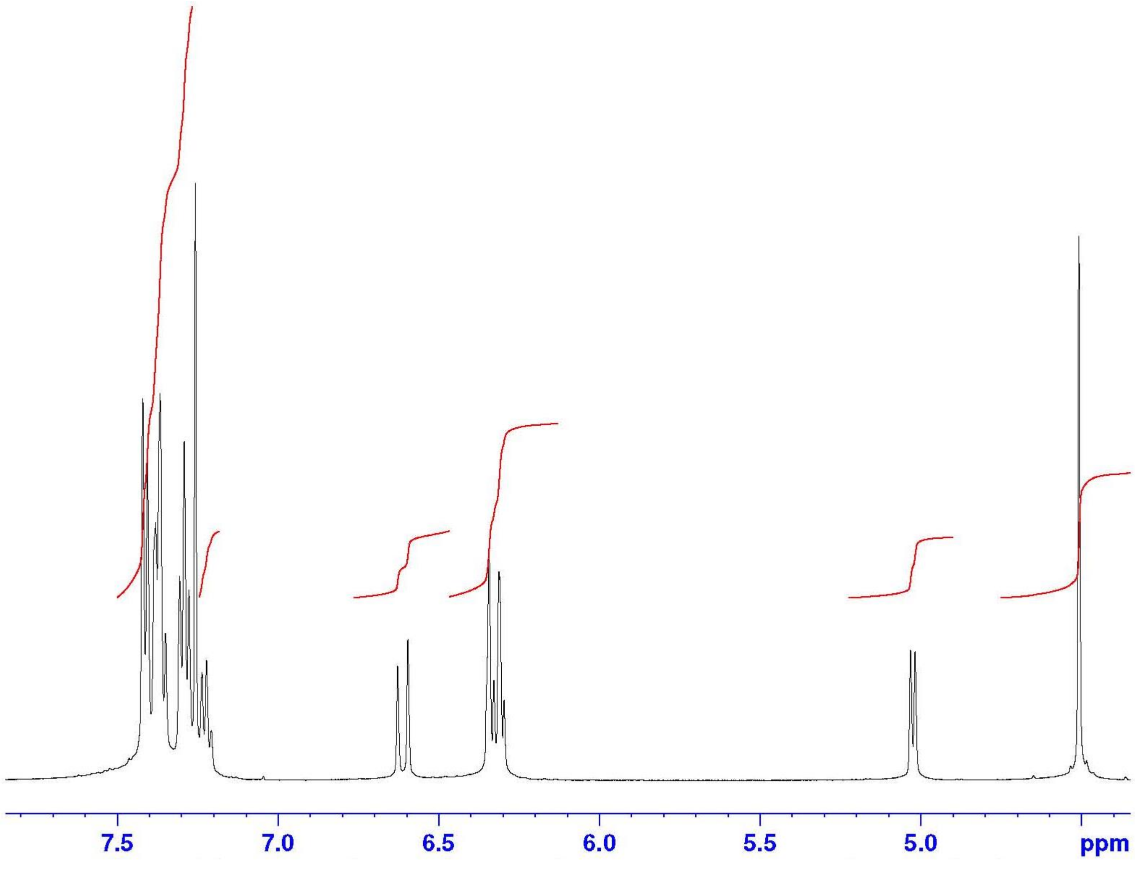

^{1}H NMR (500 MHz, $CDCl_3$) spectrum of compound **17a<u>a</u>**

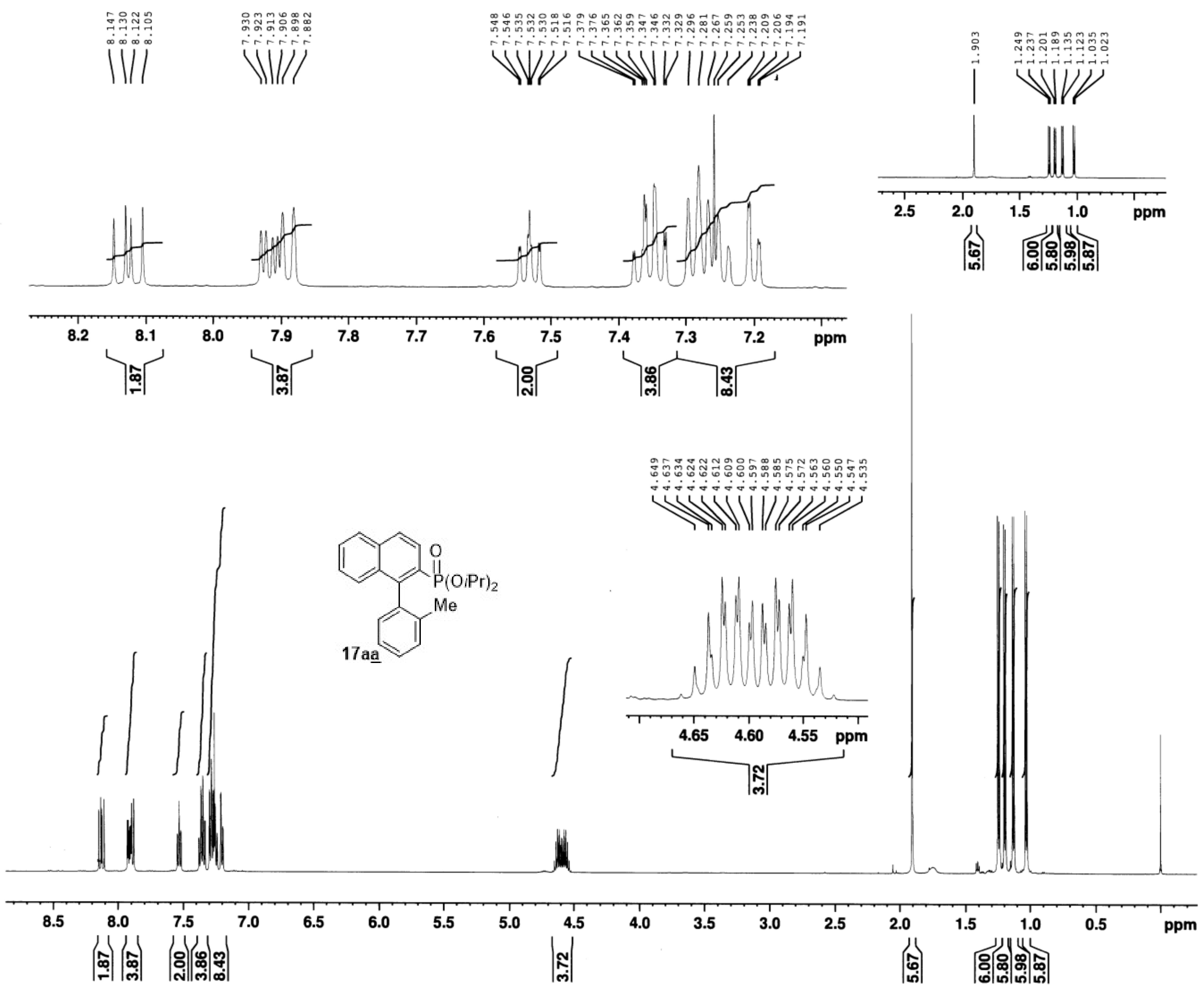

^{13}C NMR (125 MHz, $CDCl_3$) spectrum of compound **17a<u>a</u>**

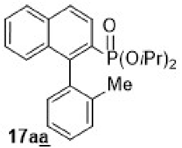
17aa

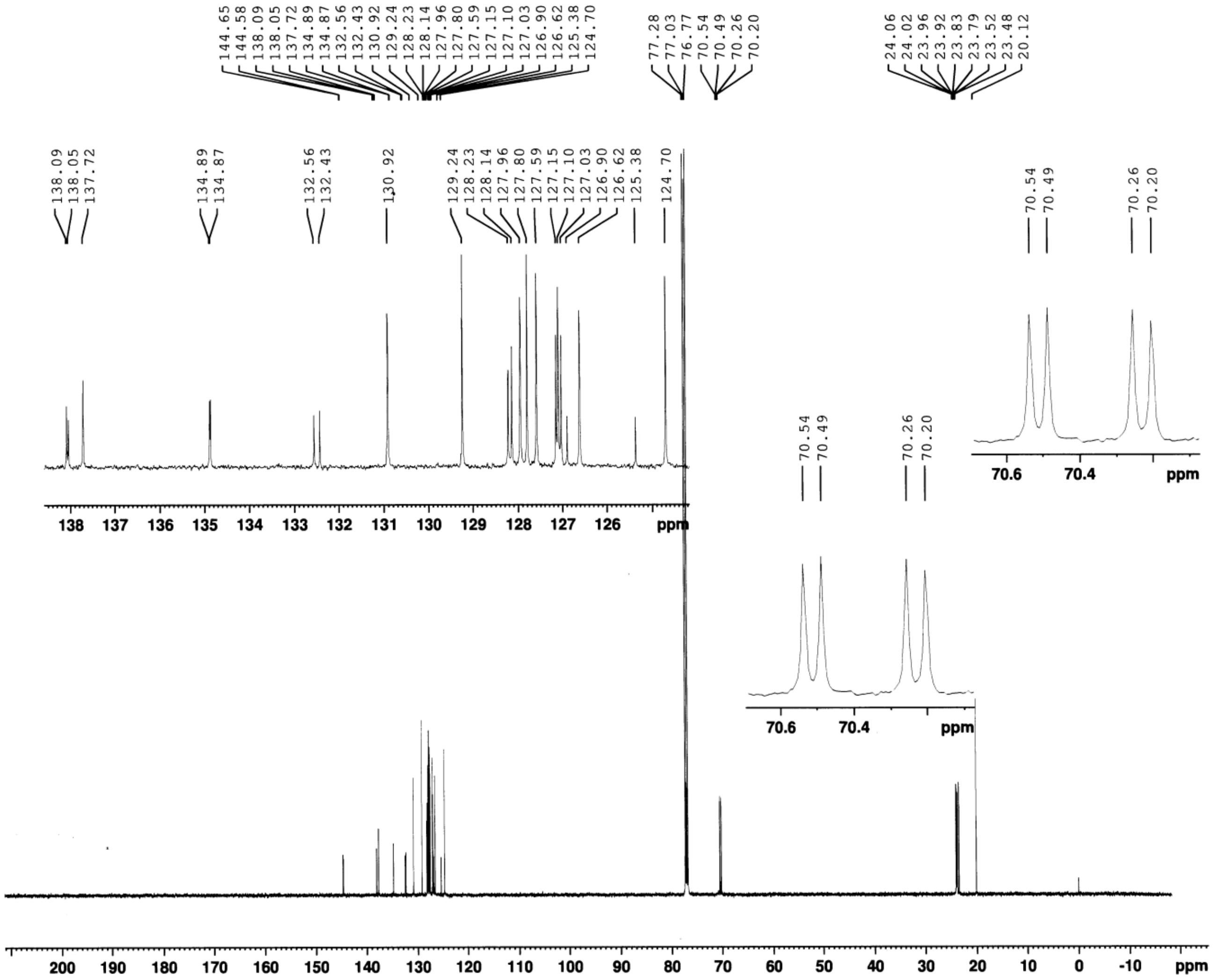

DEPT (125 MHz, $CDCl_3$) spectrum of compound **17aa**

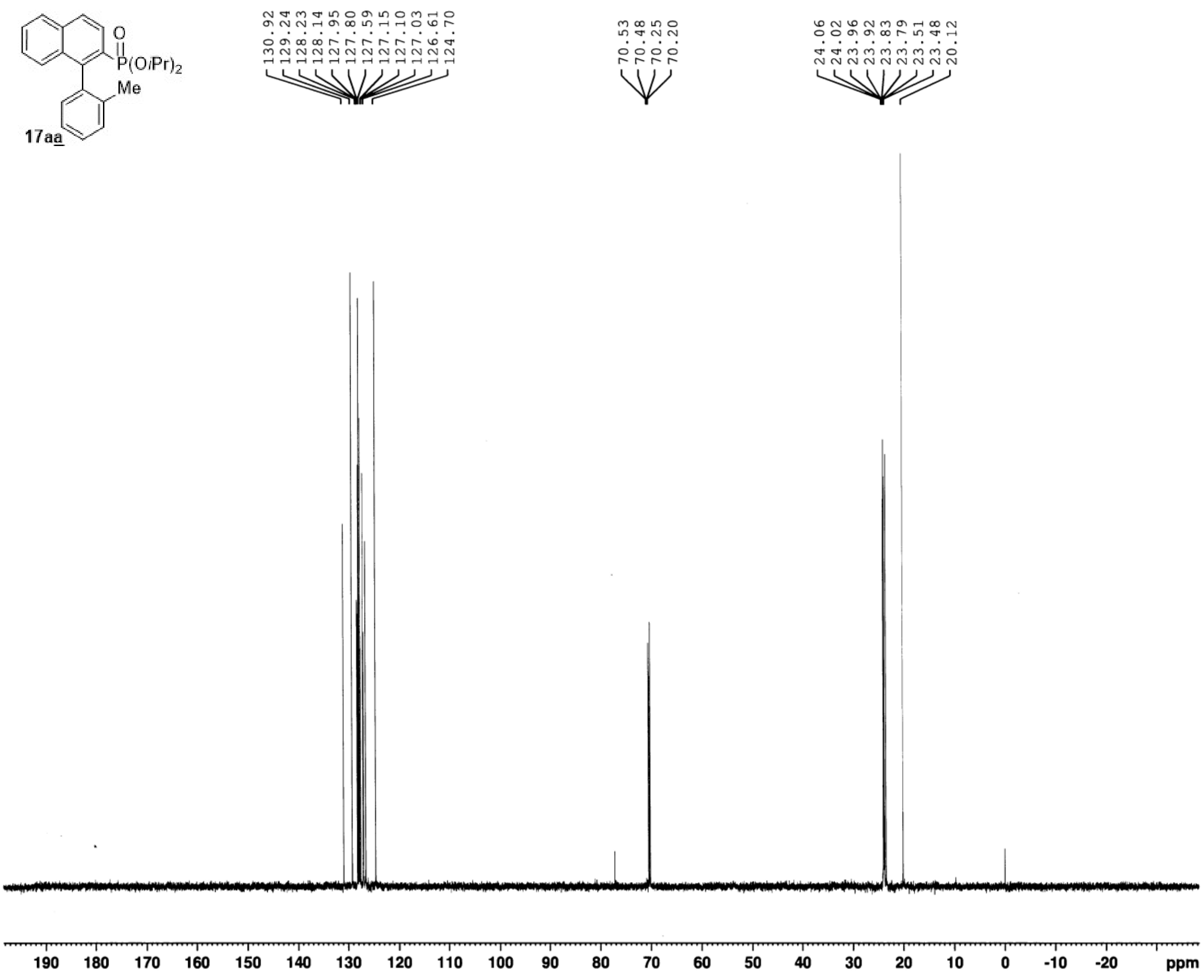

^{31}P NMR (202 MHz, $CDCl_3$) spectrum of compound **17aa**

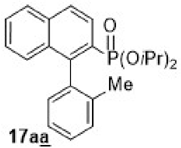

17aa

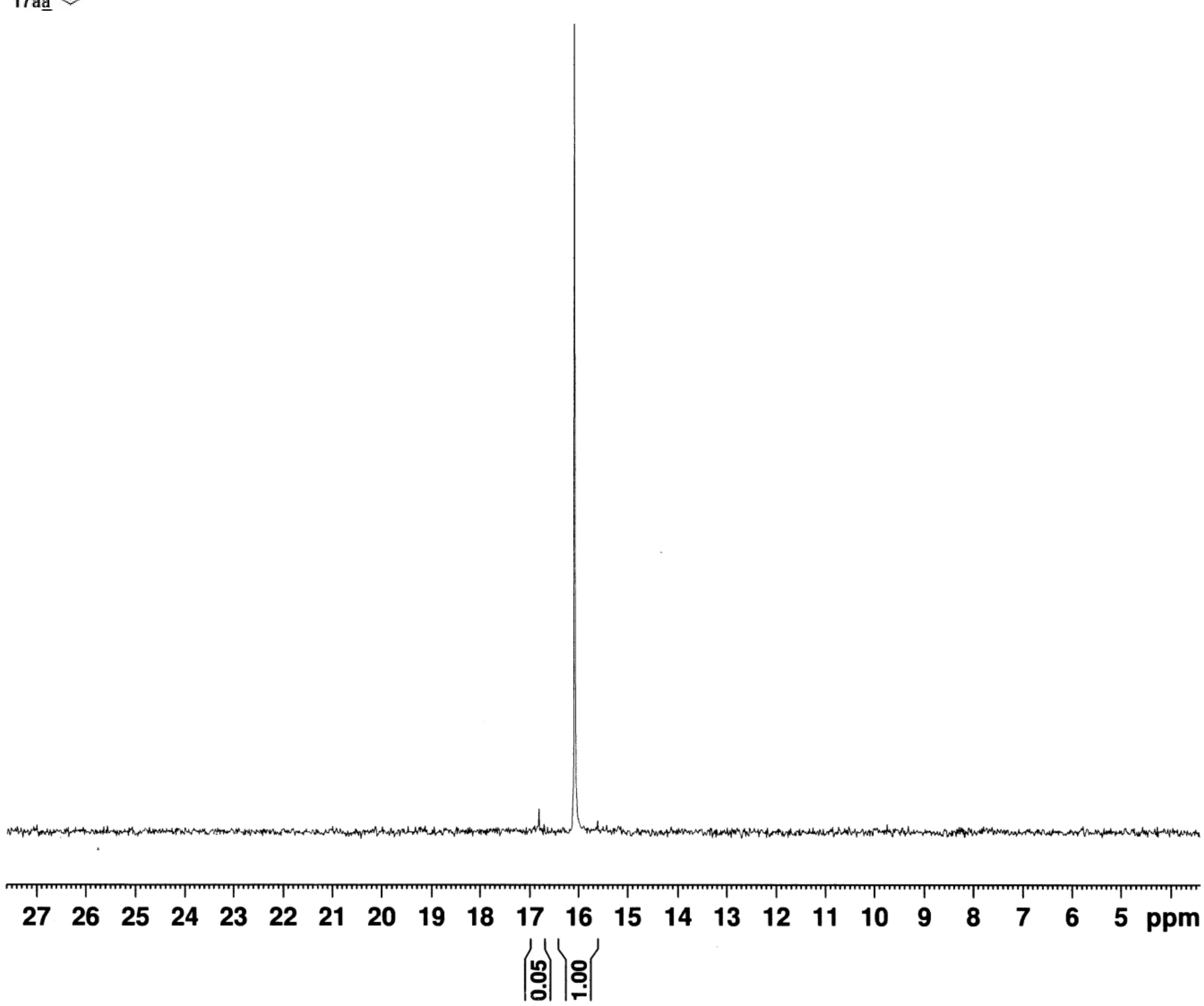

^{1}H NMR (500 MHz, $CDCl_3$) spectrum of compound **17b<u>a</u>**

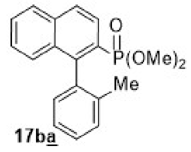

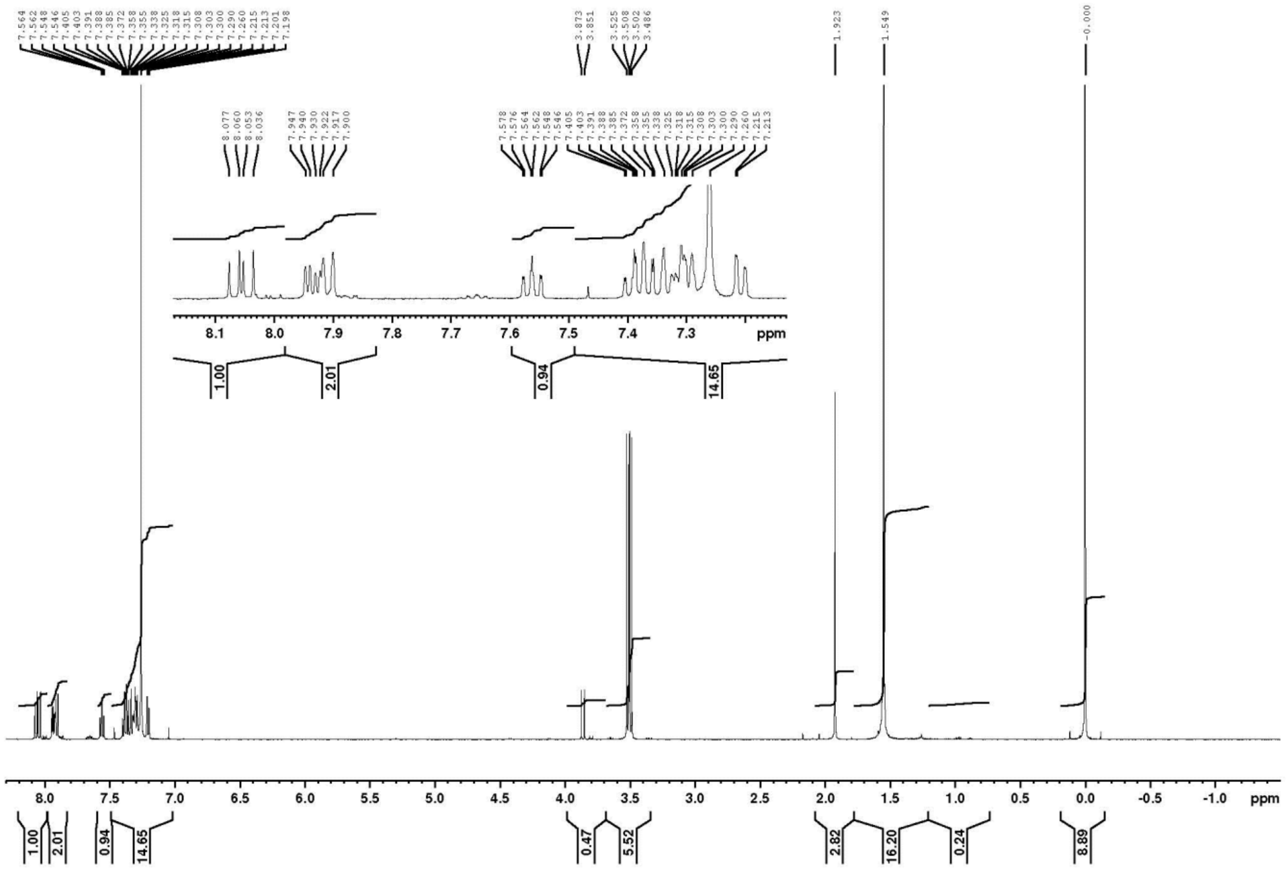

^{31}P NMR (202 MHz, $CDCl_3$) spectrum of compound **17ba**

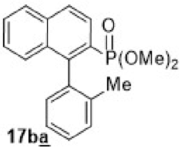

17ba

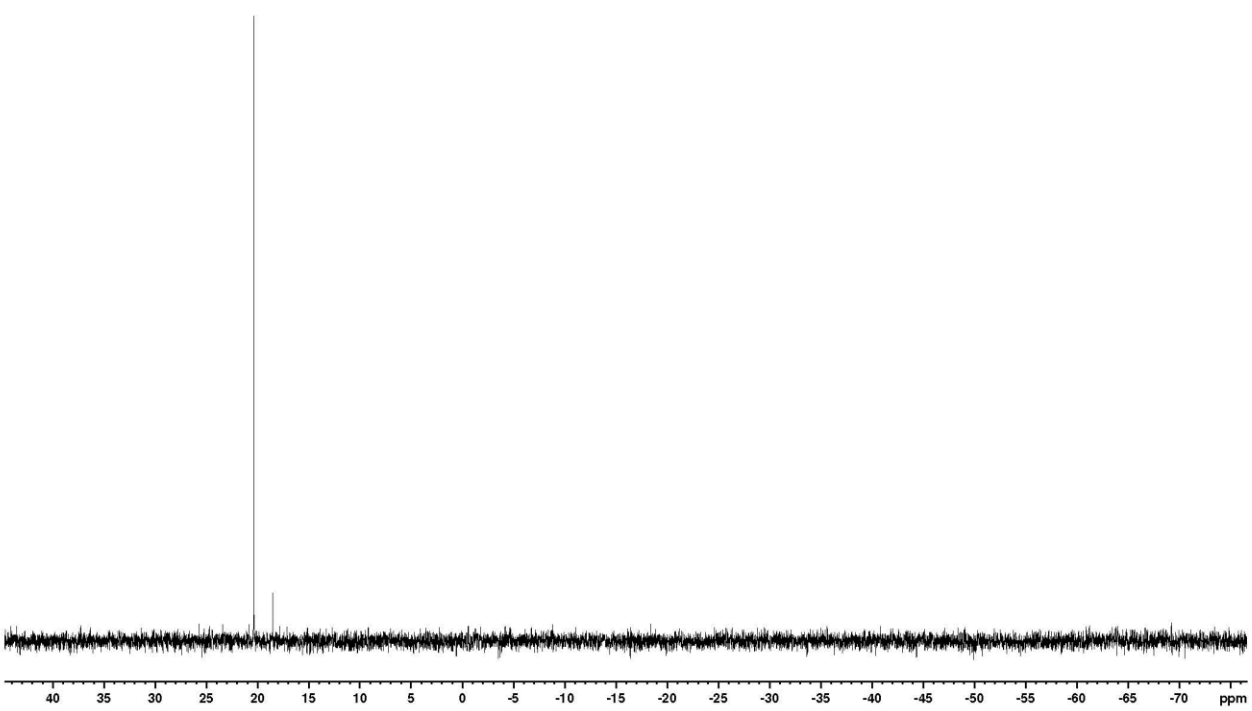

^{1}H NMR (500 MHz, $CDCl_3$) spectrum of compound **17c<u>a</u>**

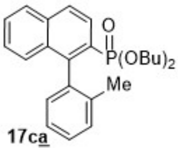

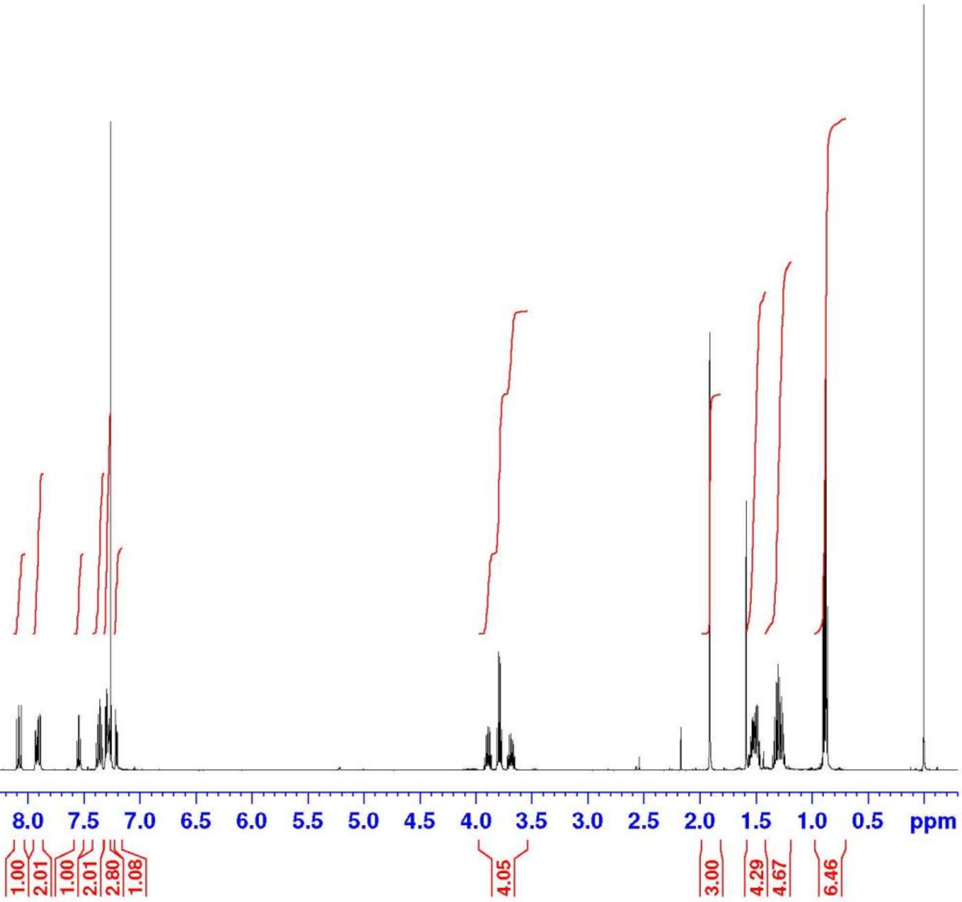

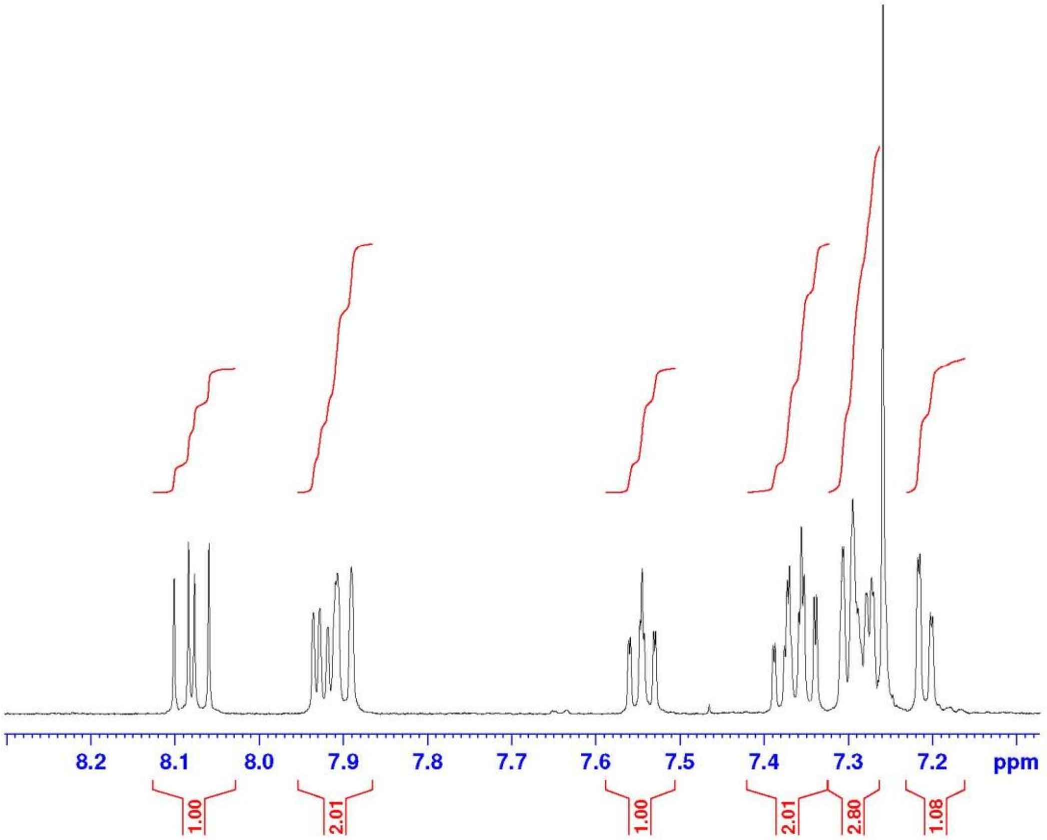

^{13}C NMR (125 MHz, $CDCl_3$) spectrum of compound **17c<u>a</u>**

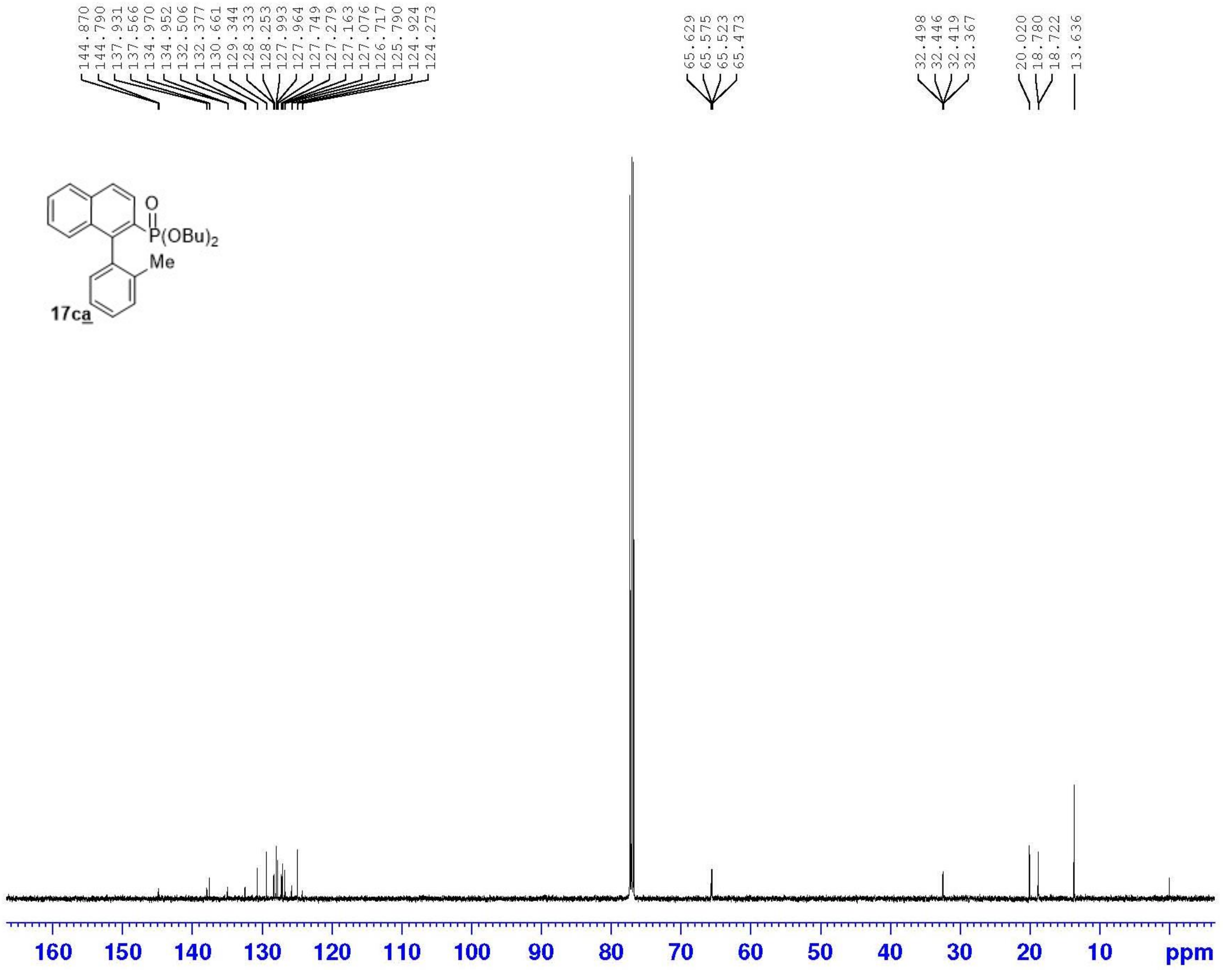

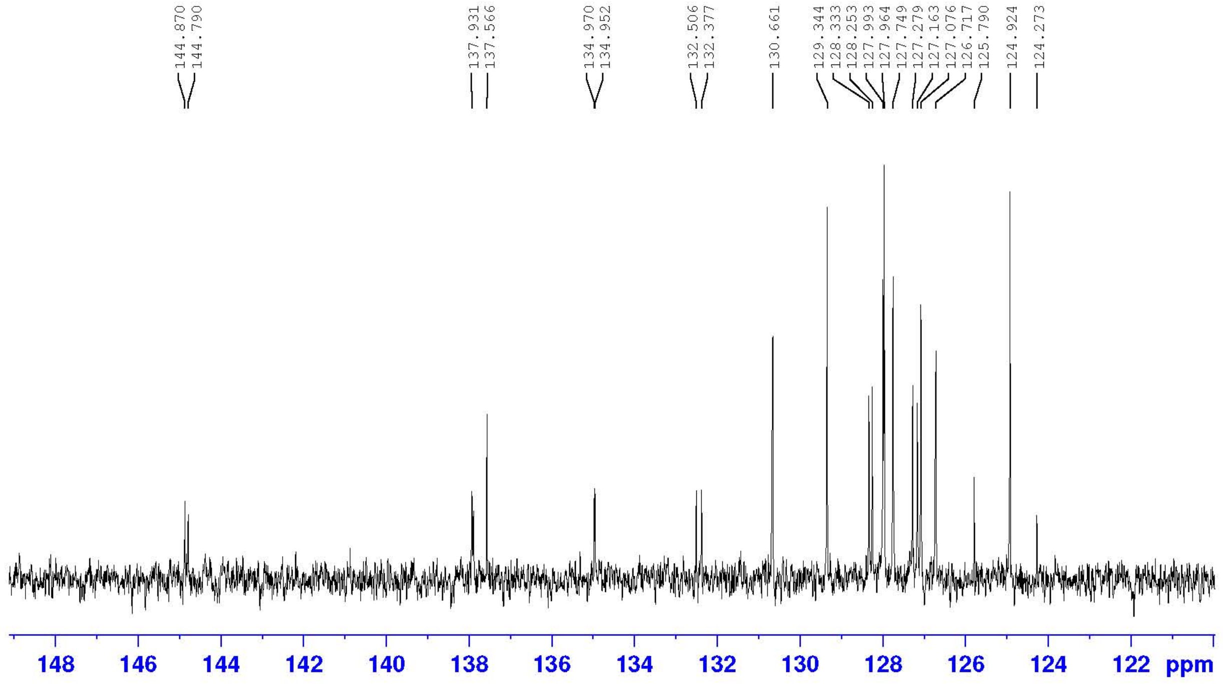

DEPT (125 MHz, $CDCl_3$) spectrum of compound **17c<u>a</u>**

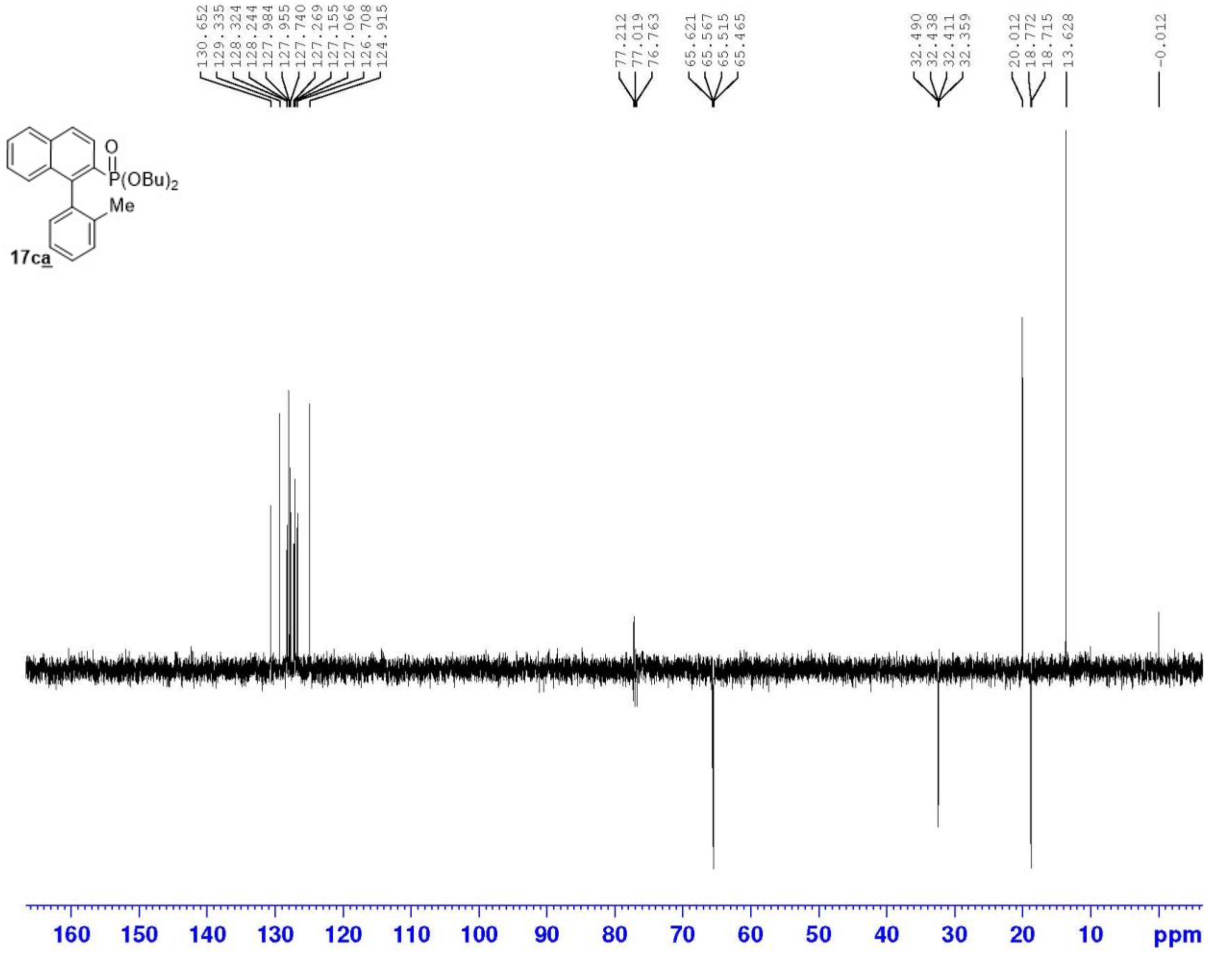

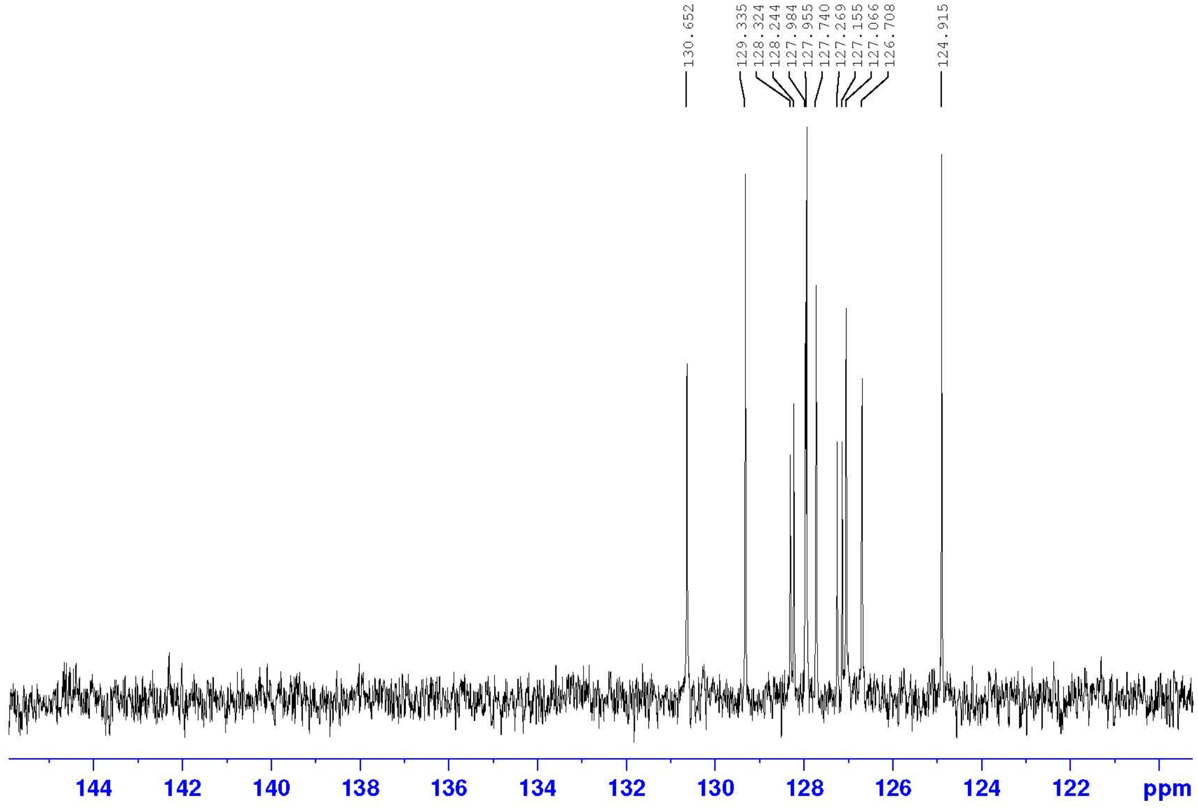

^{31}P NMR (202 MHz, $CDCl_3$) spectrum of compound **17c<u>a</u>**

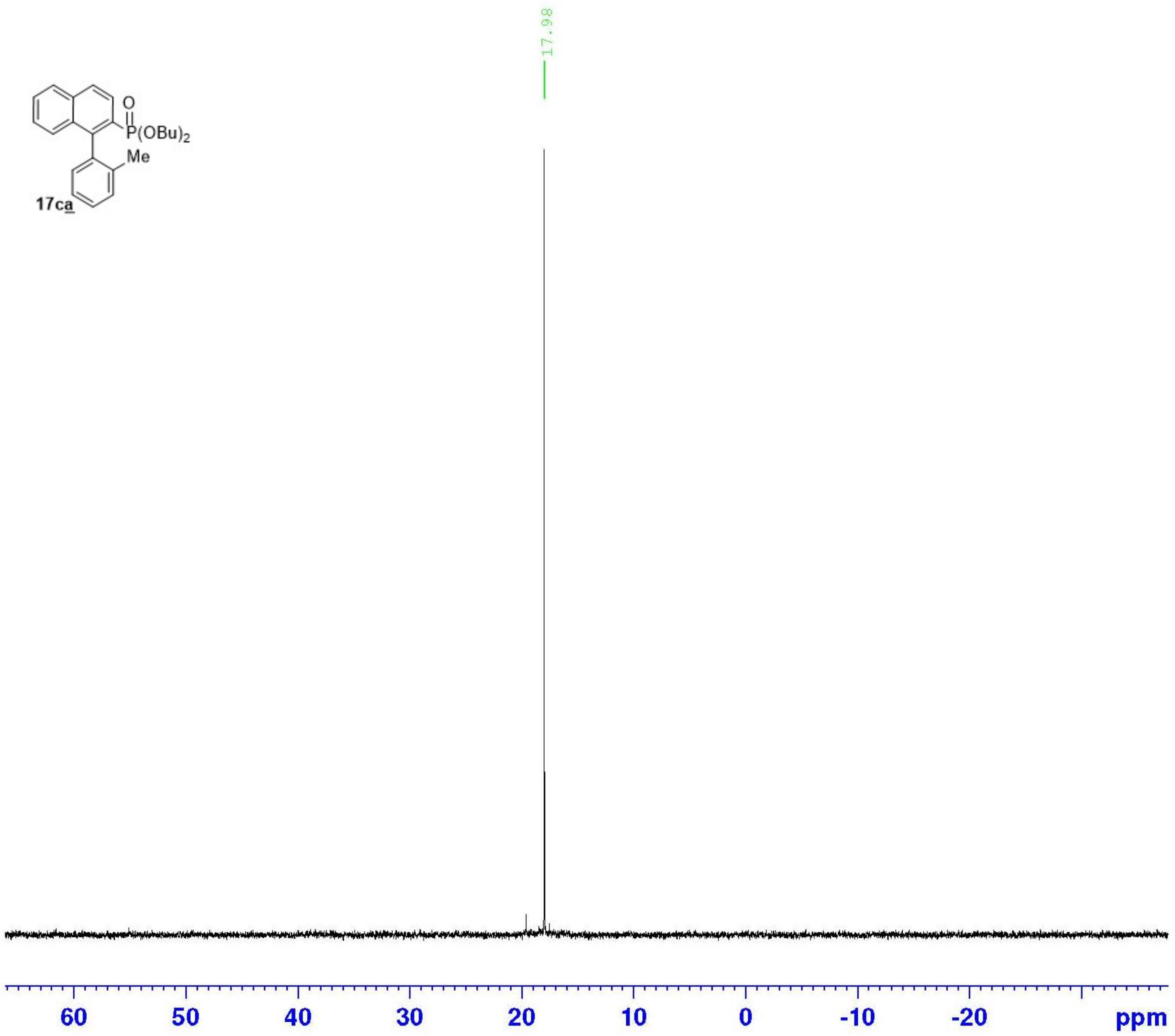

^{1}H NMR (500 MHz, $CDCl_3$) spectrum of compound **17d<u>a</u>**

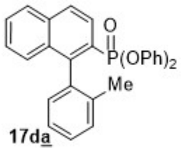

17d<u>a</u>

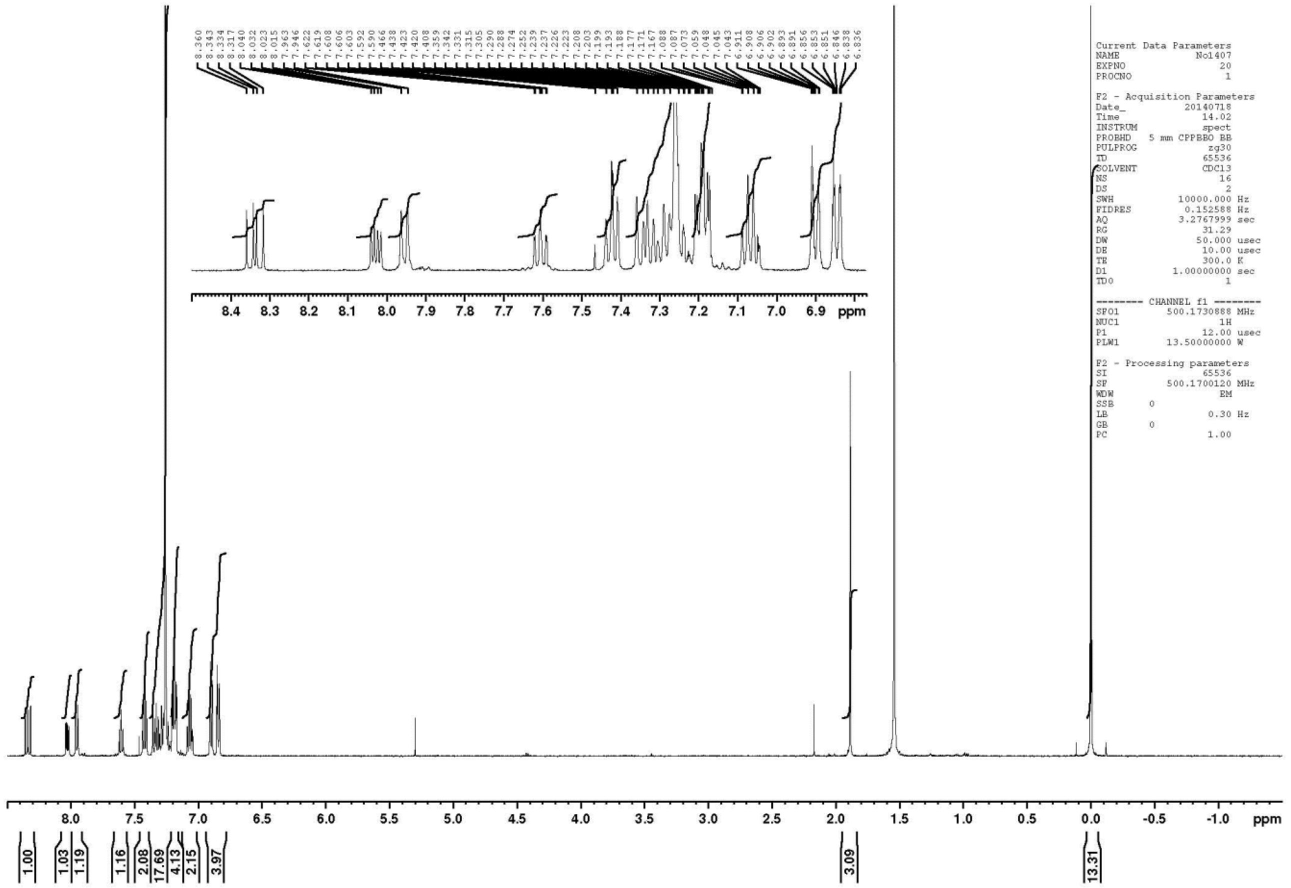

^{31}P NMR (202 MHz, $CDCl_3$) spectrum of compound **17da**

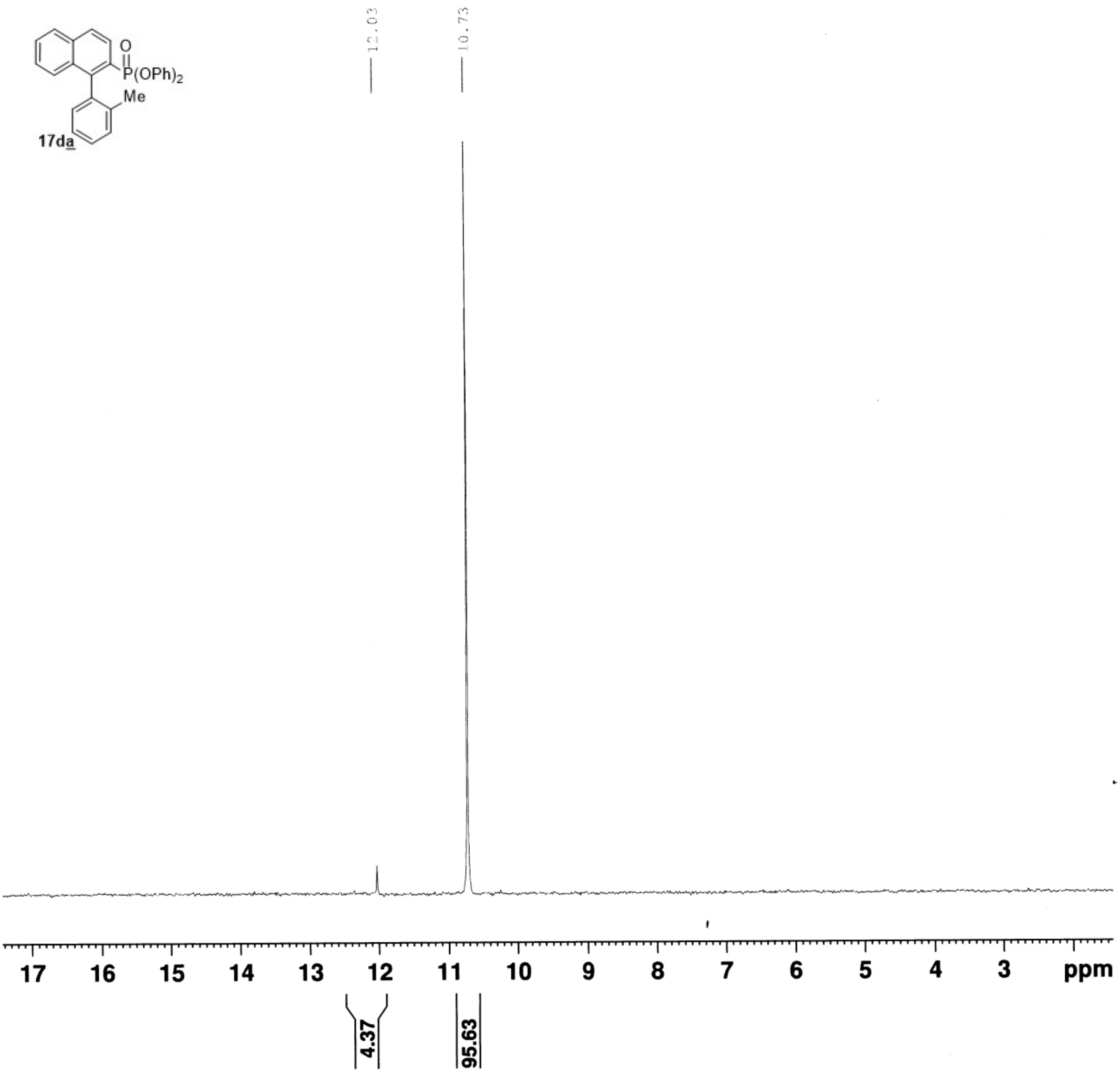

^{1}H NMR (500 MHz, $CDCl_3$) spectrum of compound **17a<u>b</u>**

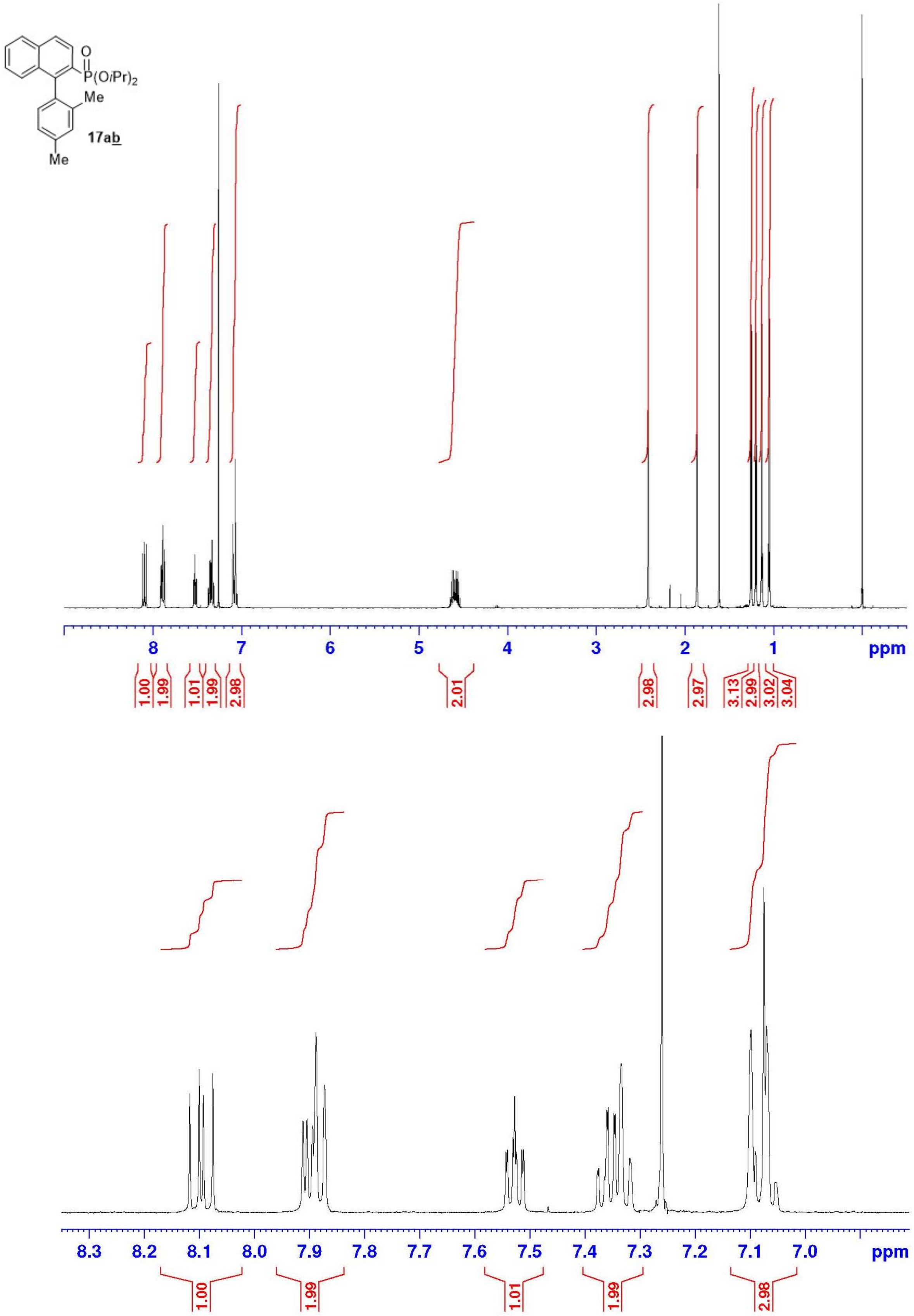

^{13}C NMR (125 MHz, $CDCl_3$) spectrum of compound **17ab**

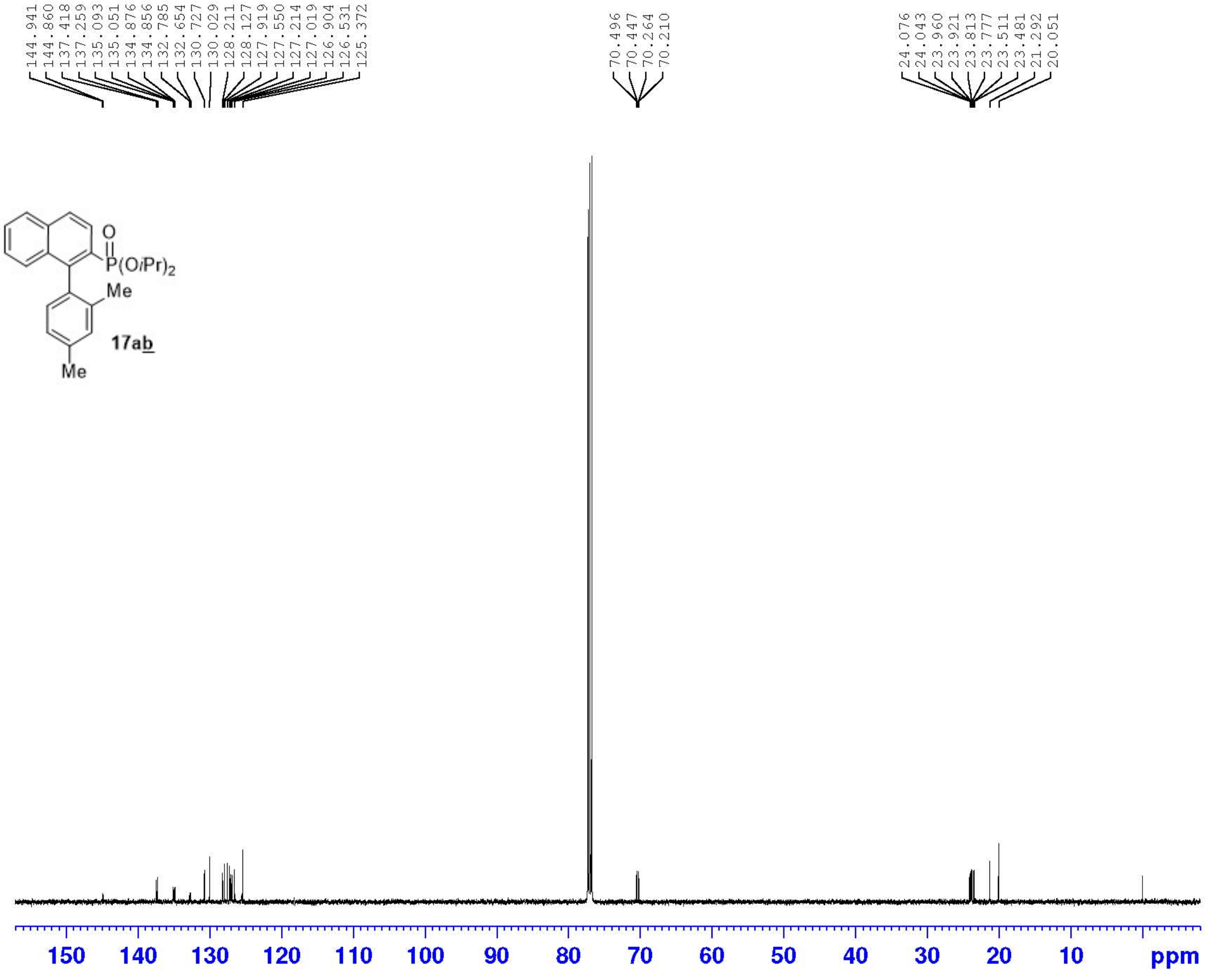

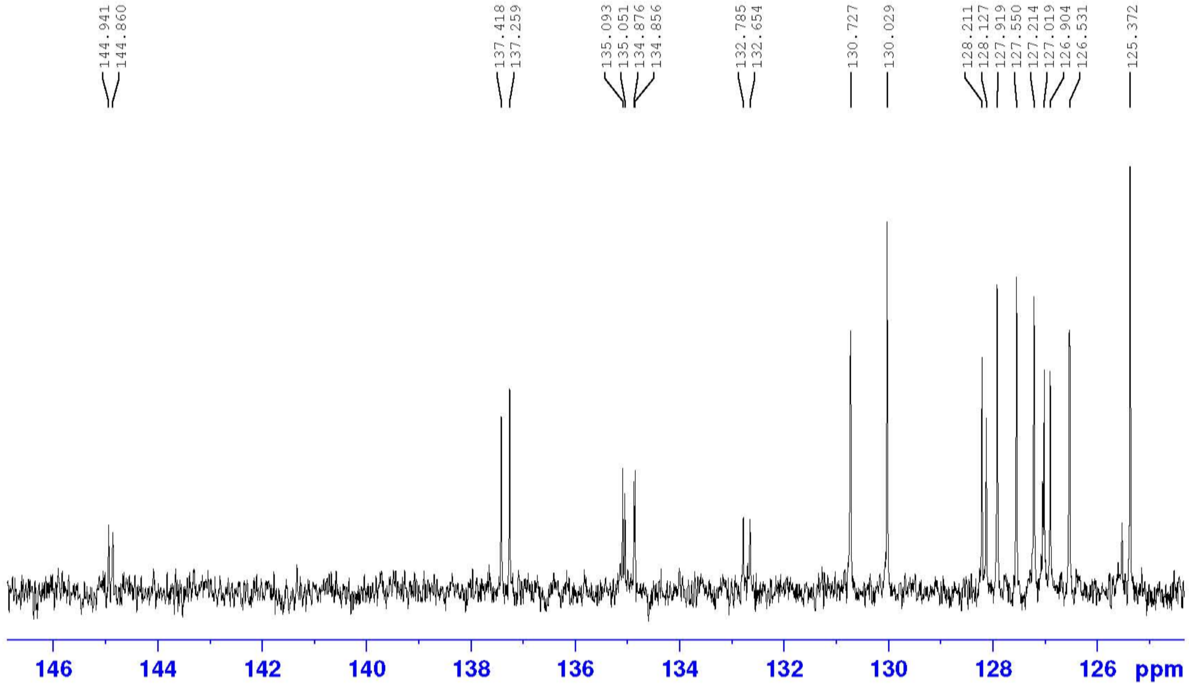

DEPT (125 MHz, $CDCl_3$) spectrum of compound **17a<u>b</u>**

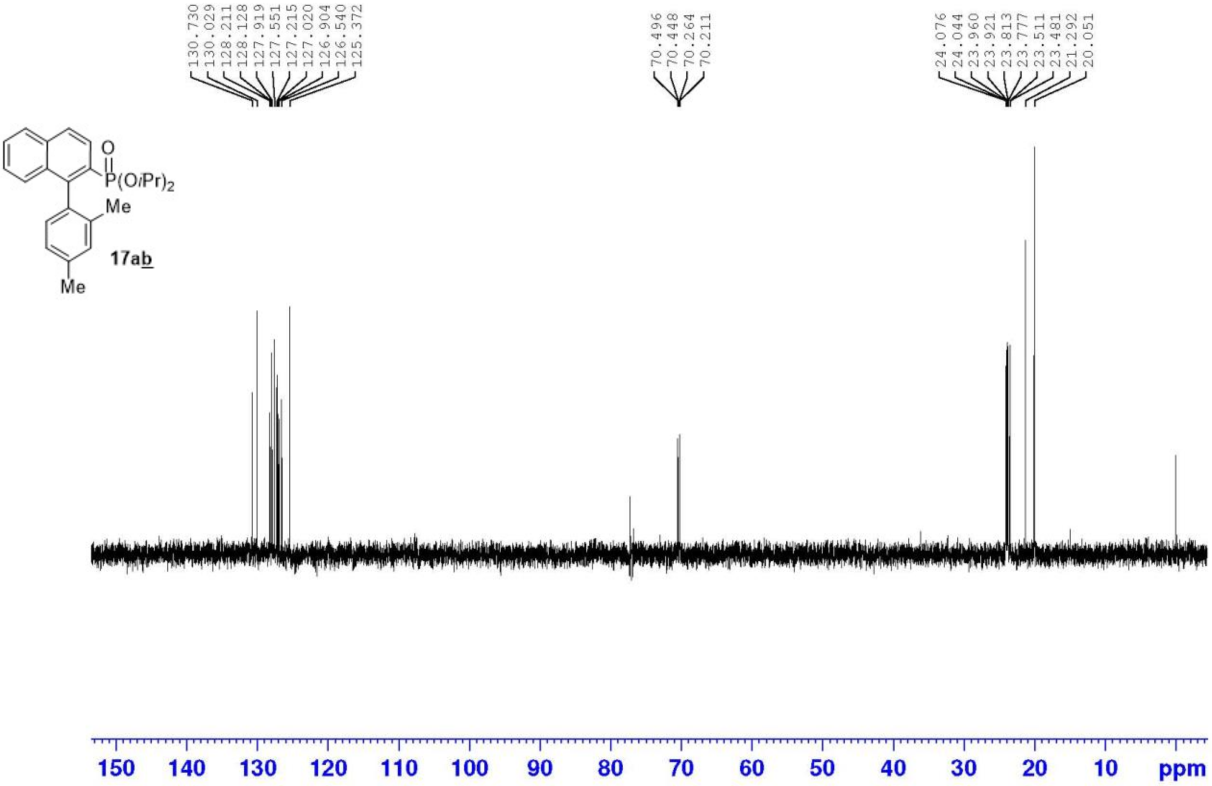

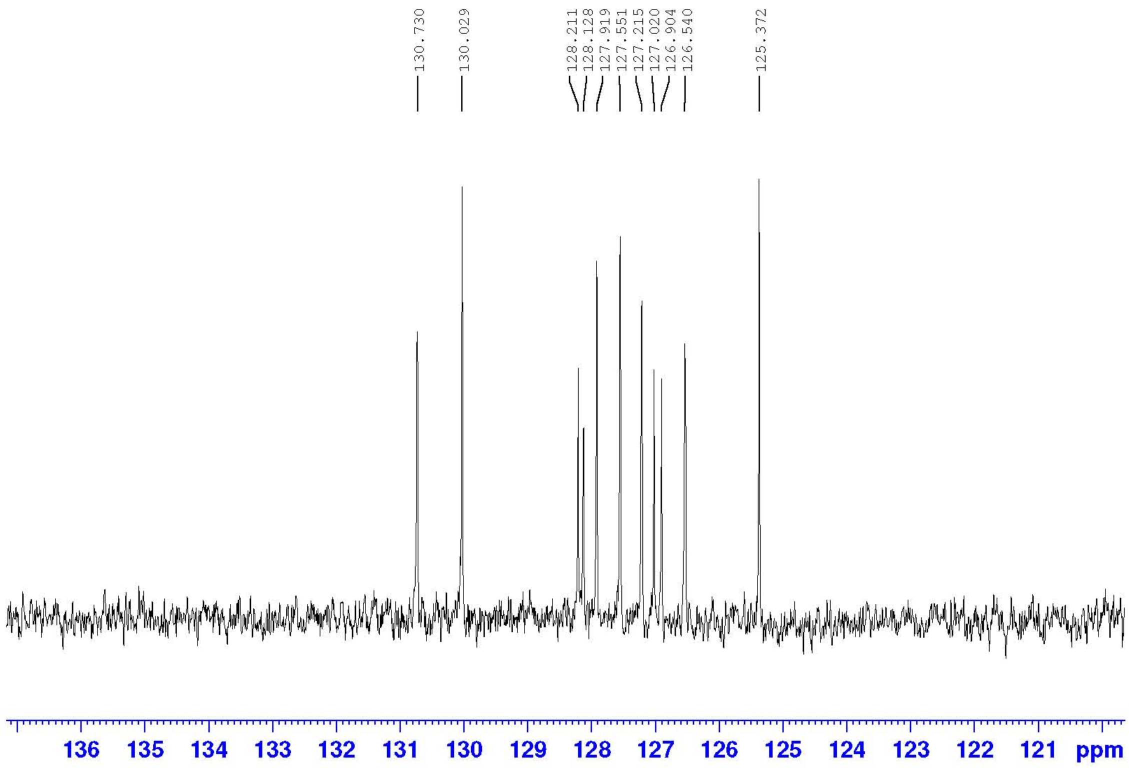

^{31}P NMR (202 MHz, $CDCl_3$) spectrum of compound **17ab**

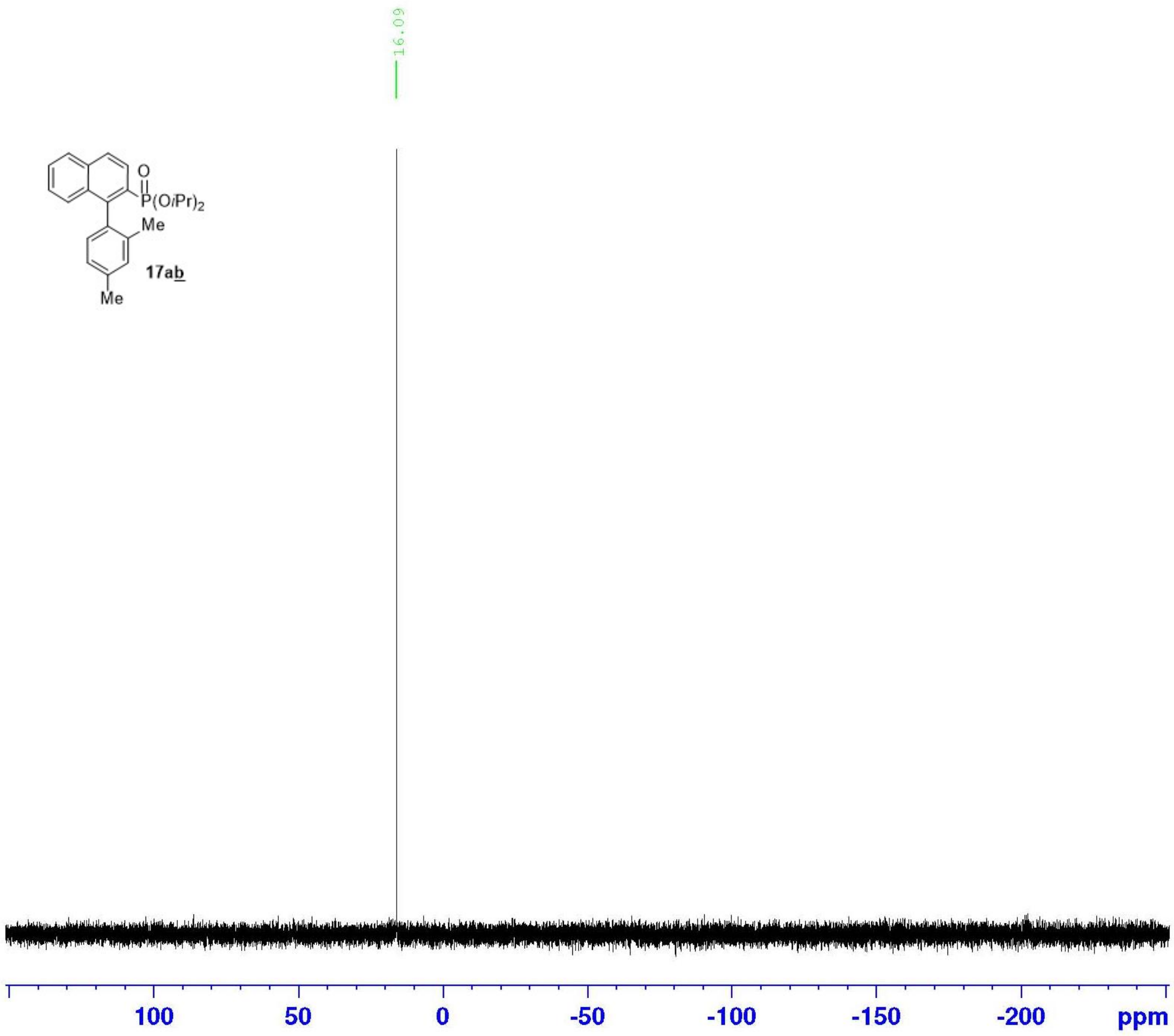

^{1}H NMR (500 MHz, $CDCl_3$) spectrum of compound **17a<u>c</u>**

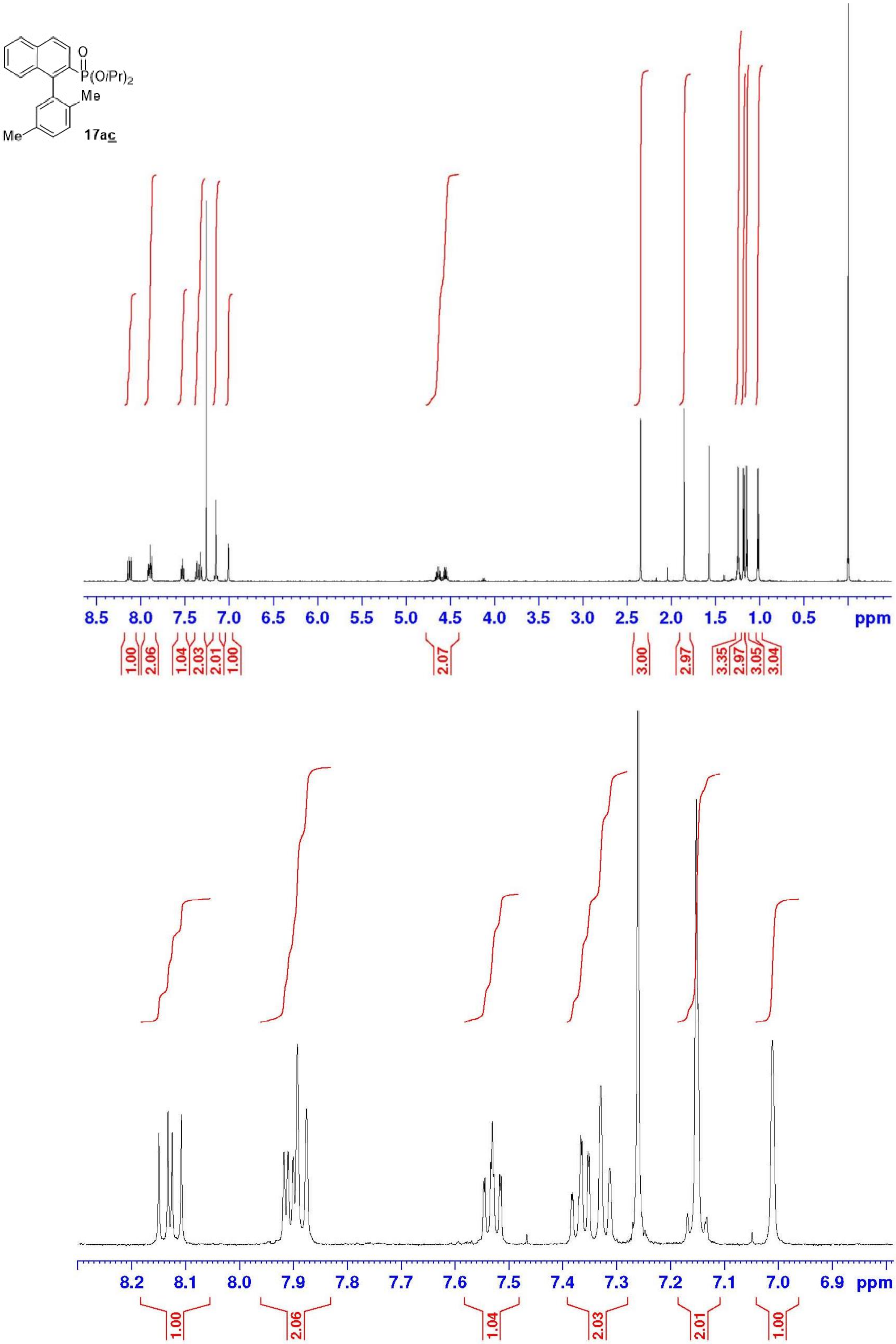

17a<u>c</u>

^{13}C NMR (125 MHz, $CDCl_3$) spectrum of compound **17a<u>c</u>**

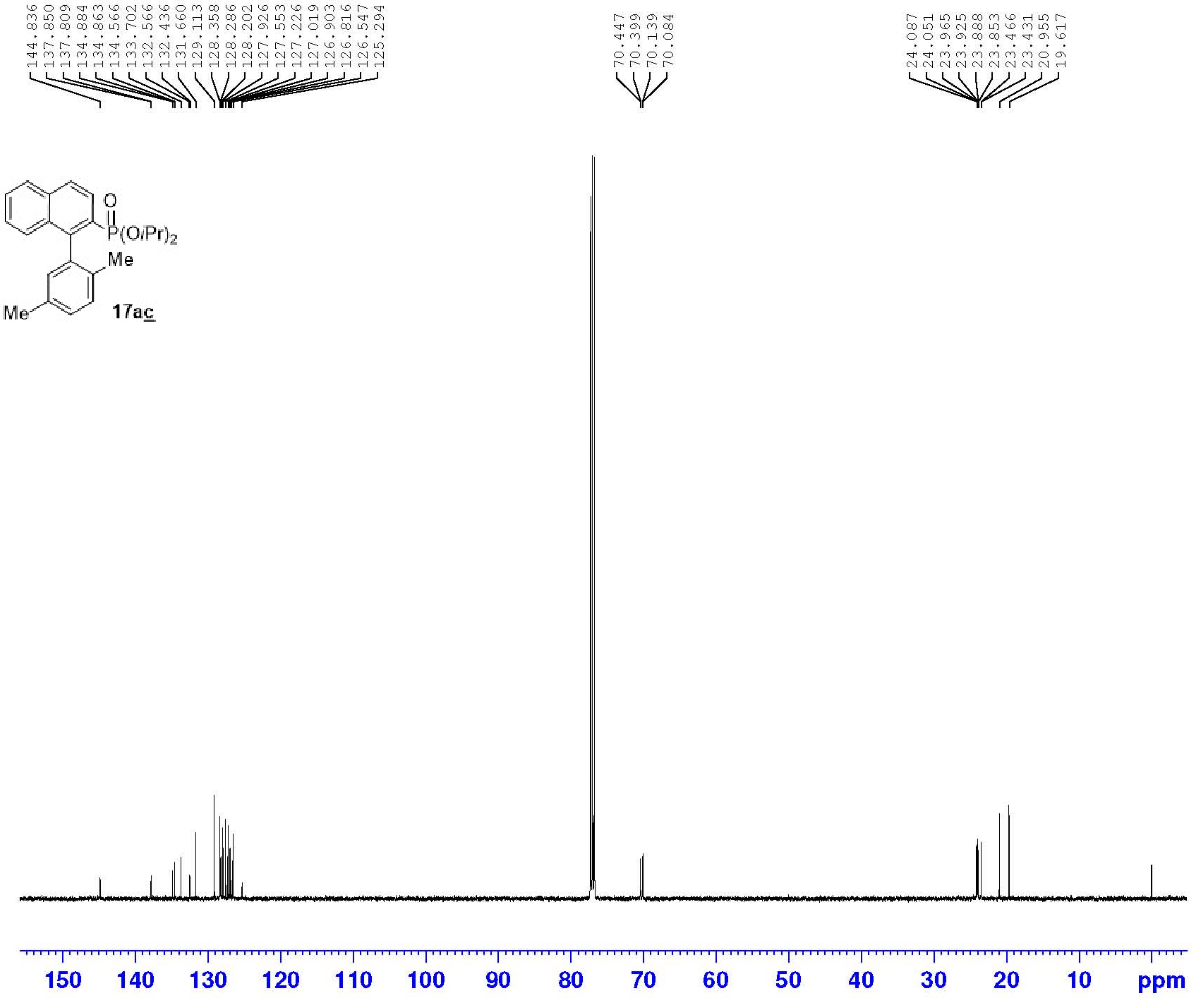

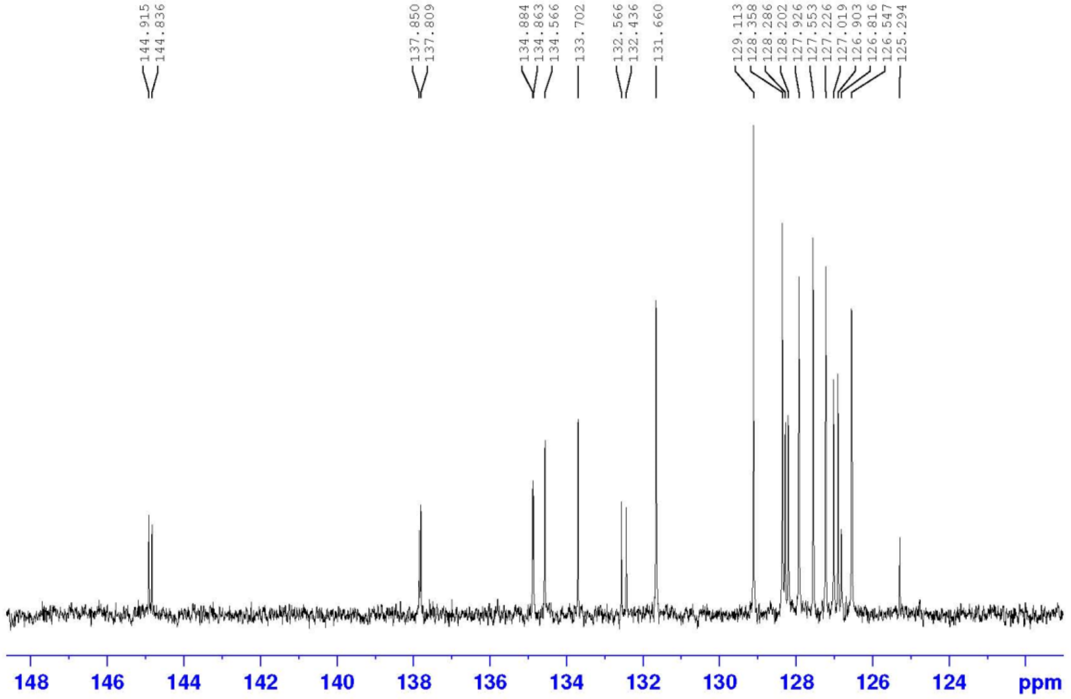

DEPT (125 MHz, $CDCl_3$) spectrum of compound **17a<u>c</u>**

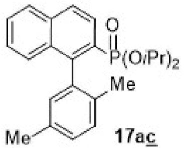

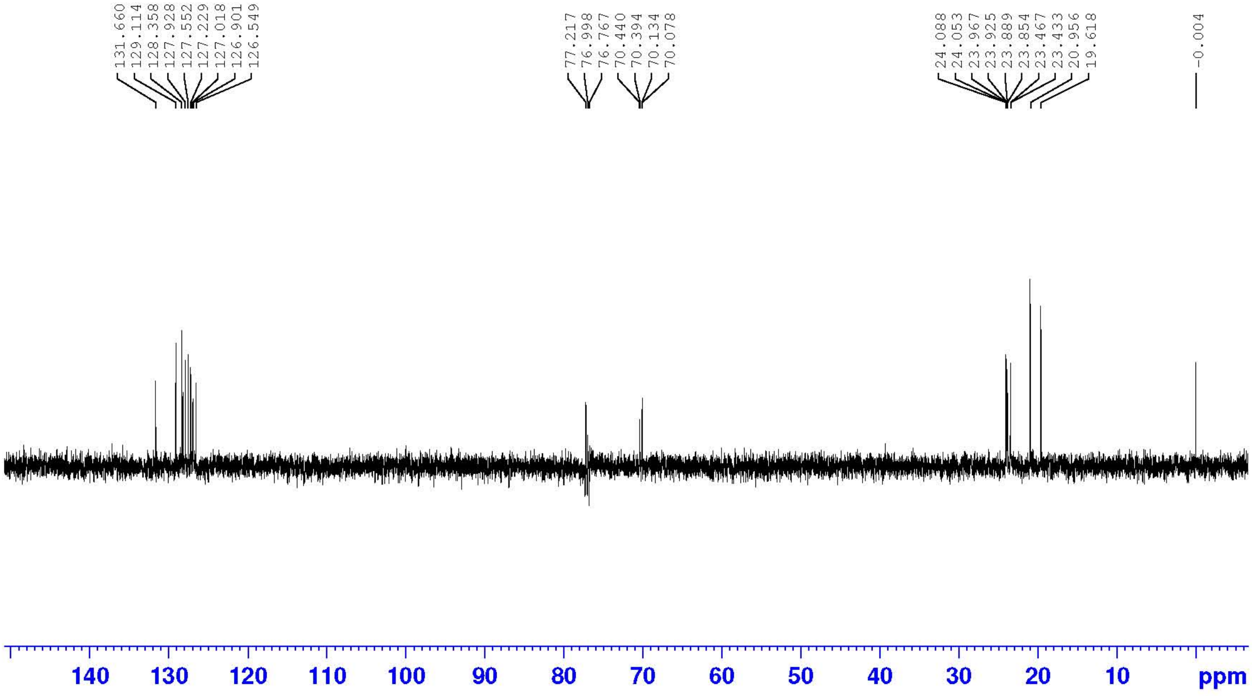

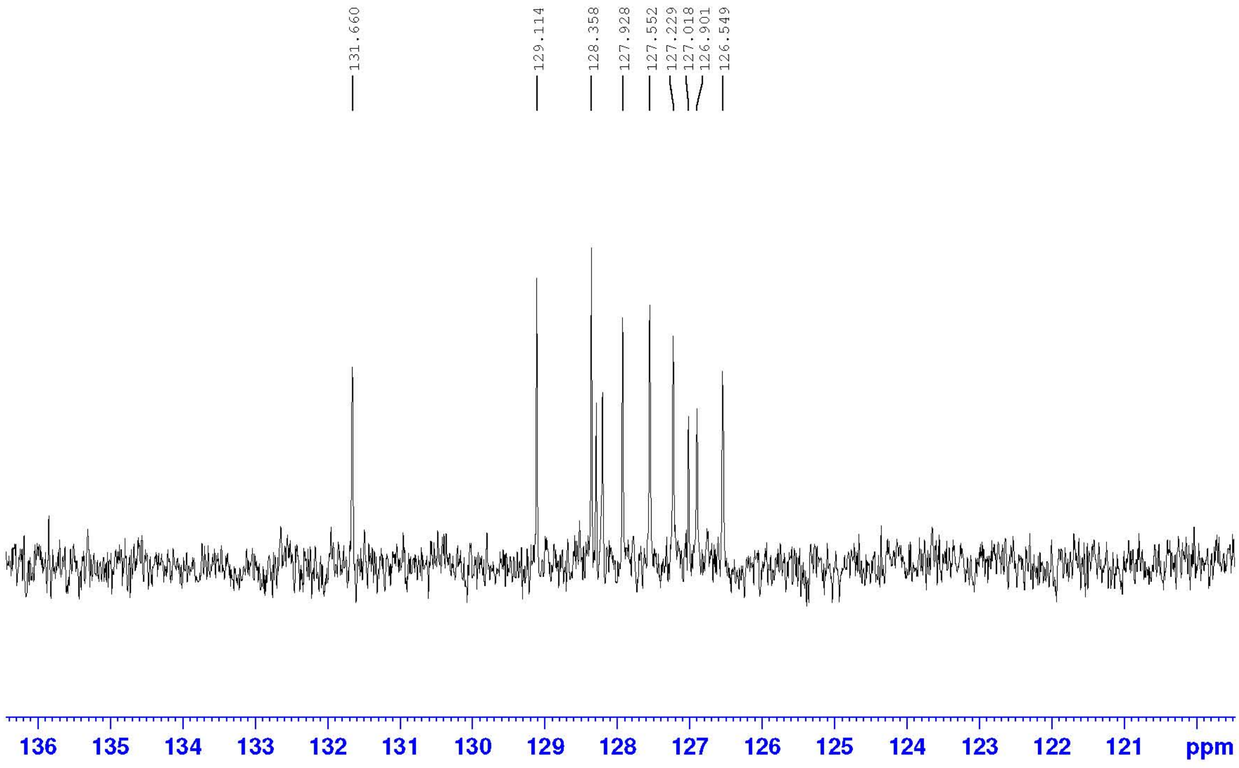

^{31}P NMR (202 MHz, $CDCl_3$) spectrum of compound **17a<u>c</u>**

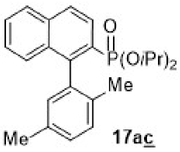

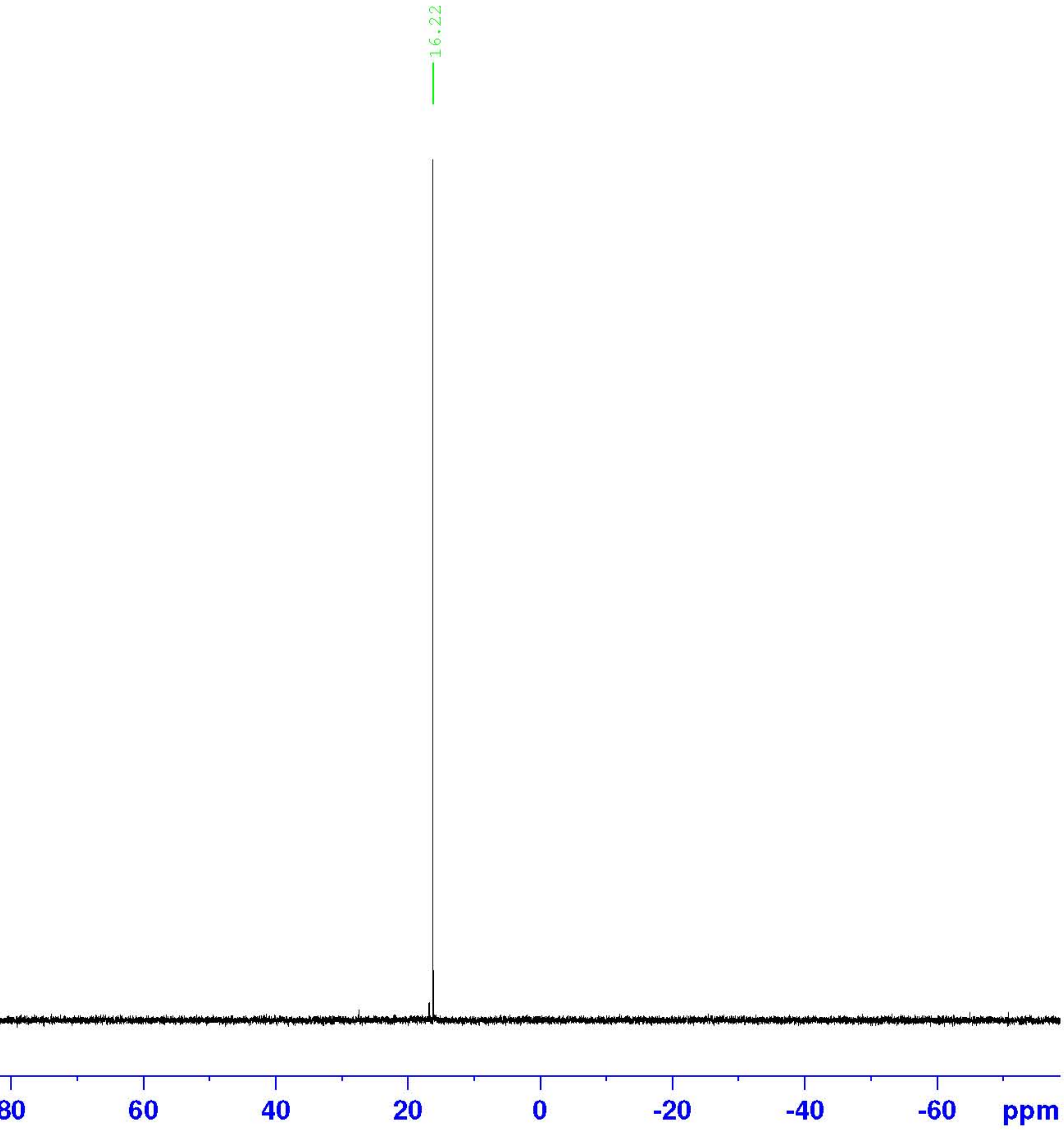

^{1}H NMR (500 MHz, $CDCl_3$) spectrum of compound **17a<u>d</u>**

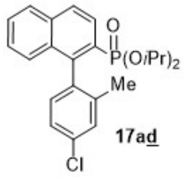

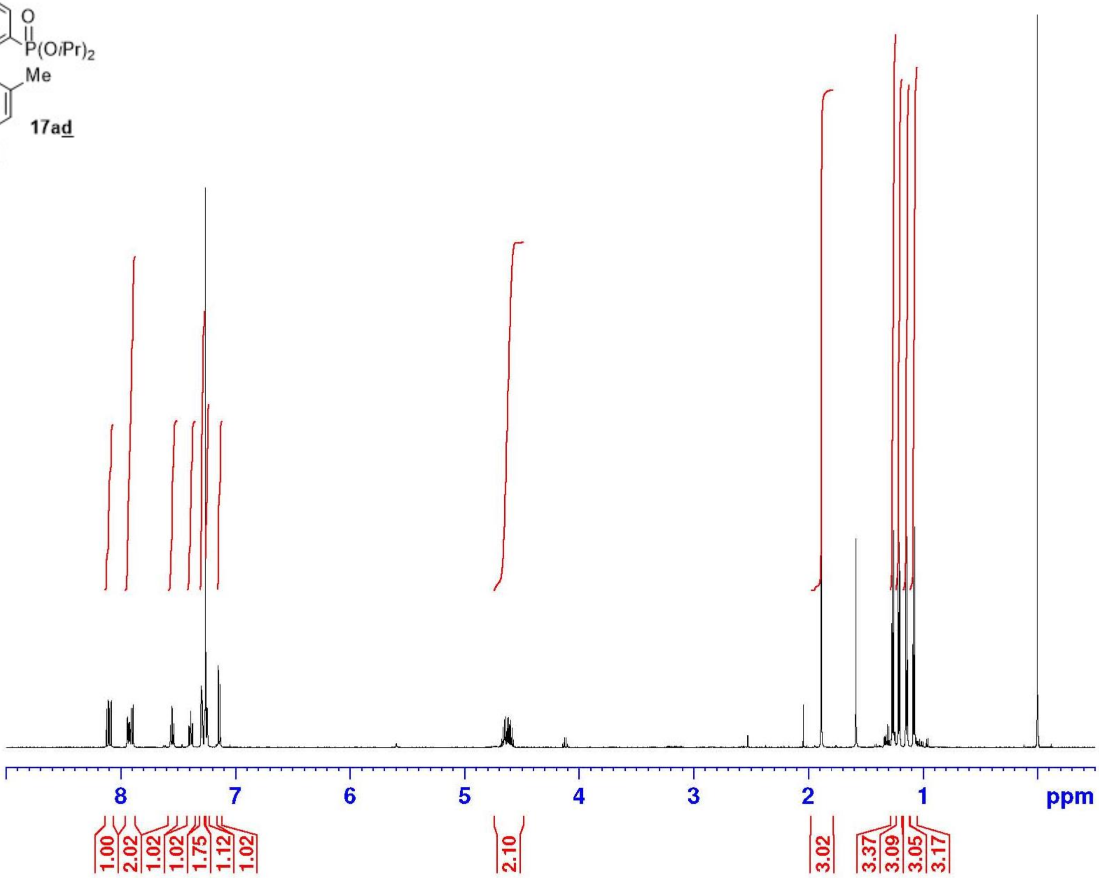

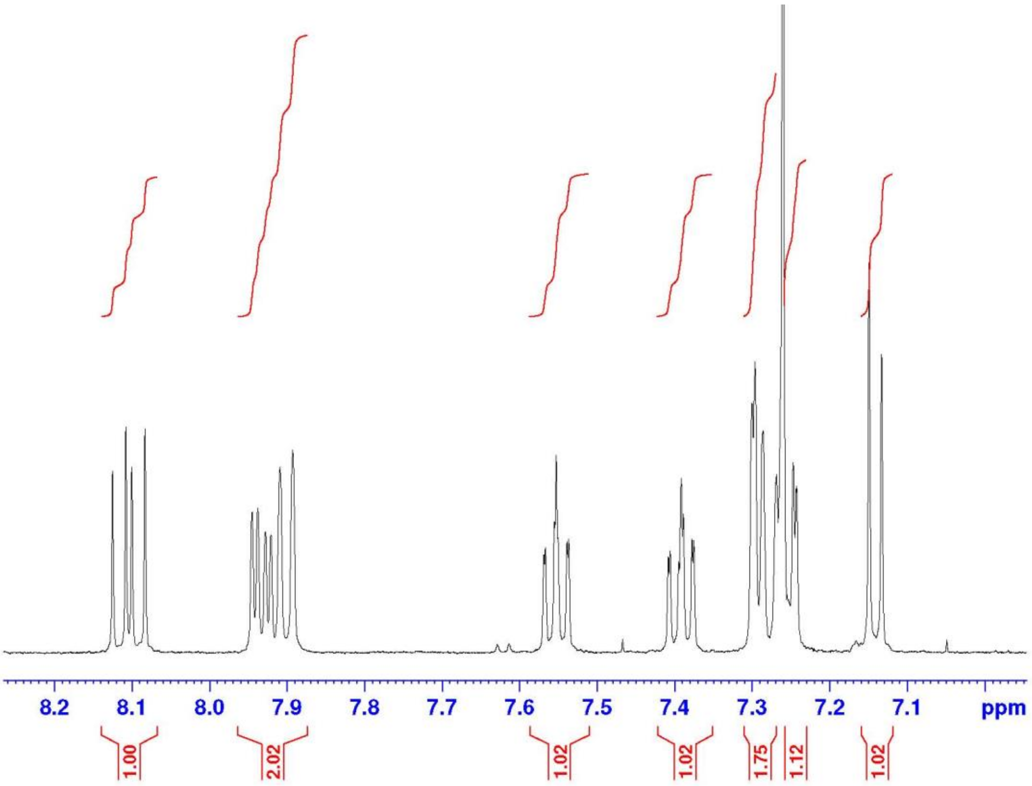

^{13}C NMR (125 MHz, $CDCl_3$) spectrum of compound **17a<u>d</u>**

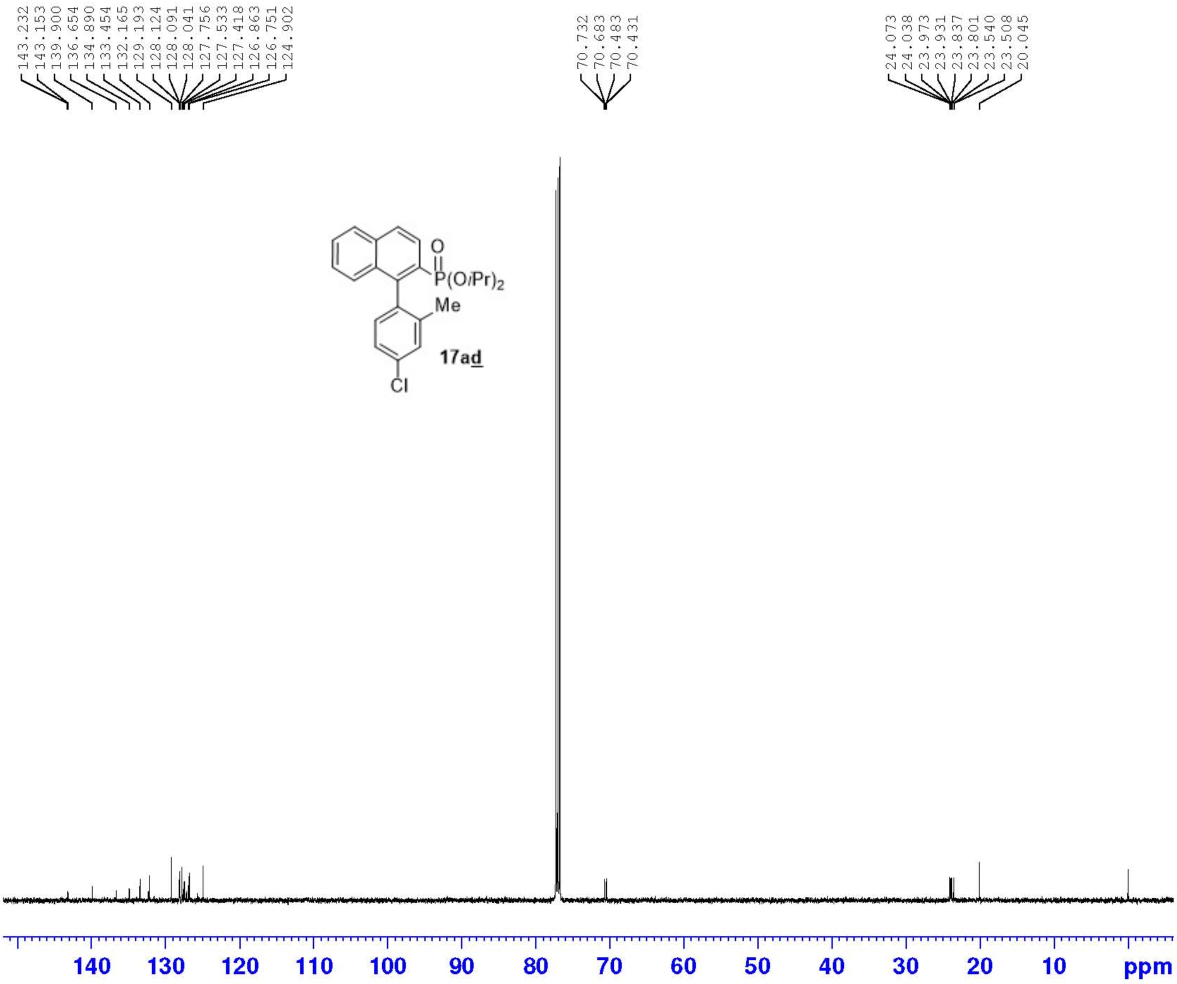

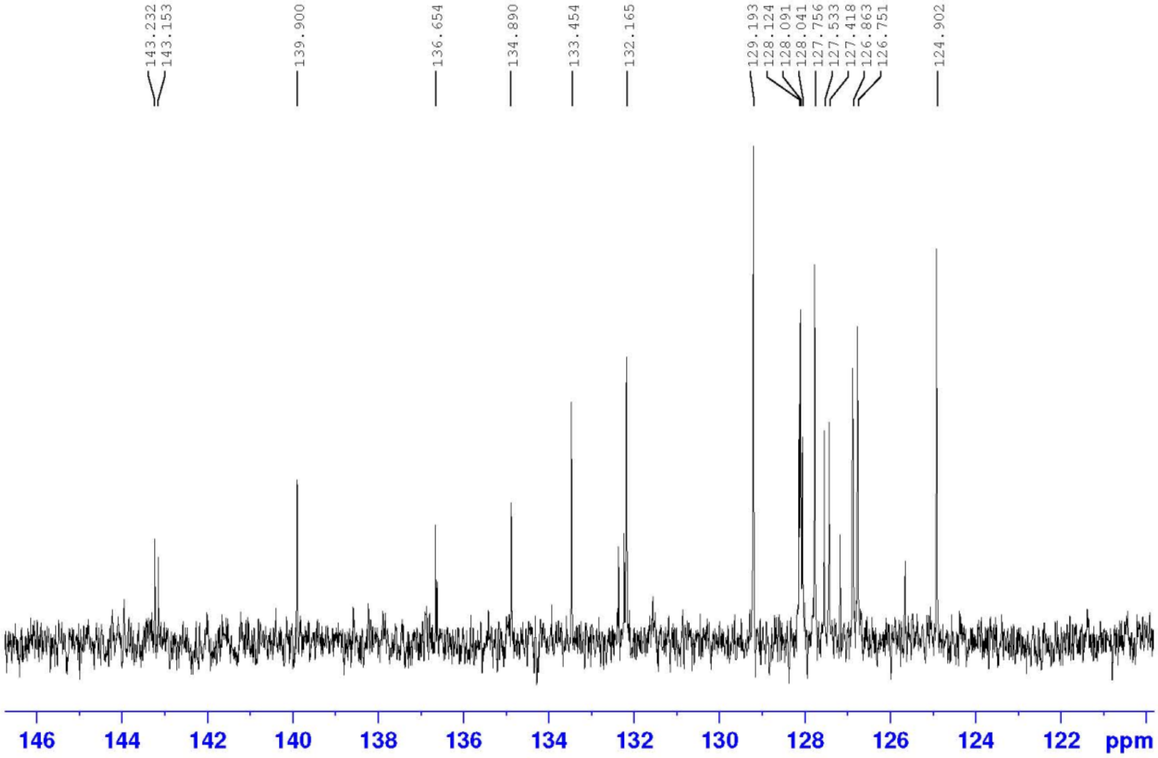

DEPT (125 MHz, $CDCl_3$) spectrum of compound **17a<u>d</u>**

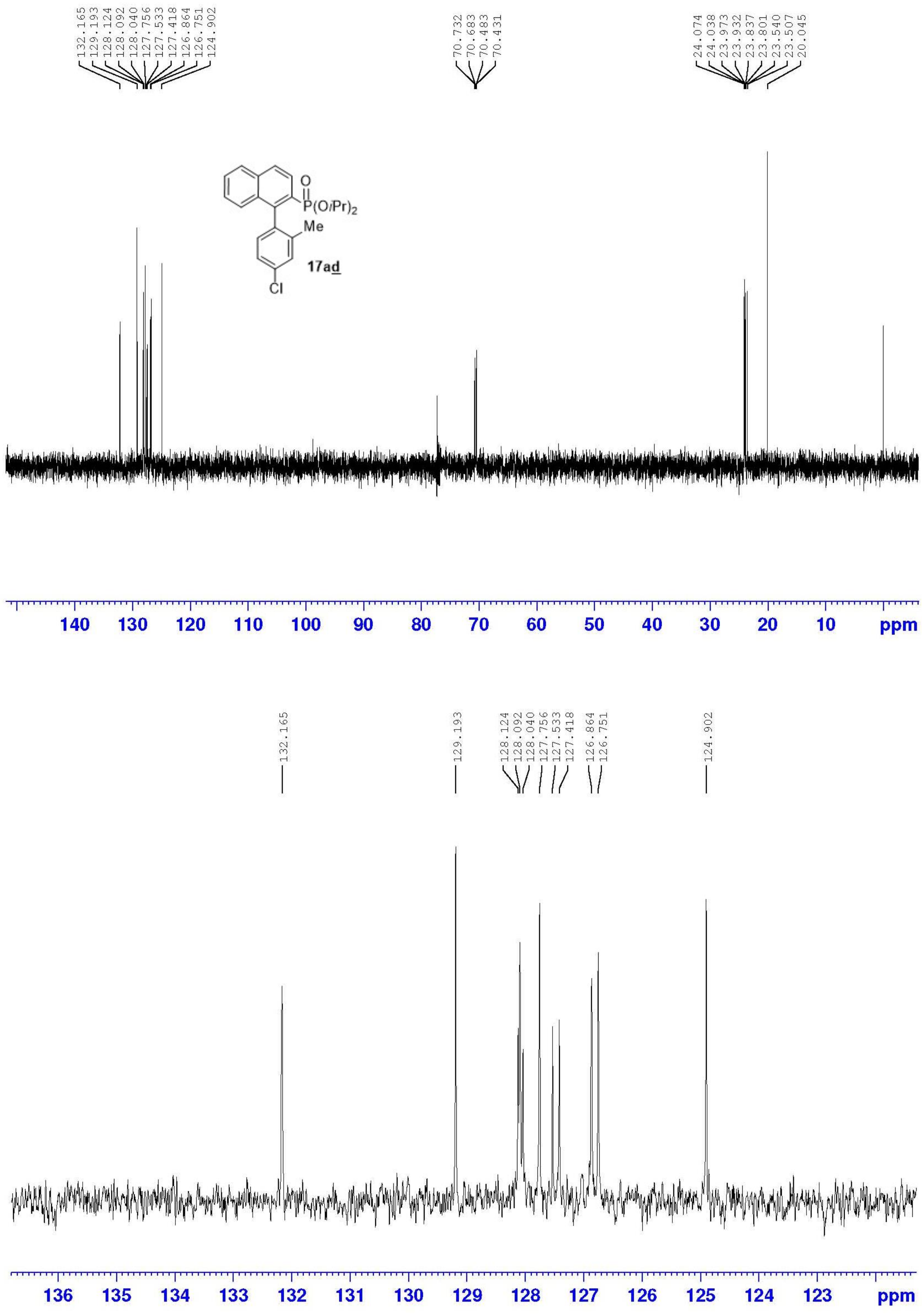

^{31}P NMR (202 MHz, $CDCl_3$) spectrum of compound **17ad**

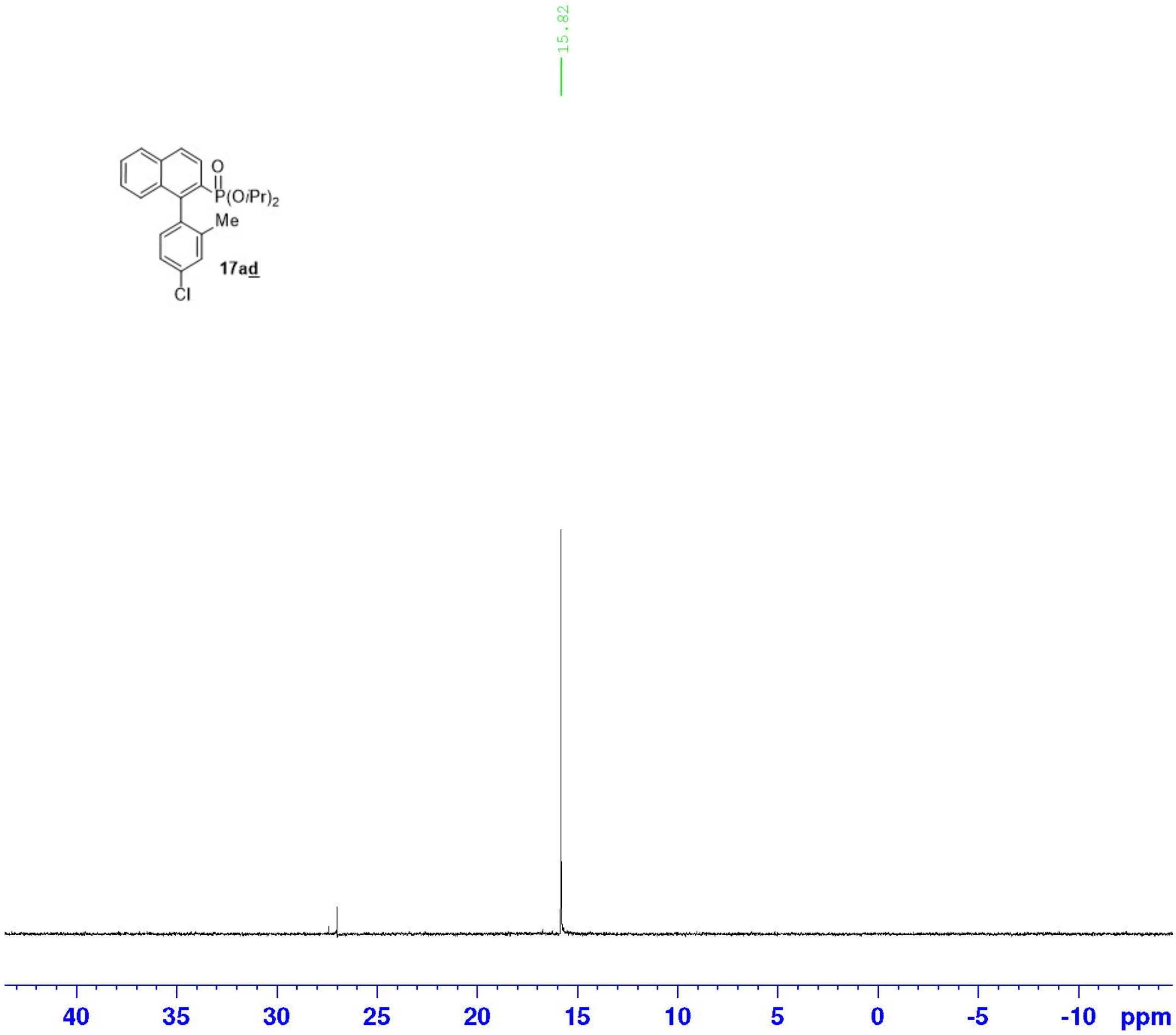

^{1}H NMR (500 MHz, $CDCl_3$) spectrum of compound **17ae**

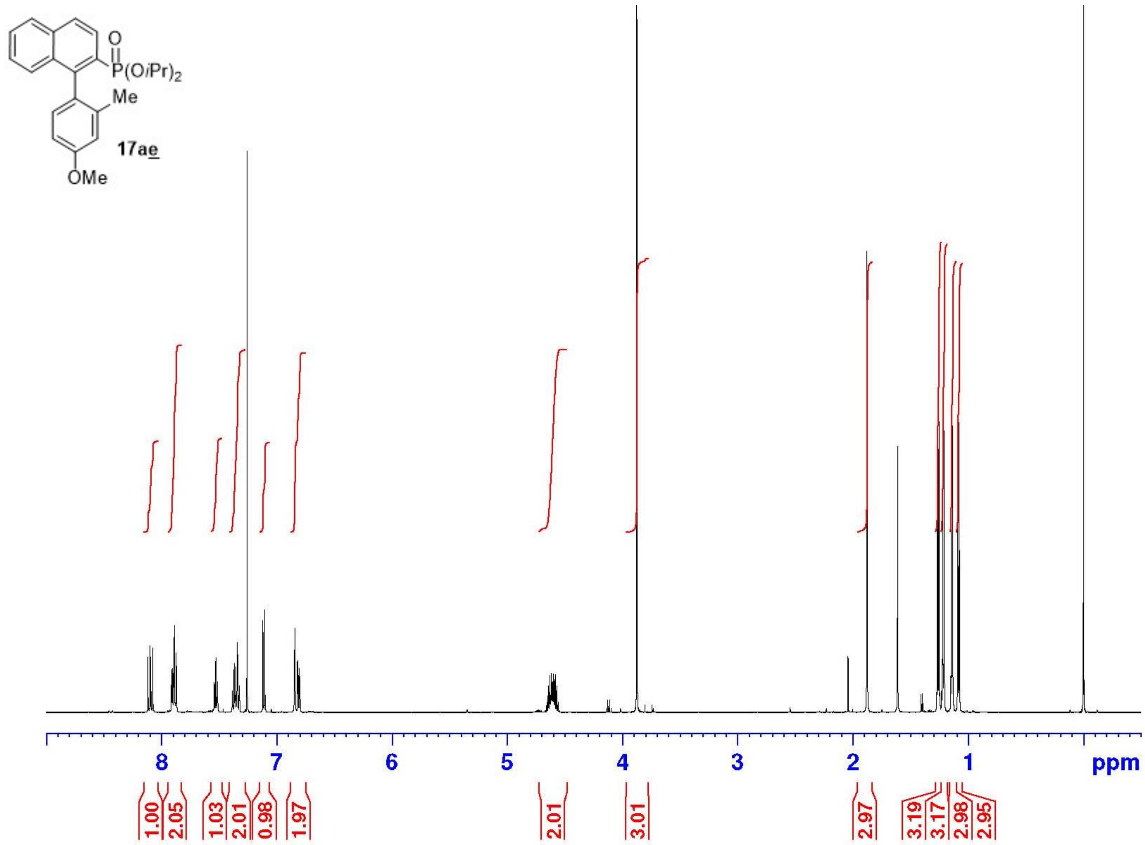

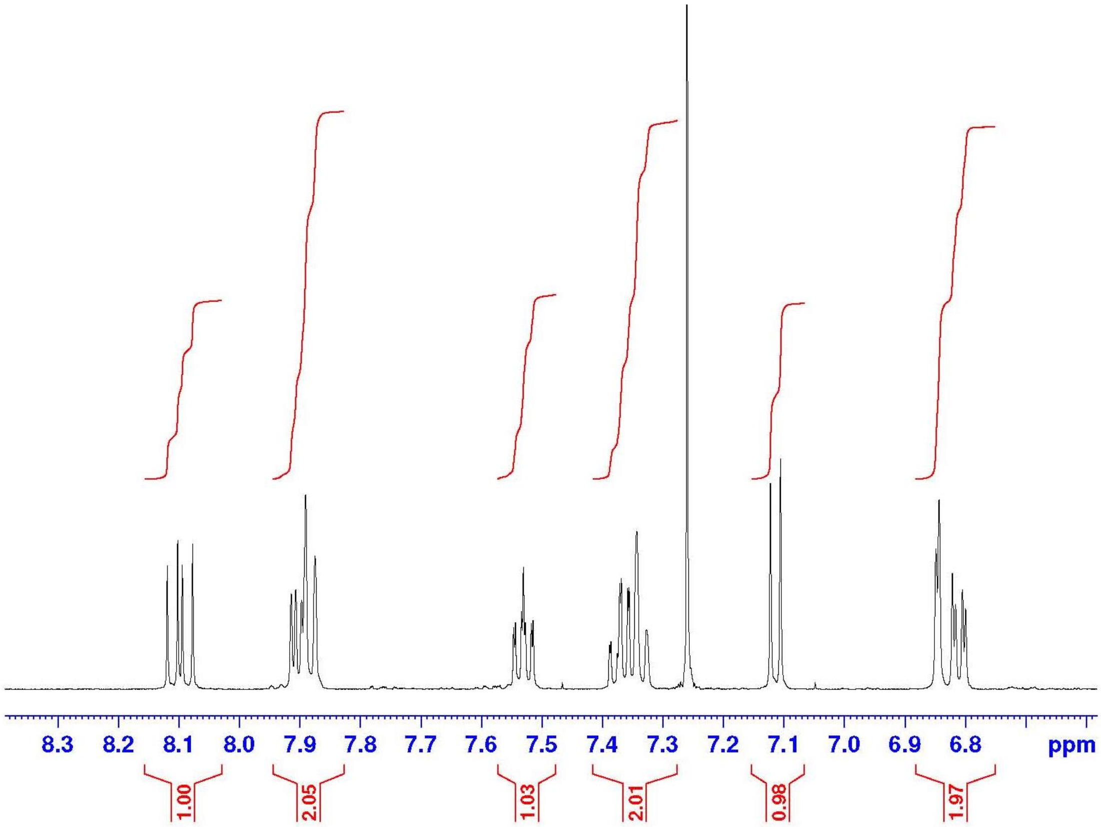

^{13}C NMR (125 MHz, CDCl$_3$) spectrum of compound **17a<u>e</u>**

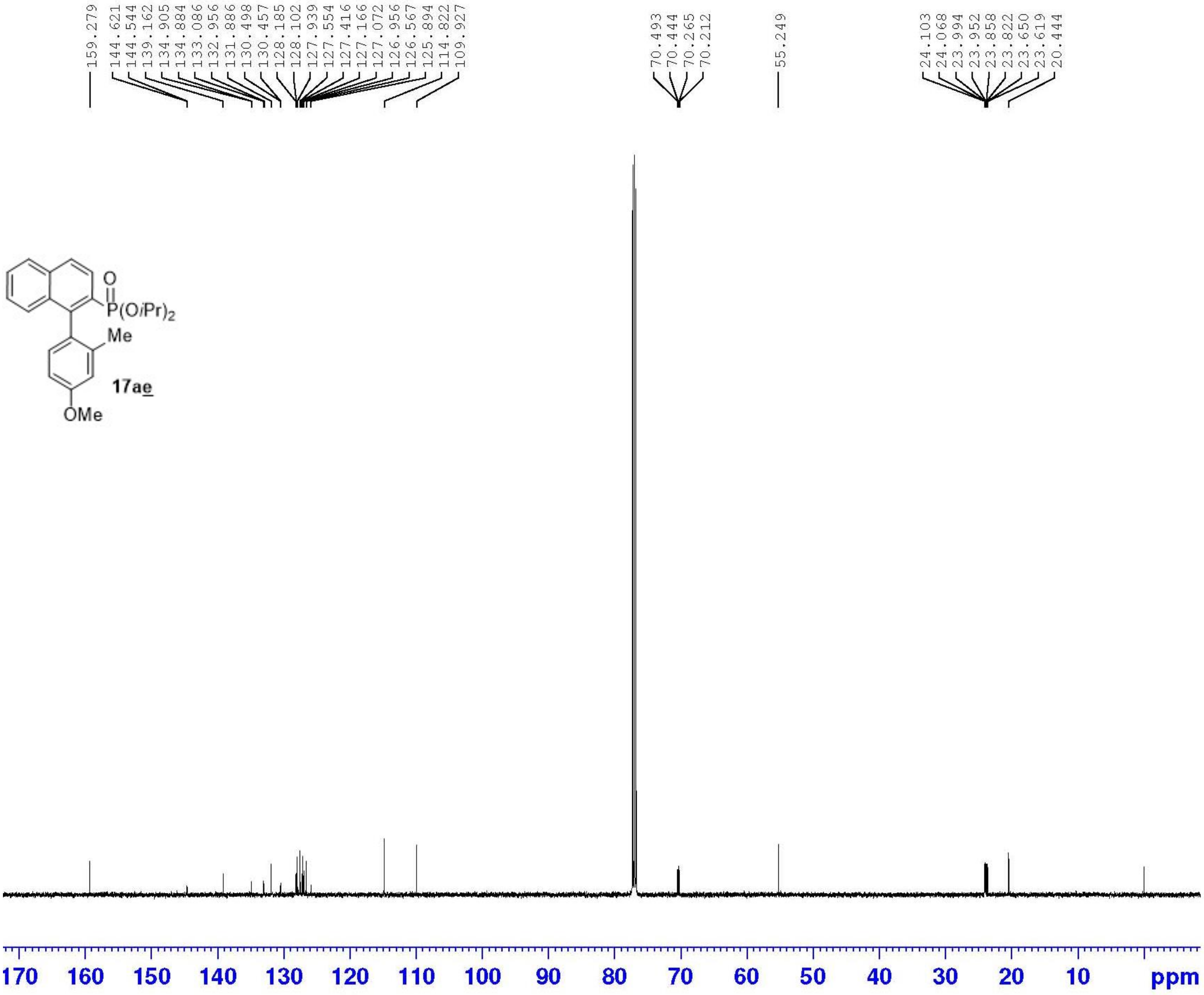

17ae

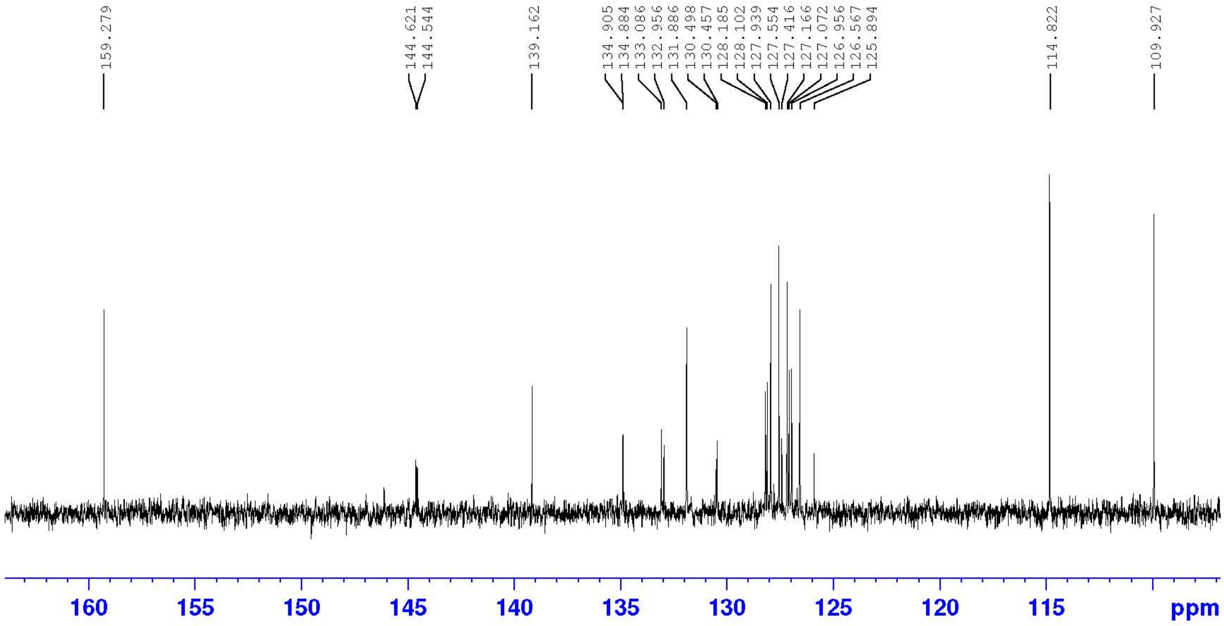

DEPT (125 MHz, $CDCl_3$) spectrum of compound **17a<u>e</u>**

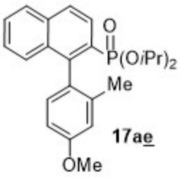

17a<u>e</u>

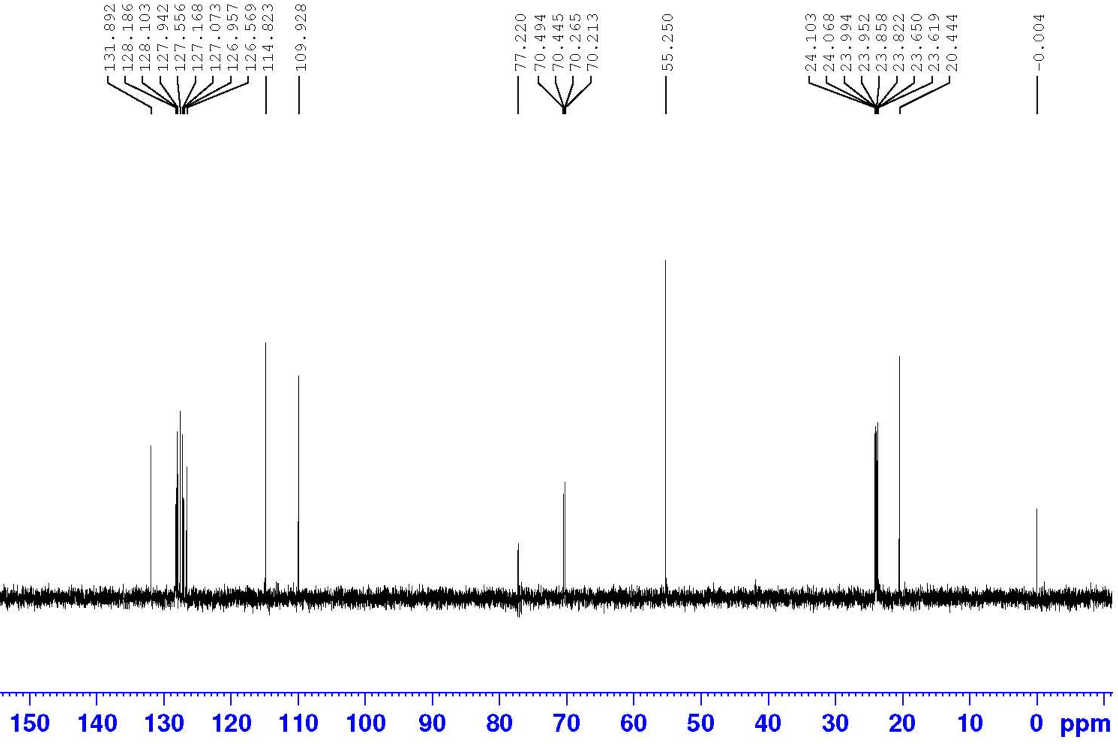

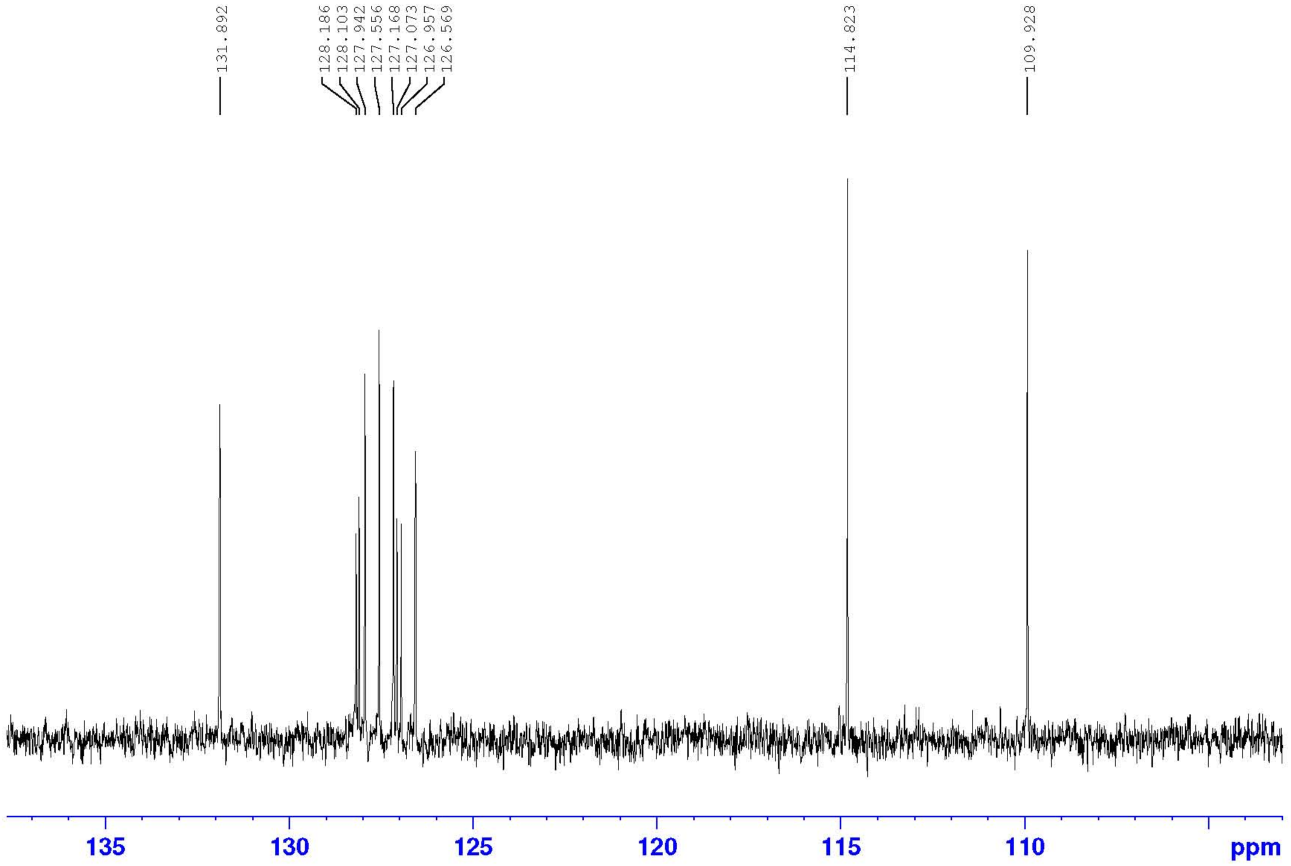

^{31}P NMR (202 MHz, $CDCl_3$) spectrum of compound **17ae**

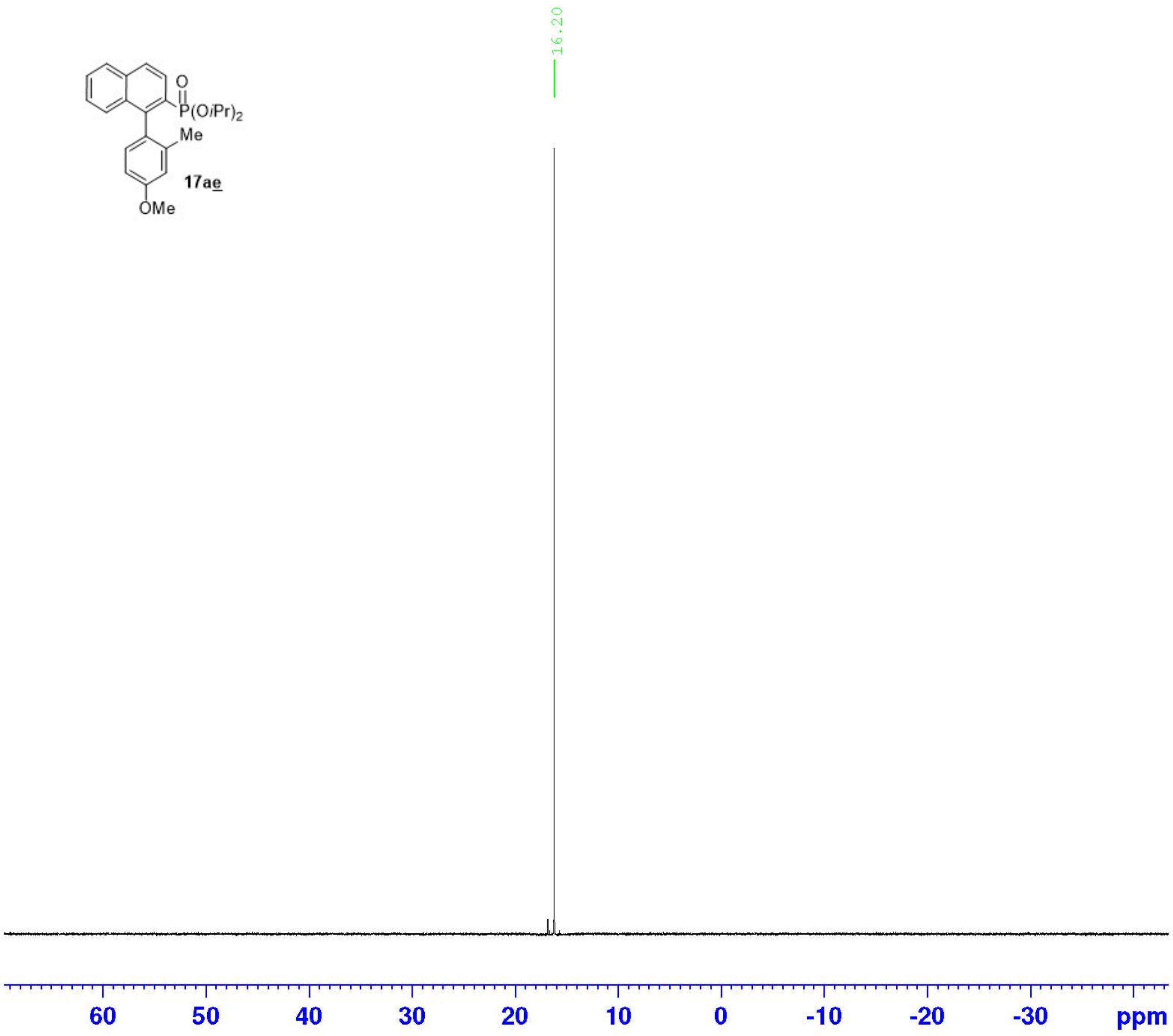

^{1}H NMR (500 MHz, $CDCl_3$) spectrum of compound 17af

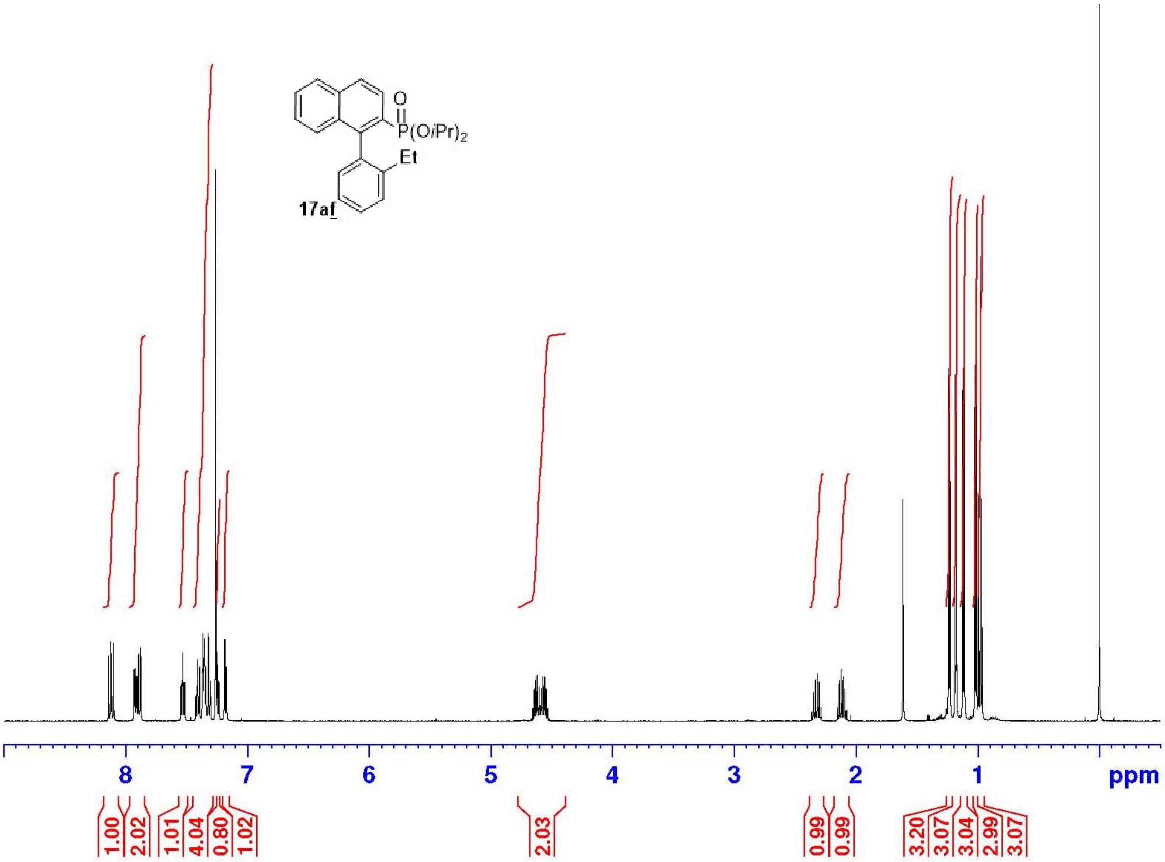

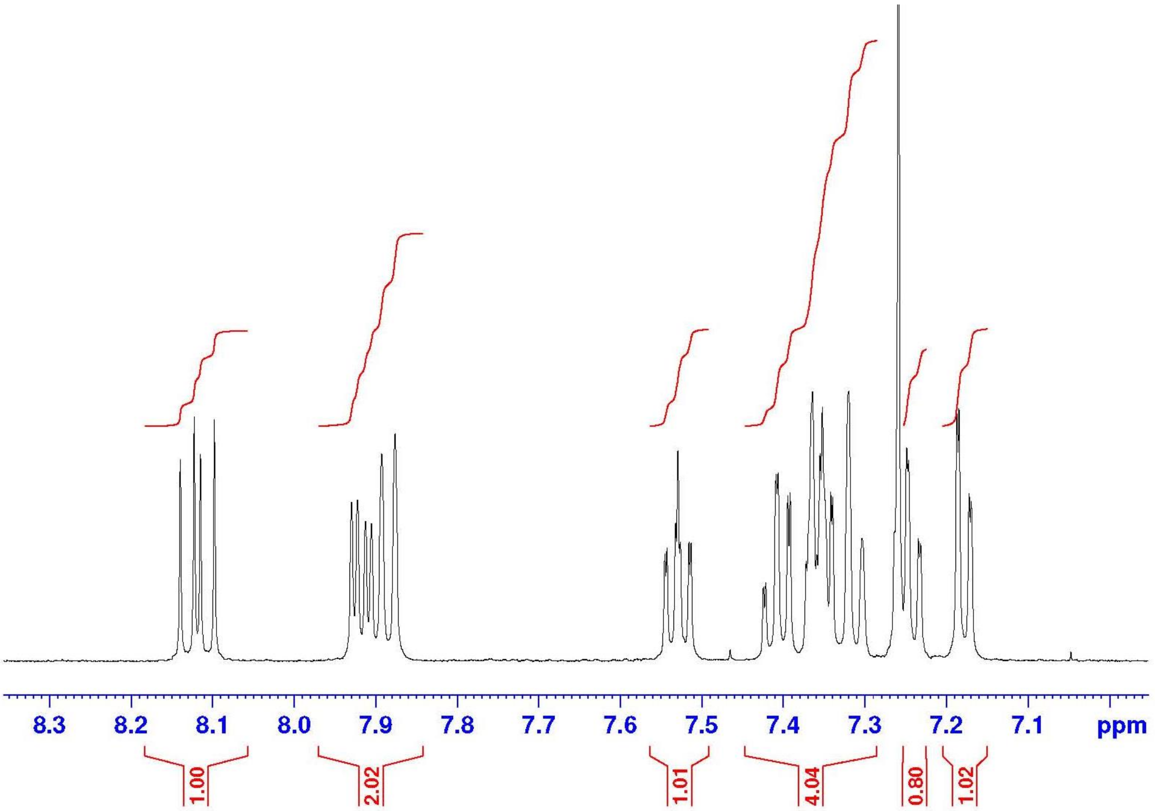

^{13}C NMR (125 MHz, $CDCl_3$) spectrum of compound 17af

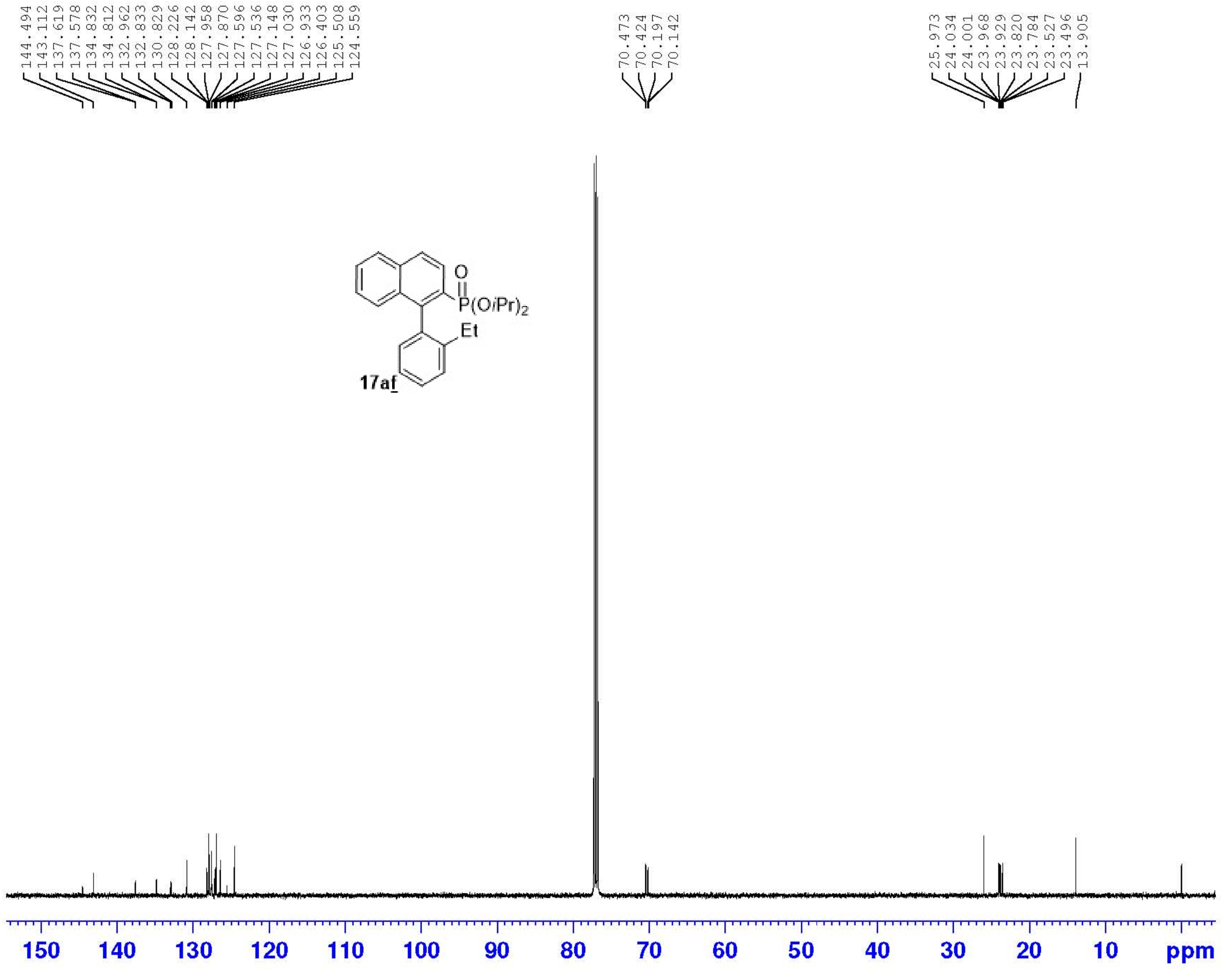

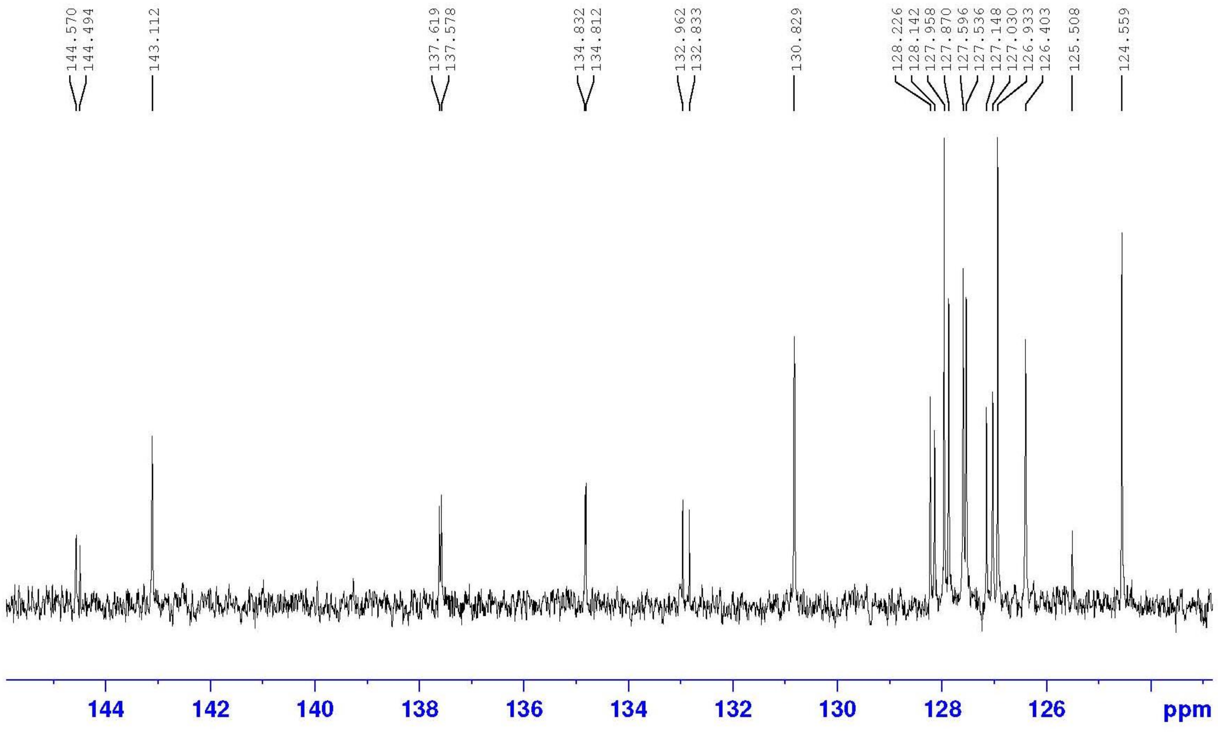

DEPT (125 MHz, $CDCl_3$) spectrum of compound 17af

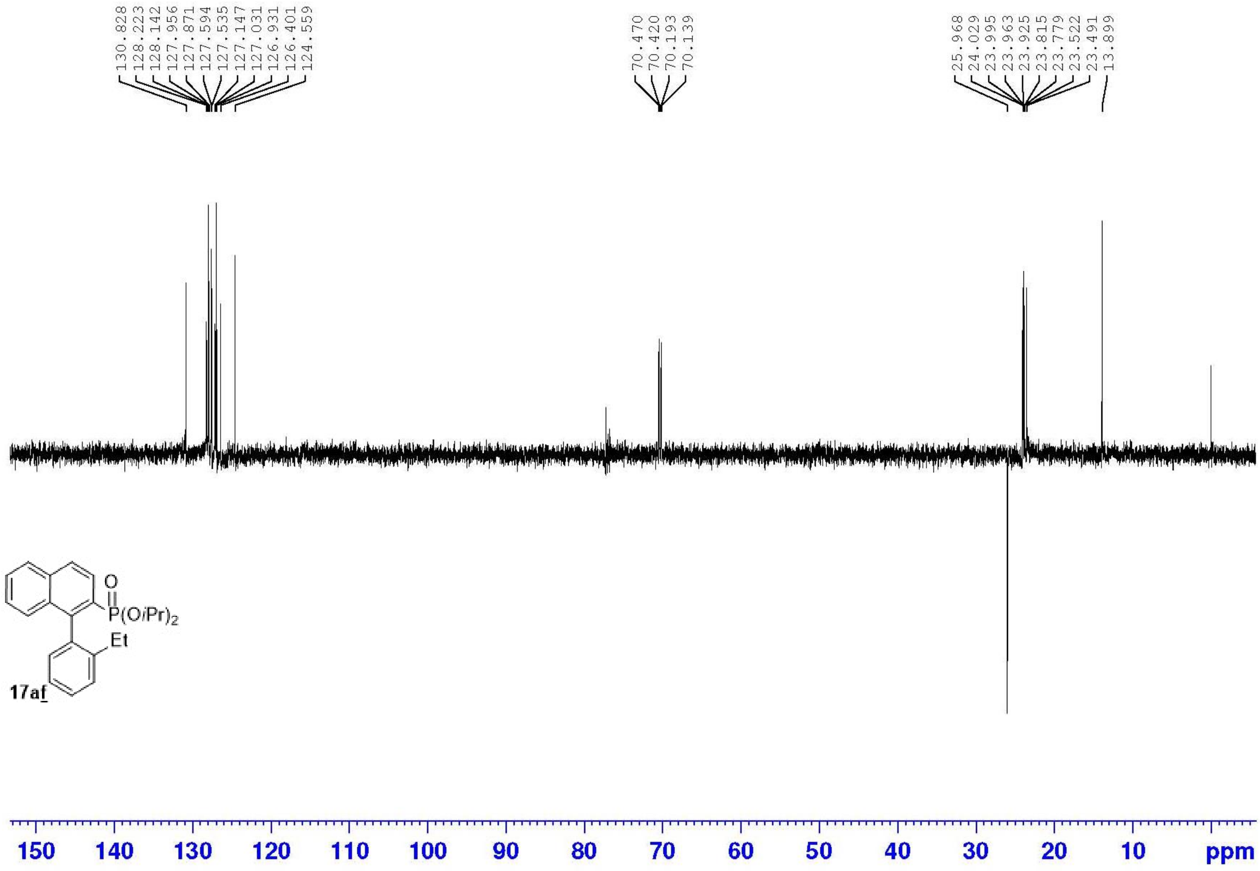

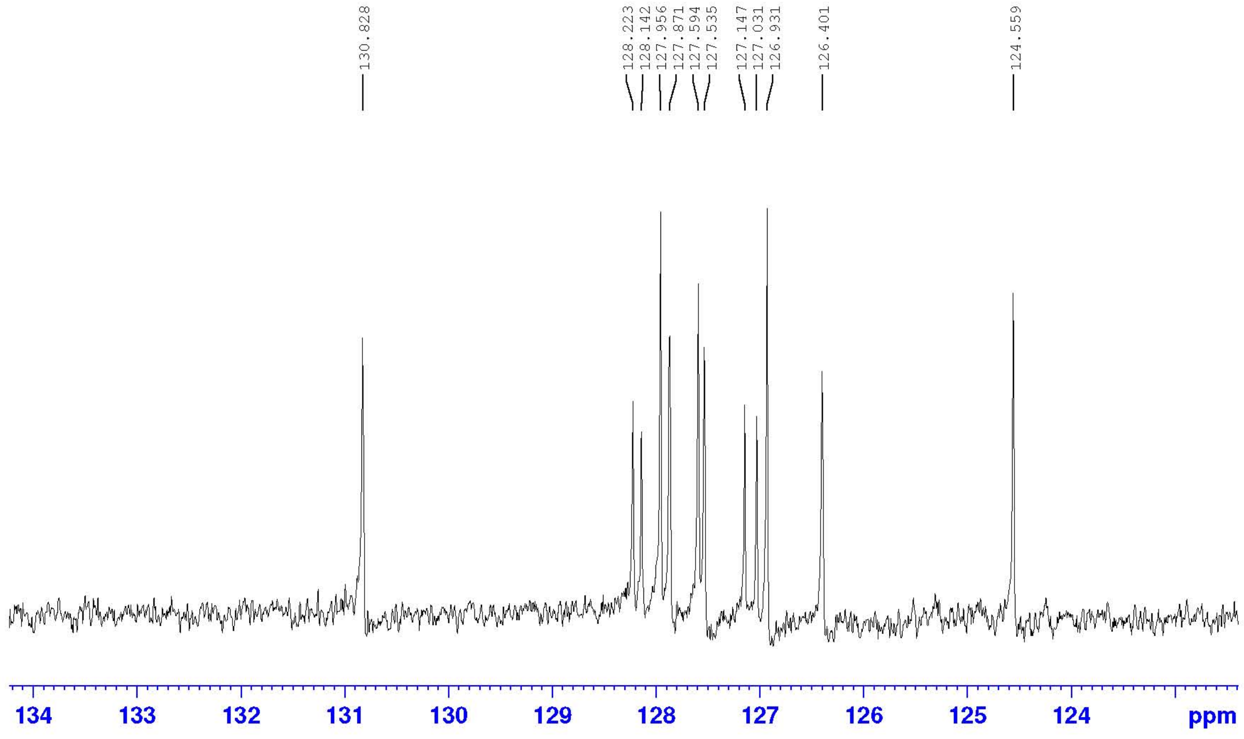

^{31}P NMR (202 MHz, $CDCl_3$) spectrum of compound 17a<u>f</u>

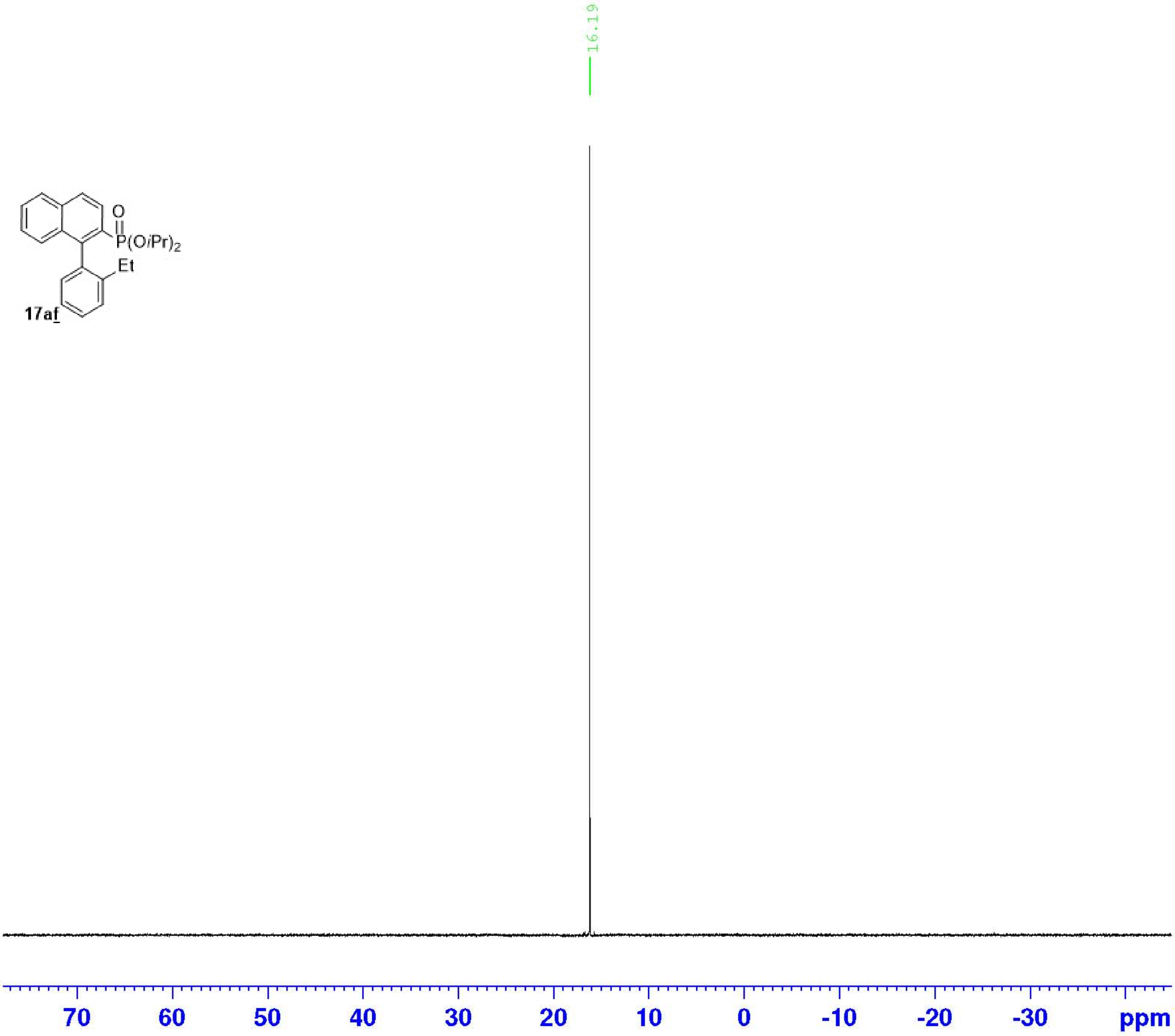

Supplement: Supplementary Information 3 [file srep36211-s3.pdf]
